# Expression-Based Functional Investigation of the Organ-Specific MicroRNAs in *Arabidopsis*

**DOI:** 10.1371/journal.pone.0050870

**Published:** 2012-11-30

**Authors:** Yijun Meng, Chaogang Shao, Xiaoxia Ma, Huizhong Wang, Ming Chen

**Affiliations:** 1 College of Life and Environmental Sciences, Hangzhou Normal University, Hangzhou, People’s Republic of China; 2 College of Life Sciences, Huzhou Teachers College, Huzhou, People’s Republic of China; 3 Department of Bioinformatics, College of Life Sciences, Zhejiang University, Hangzhou, People’s Republic of China; Sun Yat-sen University, China

## Abstract

MicroRNAs (miRNAs) play a pivotal role in plant development. The expression patterns of the miRNA genes significantly influence their regulatory activities. By utilizing small RNA (sRNA) high-throughput sequencing (HTS) data, the miRNA expression patterns were investigated in four organs (flowers, leaves, roots and seedlings) of *Arabidopsis*. Based on a set of criteria, dozens of organ-specific miRNAs were discovered. A dominant portion of the organ-specific miRNAs identified from the ARGONAUTE 4-enriched sRNA HTS libraries were highly expressed in flowers. Additionally, the expression of the precursors of the organ-specific miRNAs was analyzed. Degradome sequencing data-based approach was employed to identify the targets of the organ-specific miRNAs. The miRNA–target interactions were used for network construction. Subnetwork analysis unraveled some novel regulatory cascades, such as the feedback regulation mediated by miR161, the potential self-regulation of the genes *miR172*, *miR396*, *miR398* and *miR860*, and the miR863-guided cleavage of the *SERRATE* transcript. Our bioinformatics survey expanded the organ-specific miRNA–target list in *Arabidopsis*, and could deepen the biological view of the miRNA expression and their regulatory roles.

## Introduction

MicroRNAs (miRNAs), a well-known small RNA (sRNA) species of ∼21 nt (nucleotides), are critical regulators for numerous biological processes in plants [1]–[3]. Most miRNA genes were transcribed by RNA polymerase (Pol) II [4]–[6], resulting in primary transcripts [called primary microRNAs (pri-miRNAs)] with poly(A) (polyadenylation) tails. The pri-miRNAs are further processed into the pre-miRNAs (precursor microRNAs), and then into the miRNA/miRNA* short duplexes through two-step cleavages of Dicer-like 1 (RNase III endonucleases) with the collaboration of SERRATE (a zinc finger protein) [7]–[10] and HYPONASTIC LEAVES 1 (a double-stranded RNA binding protein) [11], [12]. Finally, the functional mature miRNAs are incorporated into the ARGONAUTE (AGO; AGO1 in most cases)-associated microRNA-induced silencing complexes (miRISCs) to repress gene expression transcriptionally or post-transcriptionally (reviewed in [1]–[3]). In addition to the other dynamic factors during miRNA biogenesis, action, and turnover, the expression patterns of the miRNAs could greatly affect or even predetermine their activities [13]. In another word, the expression profiles of the miRNAs provide us with valuable hints for interpreting their biological relevance.

The advent of the high-throughput sequencing (HTS) technology enabled us to detect the sRNA expression levels with unprecedented scale and depth. Moreover, the sequencing-based profiling could discriminate different miRNA family members sharing high sequence identity, which seemed impossible by using the previous hybridization-based technologies. Facilitated by HTS, many studies have been done to interrogate the expression patterns of the miRNAs (or other sRNAs) in different organs/tissues, or under various conditions. A recent study by Wang *et al.* (2011) provided us with a comprehensive data set showing the *in vivo* levels of the sRNAs in four different organs (flowers, leaves, roots and seedlings). Three independent library groups [wild type (WT) plant libraries, AGO1-enriched sRNA libraries, and AGO4-enriched sRNA libraries] were prepared from the four organs separately. Based on these digital expression profiles, the previous study provided us with some novel functional insights into the sRNAs associated with AGO1 and AGO4 [14]. However, the value of these organ-specific expression profiles remains to be fully exploited.

In this study, the organ-specific expression patterns of all the miRBase-registered miRNAs [release 17; a total of 266 miRNA(*)s] of *Arabidopsis* (*Arabidopsis thaliana*) were investigated based on the sRNA HTS data provided by Wang *et al.*’s study (2011) [14]. The organ-specifically expressed miRNAs in WT, AGO1-enriched and AGO4-enriched library groups were extracted separately. Interestingly, a dominant portion of the AGO4-associated miRNAs was specifically expressed in the floral organ. Conservation analysis of the AGO4-enriched miRNAs uncovered some novel sequence features. The organ-specific expression patterns were comparatively analyzed among the mature miRNAs, the pre-miRNAs and the pri-miRNAs. The expression of some miRNA clusters was also investigated. Target prediction and degradome sequencing data-based validation were performed to extract the downstream targets of the organ-specific miRNAs, which were supported by further functional analysis. Based the miRNA–target list, comprehensive gene regulatory networks were constructed. Subnetwork analysis unraveled some intriguing miRNA-mediated regulation in *Arabidopsis*: *miR161*-, *TAS* (*trans*-acting small interfering RNA) gene-, and *PPR* (*pentatricopeptide repeat*) gene-involved regulatory circuits; self-regulation of *miR172*, *miR396*, *miR398* and *miR860*; and miR863-guided cleavage of the *SERRATE* transcript. Conclusively, our large-scale bioinformatics study (please see the analytical workflow in Figure S1) provided a comprehensive list of organ-specific miRNAs and their targets, which could expand the current view of the expression, sequence characteristics, functionalities of the plant miRNAs.

## Results

### Identification of the Organ-specific miRNAs

**Figure 1:**
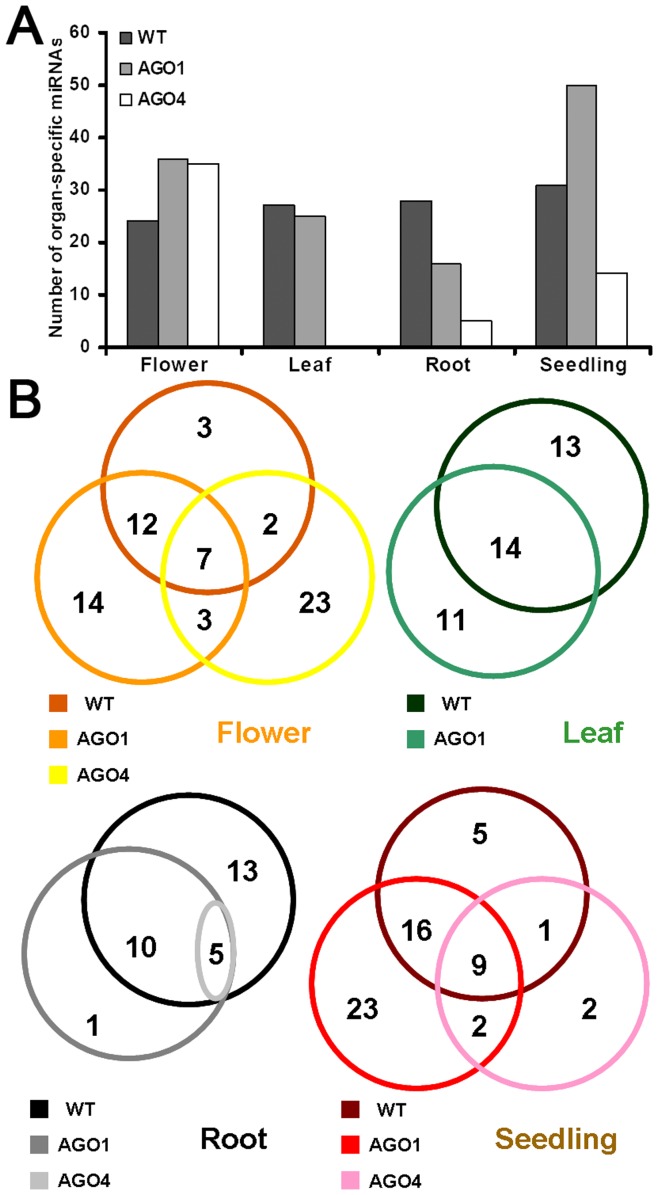
Statistical results of the organ-specific microRNAs in *Arabidopsis*. (**A**) Numbers of the organ-specific (flower, leaf, root or seedling) microRNAs in wild type plants (WT), AGO1 (ARGONAUTE 1), and AGO4. (**B**) Detailed view showing the numbers of the organ-specific microRNAs. All the statistical results were obtained based on the small RNA high-throughput sequencing data (12 data sets; see details in **Materials and Methods**) retrieved from GEO (Gene Expression Omnibus) [68]. See **Materials and Methods** for the definition of the organ-specific microRNAs.

The sRNA HTS data sets provided by Wang *et al*.’s study (2011) were divided into three groups [i.e. the sRNA libraries prepared from wild type plants (WT-related library group), the ones prepared from AGO1-associated sRNA pools (AGO1-related group), and those prepared from AGO4-associated sRNA pools (AGO4-related group); see details in **Materials and Methods**]. Each group consists of four libraries prepared from different organs (flowers, leaves, roots and seedlings). Based on these data sets, the expression patterns of all the miRBase-registered miRNAs of *Arabidopsis* were investigated (Table S1), and the organ-specific miRNAs were extracted by employing certain criteria (see criteria in **Materials and Methods**, and see results in Table S2, Table S3, and Table S4). As a result, 85, 90, and 48 organ-specific miRNAs were identified from the WT-, AGO1-, and AGO4-related library group, respectively (Figure 1A; please note: one miRNA might be identified to be highly expressed in two organs). For the WT group, the organ-specific miRNAs distribute equally among the four organs (24, 27, 28 and 31 in flowers, leaves, roots and seedlings respectively). However, it is not the case for the AGO-related groups. Within the AGO1 group, the number of the seedling-specific miRNAs (50) is much higher than the other organ-specific miRNAs (36, 25 and 16 in flowers, leaves and roots). More interestingly, in the AGO4 group, the number of the flower-specific miRNAs (35) is nearly two times larger than the summed number of the other organ-specific miRNAs (19 in total). Partial overlaps of the organ-specific miRNA populations were observed among the WT, the AGO1, and the AGO4 groups (Figure 1B and Table 1). It indicates that the accumulation levels of the organ-specific miRNAs in the WT plants could only partially reflect their final enrichment in the AGO complexes.

**Table 1:** List of the organ-specific microRNAs in *Arabidopsis*.

	Flower^8^	Leaf^9^	Root^10^	Seedling^11^
**WT** 1	ath-miR319c, ath-miR391, ath-miR859	ath-miR163, ath-miR169a, ath-miR169h,ath-miR169i, ath-miR169j, ath-miR169k,ath-miR169l, ath-miR169m, ath-miR169n,ath-miR172b*, ath-miR391, ath-miR838,ath-miR840	ath-miR169a, ath-miR169h, ath-miR169i,ath-miR169j, ath-miR169k, ath-miR169l,ath-miR169m, ath-miR169n, ath-miR5028,ath-miR773, ath-miR824, ath-miR837-3p,ath-miR837-5p	ath-miR2111a*, ath-miR2111b*, ath-miR398b,ath-miR398c, ath-miR837-3p
**AGO1** 2	ath-miR170, ath-miR172e, ath-miR2934-5p,ath-miR397a, ath-miR397b, ath-miR399a,ath-miR399b, ath-miR399c, ath-miR399d,ath-miR399f, ath-miR832-3p, ath-miR845b,ath-miR857, ath-miR868-5p	ath-miR159b, ath-miR159c, ath-miR395a,ath-miR395b, ath-miR395c, ath-miR395d,ath-miR395e, ath-miR395f, ath-miR396a,ath-miR398a, ath-miR830*	ath-miR860	ath-miR158a, ath-miR158b, ath-miR164c,ath-miR169a, ath-miR170, ath-miR171a,ath-miR390a, ath-miR390b, ath-miR397a,ath-miR775, ath-miR825, ath-miR829.2,ath-miR833-3p, ath-miR838, ath-miR841b,ath-miR842, ath-miR843, ath-miR845a,ath-miR847, ath-miR848, ath-miR850,ath-miR862-5p, ath-miR869.2
**AGO4** 3	ath-miR159a, ath-miR161.2, ath-miR162a,ath-miR162b, ath-miR163, ath-miR164a,ath-miR164b, ath-miR165a, ath-miR165b,ath-miR166a, ath-miR166b, ath-miR166c,ath-miR166d, ath-miR166e, ath-miR166f,ath-miR166g, ath-miR167a, ath-miR167b,ath-miR390a, ath-miR390b, ath-miR393a,ath-miR393b, ath-miR396b	–	–	ath-miR167a, ath-miR167b
**WT & AGO1** 4	ath-miR156h, ath-miR447a, ath-miR447b,ath-miR5017, ath-miR771, ath-miR780.1,ath-miR839, ath-miR845a, ath-miR851-5p,ath-miR856, ath-miR858, ath-miR867	ath-miR157a, ath-miR157b, ath-miR157c,ath-miR157d, ath-miR167d, ath-miR400,ath-miR5026, ath-miR825, ath-miR828,ath-miR841,ath-miR843,ath-miR847,ath-miR858,ath-miR863-3p	ath-miR167c, ath-miR169b, ath-miR169c,ath-miR172e, ath-miR395b, ath-miR395c,ath-miR395f, ath-miR829.2, ath-miR842,ath-miR869.2	ath-miR157d, ath-miR167c, ath-miR167d,ath-miR169b, ath-miR169c, ath-miR397b,ath-miR399a, ath-miR399b, ath-miR399c,ath-miR399d, ath-miR399f, ath-miR5026,ath-miR841, ath-miR857, ath-miR860,ath-miR863-3p
**WT & AGO4** 5	ath-miR408, ath-miR447a.2	–	–	ath-miR408
**AGO1 & AGO4** 6	ath-miR171a, ath-miR172a, ath-miR172b	–	–	ath-miR161.2, ath-miR400
**WT & AGO1 & AGO4** 7	ath-miR172c, ath-miR172d, ath-miR319a,ath-miR319b, ath-miR394a, ath-miR394b,ath-miR780.2	–	ath-miR172c, ath-miR172d, ath-miR822,ath-miR829.1, ath-miR846	ath-miR156a, ath-miR156b, ath-miR156c,ath-miR156d, ath-miR156e, ath-miR156f,ath-miR157a, ath-miR157b, ath-miR157c

1 Organ-specific microRNAs (miRNAs) identified from the WT-related library group.

2 Organ-specific miRNAs identified from the AGO1-related library group.

3 Organ-specific miRNAs identified from the AGO4-related library group.

4 Organ-specific miRNAs in both the WT- and the AGO1-related groups.

5 Organ-specific miRNAs in both the WT- and the AGO4-related groups.

6 Organ-specific miRNAs in both the AGO1- and the AGO4-related groups.

7 Organ-specific miRNAs in the WT-, the AGO1- and the AGO4-related groups.

8 Flower-specific miRNAs.

9 Leaf-specific miRNAs.

10 Root-specific miRNAs.

11 Seedling-specific miRNAs.

Please refer to **Materials and Methods** for the definition of the WT-, the AGO1- and the AGO4-related library groups.

See **Materials and Methods** for the identification of the organ-specific miRNAs.

**Figure 2:**
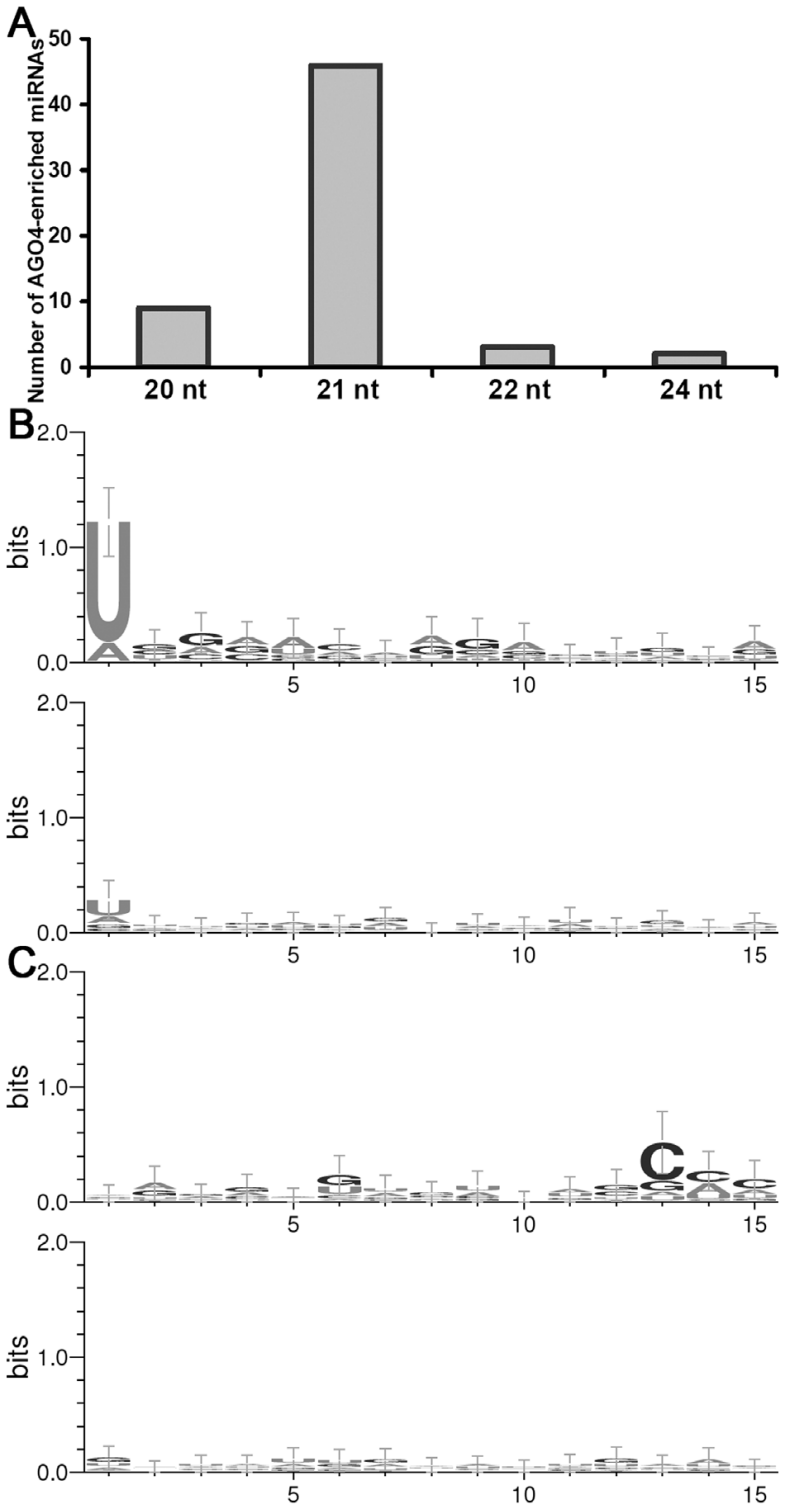
Sequence characterization of the AGO4 (ARGONAUTE 4)-enriched microRNAs. Based on the small RNA high-throughput sequencing data (four data sets belonging to the AGO4-related library group; see details in **Materials and Methods**), the microRNAs with normalized read counts higher than 10 RPM (reads per million) in either AGO4-related library were considered to be enriched in AGO4 (see detailed list in Table S5). (**A**) Sequence length distribution of the AGO4-enriched microRNAs. (**B**) Sequence conservation analysis. The 15-nt sequences from the 5′ ends of all the AGO4-enriched microRNAs were analyzed together (upper panel). The lower panel shows the result of the 5′ 15-nt sequences of the control set. (**C**) Sequence conservation analysis. The 15-nt sequences from the 3′ ends of all the AGO4-enriched microRNAs were analyzed (upper panel). The lower panel shows the result of the 3′ 15-nt sequences of the control set. For (**B**) and (**C**), the microRNAs that could not be detected in any AGO4-related libraries were treated as the control set (Table S6). The sequence logos were generated by WebLogo 3.^73.^

Previous results showed that AGO4 preferentially recruited 24-nt sRNAs with 5′ A (adenine) [15], which participated in epigenetic silencing of repeated loci and heterochromatin regions [16]. Here, we analyzed the sequence features of the AGO4-eriched miRNAs. The miRNAs with normalized read counts higher than 10 RPM (reads per million) in either of the AGO4-related libraries were considered to be AGO4-enriched (Table S5). The result shows that the AGO4-enriched miRNAs are predominantly 21 nt in length (Figure 2A). Compared to the miRNAs not detected in either of the AGO4-related libraries (see list in Table S6), the 5′ terminals of all the AGO4-enriched miRNAs are more frequently occupied by U (uridine) (Figure 2B). Besides, the third nucleotides at the 3′ ends of the AGO4-enriched miRNAs are slightly enriched in C (cytosine) (Figure 2C).

### The Expression Patterns of the miRNA Gene Products

**Figure 3:**
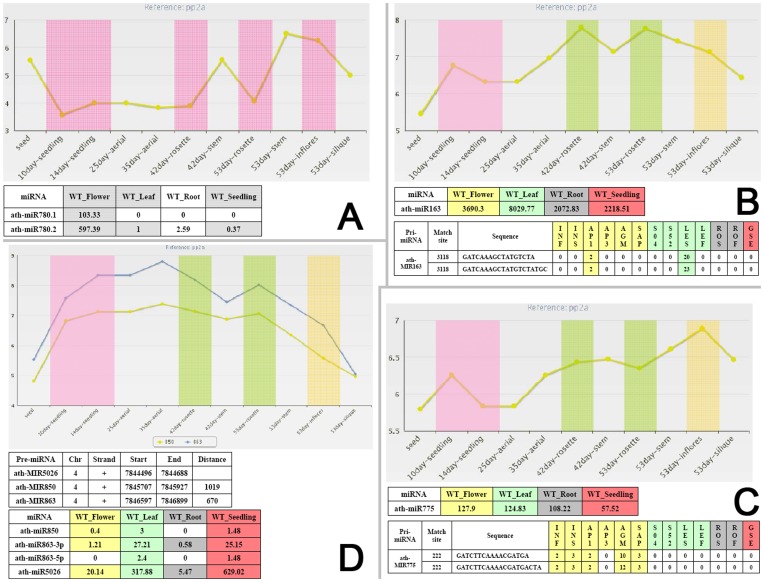
Expression pattern-based comparison between the mature microRNAs (miRNAs) and the miRNA precursors. (**A**) Expression patterns of the mature miRNAs, ath-miR780.1 and ath-miR780.2, and the corresponding pre-miRNA (precursor microRNA)/pri-miRNA (primary microRNA). For the mature miRNAs listed in the table, their detectable expression levels (in RPM; reads per million) in flowers, leaves and seedlings were highlighted in gray background based on the small RNA (sRNA) high-throughput sequencing (HTS) data. Accordingly, the mirEX-derived expression levels of the pre-miRNA/pri-miRNA detected by real-time quantitative PCR [*PP2A* (*phosphatase 2A*; *AT1G13320*) as the reference gene] in the similar organs were highlighted in pink background. (**B**) Expression patterns of the mature miRNA ath-miR163 and its precursors. For the mature miRNA listed in the first table, the detectable expression levels in flowers, leaves, roots and seedlings, based on the sRNA HTS data, were highlighted in different background colors. For the pri-miRNA in the second table, the expression levels of the identified poly(A) signals based on the MPSS (massively parallel signature sequencing) data were highlighted by the same background colors as the above table according to the organ-specific origination of the MPSS libraries. “Match site” means the location of the 5′ end of the “Sequence” within the downstream region of the pre-miRNA. Two sequences indicating the potential poly(A) signal of the *miR163* gene were identified from two independent MPSS data sets. The expression levels of the pre-miRNA/pri-miRNA detected by real-time quantitative PCR (*PP2A* as the reference gene) in different organs were highlighted similar background colors as the tables. (**C**) Expression patterns of the mature miRNA ath-miR166a and its precursors. The meanings of the expression data have been introduced in (**B**). (**D**) Expression patterns of the mature miRNAs, ath-miR850, ath-miR863-3p, ath-miR863-5p and ath-miR5026. The three miRNA genes form a cluster on *Arabidopsis* chromosome 4 (see the first table for detailed genomic positions of the corresponding pre-miRNAs). For the mature miRNAs in the second table, their expression levels in flowers, leaves, roots and seedlings, based on the sRNA HTS data, were highlighted in different background colors. The expression levels of the pre-miRNAs/pri-miRNAs detected by real-time quantitative PCR (*PP2A* as the reference gene) in different organs were highlighted in similar background colors as the second table. For (**A**) to (**D**), the sRNA HTS data sets were retrieved from GEO (Gene Expression Omnibus) [68]: WT_Flower (indicated by yellow background), GSM707678; WT_Leaf (green), GSM707679; WT_Root (gray), GSM707680; WT_Seedling (red), GSM707681. For (**B**) and (**C**), the MPSS data sets (“17bp_summary.txt.gz” and “20bp_summary.txt.gz”) were retrieved from *Arabidopsis* MPSS Plus Database.^18^ In both data sets, the libraries INF, INS, AP1, AP3, AGM and SAP were prepared from flowers (yellow). S04, S52, LES and LEF were prepared from leaves (green). ROS and ROF were prepared from roots (gray), and GSE from young seedlings (red). The pre-miRNA/pri-miRNA expression data were retrieved from mirEX.^17^ Note: for all the panels, the *y* axes are in log scale.

Although there are numerous dynamic factors influencing the *in vivo* levels of the functional miRNAs [13], we imagined that the expression levels of the miRNA gene products should be one of the major components determining the miRNA activities. In another word, the identified list of the organ-specific miRNAs should be partially supported by the expression patterns of the corresponding pre-miRNAs and the pri-miRNAs. For this purpose, the recently published database mirEX (http://comgen.pl/mirex/) [17] is quite useful. Thus, we made a comparison between the expression levels of the *Arabidopsis* pre-miRNAs/pre-miRNAs (obtained from mirEX) and those of the mature miRNAs (obtained from HTS data sets mentioned above; see Table S1). Specifically, the expression levels of the pre-miRNAs/pri-miRNAs detected in “10-day seedlings” and “14-day seedlings” were compared with those of the mature miRNAs detected in the WT seedling library (i.e. GSM707681). The expression levels of the pre-miRNAs/pri-miRNAs in “42-day rosette leaves” and “53-day rosette leaves” were compared with those of the mature miRNAs in the WT leaf library (i.e. GSM707679). The pre-miRNA/pri-miRNA expression levels in “53-day inflorescences” were compared with those of the mature miRNAs in the WT flower library (GSM707678). As a result, similar expression patterns between the miRNAs and their precursors were found for a set of miRNAs including miR169b, miR172c/d, miR391, miR771, miR780.1/.2, miR837-3p/−5p, miR845a, miR851-5p, miR825, miR841, miR857, and miR2111b* (Figure 3A and Figure S2).

Another valuable resource is the mRNA MPSS (massively parallel signature sequencing) data. Considering the fact that the MPSS tag of a given transcript is theoretically located at the Sau3A recognition site nearest to the 5′ end of the polyadenylation tail (see detailed instruction in *Arabidopsis* MPSS Plus Database (http://mpss.udel.edu/at/mpss_index.php) [18]), and that most miRNA genes are transcribed by RNA Pol II [4]–[6], the MPSS short reads could be used for mapping the potential poly(A) sites of the miRNA genes. To this end, all the MPSS sequences were mapped to the 10-kb (kilobase) sequences downstream of the pre-miRNAs of all the organ-specific miRNAs. The sites supported by two short reads of different lengths (i.e. 17 nt and 20 nt from the two data sets, “17bp_summary.txt.gz” and “20bp_summary.txt.gz”, respectively) were considered to be the poly(A) site candidates (Table S7). For both the 17-nt and the 20-nt MPSS tags near the potential poly(A) sites of the miRNA genes, 13 libraries (“INF”, “INS”, “AP1”, “AP3”, “AGM” and “SAP” prepared from floral organ; “S04”, “S52”, “LES” and “LEF” prepared from leaves; “ROS” and “ROF” prepared from roots; “GSE” prepared from seedlings. See detailed information in **Materials and Methods**) were utilized to unravel their expression patterns. The expression patterns of the poly(A) site signatures were then treated as the expression profiles of the pri-miRNAs, which were compared with those of the mature miRNAs and the miRNA precursors obtained above. As a result, nine miRNA genes (*miR156a*, *miR163*, *miR168a*, *miR172b*, *miR399b*/*d*, *miR447b*, *miR856* and *miR2111a*) possess consistent or at least partially overlapping expression patterns between the miRNA(*)s and the corresponding precursors. For instances, the transcription products of *miR156a*, *miR399b*/*d* and *miR2111a* were specifically expressed in the young seedlings. *miR163* and *miR168a* were highly expressed in the leaf organ, and *miR447b* and *miR856* were predominantly expressed in the floral organ (Figure 3B and Figure S3). It indicates that the organ-specific accumulation of the mature miRNAs could be partially reflected by the primary and the secondary transcription levels of the miRNA genes. However, it is not always the case. The expression patterns of certain pri-miRNAs were quite consistent with those of the pre-miRNAs/pri-miRNAs obtained from mirEX, but different from the mature miRNAs (Figure S4). For example, both the poly(A) tag-based and the mirEX-derived expression data indicated that the miRNA precursors of *miR775* were highly accumulated in the flowers of *Arabidopsis*, while no specific expression pattern was observed for the mature miRNA in the WT plant (Figure 3C).

The genomic structures and the sequence characteristics of miRNA clusters have been previously studied in several plants [19]–[22]. Pieces of evidences indicate that miRNA genes resided within a cluster share similar transcriptional features which may attribute to the common upstream promoter. However, few systematic surveys have been carried out to experimentally confirm this notion. Here, the expression patterns of the miRNA clusters were investigated in the four organs of *Arabidopsis*. For one miRNA cluster, it must contain two or more miRNA genes, and the distance between the two neighboring pre-miRNAs should be less than 10 kb. As a result, 24 clusters were identified (Table S8). Based on the mirEX-derived expression data, quite consistent expression patterns are shared by the pre-miRNAs/pri-miRNAs belonging to a cluster, such as the cluster *miR395a–miR395b–miR395c*, *miR398b–miR398c*, *miR447a–miR447b–miR447c*, *miR162a–miR834*, *miR771–miR851*, and *miR850–miR863–miR5026* (Figure 3D and Figure S5). For these clusters, the expression patterns of the mature miRNAs always overlap with those of the miRNA precursors. However, some exceptions exist. For example, within the *miR162a–miR834* cluster, the expression profiles of the two miRNA precursors are quite similar with each other, whereas the *in vivo* levels of the mature miR834 could not be detected in the four organs of WT plant (Figure S5). It indicates that the processing efficiency of the two miRNA precursors varied greatly. One cluster, *miR395a–miR395b–miR395c*, shows an unusual strand distribution pattern. *miR395b* and *miR395c* located on the “+” strand while *miR395a* was on the opposite strand. Interestingly, the expression patterns of the miRNA precursors and the mature miRNAs of the three genes are quite consistent. It indicates that the three miRNA genes might share a common promoter possessing bidirectional regulatory role.

### Degradome Sequencing Data-based Identification of miRNA–target Pairs

To further unravel the biological relevance of the organ-specific miRNAs, we set out to identify their target genes. First, all the miRNAs were subjected to transcriptome-wide target prediction by using miRU algorithm (see **Materials and Methods**) [23], [24]. Then, the predicted miRNA–target interactions were validated by degradome sequencing data. As a result, a total of 745 miRNA–target pairs were identified (Table S9), and most of them were supported by evident cleavage signals resided within the 10^th^ to 11^th^ nucleotide regions of the miRNAs (Figure S6), which were the canonical sites for plant miRNA action [3], [25]–[27].

**Figure 4:**
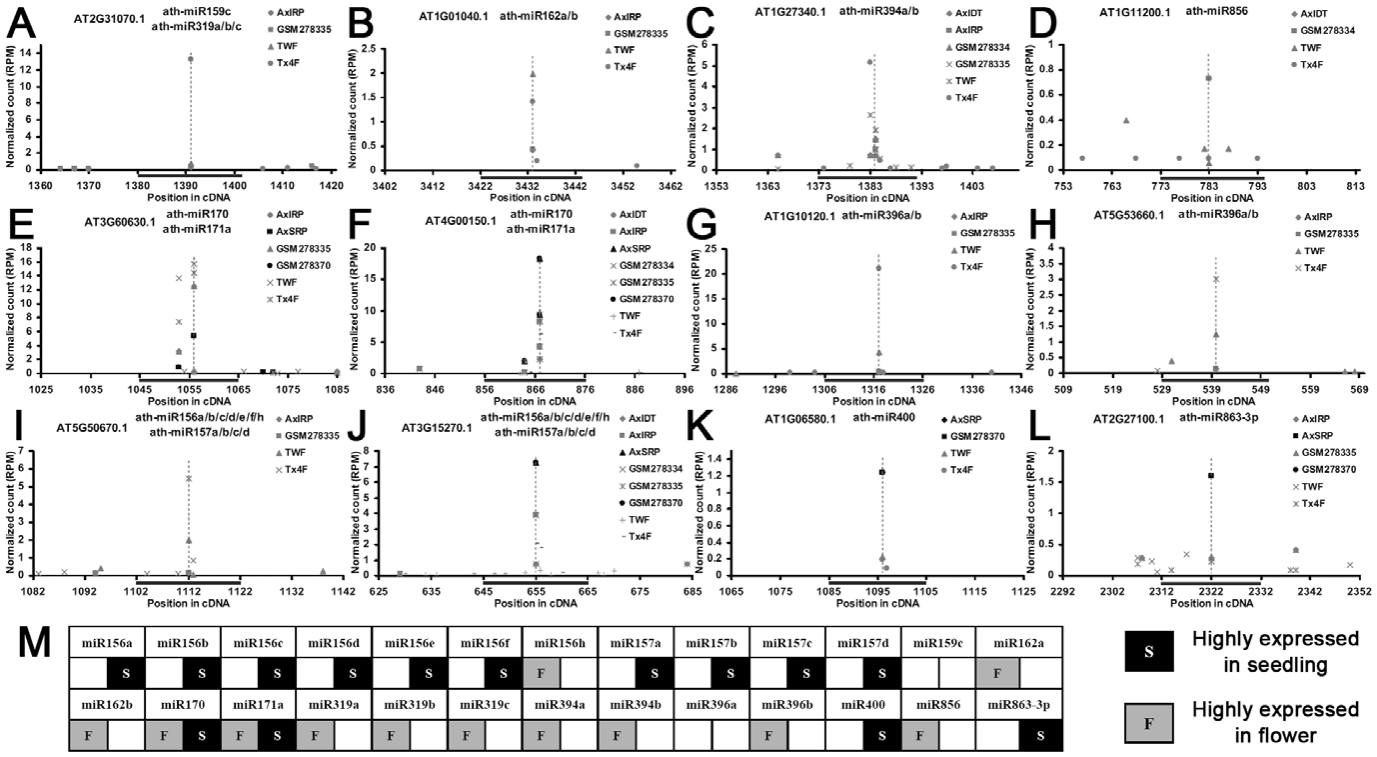
Organ-specific validation of the microRNA targets based on degradome sequencing data. For (**A**) to (**L**), the target transcripts and the microRNA regulators are listed on the top of each target plot (t-plot). The *x* axes measure the positions of the target binding regions (indicated by gray bars) on the target transcripts. The *y* axes measure the intensity (in RPM, reads per million) of the cleavage signals based on the degradome data. The degradome signals belonging to GSM278370 and AxSRP which were prepared from the *Arabidopsis* seedlings were represented by black dots, and those from the libraries prepared from the flowers were represented by gray ones (see detailed library information in **Materials and Methods**). The prominent cleavage sites are denoted by dashed lines. (**M**) Expression levels of the mature microRNAs listed in (**A**) to (**L**) in the seedlings and the flowers. Based on the small RNA high-throughput sequencing data, the microRNAs specifically expressed in the seedlings or the flowers (either in the WT-related library group, or in the AGO1-related library group, or in the AGO4-related library group; see detailed information in **Materials and Methods**) were indicated by black “S” or gray “F” respectively.

We queried whether these miRNAs could play an organ-specific regulatory role on their targets. To this end, the degradome libraries were divided into two groups, the ones prepared from seedlings (GSM278370 and AxSRP) and those prepared from flowers (GSM278333, GSM278334, GSM278335, AxIDT, AxIRP, Col, ein5l, TWF, and Tx4F; see details in **Materials and Methods**). Based on this classification, dozens of miRNAs were found to exert organ-specific regulation on the targets, which was coincident with the miRNAs’ expression patterns. For example, the targets of the flower-specific miRNAs, miR162a/b, miR394a/b and miR856, were specifically cleaved in the floral organ (Figure 4B, C, D). For the seedling-specific miRNAs, such as miR400 and miR863-3p, their targets were intensively sliced in the young seedlings (Figure 4K, L). For the miRNAs highly expressed in both the flowers and the seedlings, such as miR170 and miR171a, prominent cleavage signals of the corresponding targets could be detected in both organs (Figure 4E, F).

This analysis integrating the expression of the miRNAs with the degradome-based target cleavage patterns also enabled us to discriminate the actual effects of the homologous miRNAs on the common predicted targets. For instances, both miR159c and miR319a-c were indicated to target AT2G31070.1 (a *TCP* family gene) based on miRU prediction and the degradome-based cleavage signals (Figure 4A). However, when considering the fact that only miR319a-c were highly expressed in the flowers, and that the cleavage signals were flower-specific (Figure 4M), we proposed that miR319a-c but not miR159c were the effective regulators of AT2G31070.1. This hypothesis is supported by a previous report on the functional specialization of *Arabidopsis* miR159 and miR319 [28]. This Both AT1G10120.1 and AT5G53660.1 were predicted to be targeted by miR396a and miR396b belonging to the same family. However, the cleavage signals were flower-specific, and only miR396b was highly expressed in the floral organ (Figure 4G, H, M). Thus, miR396b might be the stronger candidate to exert the repressive regulation on the two targets. Besides, miR156a-f/h and miR157a-d were the potential regulators of AT5G50670.1 whose cleavage remnants were exclusively identified in the flowers (Figure 4I). However, only miR156h was flower-specific (Figure 4M), indicating its dominant repressive role on AT5G50670.1 in the floral organ. Different from the above case, the cleavage remnants of AT3G15270.1 were found in both the flowers and the seedlings (Figure 4J), suggesting that flower-specifically expressed miR156h, and seedling-specific miR156a-f and miR157a-d might regulate AT3G15270.1 in different organs.

**Figure 5:**
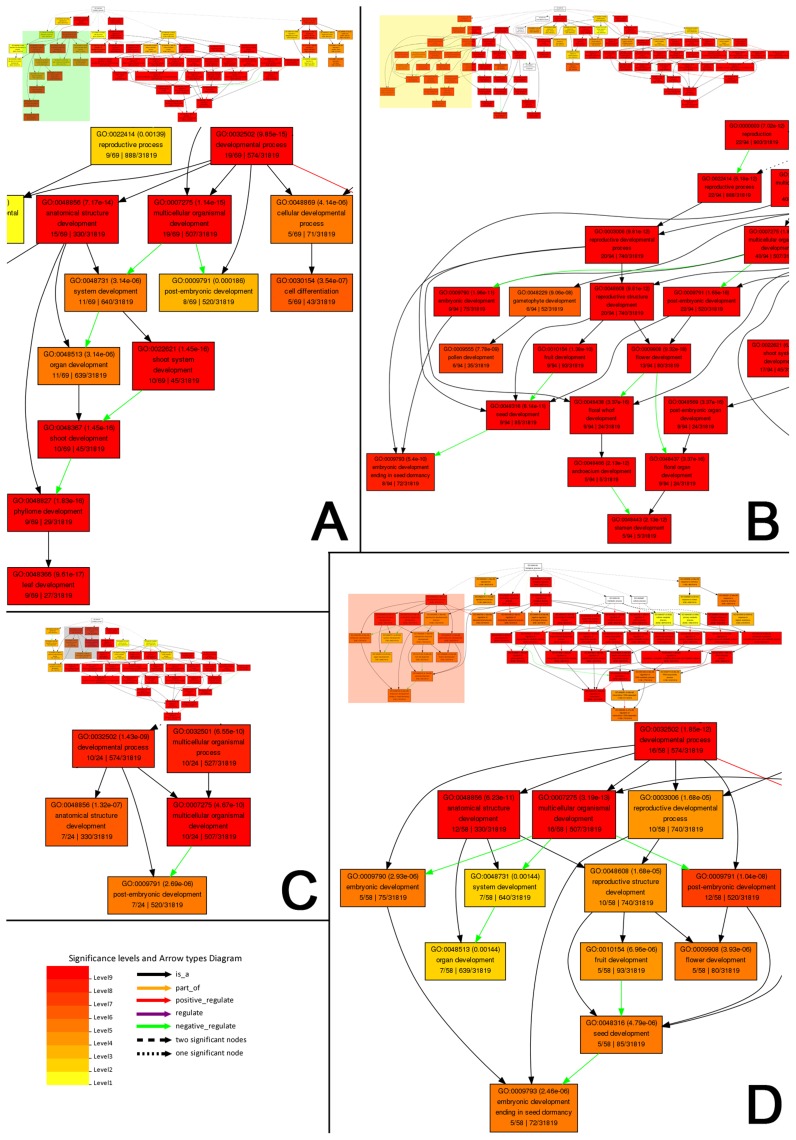
GO (Gene Ontology) term enrichment analysis of the validated targets of the organ-specific microRNAs. Based on the small RNA high-throughput sequencing data, the targets of the organ-specific microRNAs in either of the three library groups (i.e. the WT-related library group, the AGO1-related group, or the AGO4-related group; see details in **Materials and Methods**) were included for this analysis. (**A**) Analysis of the targets of the leaf-specific microRNAs within the “Biological Process” category. (**B**) Analysis of the targets of the flower-specific microRNAs within the “Biological Process” category. (**C**) Analysis of the targets of the root-specific microRNAs within the “Biological Process” category. (**D**) Analysis of the targets of the seedling-specific microRNAs within the “Biological Process” category. This analysis was performed by using agriGO,^28^ selecting the “*Arabidopsis* genome locus (TAIR)” as a control set. For (**A**) to (**D**), the figure keys are shown at the bottom left.

To gain further functional insights into the miRNA–target interactions, all the targets were subjected to functional enrichment analysis by using AgriGO [29] [using “Singular Enrichment Analysis”; species: *Arabidopsis thaliana*; reference: *Arabidopsis* gene model (TAIR, The *Arabidopsis* Information Resource)]. First, based on the expression patterns of the miRNAs, the targets were divided into four groups: the targets regulated by the flower-specific, the leaf-specific, the root-specific, and the seedling-specific miRNAs. Since some miRNAs were highly accumulated in more than one organ, certain targets were assigned to two or more groups, pointing to the potential biological roles of these genes in various organs. The GO (Gene Ontology) term-based functional analysis shows that the target genes possess organ-specific biological functions, which are coincident with the expression patterns of the corresponding miRNA regulators. For instances, the functions of the genes targeted by the leaf-specific miRNAs were significantly enriched in leaf development (Figure 5A), the genes regulated by the flower-specific miRNAs possessed enriched functions involved in floral organ development (Figure 5B), and the functions of the genes targeted by the seedling-specific miRNAs were enriched in embryonic and post-embryonic development (Figure 5D). For the genes regulated by the root-specific miRNAs, the GO term “root development” was not found in the result of this enrichment analysis. However, the enriched GO terms “multicellular organismal development”, “anatomical structure development” and “post-embryonic development” were potentially related to “root development” (Figure 5C). Taken together, the functional analysis of the targets demonstrated that a high correlation existed between the miRNA expression patterns and the organ-specific functions of the target genes. Considering the notion that most plant miRNAs were incorporated into AGO1-associated miRISCs to guide target cleavages [1]–[3], an independent functional enrichment analysis was performed for the targets of the organ-specific miRNAs identified from the AGO1-related libraries (GSM707682, GSM707683, GSM707684 and GSM707685; see details in **Materials and Methods**). Again, a well correlation between the miRNA expression patterns and the functions of their target genes was observed (Figure S7).

### Network Construction and Subnetwork Analysis

**Figure 6:**
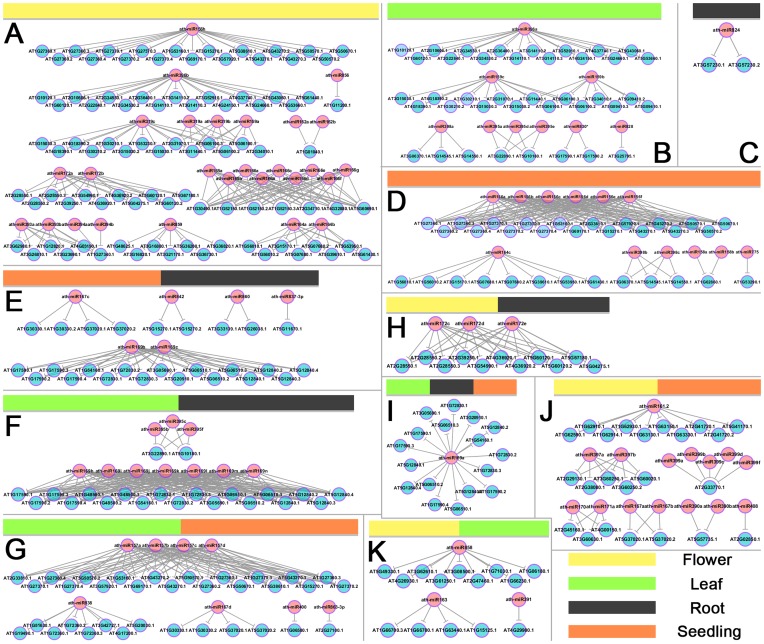
Organ-specific microRNA-mediated regulatory networks. (**A**) Network mediated by flower-specific microRNAs. (**B**) Network mediated by leaf-specific microRNAs. (**C**) Network mediated by root-specific microRNAs. (**D**) Network mediated by seedling-specific microRNAs. (**E**) Network mediated by the microRNAs specifically expressed in roots and seedlings. (**F**) Network mediated by the microRNAs specifically expressed in leaves and roots. (**G**) Network mediated by the microRNAs specifically expressed in leaves and seedlings. (**H**) Network mediated by the microRNAs specifically expressed in flowers and roots. (**I**) Network mediated by the microRNAs specifically expressed in leaves, roots, and seedlings. (**J**) Network mediated by the microRNAs specifically expressed in flowers and seedlings. (**K**) Network mediated by the microRNAs specifically expressed in flowers and leaves. All the networks were constructed based on the validated microRNA target list by using Cytoscape.^29^ The different color bar combinations indicate the specific expression patterns of the microRNAs involved in each network. See the bottom right for the meanings of the color bars.

Based on the obtained miRNA–target pairs, the miRNA-mediated regulatory networks were constructed by using Cytoscape [30]. According to the miRNA expression patterns, the networks were classified into eleven categories (Figure 6).

Several subnetworks well support the known miRNA-mediated regulation. For example, distinct regulatory cascades involving miR159 and miR319 were included in the subnetworks mediated by the leaf-specific miRNAs and those mediated by the flower-specific miRNAs respectively (Figure 6A, B). It was consistent with the previous report that the sequence and expression differences led to functional specialization between miR159 and miR319 in *Arabidopsis* [28]. The flower-related subnetworks mediated by miR156, miR159, miR165/miR166, miR171 and miR172 (Figure 6A, J) correlate well with the reported biological roles of these miRNAs in floral organ development [26], [31]–[39]. Within the leaf-specific subnetworks (Figure 6B), the miR159- and miR396-mediated regulatory cascades are supported by several previous reports [40]–[42]. Within the seedling-related subnetworks (Figure 6D, G), the miR156- and miR157-involved cascades well reflect their reported functions in embryogenesis [43] and maintaining normal seedling development [44]. Notably, different sets of the target genes point to the functional specialization between the two miRNA families within the seedling-related subnetworks. Based on the result that miR156a-f were specifically expressed in seedlings, and miR156h and miR172a-e were highly expressed in flowers (Table 1), the collaboration between miR156 and miR172 for controlling vegetative-to-reproductive phase transition in *Arabidopsis* was imaginable. This hypothesis is supported by a previous study [45]. Moreover, the distinct expression patterns between miR156a-f and miR156h (Table 1) point to the possibility that different members of miR156 family may function in distinct organs or at specific developmental stages. Considering the fact that certain miRNAs were highly accumulated in two or more organs, their regulatory cascades might be involved in multi-organ development (Figure 6E to K).

Several novel cascades were uncovered. *F-box* genes were reported to be highly expressed in the sperm cells of *Arabidopsis* [46], and Kim *et al.*’s study (2008) uncovered the role of an F-box protein (FBL17) in germline proliferation [47]. Consistent with a recent study [48], miR859 was abundant in the floral organ in both the WT- and the AGO1-related library groups (Table S1). Based on the degradome data, miR859 targets seven *F-box* genes (Figure 6A), strongly indicating a potential role of this miRNA in reproduction. Based on the TAIR annotation, *AT2G02850* targeted by miR408 encodes plantacyanin (one of blue copper proteins) and is involved in anther development and pollination. It well correlates with the abundant levels of miR408 in flower-prepared libraries belonging to WT- and AGO1-related groups (Table S1). Additionally, *AT2G02850* was experimentally confirmed to be cleaved by miR408 in *Arabidopsis* [49], and miR408 was suggested to mediate copper signaling [49]–[51]. Also based on the previous reports [49]–[51], *miR397* was indicated to be copper-responsive. Supportively, the target genes of miR397, i.e. *AT5G60020*, *AT2G38080* and *AT2G29130* (all encode laccase-like multicopper oxidases), are copper-related [52]. *miR391* was highly expressed in the flowers and the leaves (Table S1). According to the TAIR annotation, one target of miR391, *AT4G29900* (Figure 6K), is involved in shoot development and inflorescence morphogenesis. Thus, it fits well with the expression pattern of *miR391*. Consistent with our recent report [53], the regulatory activities of certain miRNA*s were observed. *AT3G17590* was targeted by miR830* (Figure 6B), and it plays a potential role in chromatin modifications [54]. A recent result showed that miR837-3p was involved in phosphate signal transduction in *Solanum lycopersicum* [55]. Based on the TAIR annotation, one target of miR837-3p, *AT5G11670* (Figure 6E), encodes a cytosolic enzyme implicated in malate metabolism and possibly involved in the oxidative pentose phosphate pathway. It suggests that miR837-3p-mediated phosphate signaling might be conserved in *Arabidopsis*.

**Figure 7:**
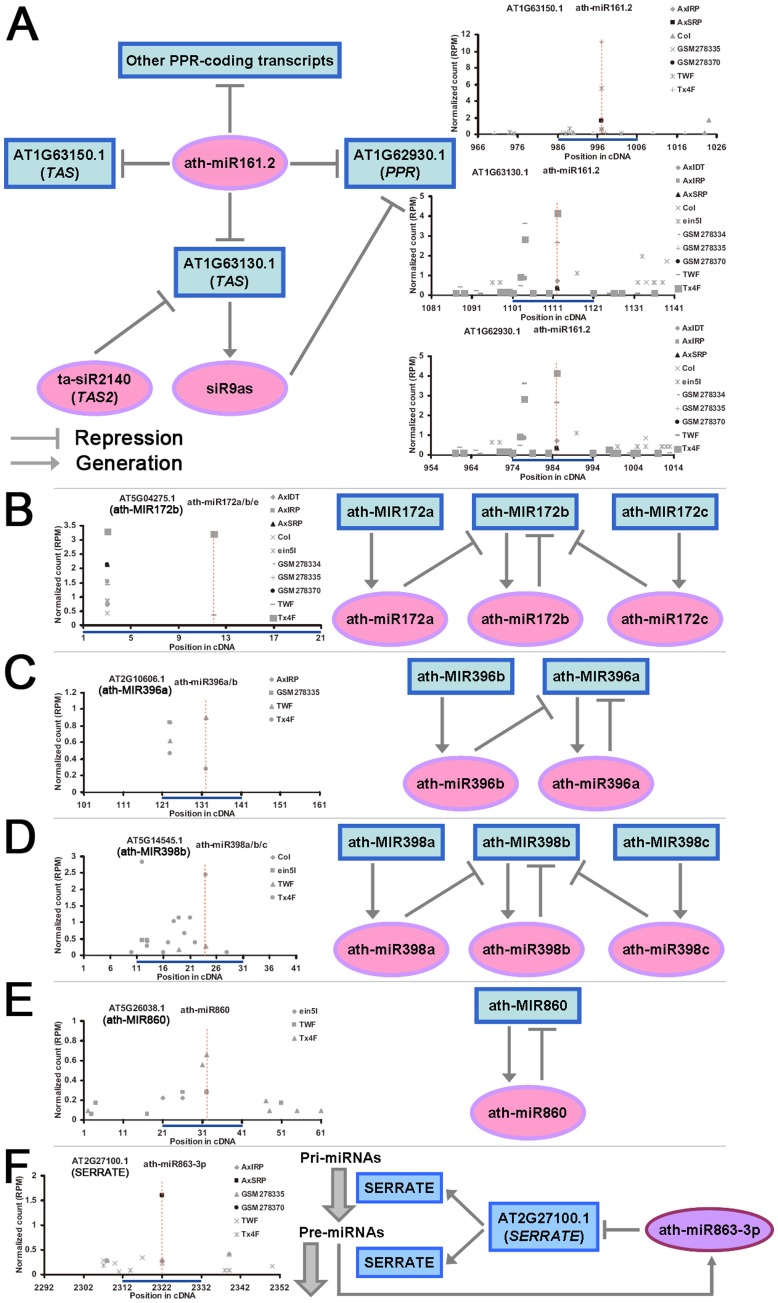
Certain intriguing subnetworks mediated by organ-specific microRNAs. (**A**) ath-miR161.2-mediated subnetwork involving *PPR* (*pentatricopeptide repeat*) genes and *trans*-acting small interfering RNA (ta-siRNA)-generating (*TAS*) genes. (**B**) miR172-involved self-regulatory network. (**C**) miR396-involved self-regulatory network. (**D**) miR398-involved self-regulatory network. (**E**) miR860-involved self-regulatory network. (**F**) ath-miR863-3p-mediated regulation of *SERRATE*. For (**A**) to (**F**), the degradome-based evidences for the microRNA-mediated target cleavages are shown by the target plots (t-plots). The target transcripts and the microRNAs are listed on the top of each plot. The *x* axes measure the positions of the target binding regions (indicated by blue bars) on the target transcripts. The *y* axes measure the intensity (in RPM, reads per million) of the cleavage signals based on the degradome sequencing data. The degradome signals belonging to GSM278370 and AxSRP which were prepared from the *Arabidopsis* seedlings were represented by black dots, and those from the libraries prepared from the flowers were represented by gray ones (see detailed library information in **Materials and Methods**). The prominent cleavage sites are denoted by red dashed lines.

Based on the TAIR annotations, some targets of miR161.2, such as AT1G63150.1 and AT1G63130.1, were capable of producing ta-siRNAs (*trans*-acting small interfering RNAs). AT1G63130.1 was also targeted by ta-siR2140, and could generate siR9as regulating one *PPR* target of miR161.2, i.e. *AT1G62930*. Thus, the regulatory circuit involving both miR161.2 and ta-siRNAs could ensure physiologically proper expression levels of the *PPR* and the *TAS* genes (Figure 7A). Supported by the previous reports [56], [57], potential self-regulation was observed for several miRNA genes, such as *miR172b*, *miR396a*, *miR398b*, and *miR860* (Figure 7B to E). For these miRNA genes, the miRNA precursors (pri-miRNAs and/or pre-miRNAs) were cleaved by their own mature miRNAs. Another intriguing subnetwork involves miR863-3p (Figure 7F). The *SERRATE* gene (*AT2G27100*) encoding a zinc finger protein was identified to be targeted by miR863-3p, and the cleavage signals were significantly high in the degradome libraries prepared from the young seedling (left panel of Figure 7F), which was consistent with the leaf-/seedling-specific expression pattern of miR863-3p (Table 1 and Table S1). Notably, SERRATE is essential for the processing of the miRNA precursors into the miRNA/miRNA* duplexes in *Arabidopsis* [7]–[10], [58]–[61]. This subnetwork indicates that a novel regulatory layer involving miR863-3p might exist for modulating the processing efficiency of the miRNA gene products, which was worthwhile for further investigation.

## Discussion

### Sequence Features and Functional Implications of the AGO4-associated miRNAs

In this study, the expression patterns of the *Arabidopsis* miRNA genes, including the accumulation levels of the pri-miRNAs, the pre-miRNAs and the mature miRNAs, were investigated in various organs. First, by utilizing three sets of sRNA HTS data, the organ-specific miRNAs were identified from the WT-, the AGO1-, and the AGO4-related library groups, separately (Table 1). Particularly, the organ-specific miRNAs extracted from the AGO4-related libraries were highly enriched in the floral organ (Figure 1A). Although pieces of evidences, such as the association of miR172 with AGO4 [31], [32], [62], indicate the potential role of AGO4 in floral organ development, further investigations are needed to uncover the biological relevance of the association of flower-specific miRNAs with AGO4 in *Arabidopsis*. Previous studies showed that AGO4 preferentially recruited 24-nt, 5′ A-started sRNAs to exert its role in transcriptional gene silencing at chromatin level [15], [63]–[65]. However, the biological relevance of AGO4–miRNA association and the AGO4-mediated target cleavages are still elusive [66]. In this study, the sequence characteristics of the miRNAs highly enriched in AGO4 were analyzed (Figure 2). As a result, a relatively conserved “C” was identified on the 3^rd^ nucleotide at the 3′ ends of these miRNAs. Moreover, compared to the control set, the AGO4-enriched miRNAs start with 5′ U more preferentially, which is quite different from the previous report on the AGO4-associated sRNAs [15]. Whether these two sequence features could determine the preferential association of a specific miRNA with AGO4, and whether the AGO4 complexes guided by these miRNAs could perform target cleavages need to be addressed.

### Gain Further Insights from the Expression Pattern-based Analysis

Based on the expression data of the pre-miRNAs and the pri-miRNAs derived from mirEX and MPSS data sets, an expression pattern-based comparison was performed among the miRNAs, the miRNA precursors. Several miRNA genes with consistent expression patterns between the mature miRNAs and the precursors were identified (Figure 3A to C, and Figure S2 to S4). However, it is not the case for all the miRNA genes. In this regard, although the expression of the primary and the secondary transcripts could partially reflect the *in vivo* abundances of the corresponding miRNA(*)s, the actual regulatory activities of the miRNA(*)s could be affected by other factors such as the organ-specific processing efficiency. This hypothesis is well supported by the recent notion that the plant miRNA activities should be calculated in a more dynamic way [13].

We also investigated the expression patterns of the miRNA clusters throughout the *Arabidopsis* genome (Table S8). Out of the 24 clusters, six are quite interesting that a similar expression pattern is shared by all the miRNA precursors belonging to a specific cluster. For some of the six clusters, the expression patterns of the mature miRNAs within a cluster are also quite consistent with the precursors. These observations suggest that the coordinate expression status of the miRNAs belonging to a cluster might result from a common upstream regulatory region essential for controlling their organ-specific expression. However, for most of the remaining miRNA clusters, distinct expression patterns were observed for the miRNA precursors belonging to the same cluster. Excluding the factors such as the degradation of the miRNA precursors and the bias introduced during biological sample preparation, these miRNA genes may not share a common promoter as expected. In another word, the 10-kb rule, which was widely adopted for miRNA cluster definition, needs revision.

### miRNA-mediated Organ-specific Regulation

In many cases, two or more miRNAs belonging to the same family were predicted to share common binding sites on specific target transcripts. *In silico* target prediction followed by canonical degradome-based validation did not allow us to uncover the exact contribution of a specific miRNA to the cleavages of its predicted target(s). Fortunately, we found that in some cases, different members of the same miRNA family exhibited distinct expression patterns, indicating a functional diversification among the family members. Based on this observation, we raised the scenario that if a specific miRNA–target interaction was true, the cleavage signals should be predominantly, at least in some cases, detected in the organ(s) that the miRNA was highly expressed. This approach by combining the miRNA expression patterns with the distributions of the degradome signatures in different organs allowed us to extract the strong candidate for a specific cleavage action from the miRNAs sharing high sequence identities (Figure 4).

### Biological Hints Inferred from the Organ-specific miRNA-mediated Networks

Based on the identified miRNA–target pairs, comprehensive regulatory networks were constructed (Figure 6). The biological meanings of these networks were partially uncovered by subnetwork analysis. Numerous subnetworks are supported by the previous studies, indicating a high reliability of these networks. Besides, some novel regulatory cascades were discovered (Figure 7), such as miR161.2-, ta-siRNA- and *PPR* gene-involved subnetworks, and the self-regulation of *miR172b*, *miR396a*, *miR398b*, *miR860*. Another novel finding was the regulation of *SERRATE* by miR863-3p, which has never been reported. As introduced in the above sections, SERRATE is indispensable for accurate and efficient processing of the miRNA precursors. The miR863-3p-medieted post-transcriptional modulation of the expression of *SERRATE* could make a great contribution to the homeostasis of miRNA production. In this regard, this regulatory pathway should be highly conserved in plants. But, strikingly, based on the miRBase registries (the latest version, release 18), miR863 was only identified in *Arabidopsis*. Thus, this novel regulation needs further interrogation.

## Materials and Methods

### Data Sources

All the sequence information of the *Arabidopsis* miRNAs was retrieved from miRBase (http://www.mirbase.org/; release 17) [67].

The sRNA HTS data used in this study is a gift from a recent study [14], and can be downloaded from GEO (Gene Expression Omnibus; http://www.ncbi.nlm.nih.gov/geo/) [68]. The WT-related library group includes: GSM707678 (WT_Flower: prepared from the flowers of six-week-old wild type plants), GSM707679 (WT_Leaf: from the leaves of four-week-old wild type plants), GSM707680 (WT_Root: from the roots of four-week-old wild type plants), and GSM707681 (WT_Seedling: from wild type seedlings). The AGO1-related library group contains: GSM707682 (AGO1_Flower: AGO1-associated sRNAs from the flowers of six-week-old wild type plants), GSM707683 (AGO1_Leaf: AGO1-associated sRNAs from the leaves of four-week-old wild type plants), GSM707684 (AGO1_Root: AGO1-associated sRNAs from the roots of four-week-old wild type plants), and GSM707685 (AGO1_Seedling: AGO1-associated sRNAs from wild type seedlings). The AGO4-related library group includes: GSM707686 (AGO4_Flower: AGO4-associated sRNAs from the flowers of six-week-old wild type plants), GSM707687 (AGO4_Leaf: AGO4-associated sRNAs from the leaves of four-week-old wild type plants), GSM707688 (AGO4_Root: AGO4-associated sRNAs from the roots of four-week-old wild type plants), and GSM707689 (AGO4_Seedling: AGO4-associated sRNAs from wild type seedlings).

The mRNA MPSS data (data set names: “17bp_summary.txt.gz” and “20bp_summary.txt.gz”) used for detecting potential poly(A) sites of the miRNA genes were retrieved from *Arabidopsis* MPSS Plus Database (http://mpss.udel.edu/at/mpss_index.php) [18]. Both data sets contain 17 libraries, and the expression data from the following 13 libraries were used: INF (inflorescence: mixed stage, immature buds), INS (inflorescence: mixed stage, immature buds), AP1 (*ap1–10* inflorescence: mixed stage, immature buds), AP3 (*ap3–6* inflorescence: mixed stage, immature buds), AGM (agamous inflorescence: mixed stage, immature buds), SAP (*sup*/*ap1* inflorescence: mixed stage, immature buds), S04 (leaves: 4 hr after salicylic acid treatment), S52 (leaves: 52 hr after salicylic acid treatment), LES (leaves: 21 day, untreated), LEF (leaves: 21 day, untreated), ROS (root: 21 day, untreated), ROF (root: 21 day, untreated), and GSE (germinating seedlings).

The *Arabidopsis* genome information was retrieved from the FTP site of TAIR (release 9) [69].

The expression data of the *Arabidopsis* pre-miRNAs/pri-miRNAs were retrieved from mirEX (http://comgen.pl/mirex/) [17].

The *Arabidopsis* degradome sequencing data sets GSM278333 (prepared from wild type inflorescences), GSM278334 (wild type inflorescences), GSM278335 (wild type inflorescences), and GSM278370 (wild type seedlings) were retrieved from GEO, and AxIDT (prepared from wild type inflorescences), AxIRP (wild type inflorescences), AxSRP (wild type seedlings), Col (wild type inflorescences), ein5l (inflorescences of the *ein5–6* mutant), TWF (wild type inflorescences), and Tx4F (inflorescences of the *xrn4* mutant) were retrieved from *Arabidopsis* PARE Database (http://mpss.udel.edu/at_pare/) [18].

### Identification of Organ-specific miRNAs

In order to allow cross-library comparison, each sRNA HTS data set was normalized. The normalized read count (in RPM; reads per million) of a short sequence from a specific library was calculated by dividing the raw read count of this sequence by the total read counts of the library, and then multiplied by 10^6^. A miRNA was considered to be organ-specifically expressed if: its expression level in a specific organ is three or more times higher than at least two of the other three organs. Besides, its expression level in the specific organ should be higher than 10 RPM. In this regard, one miRNA might be identified to be highly expressed in two organs of *Arabidopsis*. The organ-specific miRNAs were identified from the WT-, AGO1- and AGO4-related library group separately.

### MPSS Data-based Identification of Potential Poly(A) Sites of the Organ-specific miRNA Genes

As introduced in *Arabidopsis* MPSS Plus Database (http://mpss.udel.edu/at/mpss_index.php),^18^ “the position of the MPSS tag for a given gene or transcript should coincide with the first Sau3A site 5′ of the polyadenylation site.” Thus, the mRNA MPSS data sets (“17bp_summary.txt.gz” and “20bp_summary.txt.gz”) were used for detecting potential poly(A) sites of the miRNA genes. First, the 10-kb sequences downstream of the pre-miRNAs of all the organ-specific miRNAs were retrieved from TAIR (release 9) [69] according to the genomic positions of the pre-miRNAs provided by miRBase (release 17) [67]. Then, we mapped all the MPSS short sequences to the 10-kb sequences by using BLAST algorithm [70], and only the perfectly matched sequences were retained. For a potential poly(A) site, there must be two MPSS sequences of different lengths (i.e. 17 nt and 20 nt). The expression data of these poly(A) signal-related MPSS short sequences was able to reflect the expression patterns of the pri-miRNAs. Then, we made a comparison among the MPSS data-based expression patterns of the pri-miRNAs, mirEX-derived expression data of the pre-miRNAs/pri-miRNAs, and the miRNA expression data from the WT-related library group.

### Target Prediction and Degradome Sequencing Data-based Validation

Target prediction was performed by using miRU algorithm^23, 24^ with default parameters (i.e. Total penalty score allowed = 3; G:U pairs allowed = 6; Indels (insertions and deletions) allowed = 1; Other mismatches allowed = 3). Eleven degradome sequencing data sets (see **Data sources**) were utilized to validate the predicted miRNA–target pairs. First, read count normalization was performed for all the degradome sequencing data sets as described above. Then, two-step filtering was performed to extract the reliable miRNA–target pairs. During the first step, the predicted miRNA binding sites along with the 50-nt surrounding sequences at both ends were subjected to BLAST^70^ with the purpose to save the calculation time. For the BLAST, all the degradome data sets were used at the same time to do a comprehensive search. It was based on the scenario that a miRNA–target pair could be selected as the candidate once the cleavage signal(s) was/were found in any data set(s). The predicted targets were retained for further filtering if they meet the following criterion: there must be perfectly matched degradome signatures with their 5′ ends resided within 8–14 nt regions away from the 5′ ends of the target binding sites. These transcripts were subjected to a second BLAST, and the degradome signatures along each transcript were obtained to provide a global signal/noise view. Referring to our previous study,^71^ both the global and the local t-plots (target plots)^57, 72^ were drawn. Finally, exhaustive manual filtering was performed, and only the transcripts with cleavage signals easy to be recognized when compared to the noises within and surrounding the target binding regions were extracted as the validated miRNA–target pairs. The GO term enrichment analysis was performed by using agriGO (http://bioinfo.cau.edu.cn/agriGO/analysis.php).^28.^

## Supporting Information

Figure S1 **Workflow of the main analyses in this study.**
(PDF)

Figure S2 **List of the microRNA genes with consistent expression patterns between the mature miRNAs (based on small RNA high-throughput sequencing data) and the miRNA precursors (based on the data provided by mirEX).** For the mature miRNAs listed in the tables, their detectable expression levels (normalized in RPM; reads per million) in flowers, leaves, roots and seedlings were highlighted in gray background. The small RNA high-throughput sequencing data sets were retrieved from GEO (Gene Expression Omnibus; http://www.ncbi.nlm.nih.gov/geo/) [68]: WT_Flower, GSM707678; WT_Leaf, GSM707679; WT_Root, GSM707680; WT_Seedling, GSM707681. Accordingly, the mirEX-derived expression levels of the pre-miRNA/pri-miRNA detected by real-time quantitative PCR [*PP2A* (*phosphatase 2A*; *AT1G13320*) as the reference gene] in the similar organs were highlighted in pink background. Please note: the *y* axis is in log scale.
(PDF)

Figure S3 **List of the microRNA genes with consistent expression patterns between the mature miRNAs (based on small RNA high-throughput sequencing data) and their precursors (based on the data provided by mirEX and the MPSS data).** For the mature miRNAs listed in the first tables, their detectable expression levels (normalized in RPM; reads per million) in flowers, leaves, roots and seedlings were highlighted in different background. The HTS data sets were retrieved from GEO (Gene Expression Omnibus; http://www.ncbi.nlm.nih.gov/geo/) [68]: WT_Flower, GSM707678; WT_Leaf, GSM707679; WT_Root, GSM707680; WT_Seedling, GSM707681. For the pri-miRNAs listed in the second tables, the detectable levels of the identified poly(A) signals based on MPSS (massively parallel signature sequencing) data were highlighted by the same background colors as above according to the organs analyzed. The MPSS data sets were retrieved from Next-Gen Sequence Databases (http://mpss.udel.edu/at/mpss_index.php) [18]. The libraries INF, INS, AP1, AP3, AGM and SAP were prepared from *Arabidopsis* flowers (indicated by yellow background). S04, S52, LES and LEF were prepared from leaves (green background). ROS and ROF were prepared from roots (gray), and GSE from young seedlings (red). The expression levels of the pre-miRNAs/pri-miRNAs detected by real-time PCR [PP2A (phosphatase 2A; AT1G13320) as the reference gene] in the similar organs retrieved from mirEX (http://comgen.pl/mirex/) [17] were also highlighted in different background as indicated above. Please note: the *y* axis is in log scale.
(PDF)

Figure S4 **List of the microRNA genes with consistent expression patterns between the pre-miRNAs/pri-miRNAs (based on the data provided by mirEX) and the pri-miRNAs (based on the MPSS data).** For the mature miRNAs listed in the first tables, their detectable expression levels (normalized in RPM; reads per million) in flowers, leaves, roots and seedlings, based on the small RNA (sRNA) high-throughput sequencing (HTS) data, were highlighted in different background. The sRNA HTS data sets were retrieved from GEO (Gene Expression Omnibus; http://www.ncbi.nlm.nih.gov/geo/) [68]: WT_Flower, GSM707678; WT_Leaf, GSM707679; WT_Root, GSM707680; WT_Seedling, GSM707681. For the pri-miRNAs listed in the second tables, the detectable levels of the identified poly(A) signals based on MPSS (massively parallel signature sequencing) data were highlighted by the same background colors as above according to the organs analyzed. The MPSS data sets were retrieved from Next-Gen Sequence Databases (http://mpss.udel.edu/at/mpss_index.php) [18]. The libraries INF, INS, AP1, AP3, AGM and SAP were prepared from *Arabidopsis* flowers (indicated by yellow background). S04, S52, LES and LEF were prepared from leaves (green background). ROS and ROF were prepared from roots (gray), and GSE from young seedlings (red). The expression levels of the pre-miRNAs detected by real-time PCR [PP2A (phosphatase 2A; AT1G13320) as the reference gene] in the similar organs retrieved from mirEX (http://comgen.pl/mirex/) [17] were also highlighted in different background as indicated above. Please note: the *y* axis is in log scale.
(PDF)

Figure S5 **Expression of the microRNA clusters.** The genomic positions of the pre-miRNAs (precursor microRNAs) according to miRBase (release 17) [67] were listed in the first tables. For the mature miRNAs listed in the second tables, their detectable expression levels (normalized in RPM; reads per million) in flowers, leaves, roots and seedlings, based on the small RNA (sRNA) high-throughput sequencing (HTS) data, were highlighted in different background. The sRNA HTS data sets were retrieved from GEO (Gene Expression Omnibus; http://www.ncbi.nlm.nih.gov/geo/) [68]: WT_Flower, GSM707678; WT_Leaf, GSM707679; WT_Root, GSM707680; WT_Seedling, GSM707681. The expression levels of the pre-miRNAs detected by real-time PCR [PP2A (phosphatase 2A; AT1G13320) or actin (AT3G18780) as the reference gene] in the similar organs retrieved from mirEX (http://comgen.pl/mirex/) [17] were also highlighted in different background as above. Please note: the *y* axis is in log scale.
(PDF)

Figure S6 **Degradome sequencing data-based identification of the targets regulated by the organ-specific microRNAs in** ***Arabidopsis*** **.** For all the sub-figures, the left panels depict the degradome signals all along the target transcripts, and the right panels provide detailed views of the signals within the regions surrounding the target recognition sites (denoted by blue horizontal lines, and the red dotted lines indicate the most prominent cleavage sites). The transcript IDs are shown on the left panels, and the microRNAs listed on the right. For all the panels, the *x* axes measure the positions of the signals along the transcripts, and the *y* axes measure the degradome signal intensity (in RPM, reads per million). For all the right panels depicting the observed cleavage signals, the signals belonging to the libraries prepared from seedlings (AxSRP and GSM278370) were denoted by black symbols, and those belonging to the libraries prepared from inflorescences (AxIDT, AxIRP, Col, ein5l, GSM278333, GSM278334, GSM278335, TWF, and Tx4F) were denoted by gray symbols.
(PDF)

Figure S7 **GO (Gene Ontology) term enrichment analysis of the validated targets of the organ-specific microRNAs in ARGONAUTE 1 (AGO1) of** ***Arabidopsis*** **.** Based on the small RNA high-throughput sequencing data (GSM707682, GSM707683, GSM707684, and GSM707685), the targets of the organ-specific microRNAs only identified from the AGO1-related library group were included for this analysis. (A) Analysis of the targets of the leaf-specific microRNAs within the “Biological Process” category. (B) Analysis of the targets of the flower-specific microRNAs within the “Biological Process” category. (C) Analysis of the targets of the seedling-specific microRNAs within the “Biological Process” category. This analysis was performed by using agriGO [29], selecting the “Arabidopsis genome locus (TAIR)” as a control set. For (A) to (C), the figure keys are shown in (D).
(PDF)

Table S1 Expression of the 266 miRBase-registered microRNA(*)s in different organs in ***Arabidopsis*** **.** All the high-throughput sequencing data sets were retrieved from GEO (Gene Expression Omnibus; http://www.ncbi.nlm.nih.gov/geo/) [68]: WT_Flower (GSM707678), WT_Leaf (GSM707679), WT_Root (GSM707680), WT_Seedling (GSM707681), AGO1_Flower (GSM707682), AGO1_Leaf (GSM707683), AGO1_Root (GSM707684), AGO1_Seedling (GSM707685), AGO4_Flower (GSM707686), AGO4_Leaf (GSM707687), AGO4_Root (GSM707688), and AGO4_Seedling (GSM707689). The expression levels were shown by normalized read counts (in RPM; reads per million).
(PDF)

Table S2 **List of the organ-specific microRNAs identified from the WT (wild type)-related library group.** For each organ-specific microRNA selected, the expression level in a specific organ (highlighted by different background colors) should be three times or more higher than the other two or three organs. The high-throughput sequencing data sets were retrieved from GEO (Gene Expression Omnibus; http://www.ncbi.nlm.nih.gov/geo/) [68]: WT_Flower (GSM707678), WT_Leaf (GSM707679), WT_Root (GSM707680), WT_Seedling (GSM707681). The expression levels were shown by normalized read counts (in RPM; reads per million).
(PDF)

Table S3 **List of the organ-specific microRNAs identified from the AGO1 (ARGONAUTE 1)-related library group.** For each organ-specific microRNA selected, the expression level in a specific organ (highlighted by different background colors) should be three times or more higher than the other two or three organs. All the high-throughput sequencing data sets were retrieved from GEO (Gene Expression Omnibus; http://www.ncbi.nlm.nih.gov/geo/) [68]: AGO1_Flower (GSM707682), AGO1_Leaf (GSM707683), AGO1_Root (GSM707684), AGO1_Seedling (GSM707685). The expression levels were shown by normalized read counts (in RPM; reads per million).
(PDF)

Table S4 **List of the organ-specific microRNAs from the AGO4 (ARGONAUTE 4)-related library group.** For each organ-specific microRNA selected, the expression level in a specific organ (highlighted by different background colors) should be three times or much higher than the other two or three organs. All the high-throughput sequencing data sets were retrieved from GEO (Gene Expression Omnibus; http://www.ncbi.nlm.nih.gov/geo/) [68]: AGO4_Flower (GSM707686), AGO4_Leaf (GSM707687), AGO4_Root (GSM707688), and AGO4_Seedling (GSM707689). The expression levels were shown by normalized read counts (in RPM; reads per million).
(PDF)

Table S5 **List of AGO4-enriched microRNAs.**
(XLS)

Table S6 **List of the microRNAs not enriched in AGO4. These microRNAs were treated as the control set.**
(XLS)

Table S7 **List of the identified potential poly(A) sites of certain organ-specific microRNAs.** “Strand”: the strand information of the listed pre-miRNAs (precursor microRNAs). “Match site”: the location of the 5′ end of a “MPSS tag” within the downstream region of the pre-miRNA. The expression levels (in RPM; reads per million) of each “MPSS tag” in the 13 libraries (“INF”, “INS”, “AP1”, “AP3”, “AGM”, and “SAP” were prepared from floral organ; “S04”, “S52”, “LES”, and “LEF” were prepared from leaves; “ROS” and “ROF” were prepared from roots; “GSE” was prepared from seedlings) were provided.
(XLS)

Table S8 **List of the identified microRNA gene clusters.** The two pre-miRNAs (precursor microRNAs) with genomic distance less than 10 kb (kilobases) were treated as a cluster. The genomic locations of the pre-miRNAs were retrieved from miRBase (release 17).
(XLS)

Table S9 **List of the microRNA–target pairs identified based on degradome sequencing data.**
(XLS)
