# Supplementary material for: Expression-Based Functional Investigation of the Organ-Specific MicroRNAs in Arabidopsis
Source: PLoS One. 2012 Nov 30;7(11):e50870. doi: 10.1371/journal.pone.0050870 (PMC3511311; doi:10.1371/journal.pone.0050870)
Supplement: Figure S1 — Workflow of the main analyses in this study. (PDF) [file pone.0050870.s001.pdf]

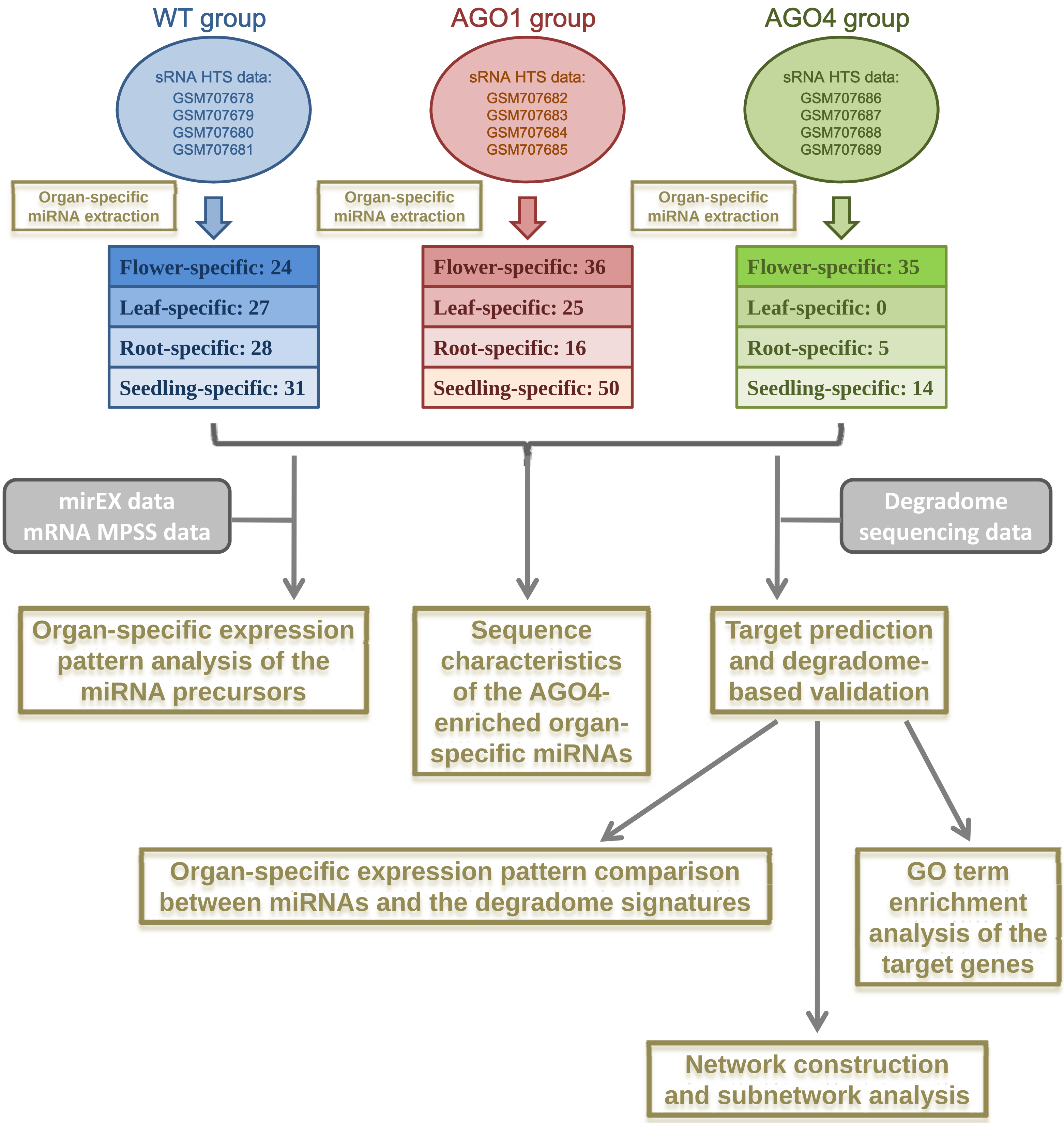
WT group
sRNA HTS data:
GSM707678
GSM707679
GSM707680
GSM707681
AGO1 group
sRNA HTS data:
GSM707682
GSM707683
GSM707684
GSM707685
AGO4 group
sRNA HTS data:
GSM707686
GSM707687
GSM707688
GSM707689
Organ-specific miRNA extraction
Organ-specific miRNA extraction
Organ-specific miRNA extraction
Flower-specific: 24
Leaf-specific: 27
Root-specific: 28
Seedling-specific: 31
Flower-specific: 36
Leaf-specific: 25
Root-specific: 16
Seedling-specific: 50
Flower-specific: 35
Leaf-specific: 0
Root-specific: 5
Seedling-specific: 14
mirEX data
mRNA MPSS data
Degradome sequencing data
Organ-specific expression pattern analysis of the miRNA precursors
Sequence characteristics of the AGO4-enriched organ-specific miRNAs
Target prediction and degradome-based validation
Organ-specific expression pattern comparison between miRNAs and the degradome signatures
GO term enrichment analysis of the target genes
Network construction and subnetwork analysis
