# Supplementary material for: Expression-Based Functional Investigation of the Organ-Specific MicroRNAs in Arabidopsis
Source: PLoS One. 2012 Nov 30;7(11):e50870. doi: 10.1371/journal.pone.0050870 (PMC3511311; doi:10.1371/journal.pone.0050870)
Supplement: Figure S2 — List of the microRNA genes with consistent expression patterns between the mature miRNAs (based on small RNA high-throughput sequencing data) and the miRNA precursors (based on the data provided by mirEX). For the mature miRNAs listed in the tables, their detectable expression levels (normalized in RPM; reads per million) in flowers, leaves, roots and seedlings were highlighted in gray background. The small RNA high-throughput sequencing data sets were retrieved from GEO (Gene Expression Omnibus; http://www.ncbi.nlm.nih.gov/geo/) [68]: WT_Flower, GSM707678; WT_Leaf, GSM707679; WT_Root, GSM707680; WT_Seedling, GSM707681. Accordingly, the mirEX-derived expression levels of the pre-miRNA/pri-miRNA detected by real-time quantitative PCR [PP2A (phosphatase 2A; AT1G13320) as the reference gene] in the similar organs were highlighted in pink background. Please note: the y axis is in log scale. (PDF) [file pone.0050870.s002.pdf]

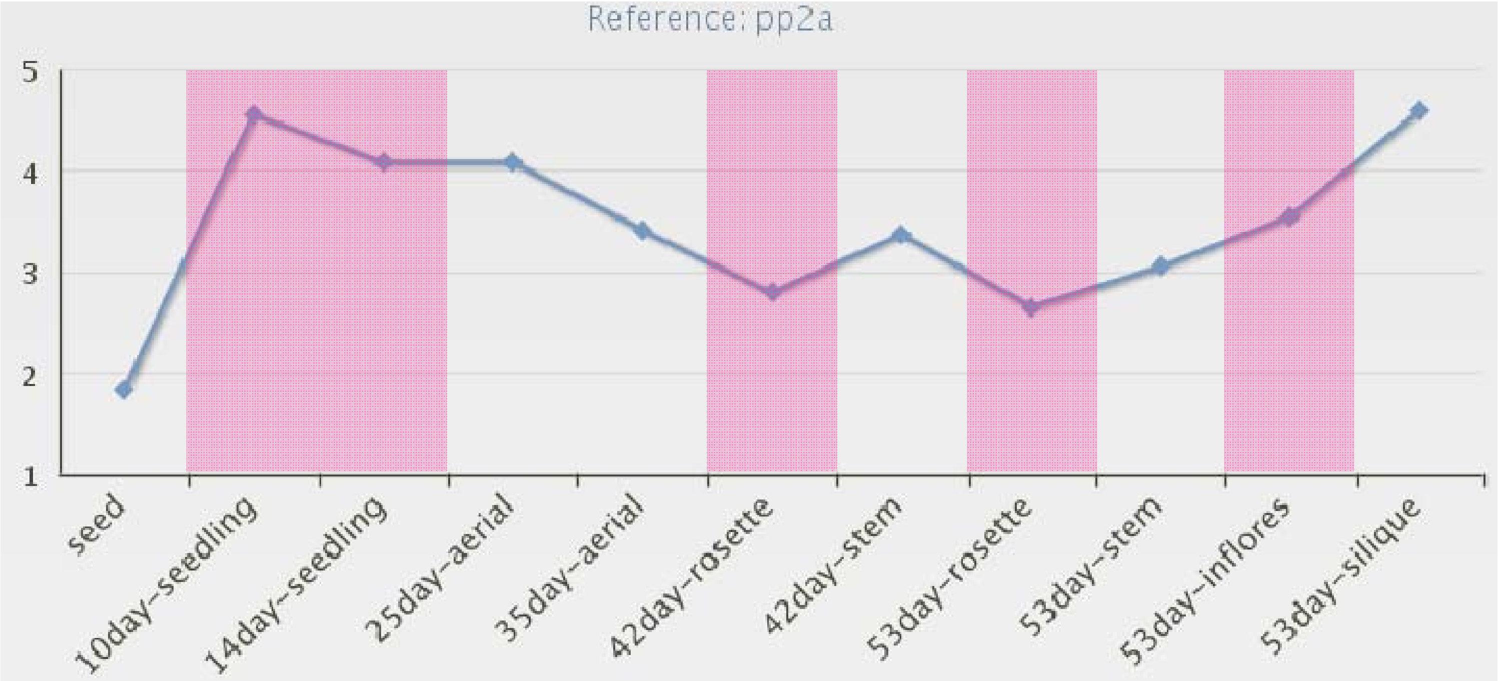


| miRNA | WT_Flower | WT_Leaf | WT_Root | WT_Seedling |
|---|---|---|---|---|
| ath-miR169b | 4.23 | 3.8 | 90.37 | 22.01 |

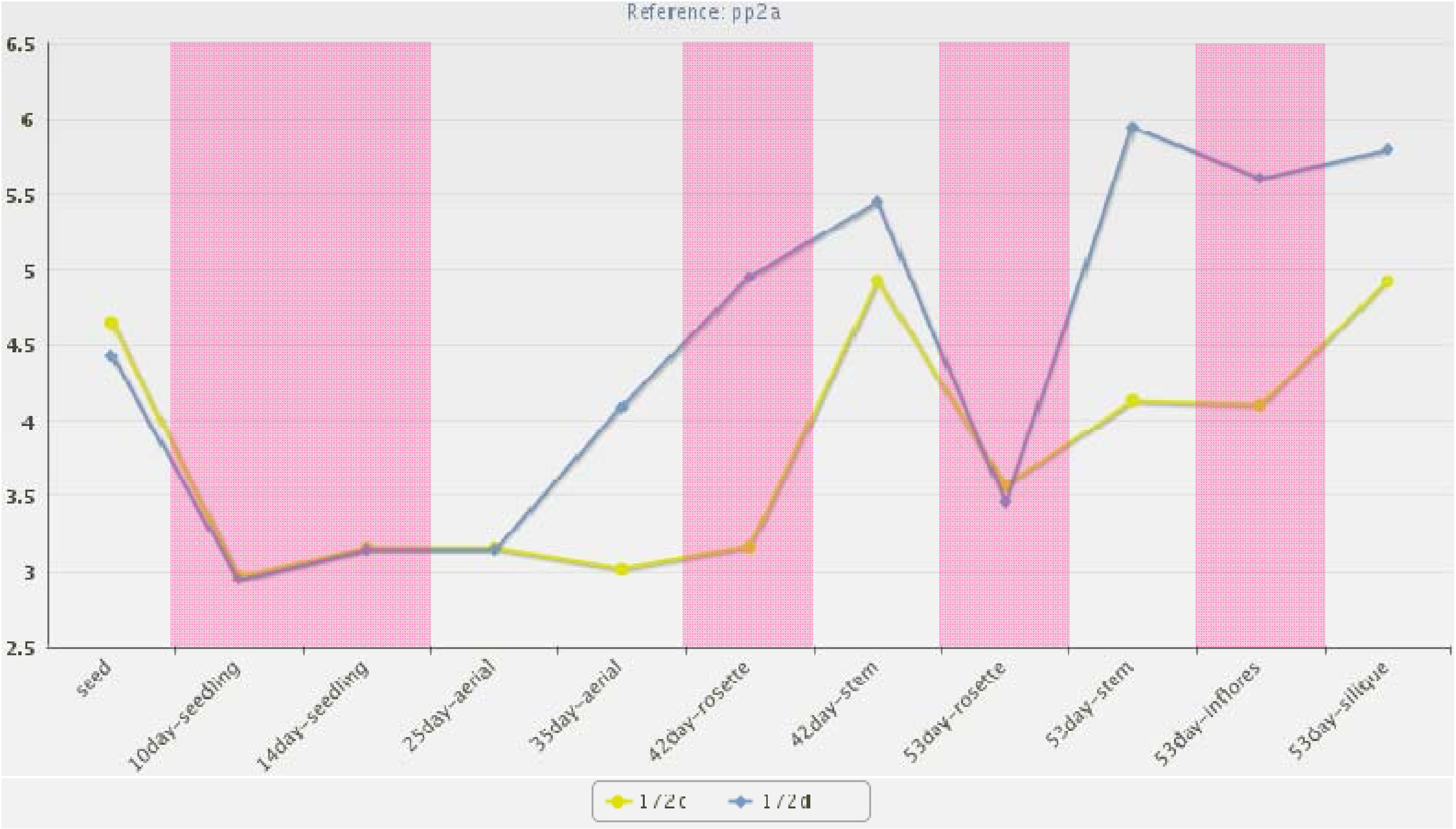


| miRNA | WT_Flower | WT_Leaf | WT_Root | WT_Seedling |
|---|---|---|---|---|
| ath-miR172c | 617.94 | 6.8 | 562.68 | 16.28 |
| ath-miR172d | 617.94 | 6.8 | 562.68 | 16.28 |

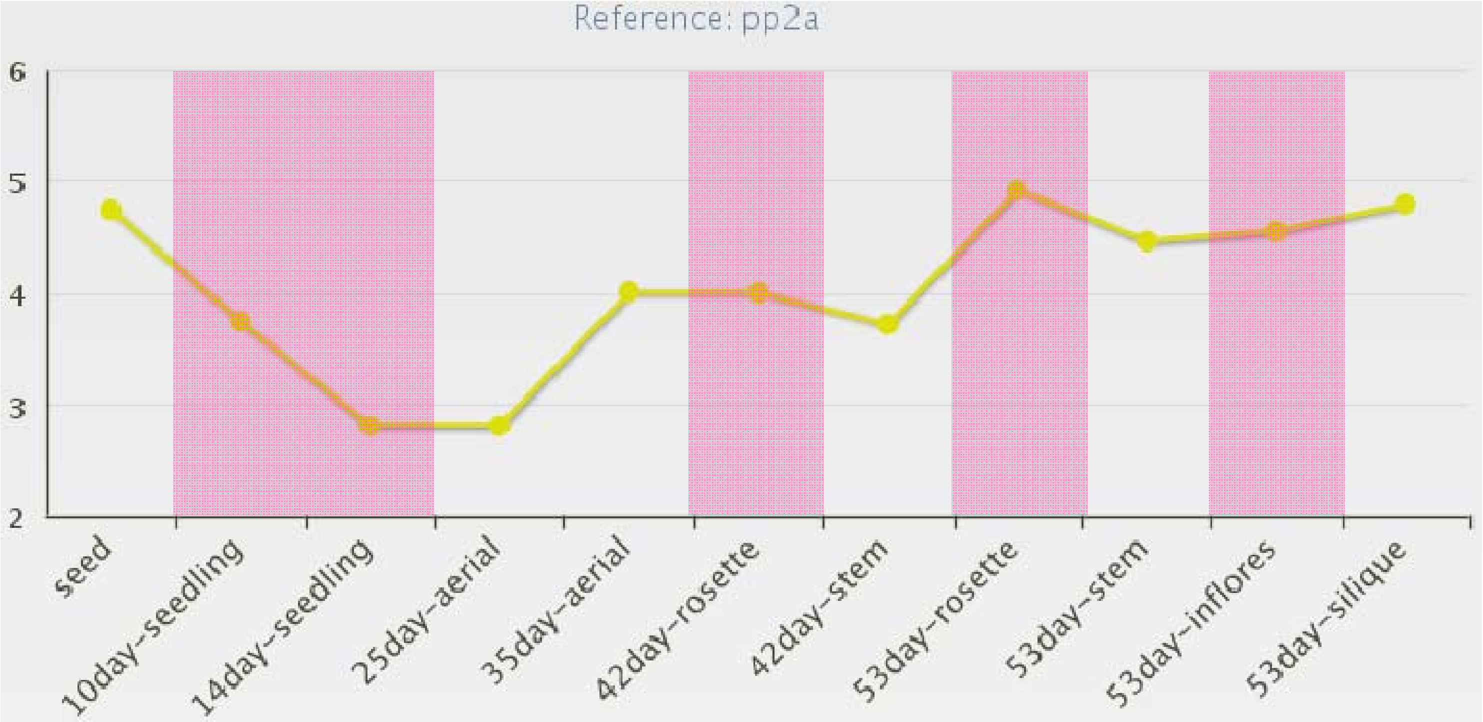


| miRNA | WT_Flower | WT_Leaf | WT_Root | WT_Seedling |
|---|---|---|---|---|
| ath-miR391 | 38.27 | 30.21 | 1.15 | 6.47 |

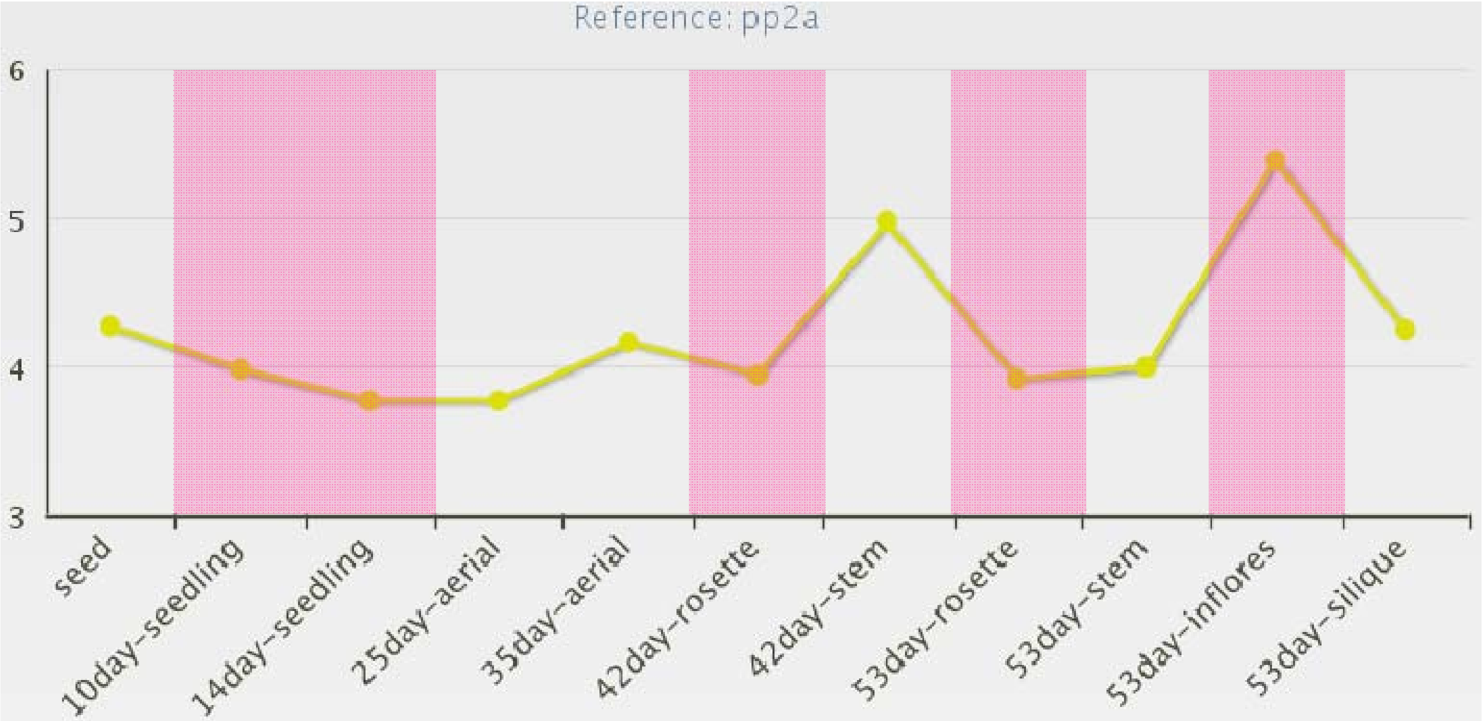


| miRNA | WT_Flower | WT_Leaf | WT_Root | WT_Seedling |
|---|---|---|---|---|
| ath-miR771 | 19.94 | 0.4 | 0 | 0 |

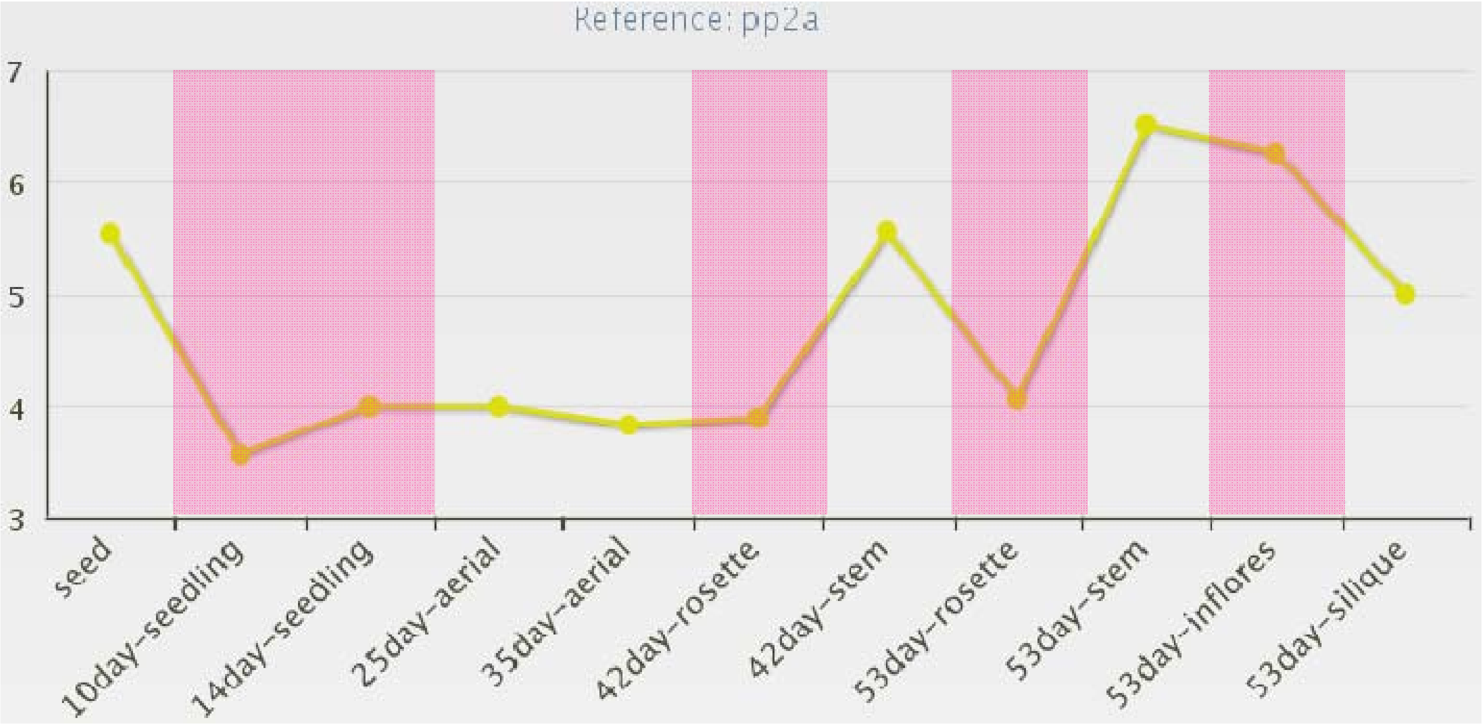


| miRNA | WT_Flower | WT_Leaf | WT_Root | WT_Seedling |
|---|---|---|---|---|
| ath-miR780.1 | 103.33 | 0 | 0 | 0 |
| ath-miR780.2 | 597.39 | 1 | 2.59 | 0.37 |

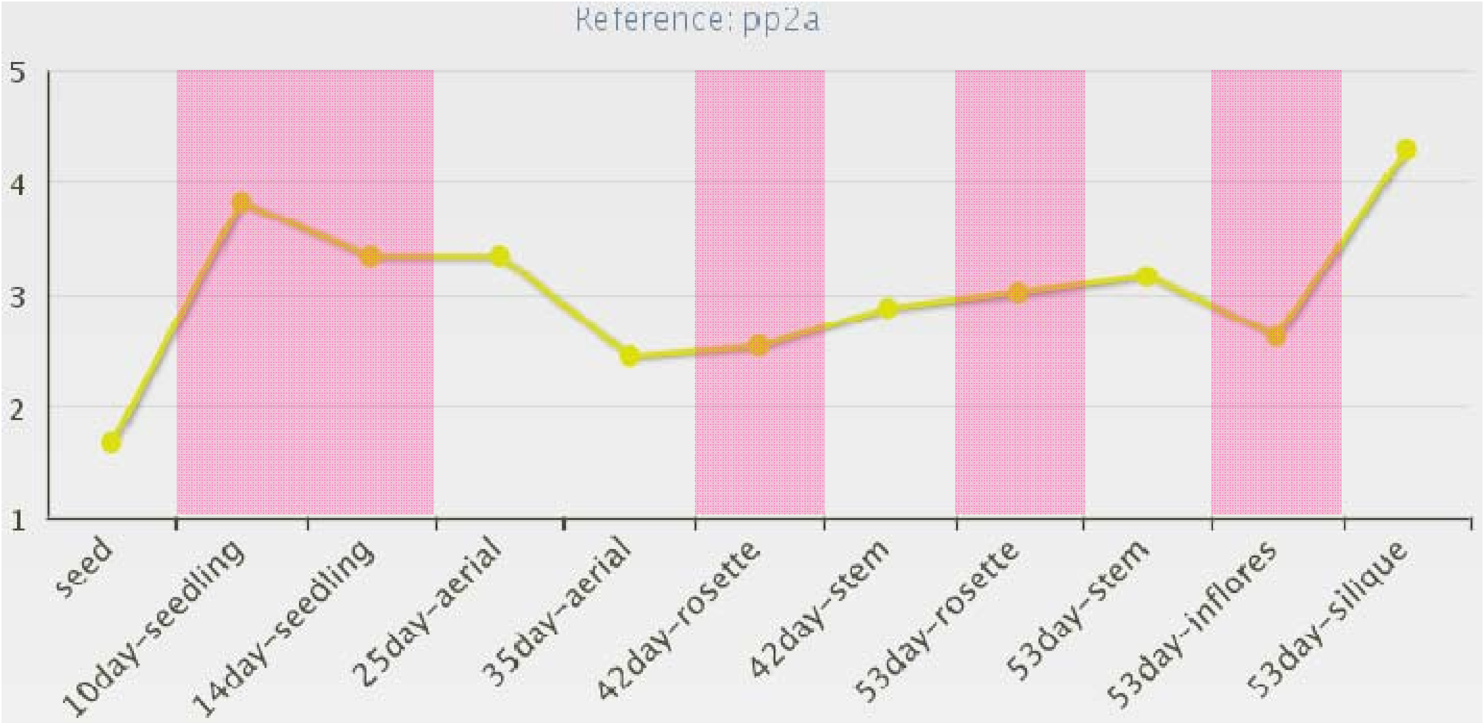


| miRNA | WT_Flower | WT_Leaf | WT_Root | WT_Seedling |
|---|---|---|---|---|
| ath-miR837-3p | 1.01 | 3 | 24.46 | 10.36 |
| ath-miR837-5p | 0.2 | 1 | 12.09 | 6.47 |

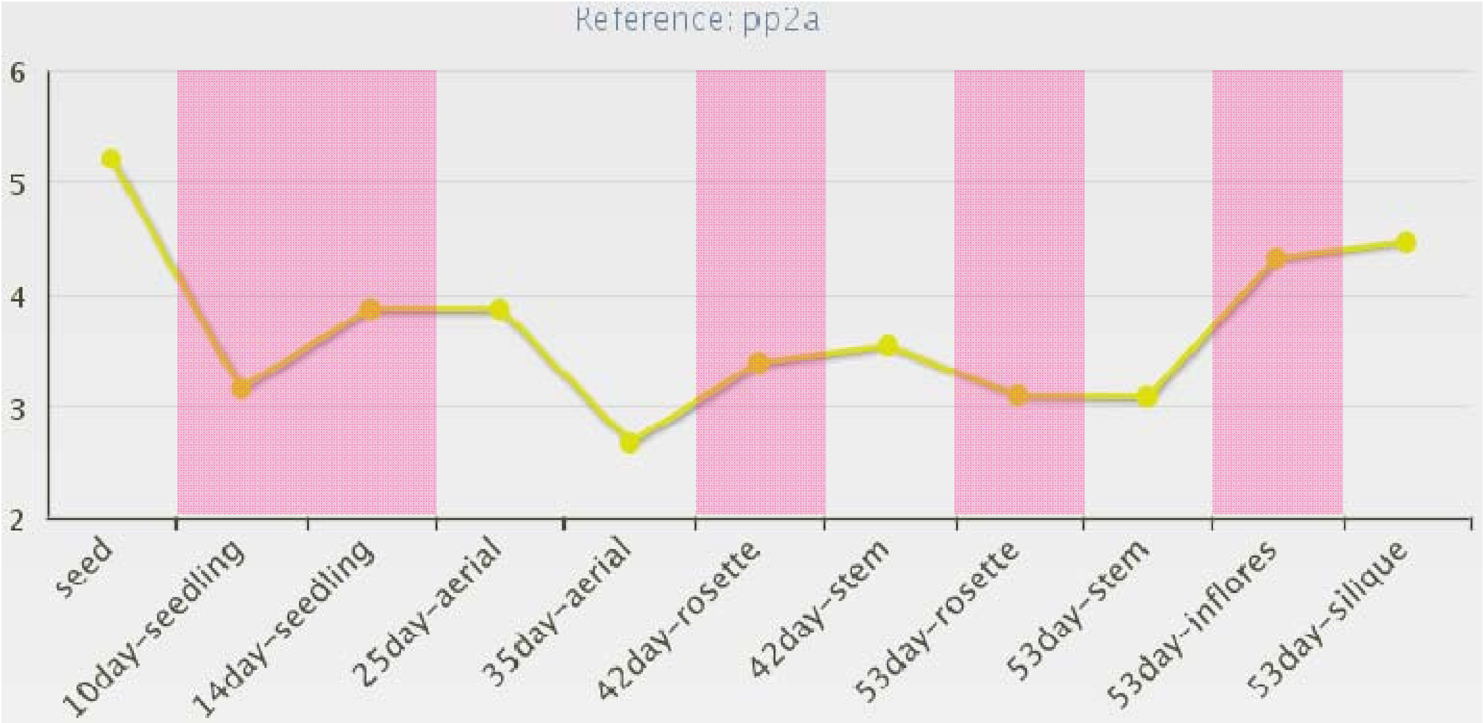


| miRNA | WT_Flower | WT_Leaf | WT_Root | WT_Seedling |
|---|---|---|---|---|
| ath-miR845a | 54.58 | 5.4 | 1.73 | 3.88 |

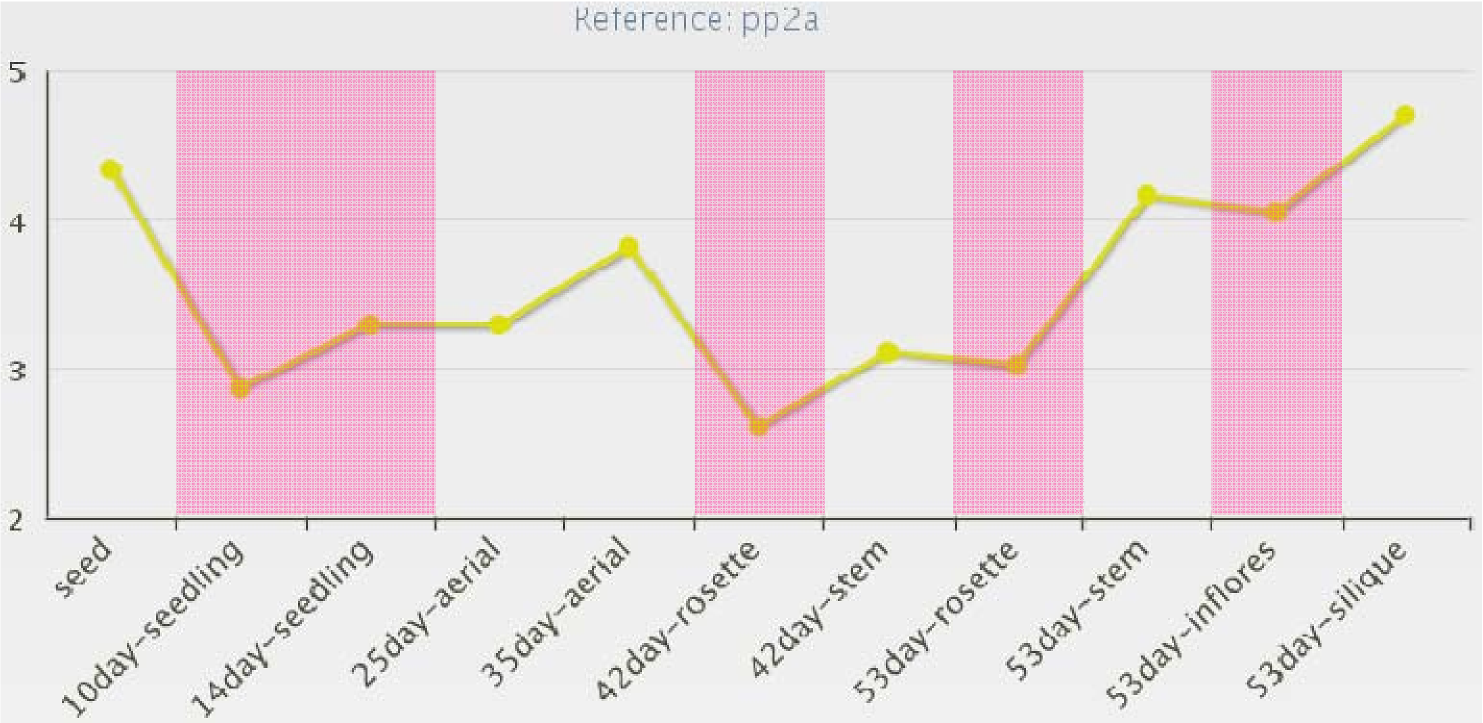


| miRNA | WT_Flower | WT_Leaf | WT_Root | WT_Seedling |
|---|---|---|---|---|
| ath-miR851-5p | 20.14 | 1 | 0 | 0.18 |

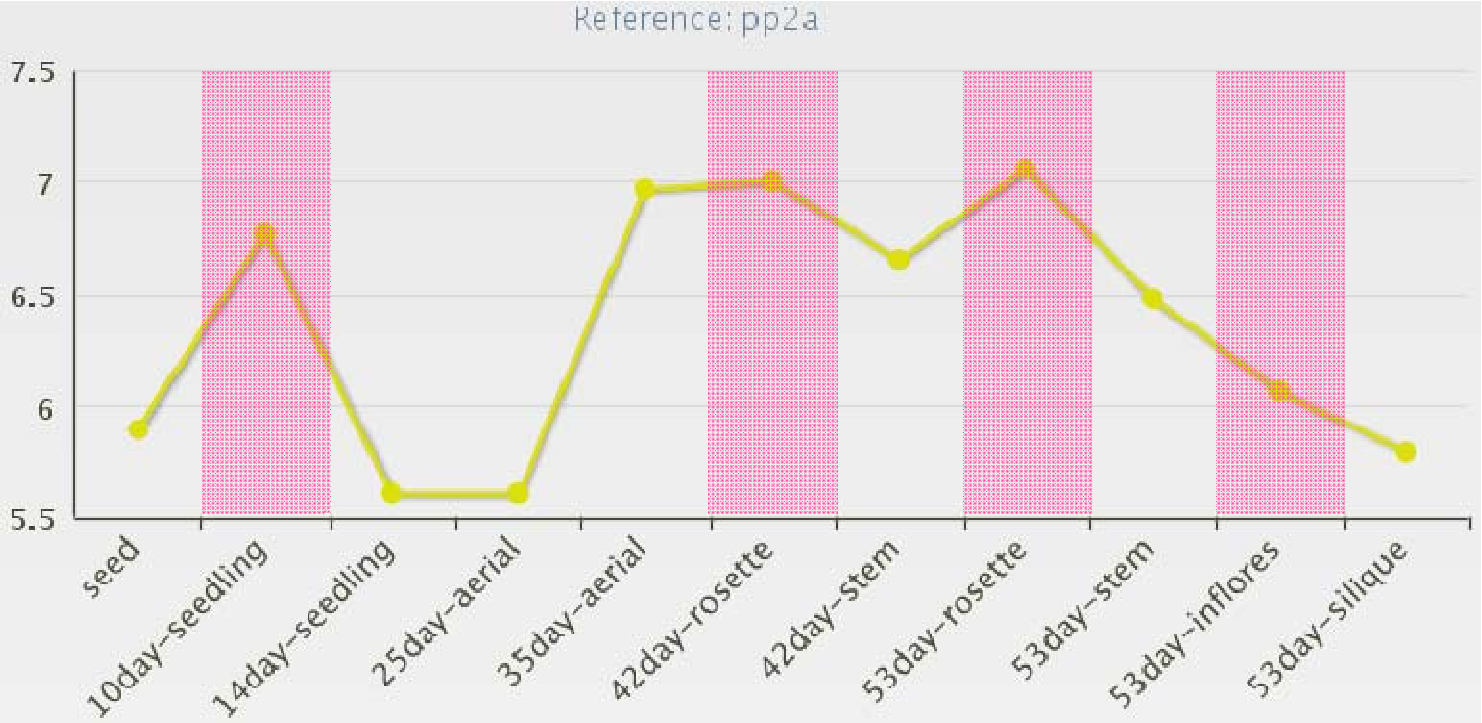


| miRNA | WT_Flower | WT_Leaf | WT_Root | WT_Seedling |
| --- | --- | --- | --- | --- |
| ath-miR825 | 17.12 | 79.42 | 0.29 | 46.24 |

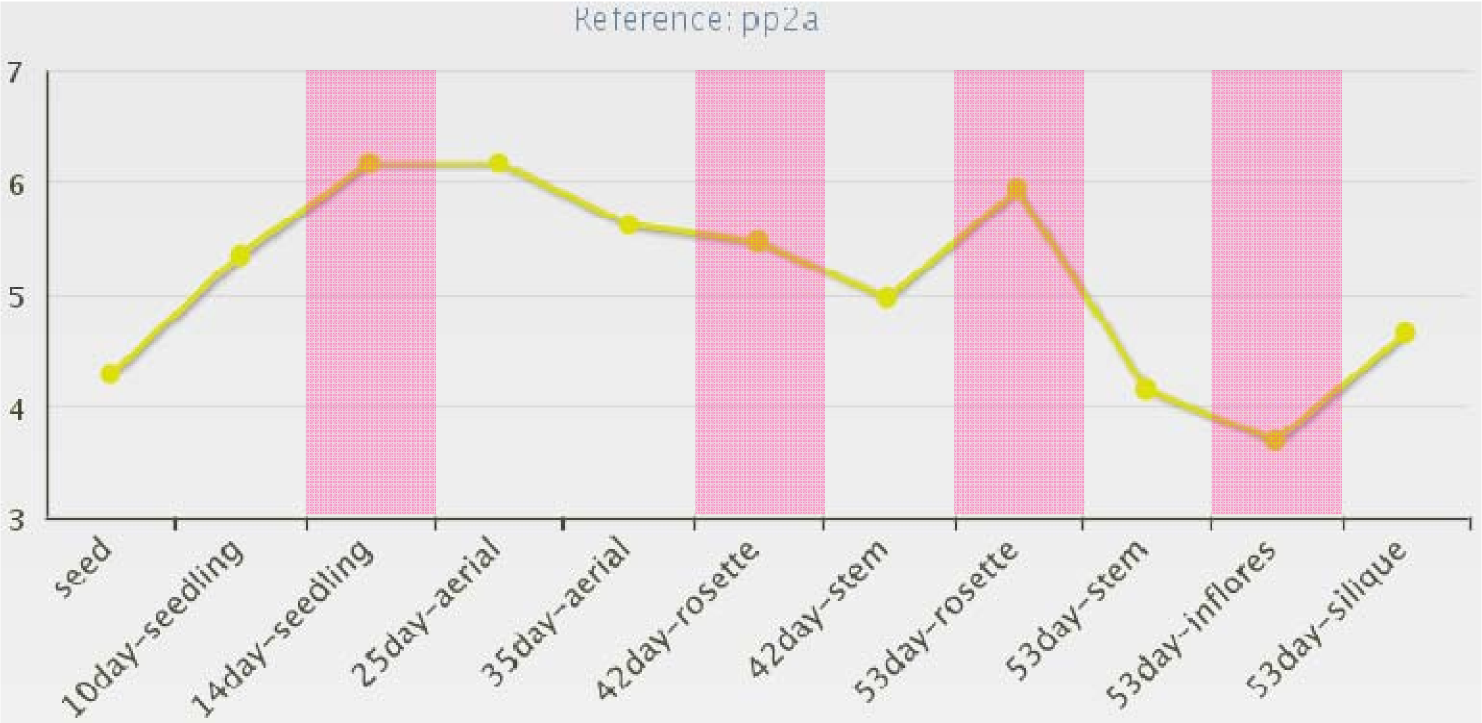


| miRNA | WT_Flower | WT_Leaf | WT_Root | WT_Seedling |
|---|---|---|---|---|
| ath-miR841 | 0.81 | 12.2 | 0 | 34.03 |

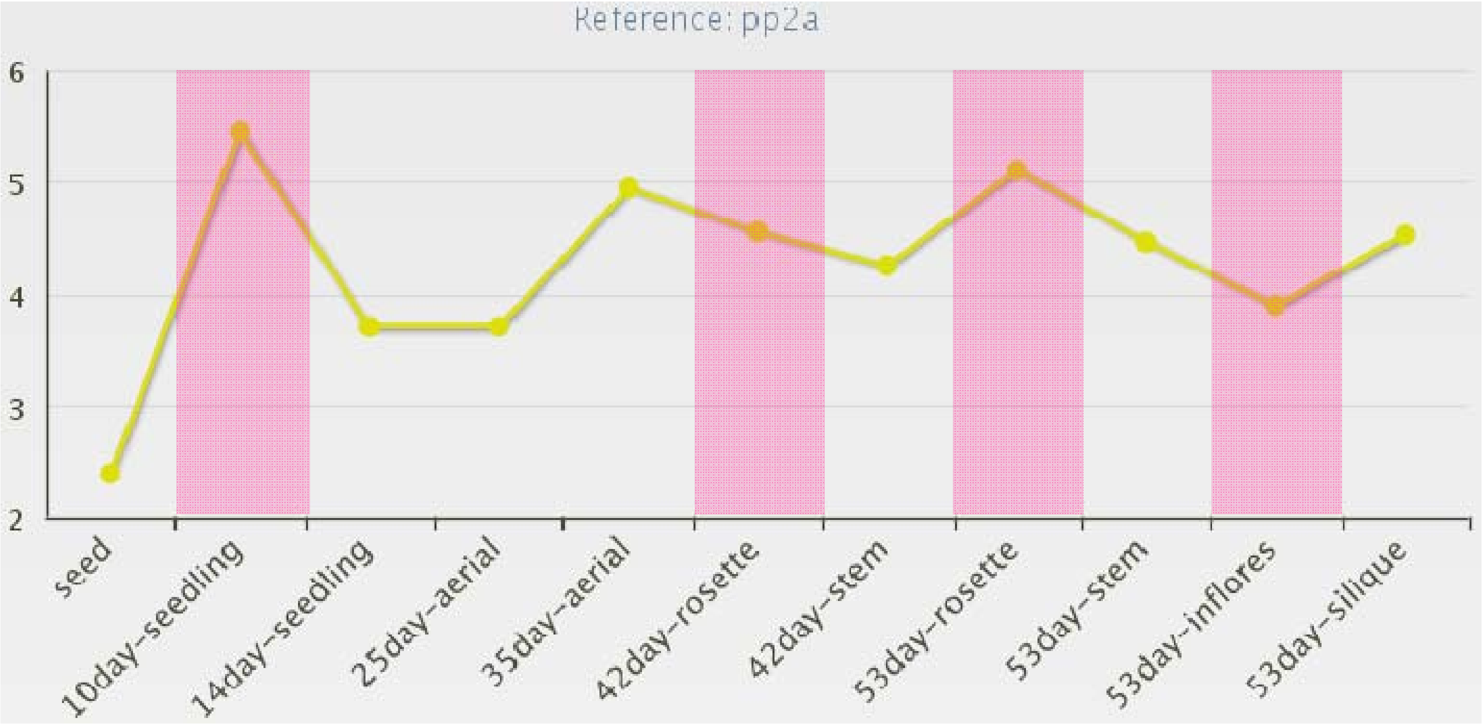


| miRNA | WT_Flower | WT_Leaf | WT_Root | WT_Seedling |
|---|---|---|---|---|
| ath-miR857 | 0 | 0 | 0 | 14.06 |

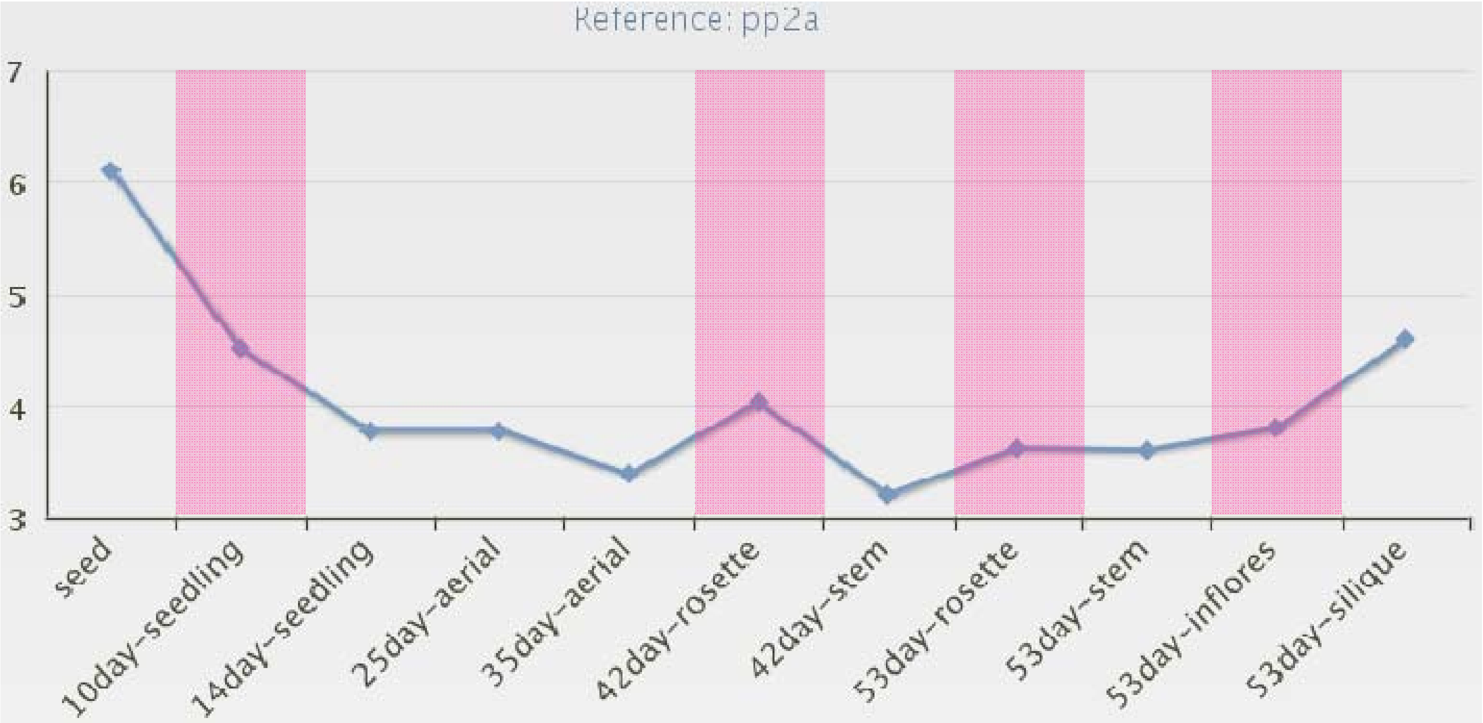


| miRNA | WT_Flower | WT_Leaf | WT_Root | WT_Seedling |
|---|---|---|---|---|
| ath-miR2111b* | 3.02 | 1.8 | 3.74 | 18.87 |
