# Supplementary material for: Expression-Based Functional Investigation of the Organ-Specific MicroRNAs in Arabidopsis
Source: PLoS One. 2012 Nov 30;7(11):e50870. doi: 10.1371/journal.pone.0050870 (PMC3511311; doi:10.1371/journal.pone.0050870)
Supplement: Figure S3 — List of the microRNA genes with consistent expression patterns between the mature miRNAs (based on small RNA high-throughput sequencing data) and their precursors (based on the data provided by mirEX and the MPSS data). For the mature miRNAs listed in the first tables, their detectable expression levels (normalized in RPM; reads per million) in flowers, leaves, roots and seedlings were highlighted in different background. The HTS data sets were retrieved from GEO (Gene Expression Omnibus; http://www.ncbi.nlm.nih.gov/geo/) [68]: WT_Flower, GSM707678; WT_Leaf, GSM707679; WT_Root, GSM707680; WT_Seedling, GSM707681. For the pri-miRNAs listed in the second tables, the detectable levels of the identified poly(A) signals based on MPSS (massively parallel signature sequencing) data were highlighted by the same background colors as above according to the organs analyzed. The MPSS data sets were retrieved from Next-Gen Sequence Databases (http://mpss.udel.edu/at/mpss_index.php) [18]. The libraries INF, INS, AP1, AP3, AGM and SAP were prepared from Arabidopsis flowers (indicated by yellow background). S04, S52, LES and LEF were prepared from leaves (green background). ROS and ROF were prepared from roots (gray), and GSE from young seedlings (red). The expression levels of the pre-miRNAs/pri-miRNAs detected by real-time PCR [PP2A (phosphatase 2A; AT1G13320) as the reference gene] in the similar organs retrieved from mirEX (http://comgen.pl/mirex/) [17] were also highlighted in different background as indicated above. Please note: the y axis is in log scale. (PDF) [file pone.0050870.s003.pdf]

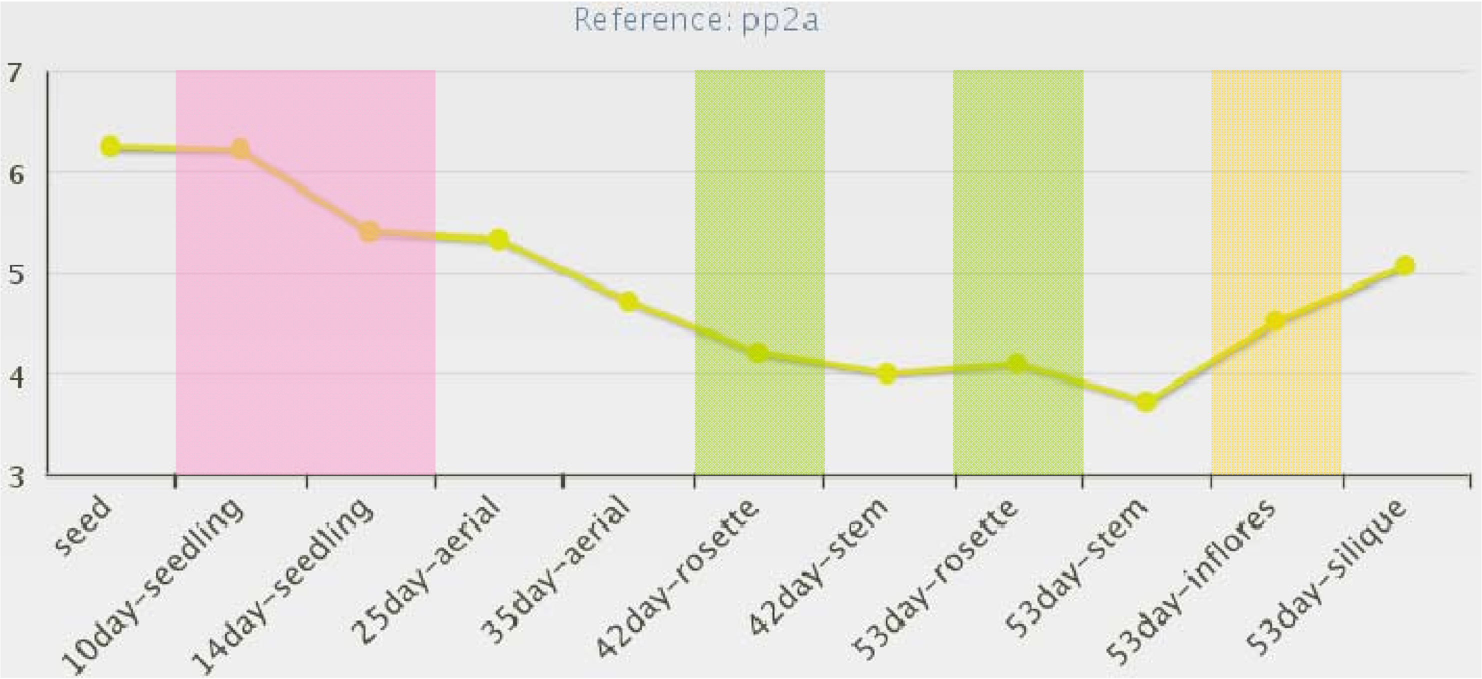

miRNA	WT_Flower	WT_Leaf	WT_Root	WT_Seedling
ath-miR156a	108.36	238.06	51.23	1495.53

Pri-miRNA	Match site	Sequence	INF	INS	AP1	AP3	AGM	SAP	S04	S52	LES	LEF	ROS	ROF	GSE
ath-MIR156a	5204	GATCTTCAAGGCGGAAT	0	0	0	0	0	0	0	0	0	0	0	0	9
	5204	GATCTTCAAGGCGGAATGCG	0	0	0	0	0	0	0	0	0	0	0	0	11
	6873	GATCGGCGCTGTGCTTG	0	0	0	0	0	0	3	3	9	0	0	0	18
	6873	GATCGGCGCTGTGCTTGGTC	0	0	0	0	0	0	3	3	9	0	0	0	23
	7029	GATCTCGGAGTTTGACC	0	0	0	0	0	0	1	2	2	0	0	0	27
	7029	GATCTCGGAGTTTGACCCGG	0	0	0	0	0	0	1	2	0	0	0	0	34

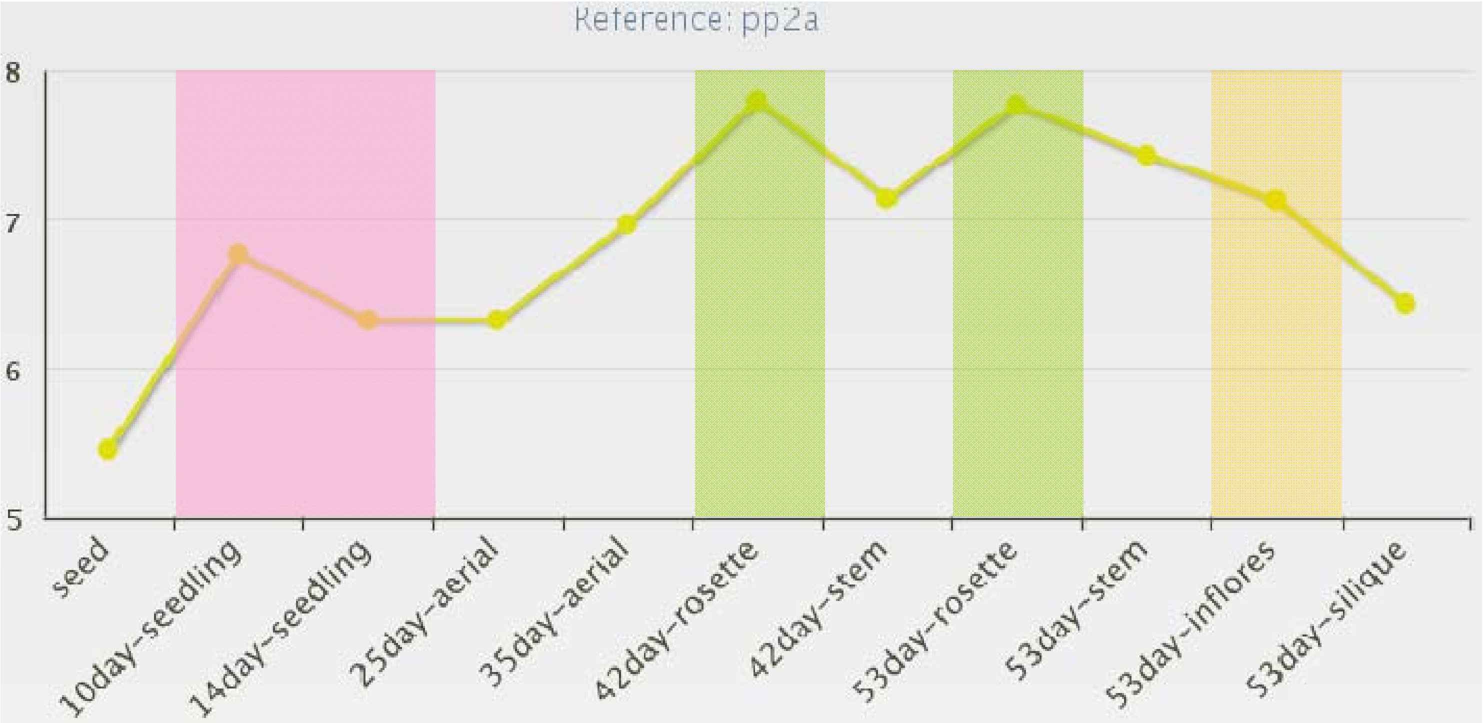

miRNA	WT_Flower	WT_Leaf	WT_Root	WT_Seedling
ath-miR163	3690.3	8029.77	2072.83	2218.51

Pri-miRNA	Match site	Sequence	INF	INS	AP1	AP3	AGM	SAP	S04	S52	LES	LEF	ROS	ROF	GSE
ath-MIR163	3118	GATCAAAGCTATGTCTA	0	0	2	0	0	0	0	0	20	0	0	0	0
	3118	GATCAAAGCTATGTCTATGC	0	0	2	0	0	0	0	0	23	0	0	0	0

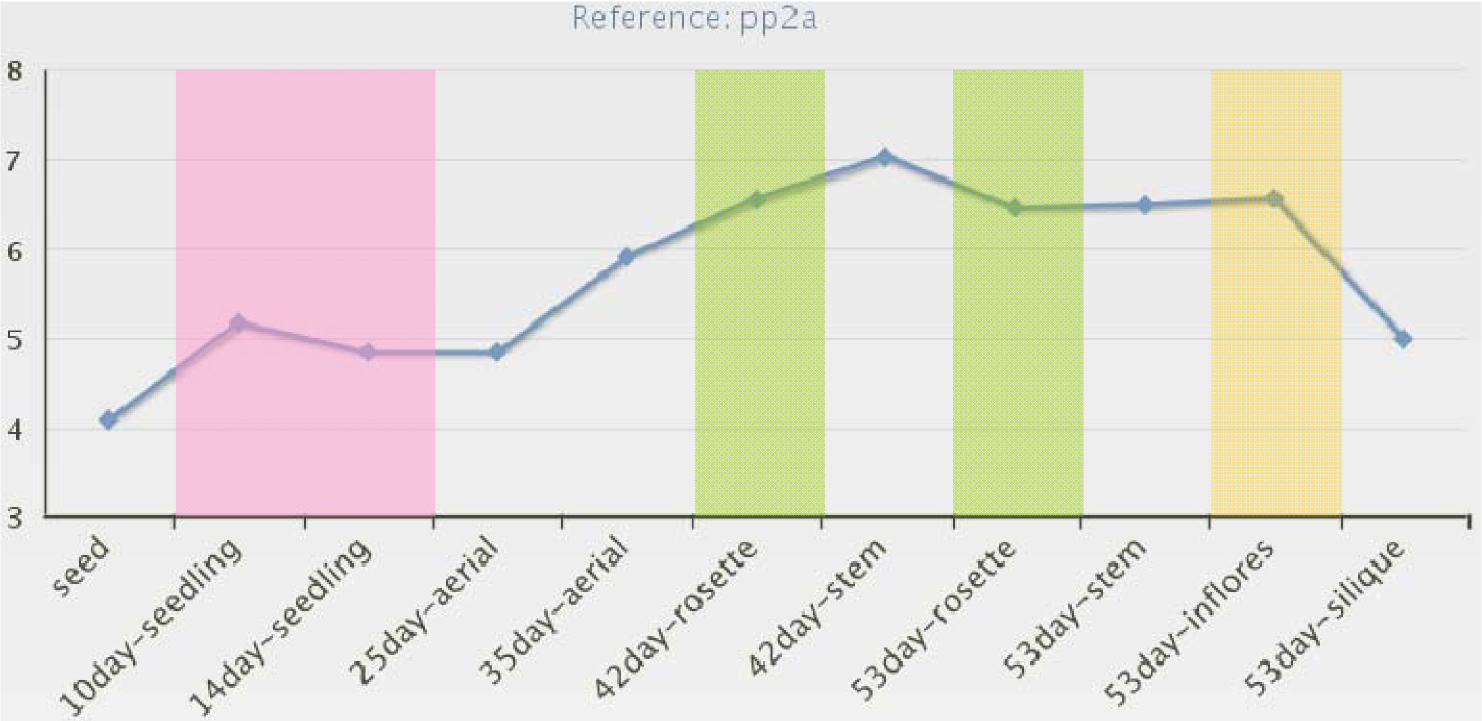

miRNA	WT_Flower	WT_Leaf	WT_Root	WT_Seedling
ath-miR172b*	22.56	76.82	5.18	22.01

Pri-miRNA	Match site	Sequence	INF	INS	AP1	AP3	AGM	SAP	S04	S52	LES	LEF	ROS	ROF	GSE
ath-MIR172b	393	GATCGGCCAGTTCGGTC	9	1	2	3	3	0	0	14	4	1	1	1	0
	393	GATCGGCCAGTTCGGTCACT	11	0	1	3	3	0	0	10	2	1	0	0	0

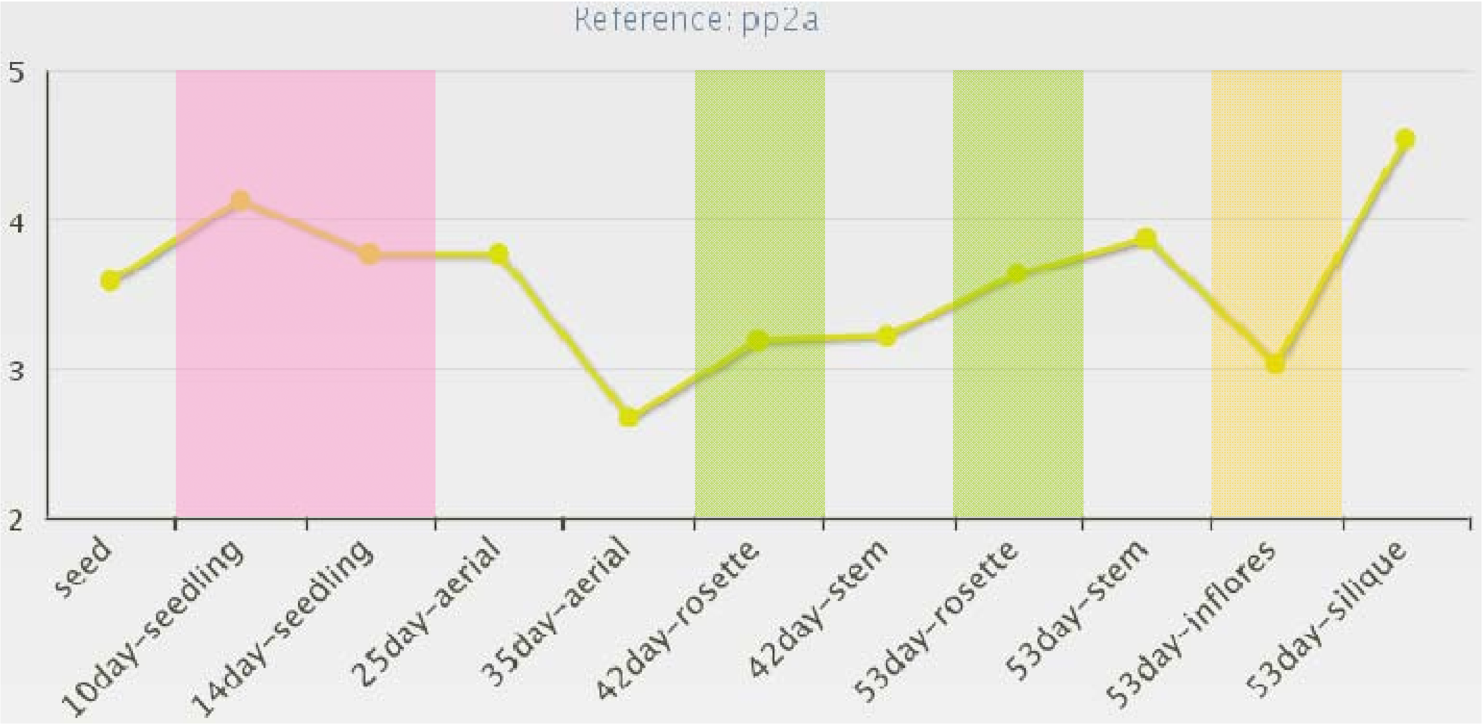

miRNA	WT_Flower	WT_Leaf	WT_Root	WT_Seedling
ath-miR2111a*	0	0	0.86	11.84

Pri-miRNA	Match site	Sequence	INF	INS	AP1	AP3	AGM	SAP	S04	S52	LES	LEF	ROS	ROF	GSE
ath-MIR2111a	5270	GATCTGGTCGATATGAA	0	0	0	0	0	0	0	0	0	0	0	0	40
	5270	GATCTGGTCGATATGAAGTG	0	0	0	0	0	0	0	0	0	0	0	0	49

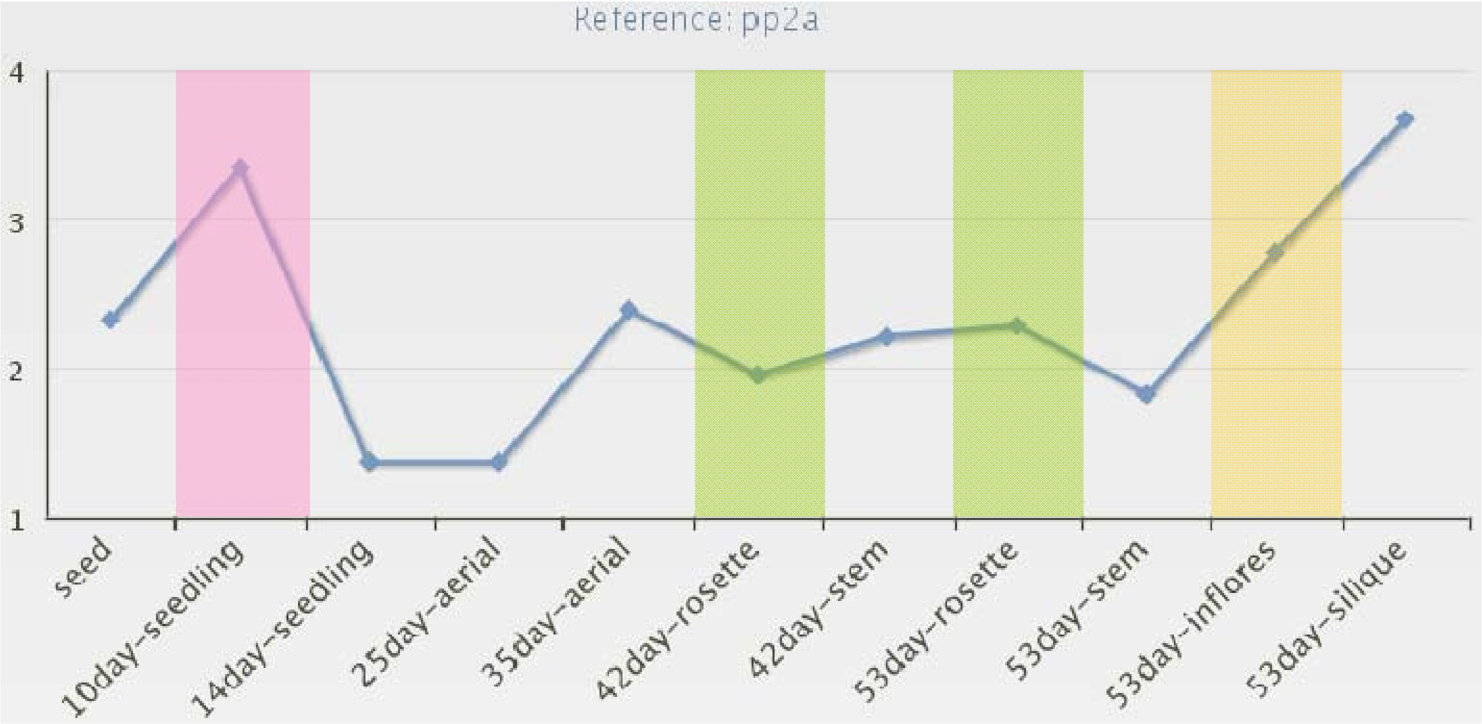

miRNA	WT_Flower	WT_Leaf	WT_Root	WT_Seedling
ath-miR399b	18.53	9.4	4.61	61.4

Pri-miRNA	Match site	Sequence	INF	INS	AP1	AP3	AGM	SAP	S04	S52	LES	LEF	ROS	ROF	GSE
ath-MIR399b	2723	GATCTTGAAGACGACCG	0	0	0	4	0	0	0	0	0	0	0	0	15
	2723	GATCTTGAAGACGACCGGAG	0	0	0	0	0	0	0	0	0	0	0	0	15
	2885	GATCGGAAAGTTTTCGC	0	0	0	0	0	0	0	0	0	0	0	0	9
	2885	GATCGGAAAGTTTTCGCCGG	0	0	0	0	0	0	0	0	0	0	0	3	11

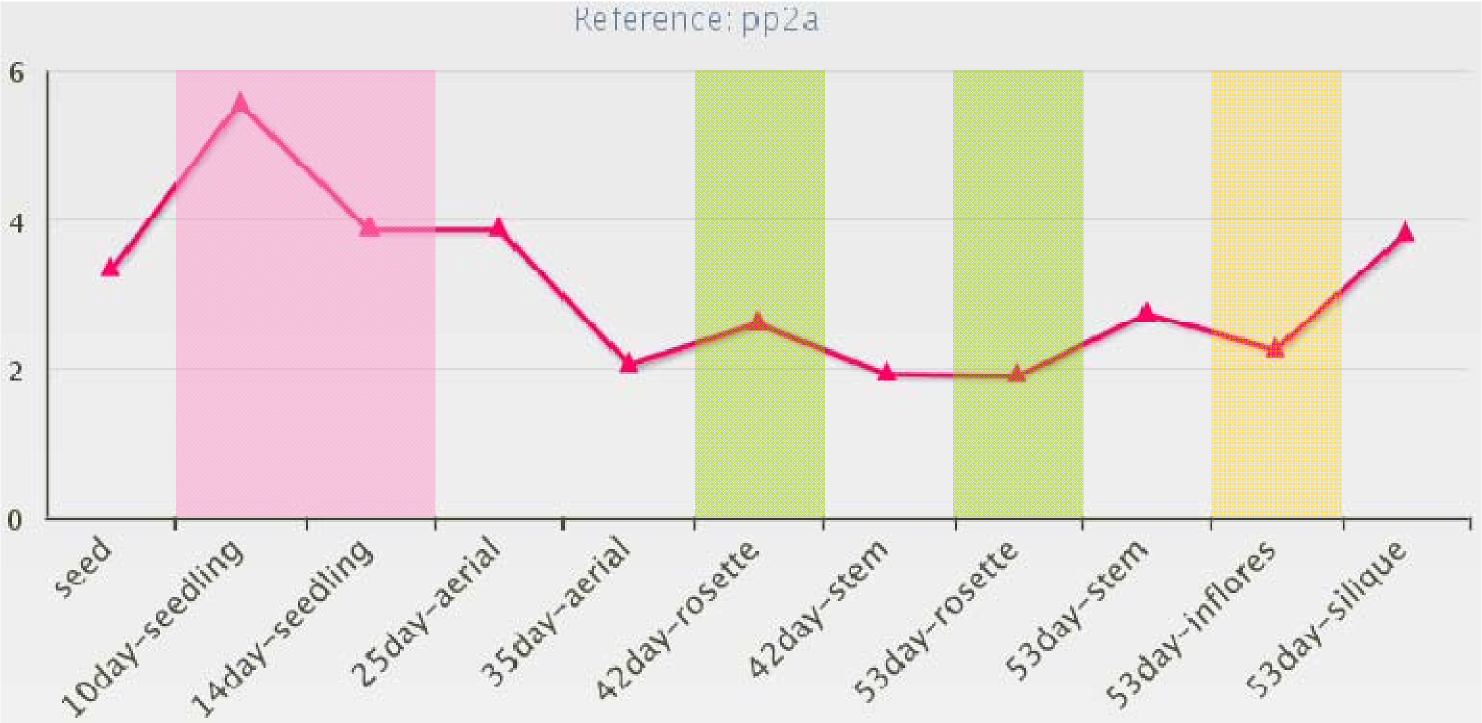

miRNA	WT_Flower	WT_Leaf	WT_Root	WT_Seedling
ath-miR399d	0.2	0	0	19.97

Pri-miRNA	Match site	Sequence	INF	INS	AP1	AP3	AGM	SAP	S04	S52	LES	LEF	ROS	ROF	GSE
ath-MIR399d	4253	GATCCCAATGCCTTGCC	0	0	2	0	0	0	0	0	0	0	0	0	18
	4253	GATCCCAATGCCTTGCCAGC	0	0	2	0	0	0	0	0	0	0	0	0	26

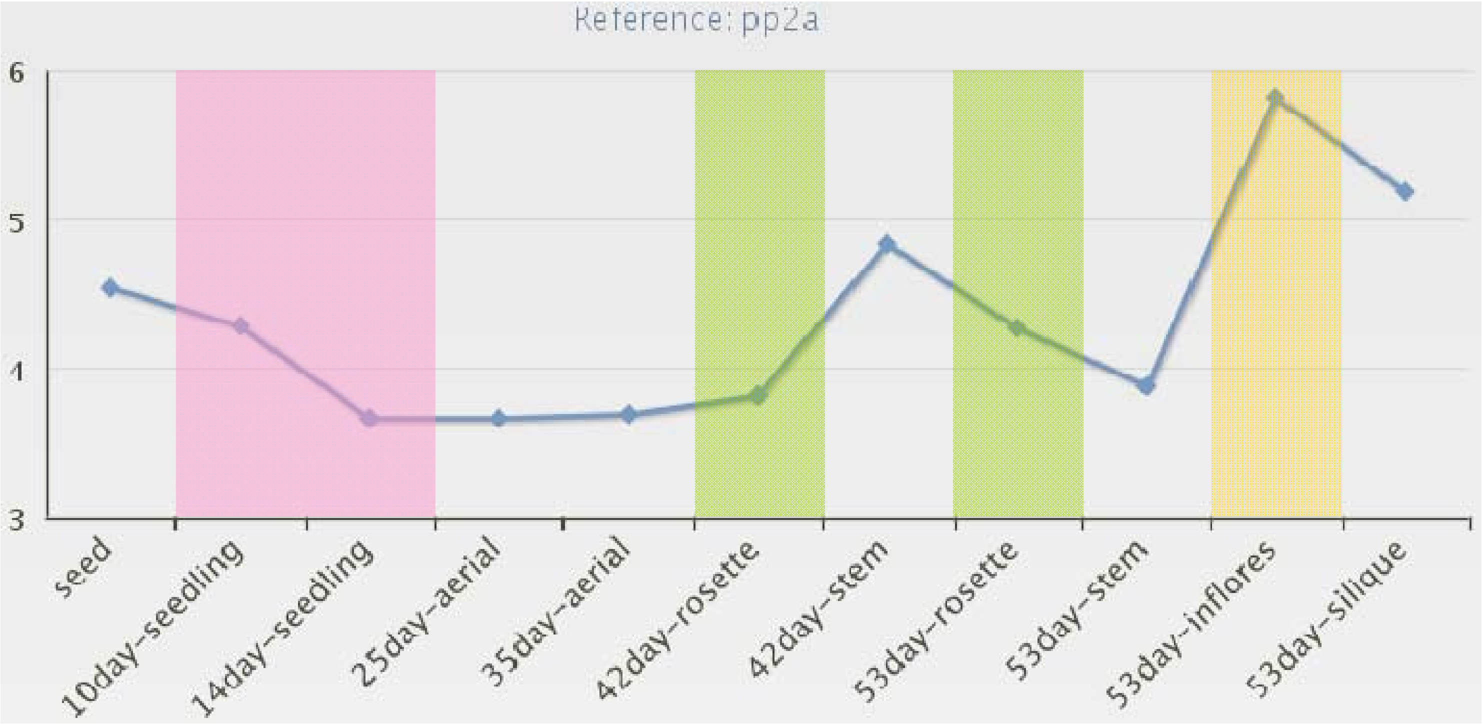

miRNA	WT_Flower	WT_Leaf	WT_Root	WT_Seedling
ath-miR447b	24.37	9.4	3.17	5.73

Pri-miRNA	Match site	Sequence	INF	INS	AP1	AP3	AGM	SAP	S04	S52	LES	LEF	ROS	ROF	GSE
ath-MIR447b	377	GATCACTACTCAGCTTT	8	0	0	1	0	0	0	0	0	0	0	0	0
	377	GATCACTACTCAGCTTTTTC	10	0	0	1	0	0	0	0	0	0	0	0	0

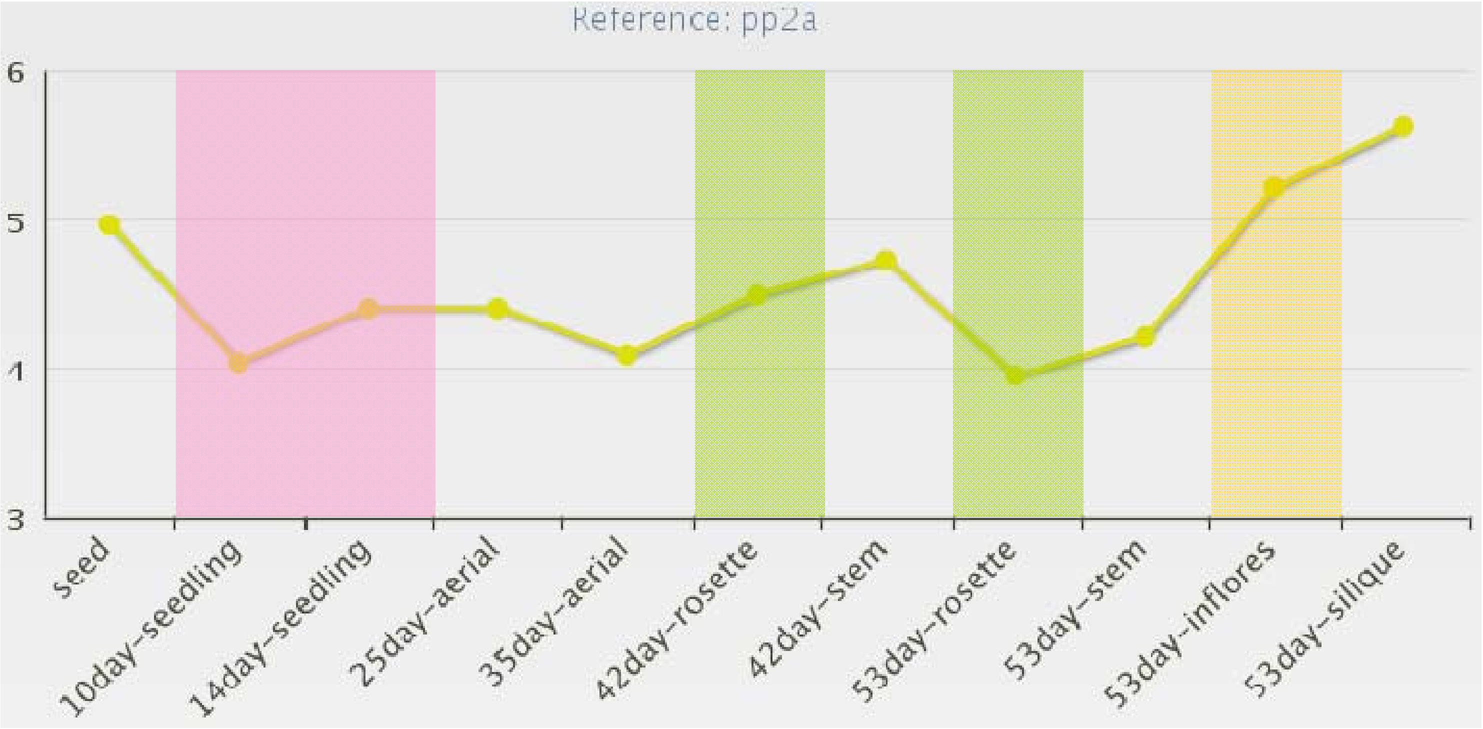

miRNA	WT_Flower	WT_Leaf	WT_Root	WT_Seedling
ath-miR856	28	0	0	0

Pri-miRNA	Match site	Sequence	INF	INS	AP1	AP3	AGM	SAP	S04	S52	LES	LEF	ROS	ROF	GSE
ath-MIR856	9862	GATCTTGTCATGACCCA	16	59	97	96	34	50	0	10	5	32	50	33	17
	9862	GATCTTGTCATGACCCAAGC	12	63	107	100	13	12	0	9	2	30	27	20	13

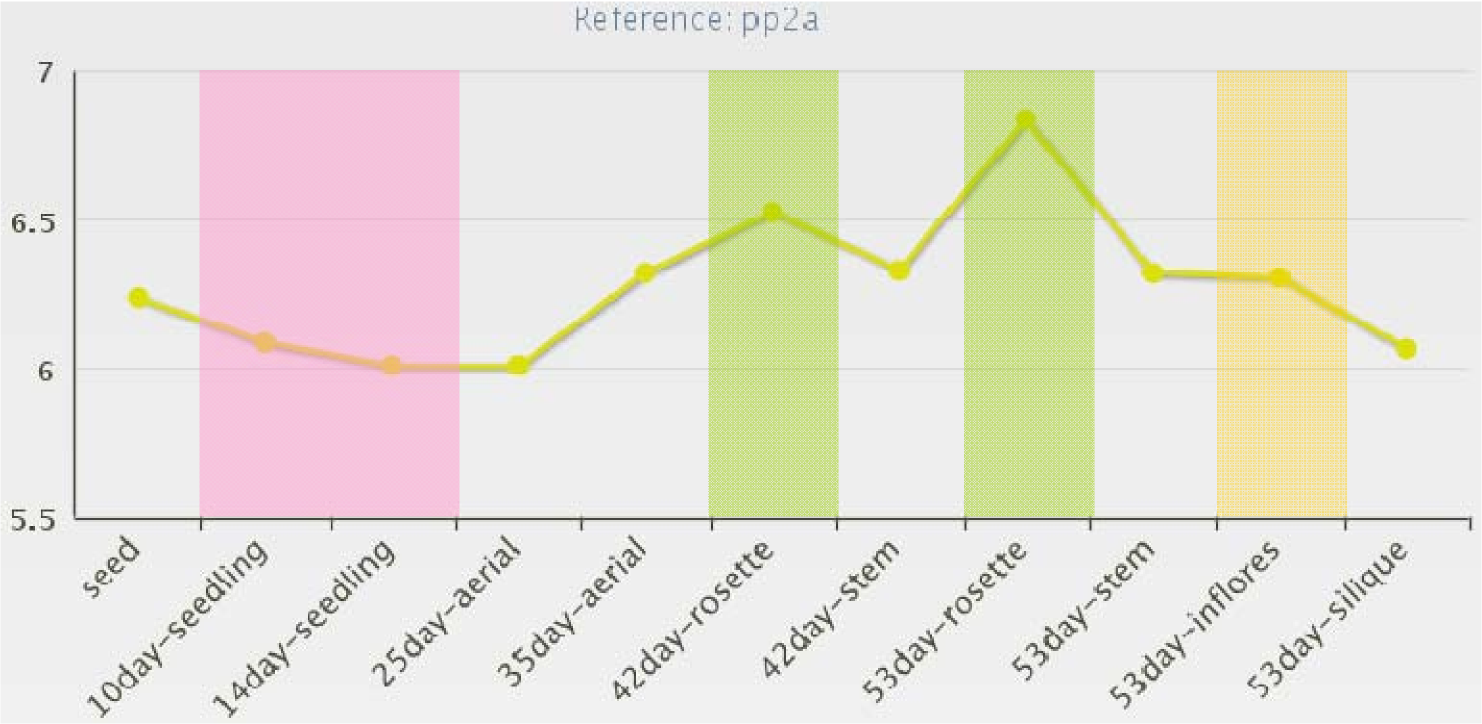

miRNA	WT_Flower	WT_Leaf	WT_Root	WT_Seedling
ath-miR168a	266.47	435.31	174.99	166.64

Pri-miRNA	Match site	Sequence	INF	INS	AP1	AP3	AGM	SAP	S04	S52	LES	LEF	ROS	ROF	GSE
ath-MIR168a	25	GATCTGGAAGATTTCTA	0	0	0	1	1	0	0	20	0	0	0	0	0
	25	GATCTGGAAGATTTCTACGC	0	0	0	2	0	0	0	22	0	0	0	0	0
	371	GATCGTTACAGCAATCT	0	0	0	3	0	0	0	0	1	15	0	0	0
	371	GATCGTTACAGCAATCTAAT	0	0	0	2	0	0	0	0	0	10	0	0	0
