# Supplementary material for: Expression-Based Functional Investigation of the Organ-Specific MicroRNAs in Arabidopsis
Source: PLoS One. 2012 Nov 30;7(11):e50870. doi: 10.1371/journal.pone.0050870 (PMC3511311; doi:10.1371/journal.pone.0050870)
Supplement: Figure S4 — List of the microRNA genes with consistent expression patterns between the pre-miRNAs/pri-miRNAs (based on the data provided by mirEX) and the pri-miRNAs (based on the MPSS data). For the mature miRNAs listed in the first tables, their detectable expression levels (normalized in RPM; reads per million) in flowers, leaves, roots and seedlings, based on the small RNA (sRNA) high-throughput sequencing (HTS) data, were highlighted in different background. The sRNA HTS data sets were retrieved from GEO (Gene Expression Omnibus; http://www.ncbi.nlm.nih.gov/geo/) [68]: WT_Flower, GSM707678; WT_Leaf, GSM707679; WT_Root, GSM707680; WT_Seedling, GSM707681. For the pri-miRNAs listed in the second tables, the detectable levels of the identified poly(A) signals based on MPSS (massively parallel signature sequencing) data were highlighted by the same background colors as above according to the organs analyzed. The MPSS data sets were retrieved from Next-Gen Sequence Databases (http://mpss.udel.edu/at/mpss_index.php) [18]. The libraries INF, INS, AP1, AP3, AGM and SAP were prepared from Arabidopsis flowers (indicated by yellow background). S04, S52, LES and LEF were prepared from leaves (green background). ROS and ROF were prepared from roots (gray), and GSE from young seedlings (red). The expression levels of the pre-miRNAs detected by real-time PCR [PP2A (phosphatase 2A; AT1G13320) as the reference gene] in the similar organs retrieved from mirEX (http://comgen.pl/mirex/) [17] were also highlighted in different background as indicated above. Please note: the y axis is in log scale. (PDF) [file pone.0050870.s004.pdf]

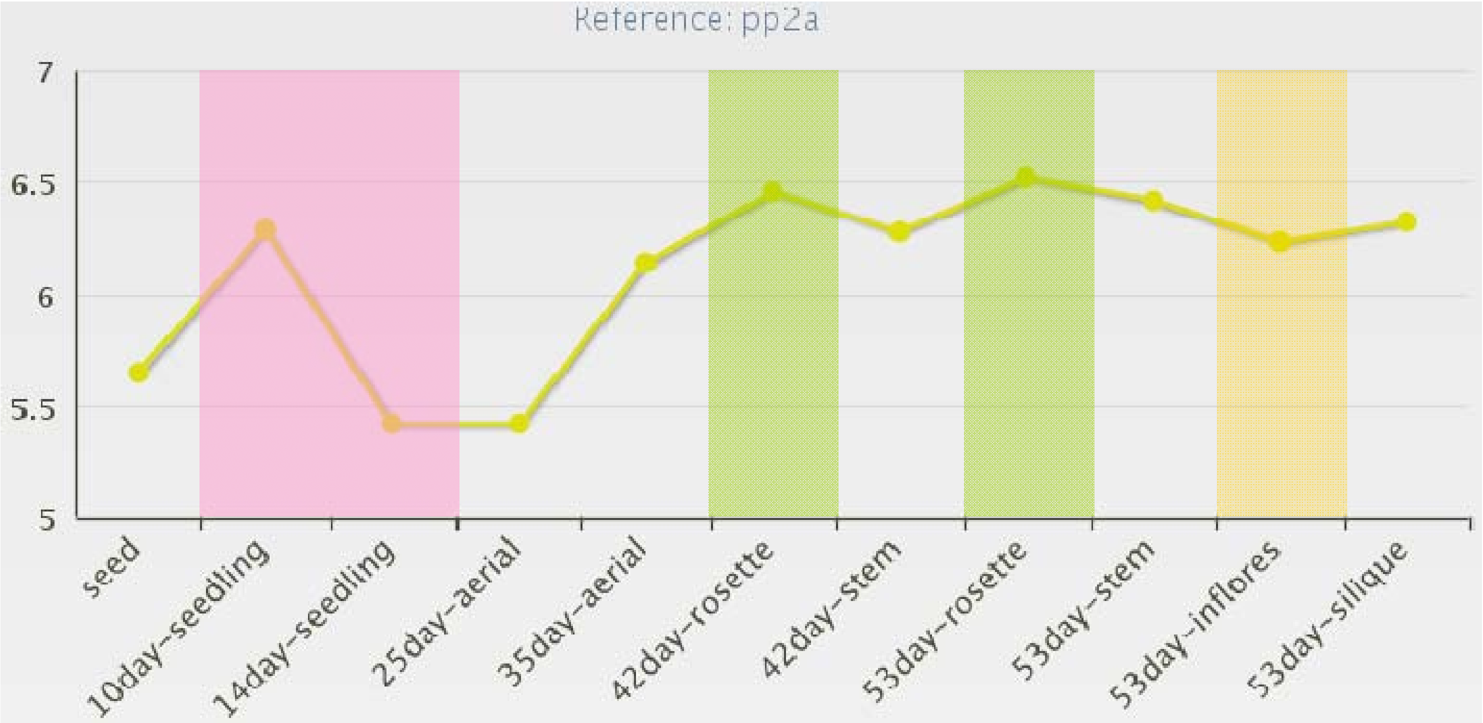

miRNA	WT_Flower	WT_Leaf	WT_Root	WT_Seedling
ath-miR166a	3261.89	2154.13	1228.68	1448.18

Pri-miRNA	Match site	Sequence	INF	INS	AP1	AP3	AGM	SAP	S04	S52	LES	LEF	ROS	ROF	GSE
ath-MIR166a	345	GATCTTCATGGTATTGA	5	0	0	0	0	0	0	0	23	0	0	1	5
	345	GATCTTCATGGTATTGAATG	6	0	0	0	0	0	0	0	23	0	0	1	7

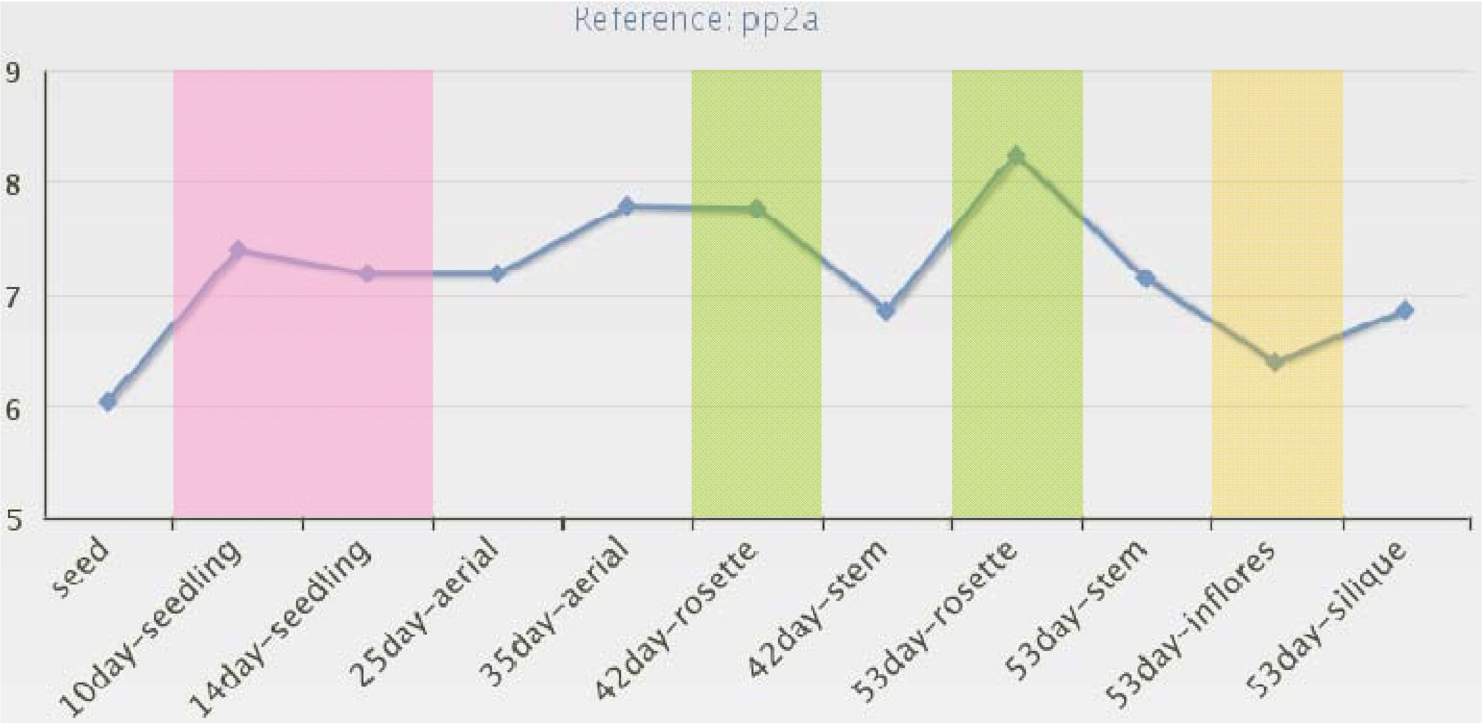

miRNA	WT_Flower	WT_Leaf	WT_Root	WT_Seedling
ath-miR398b	0.81	0.2	0	40.69

Pri-miRNA	Match site	Sequence	INF	INS	AP1	AP3	AGM	SAP	S04	S52	LES	LEF	ROS	ROF	GSE
ath-MIR398b	181	GATCTACTTATTGTCAT	0	0	0	1	0	0	15	8	37	41	14	37	0
	181	GATCTACTTATTGTCATGCT	0	0	0	1	0	0	15	9	39	47	16	43	0

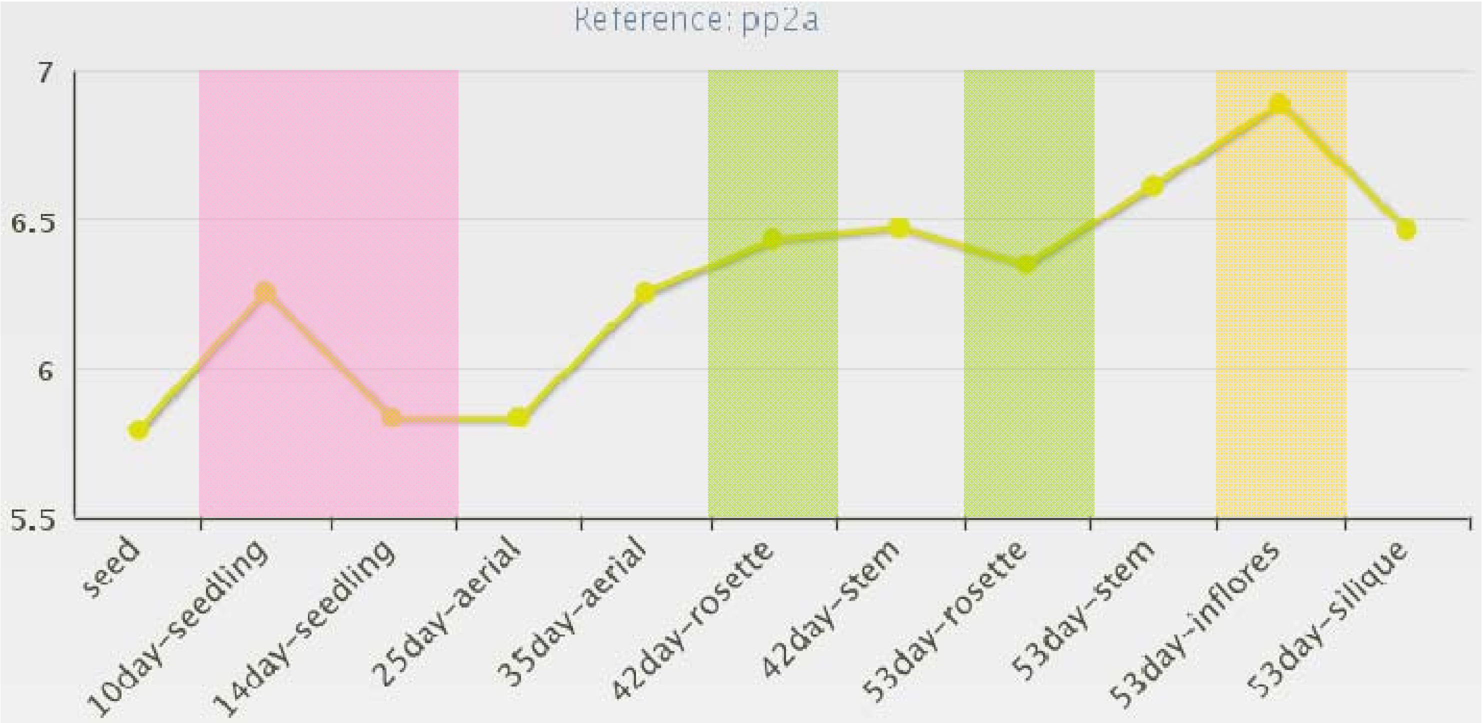

miRNA	WT_Flower	WT_Leaf	WT_Root	WT_Seedling
ath-miR775	127.9	124.83	108.22	57.52

Pri-miRNA	Match site	Sequence	INF	INS	AP1	AP3	AGM	SAP	S04	S52	LES	LEF	ROS	ROF	GSE
ath-MIR775	222	GATCTTCAAAACGATGA	2	3	2	0	10	3	0	0	0	0	0	0	0
	222	GATCTTCAAAACGATGACTA	2	3	2	0	12	3	0	0	0	0	0	0	0
