# Supplementary material for: Expression-Based Functional Investigation of the Organ-Specific MicroRNAs in Arabidopsis
Source: PLoS One. 2012 Nov 30;7(11):e50870. doi: 10.1371/journal.pone.0050870 (PMC3511311; doi:10.1371/journal.pone.0050870)
Supplement: Figure S5 — Expression of the microRNA clusters. The genomic positions of the pre-miRNAs (precursor microRNAs) according to miRBase (release 17) [67] were listed in the first tables. For the mature miRNAs listed in the second tables, their detectable expression levels (normalized in RPM; reads per million) in flowers, leaves, roots and seedlings, based on the small RNA (sRNA) high-throughput sequencing (HTS) data, were highlighted in different background. The sRNA HTS data sets were retrieved from GEO (Gene Expression Omnibus; http://www.ncbi.nlm.nih.gov/geo/) [68]: WT_Flower, GSM707678; WT_Leaf, GSM707679; WT_Root, GSM707680; WT_Seedling, GSM707681. The expression levels of the pre-miRNAs detected by real-time PCR [PP2A (phosphatase 2A; AT1G13320) or actin (AT3G18780) as the reference gene] in the similar organs retrieved from mirEX (http://comgen.pl/mirex/) [17] were also highlighted in different background as above. Please note: the y axis is in log scale. (PDF) [file pone.0050870.s005.pdf]

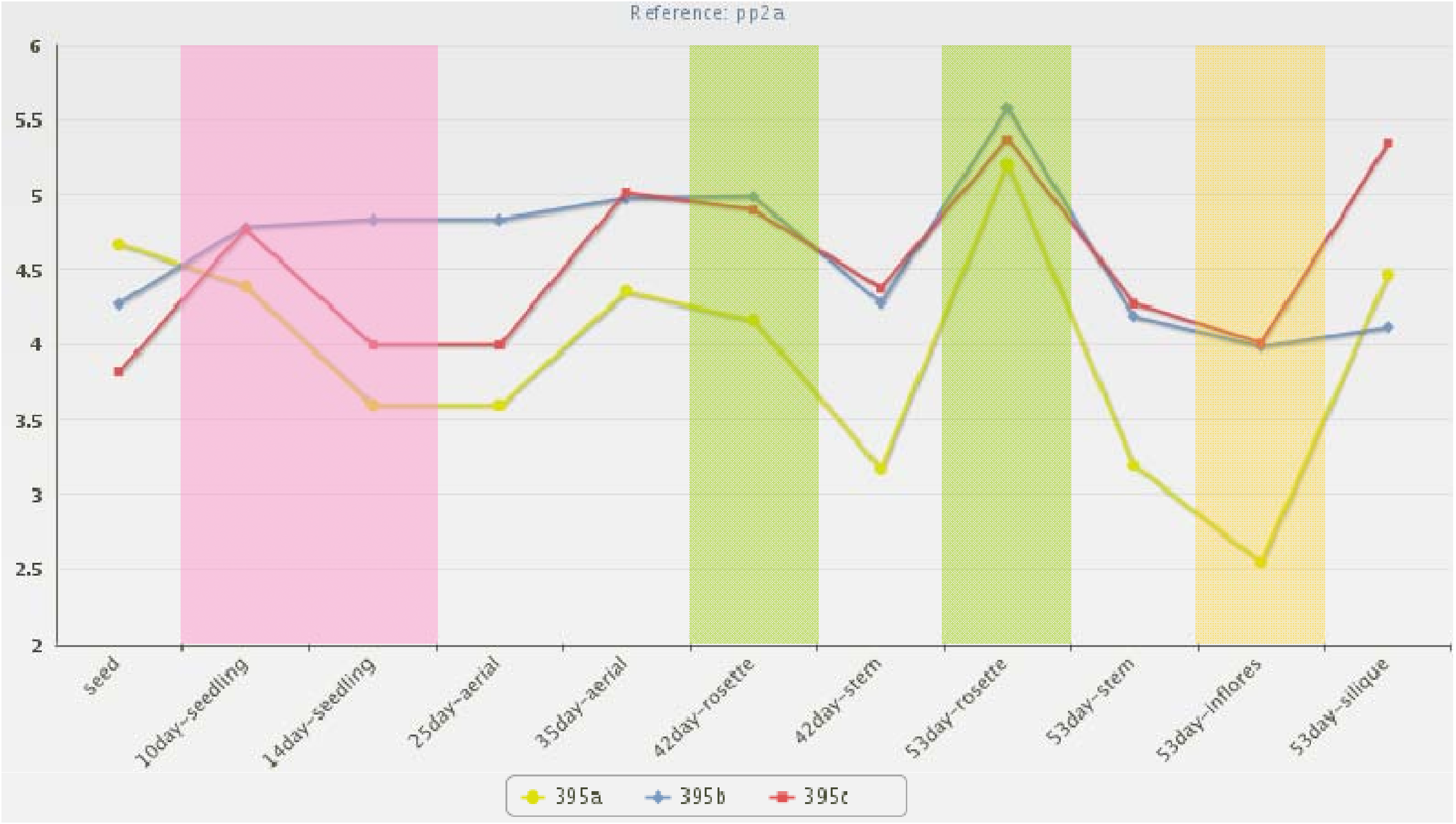

Pre-miRNA	Chr	Strand	Start	End	Distance
ath-MIR395a	1	-	9363196	9363288	
ath-MIR395b	1	+	9364471	9364570	1183
ath-MIR395c	1	+	9367080	9367179	2510

miRNA	WT_Flower	WT_Leaf	WT_Root	WT_Seedling
ath-miR395a	9.06	19	33.39	19.6
ath-miR395b	4.63	20	60.44	10.73
ath-miR395c	4.63	20	60.44	10.73

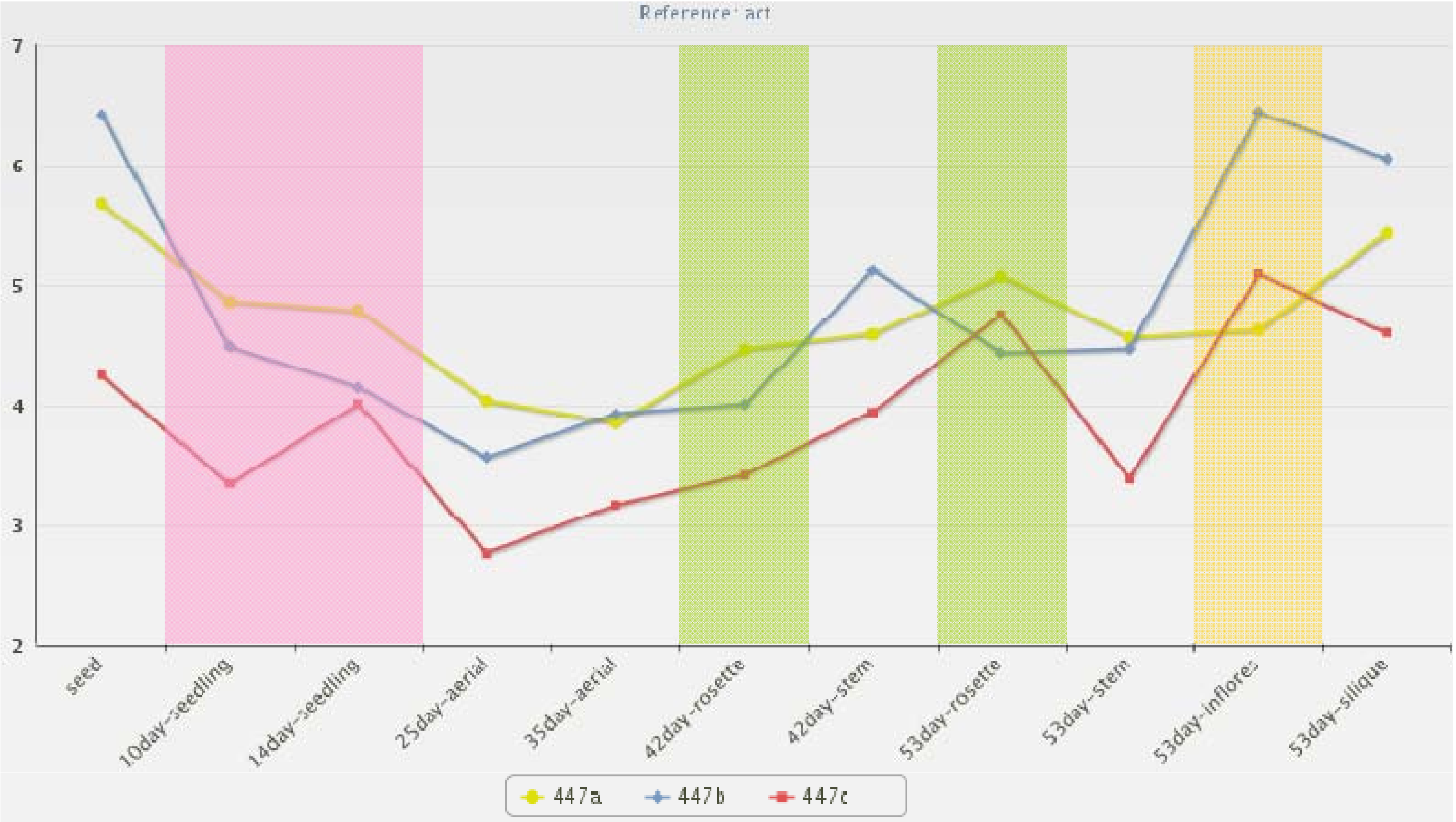

Pre-miRNA	Chr	Strand	Start	End	Distance
ath-MIR447c	4	-	1523306	1523503	
ath-MIR447a	4	-	1528134	1528370	4631
ath-MIR447b	4	-	1535426	1535661	7056

miRNA	WT_Flower	WT_Leaf	WT_Root	WT_Seedling
ath-miR447a	24.37	9.4	3.17	5.73
ath-miR447a.2	666.07	218.45	96.13	102.46
ath-miR447b	24.37	9.4	3.17	5.73
ath-miR447c-3p	0	0	0	0
ath-miR447c-5p	1.01	0.2	0	0

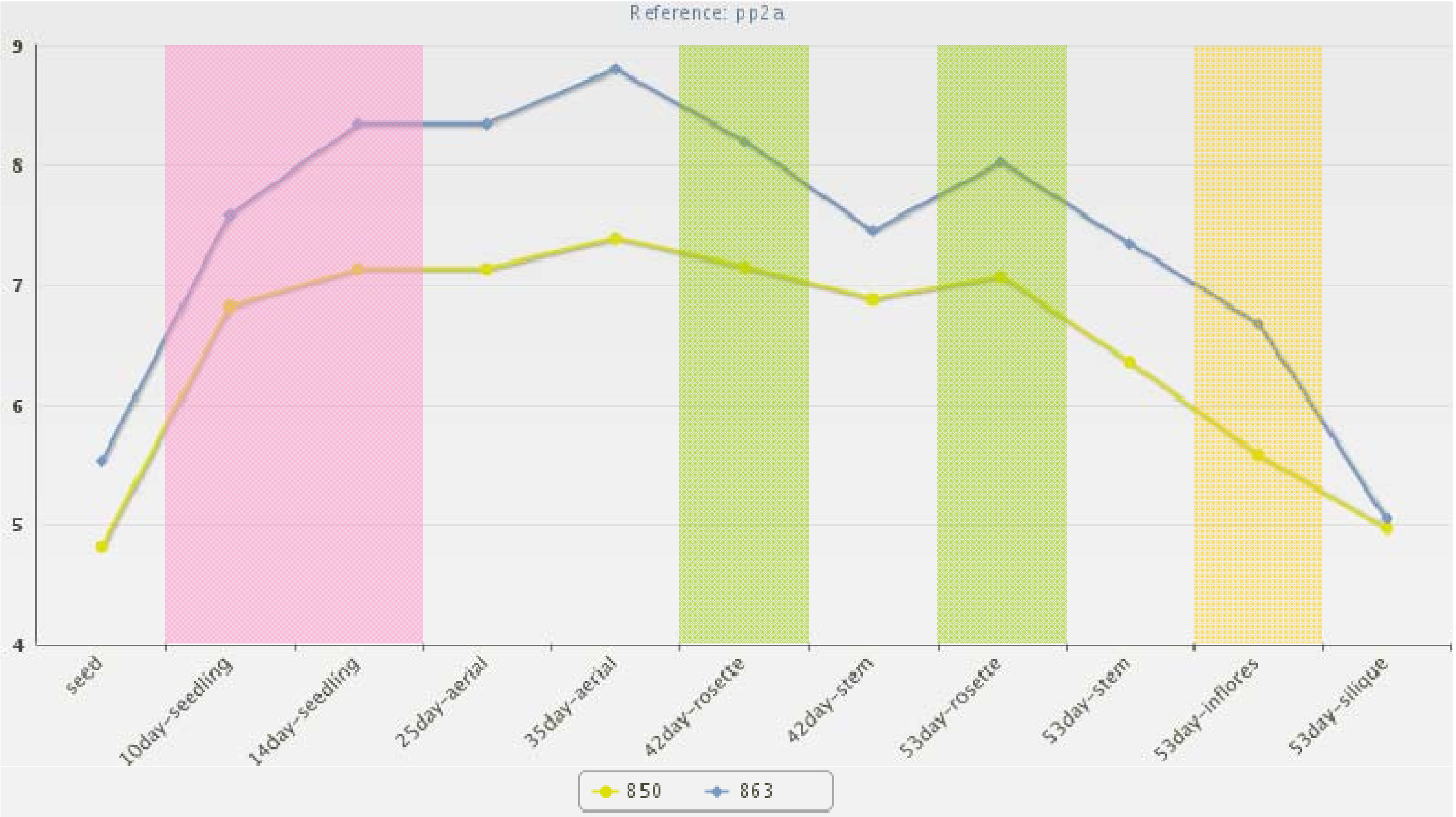

Pre-miRNA	Chr	Strand	Start	End	Distance
ath-MIR5026	4	+	7844496	7844688	
ath-MIR850	4	+	7845707	7845927	1019
ath-MIR863	4	+	7846597	7846899	670

miRNA	WT_Flower	WT_Leaf	WT_Root	WT_Seedling
ath-miR850	0.4	3	0	1.48
ath-miR863-3p	1.21	27.21	0.58	25.15
ath-miR863-5p	0	2.4	0	1.48
ath-miR5026	20.14	317.88	5.47	629.02

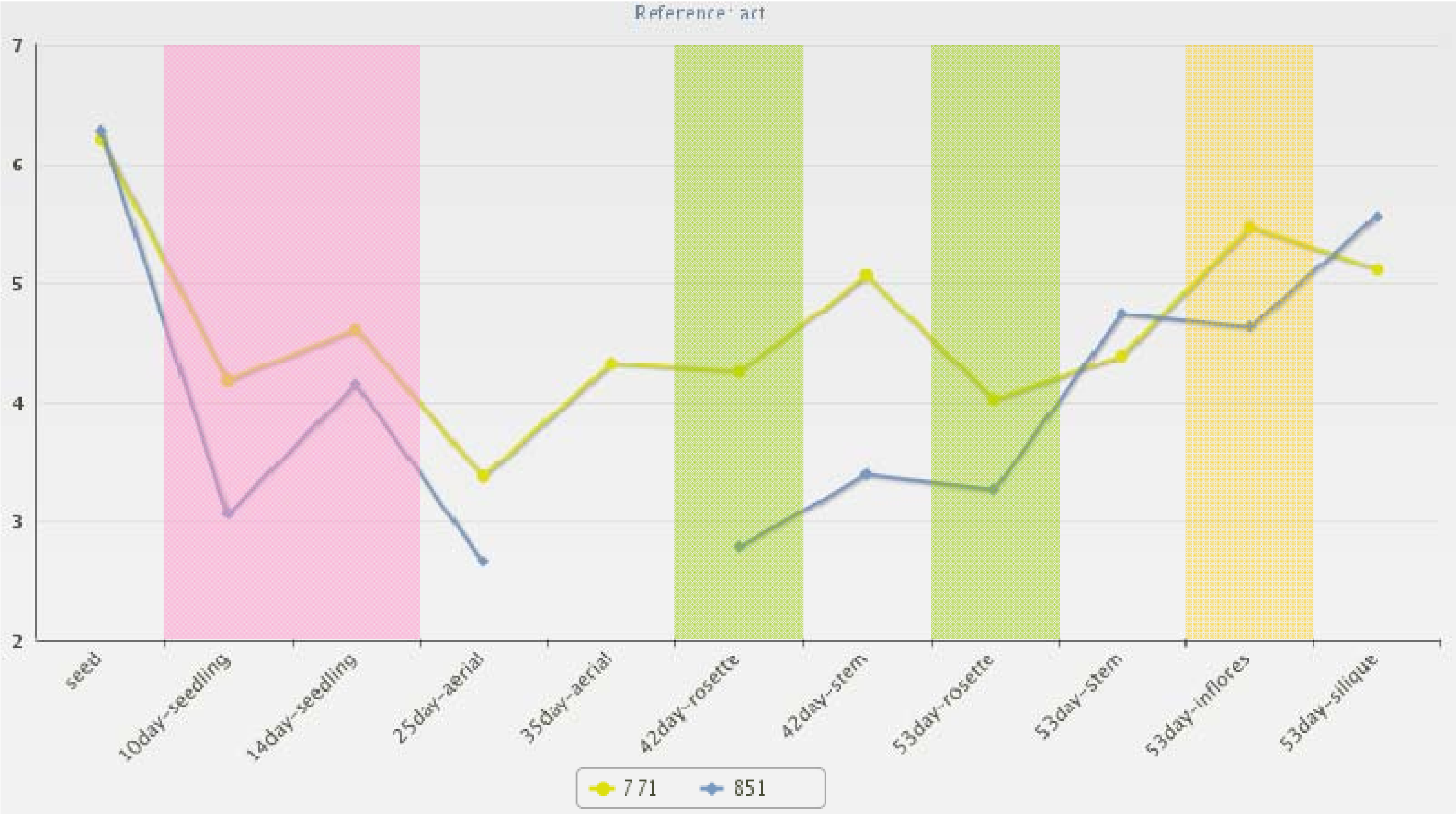

Pre-miRNA	Chr	Strand	Start	End	Distance
ath-MIR771	3	-	19659413	19659301	
ath-MIR851	3	-	19659715	19659532	414

miRNA	WT_Flower	WT_Leaf	WT_Root	WT_Seedling
ath-miR771	19.94	0.4	0	0
ath-miR851-5p	20.14	1	0	0.18
ath-miR851-3p	3.02	0	0	0

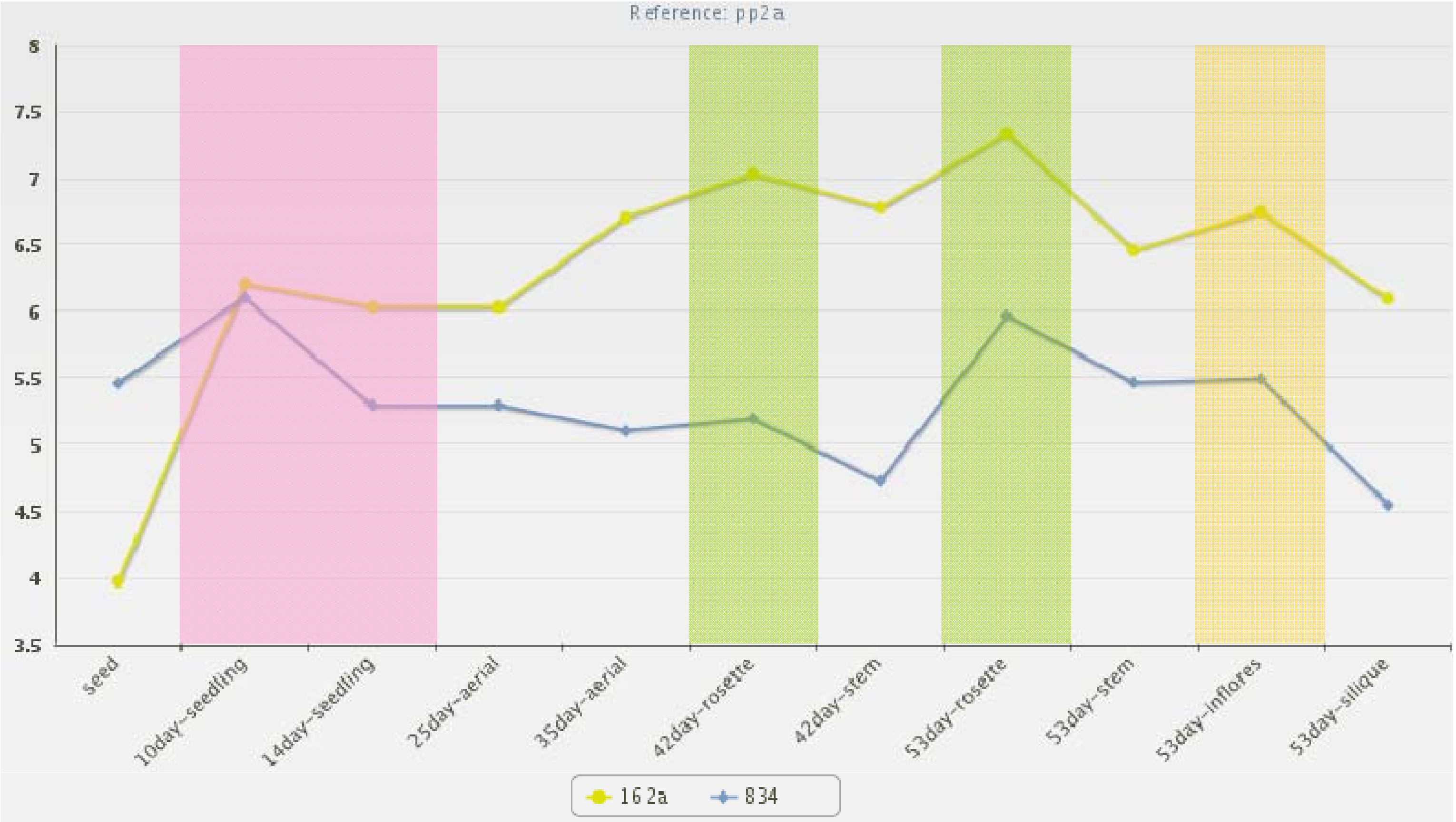

Pre-miRNA	Chr	Strand	Start	End	Distance
ath-MIR162a	5	-	2634905	2635044	
ath-MIR834	5	-	2641426	2641674	6382

miRNA	WT_Flower	WT_Leaf	WT_Root	WT_Seedling
ath-miR162a	373.62	389.9	219.89	136.5
ath-miR834	0	0	0	0

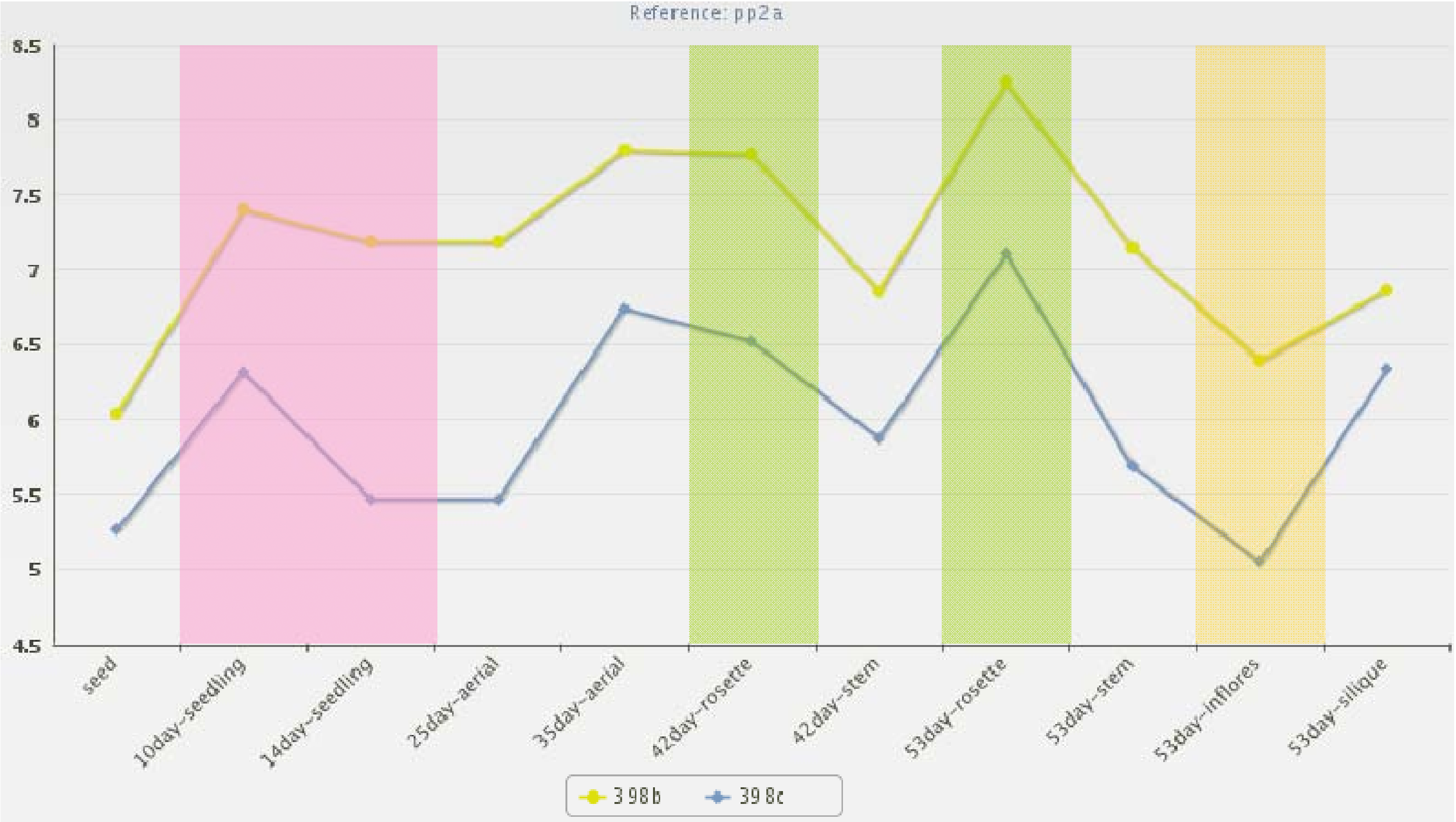

Pre-miRNA	Chr	Strand	Start	End	Distance
ath-MIR398b	5	+	4691022	4691137	
ath-MIR398c	5	+	4694694	4694808	3557

miRNA	WT_Flower	WT_Leaf	WT_Root	WT_Seedling
ath-miR398b	0.81	0.2	0	40.69
ath-miR398c	0.81	0.2	0	40.69
