# Supplementary figures and images for: Expression-Based Functional Investigation of the Organ-Specific MicroRNAs in Arabidopsis
Source: PLoS One. 2012 Nov 30;7(11):e50870. doi: 10.1371/journal.pone.0050870 (PMC3511311; doi:10.1371/journal.pone.0050870)

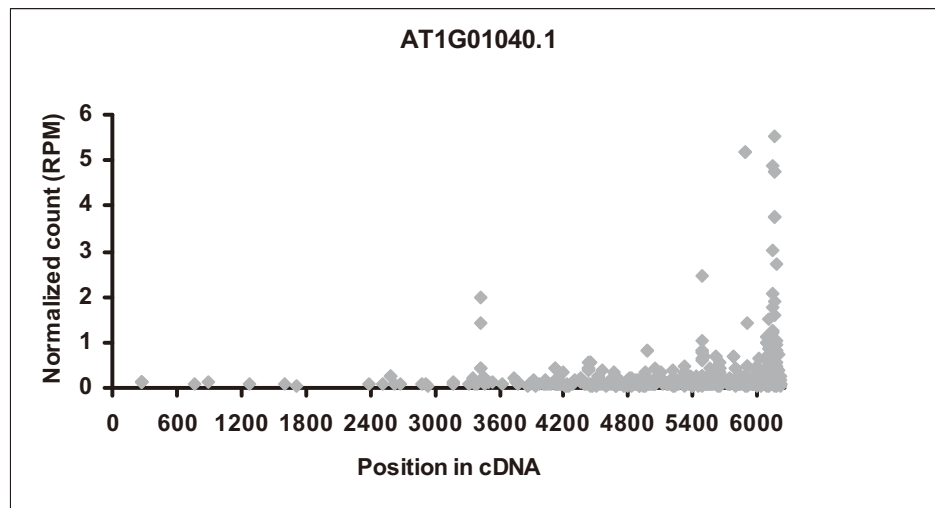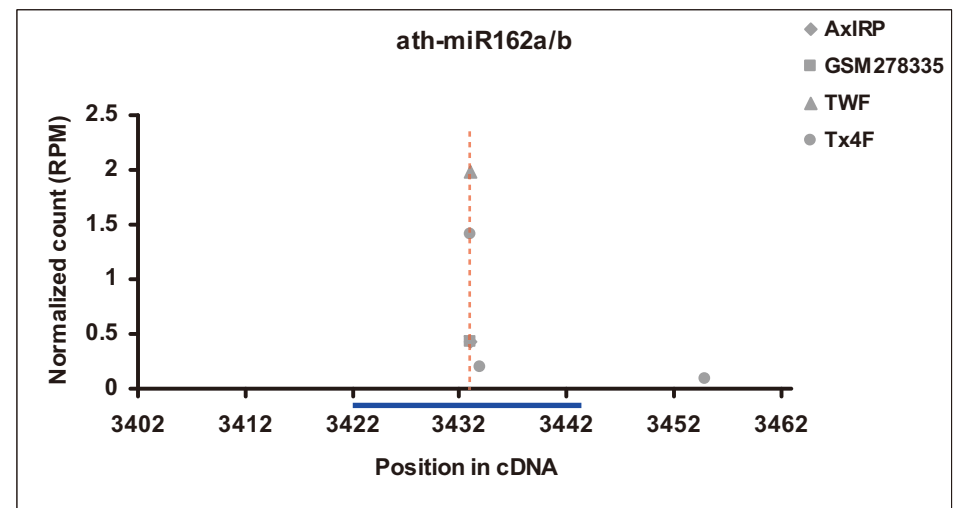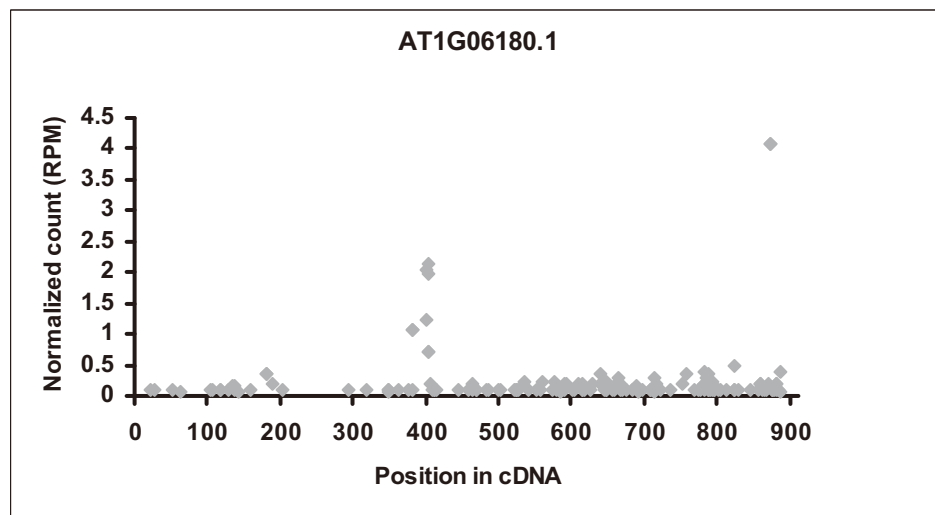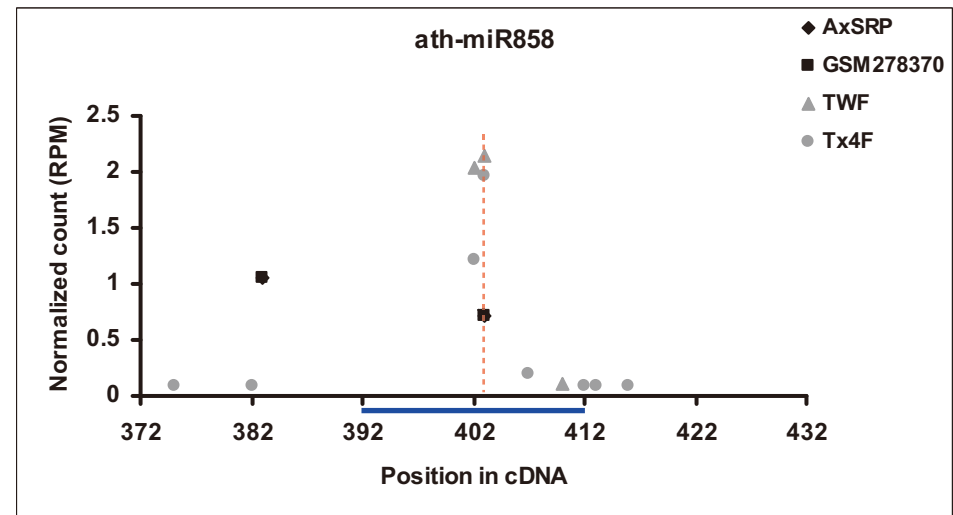

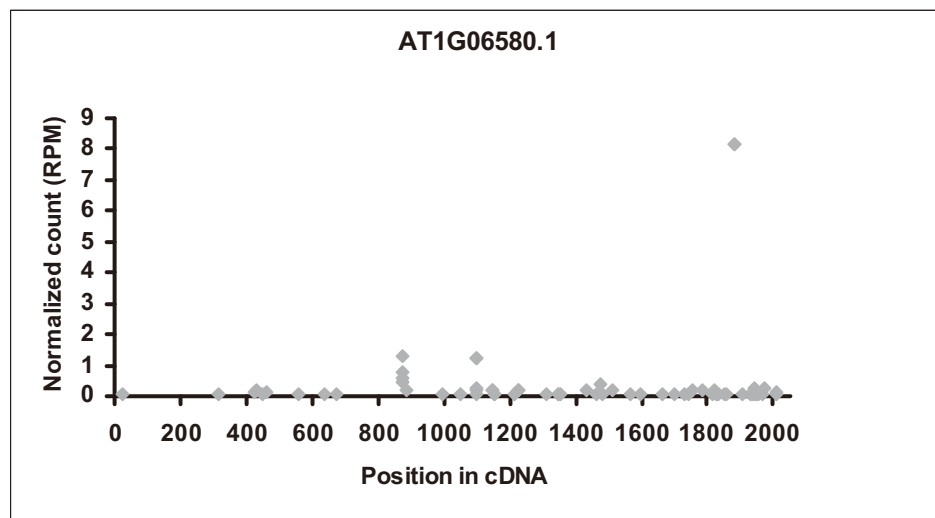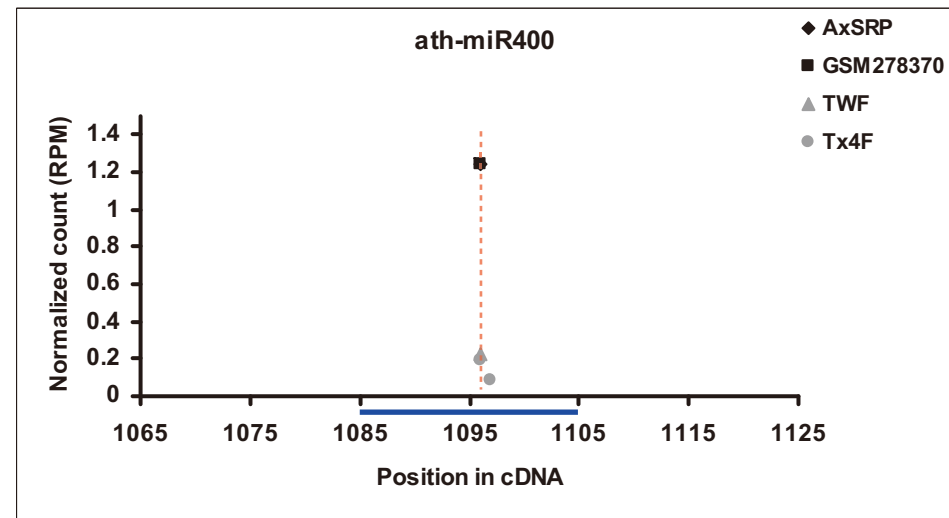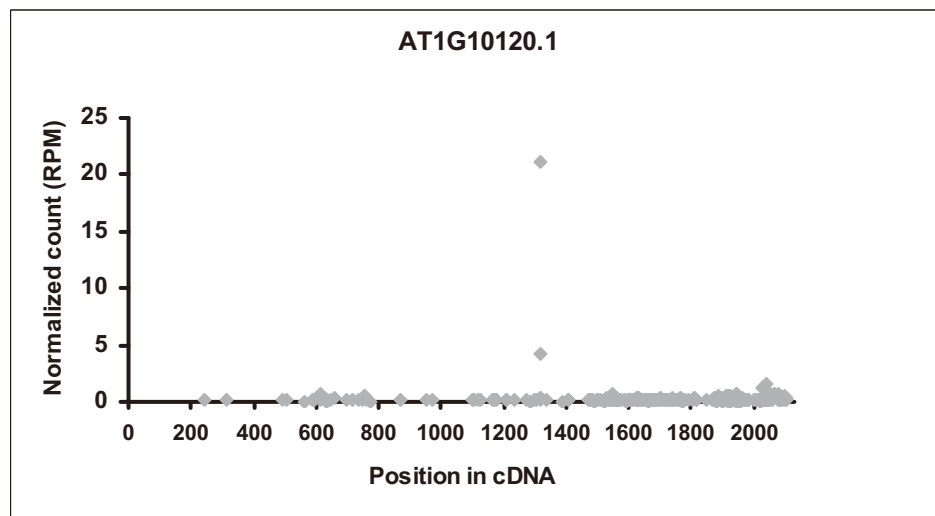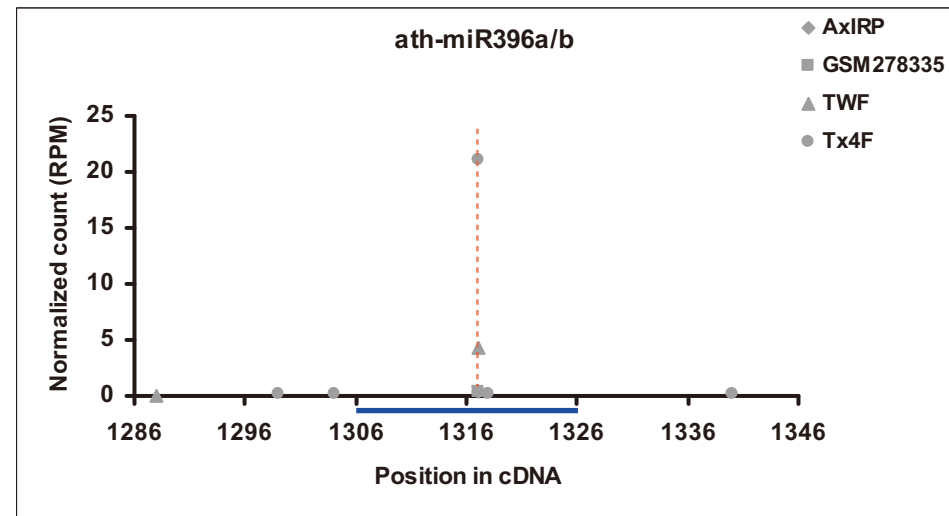

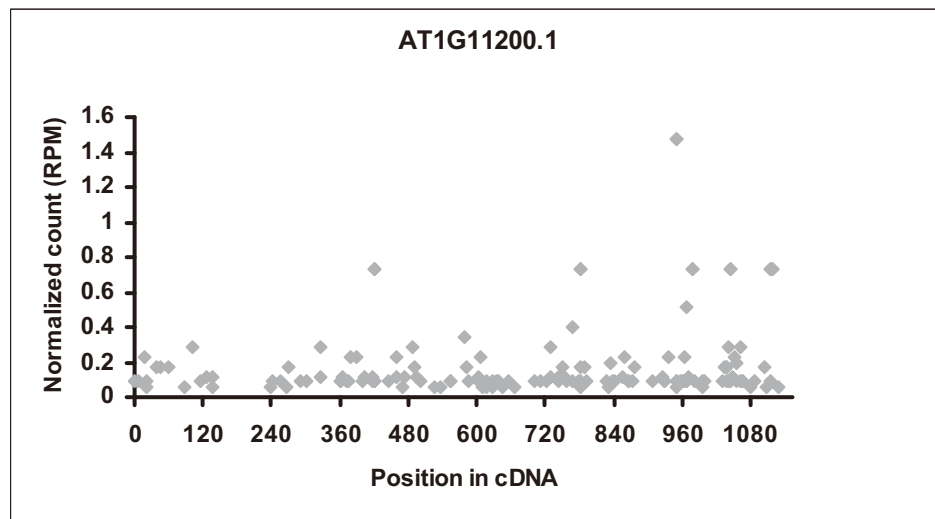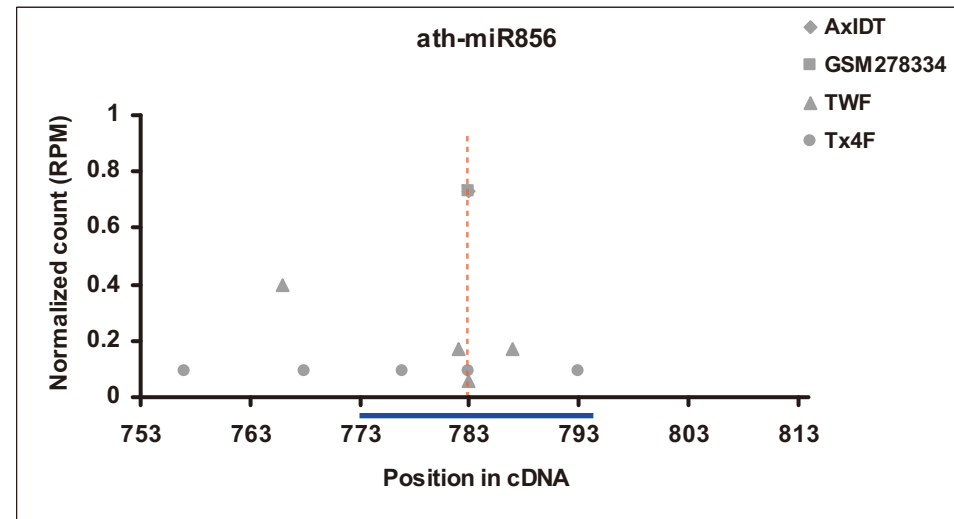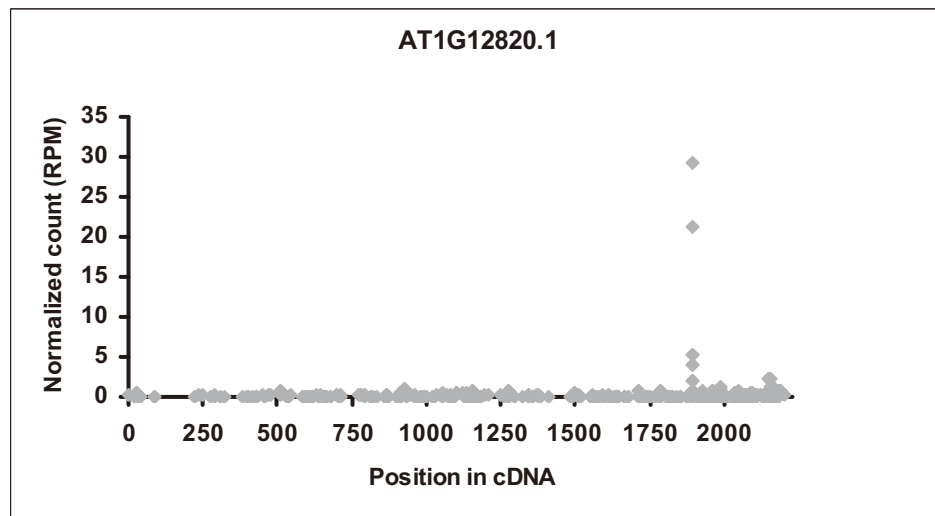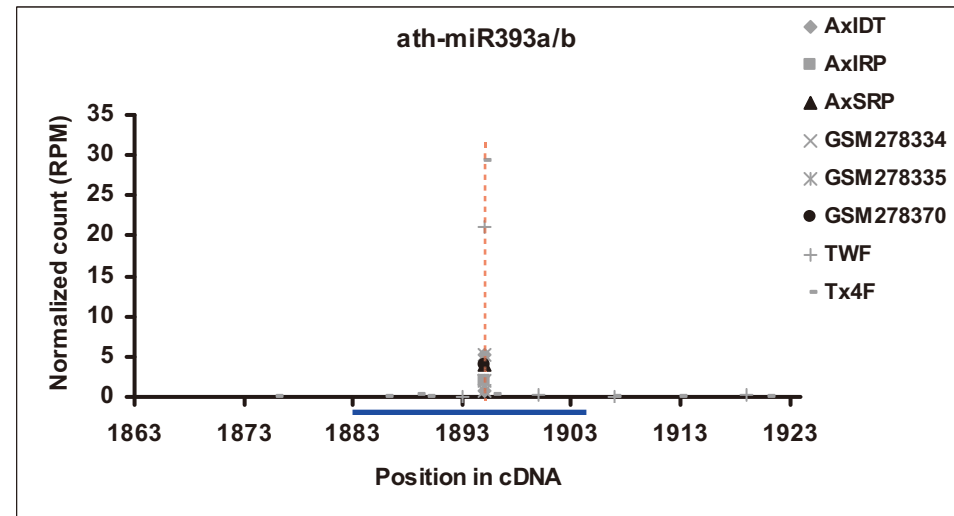

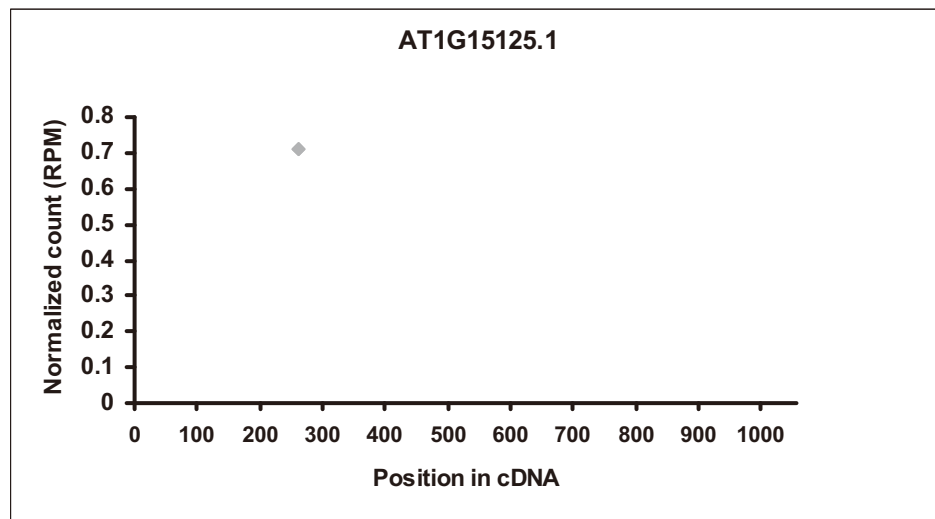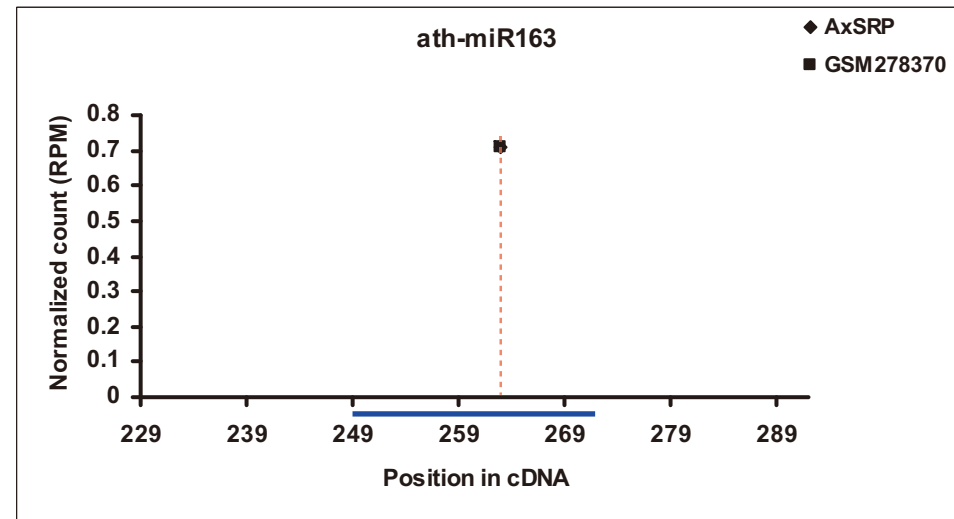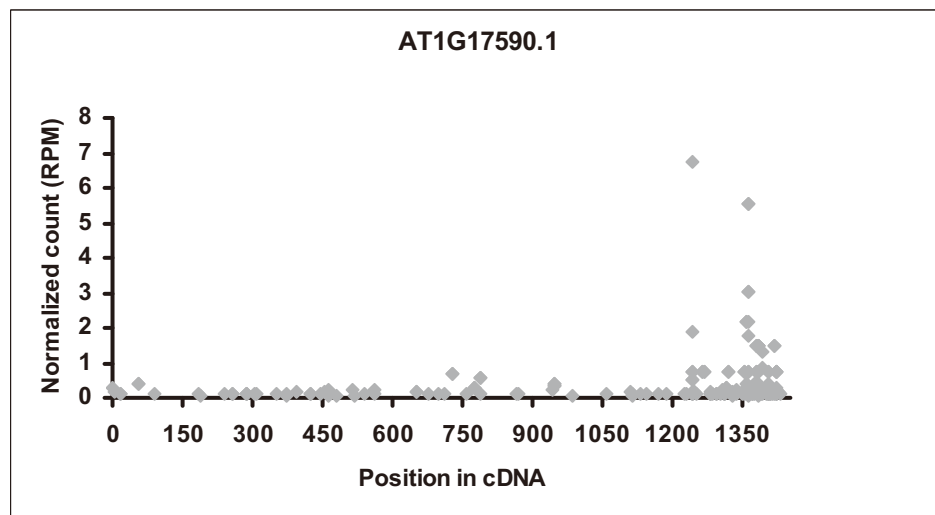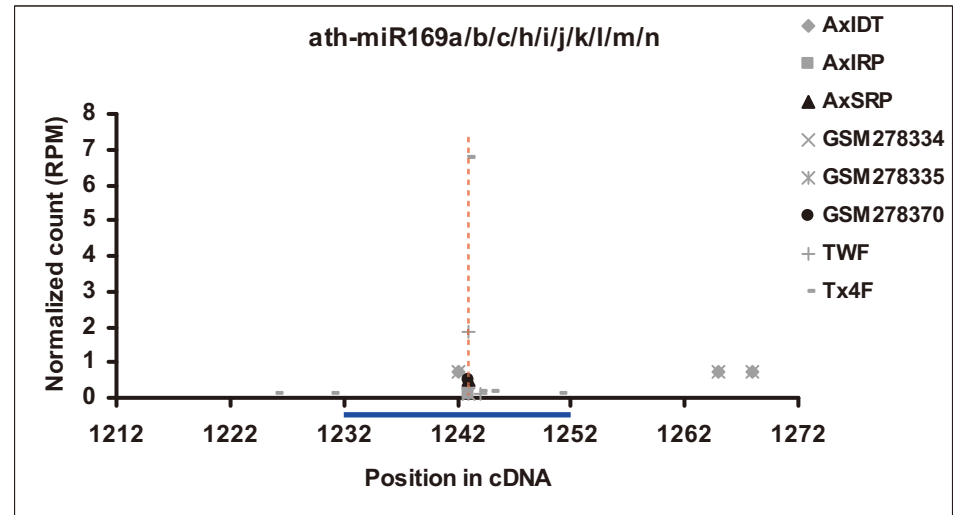

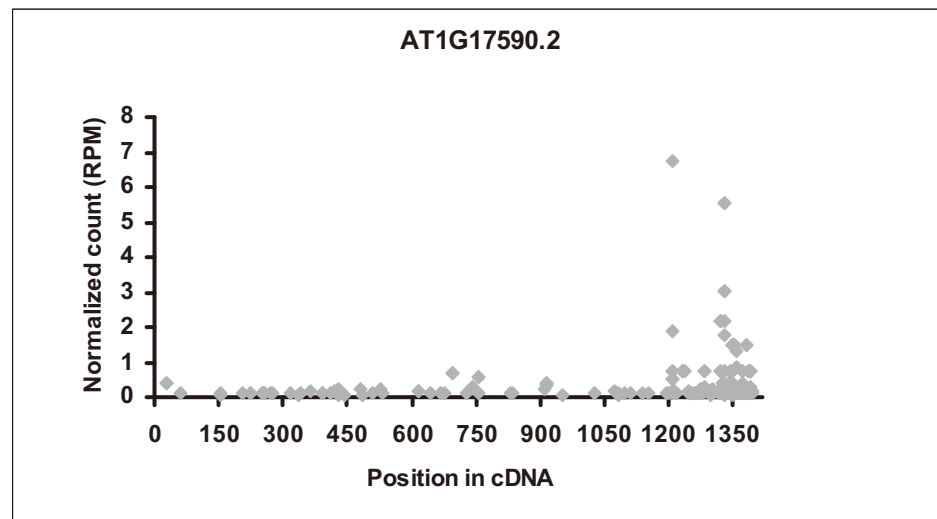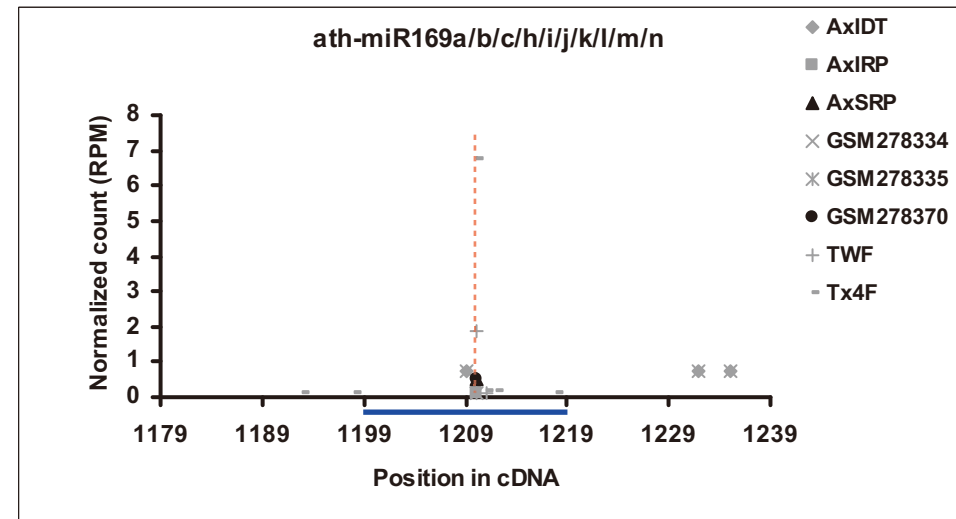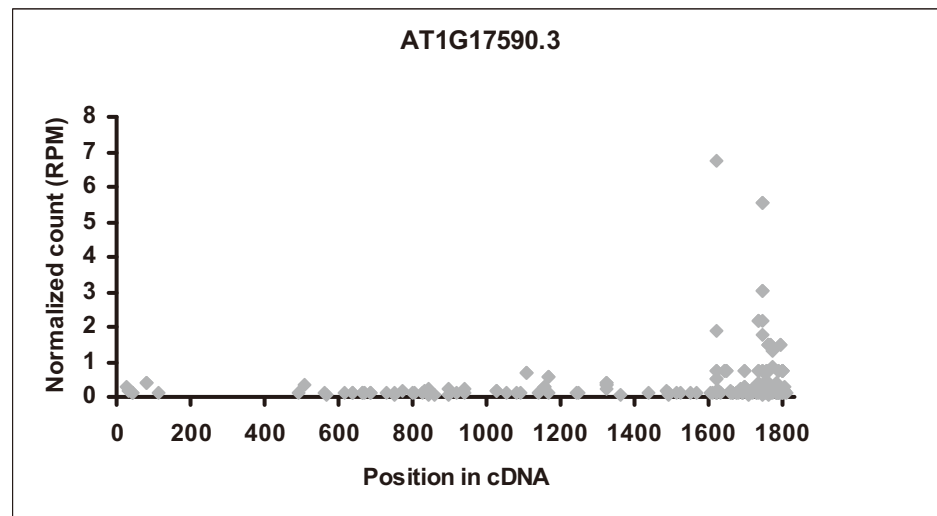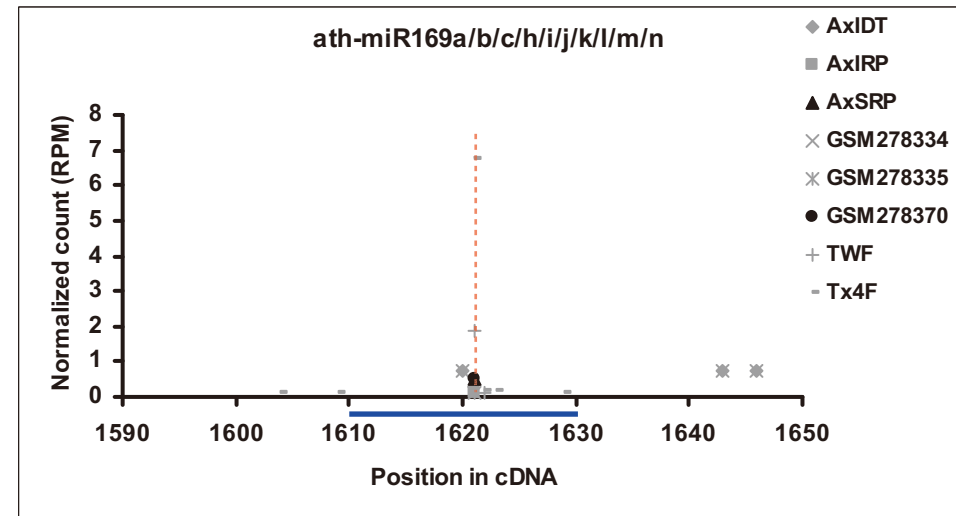

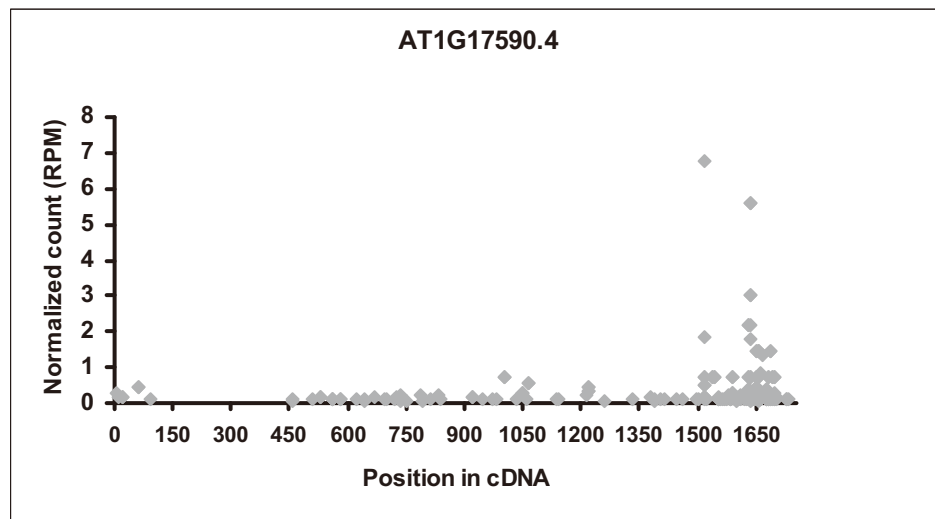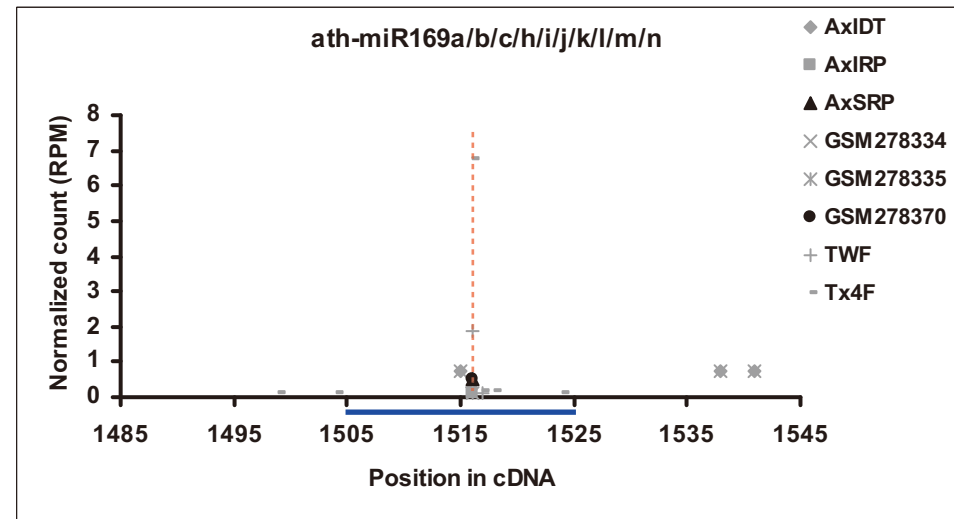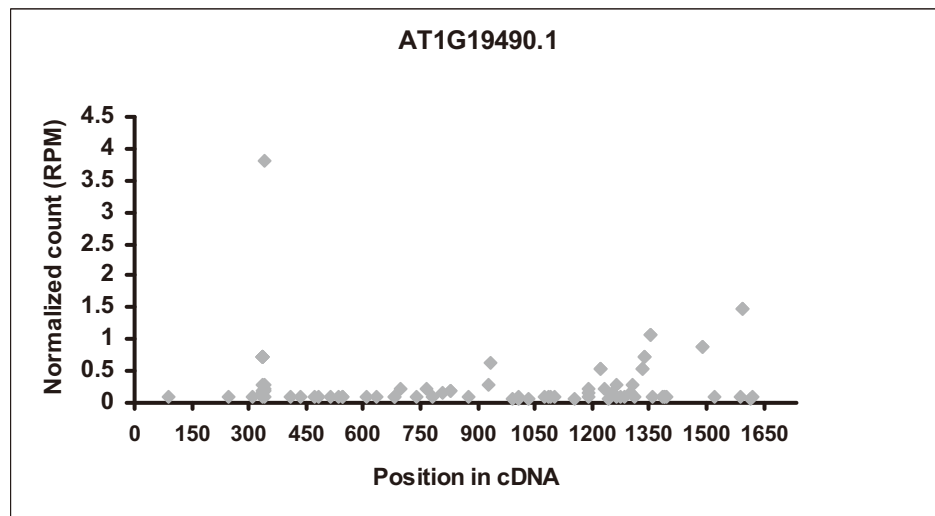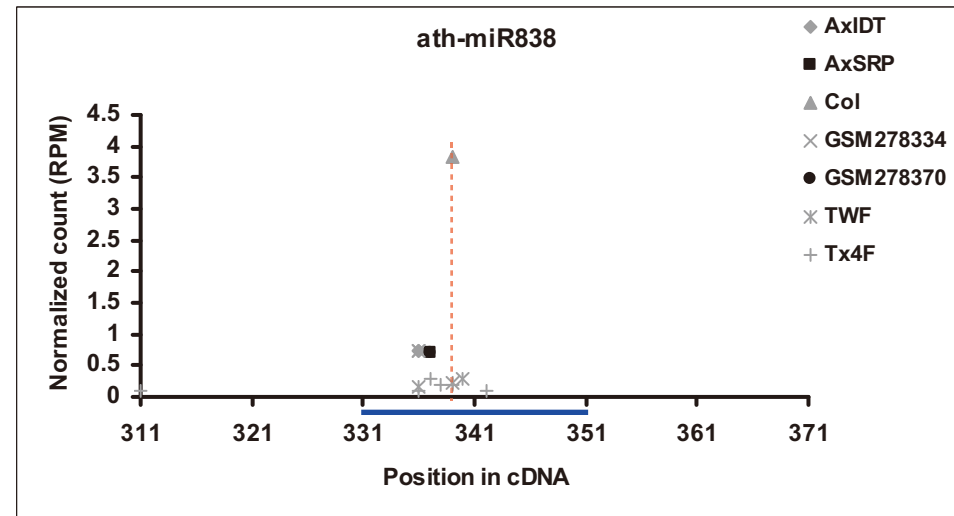

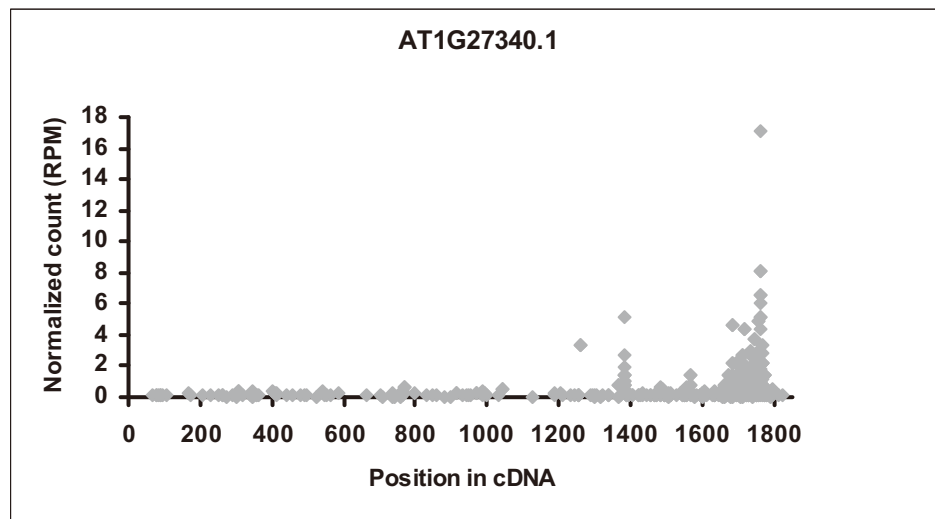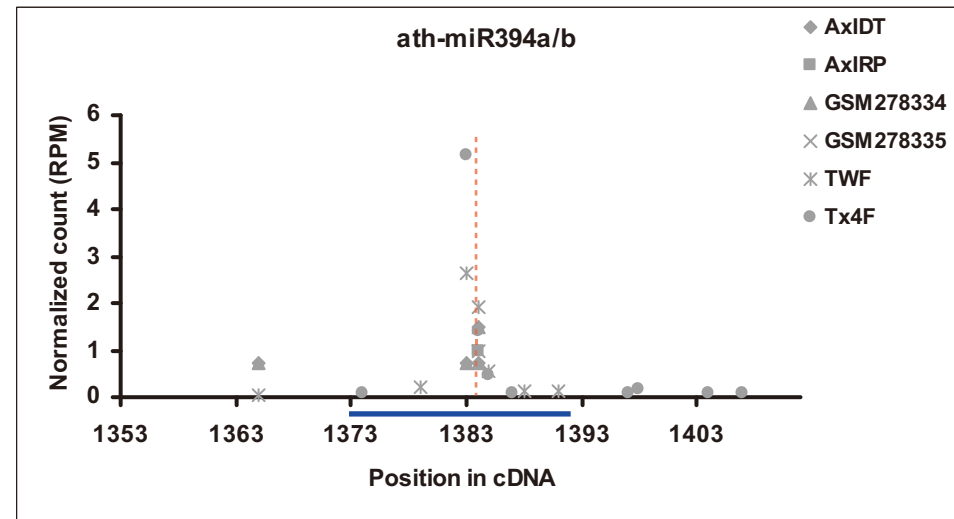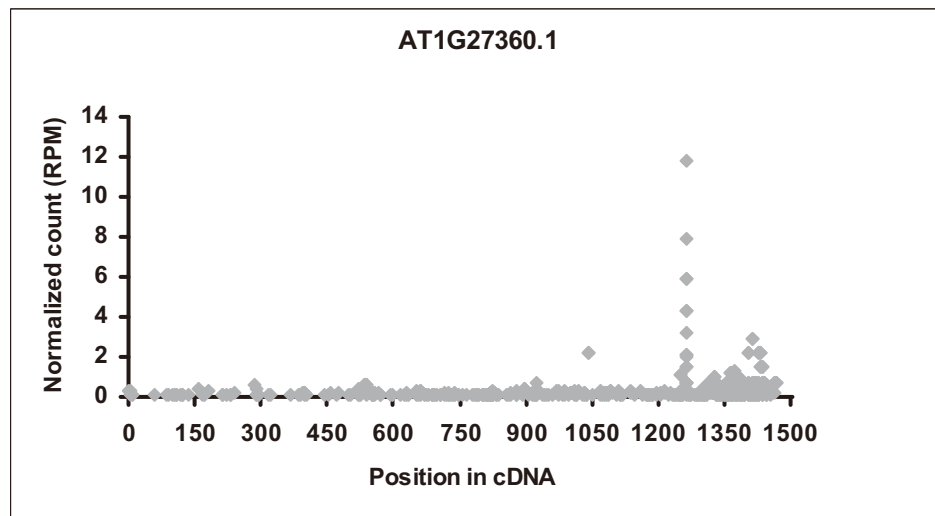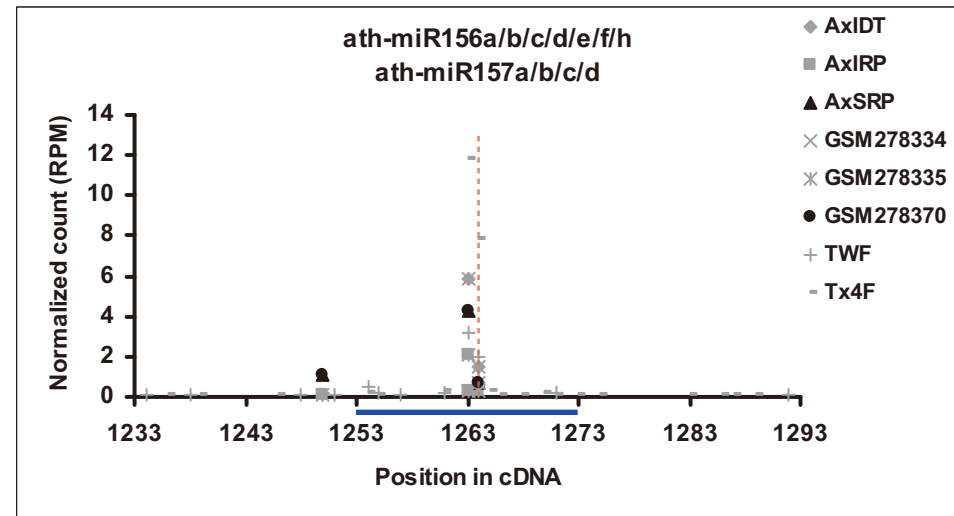

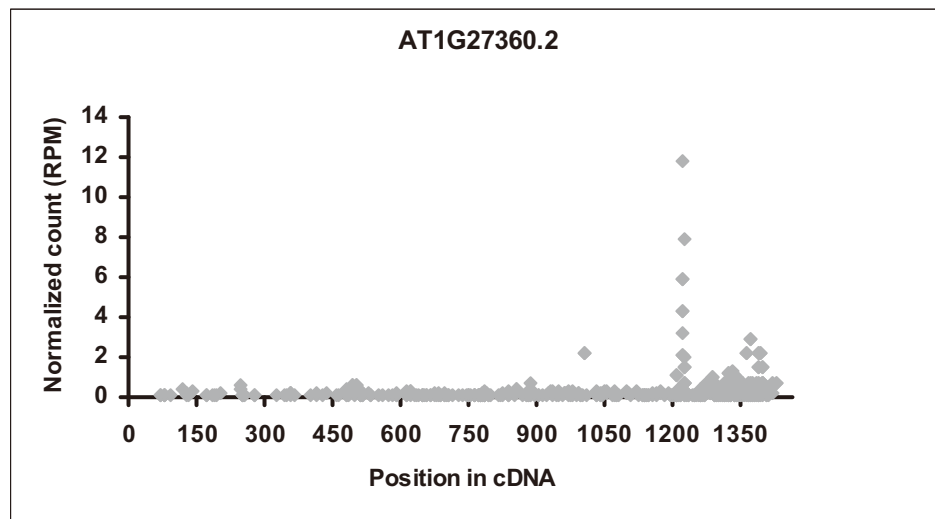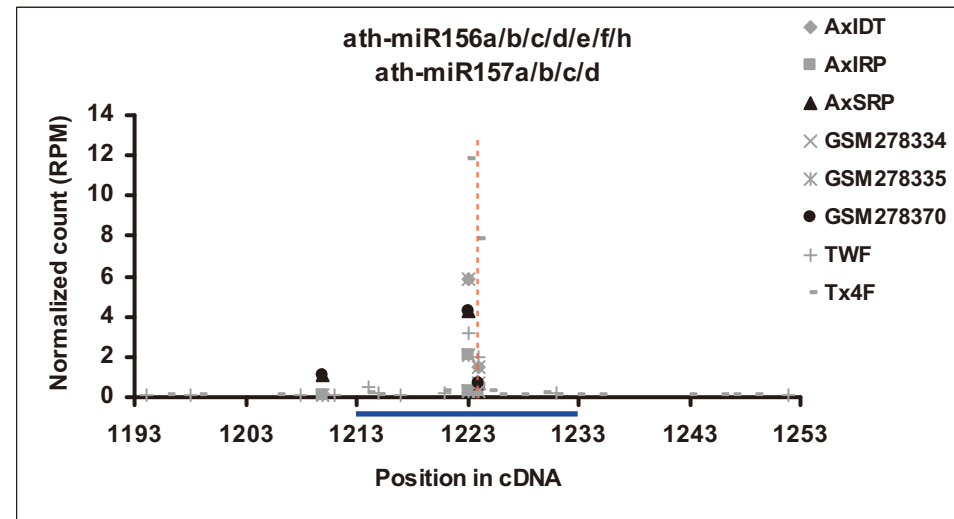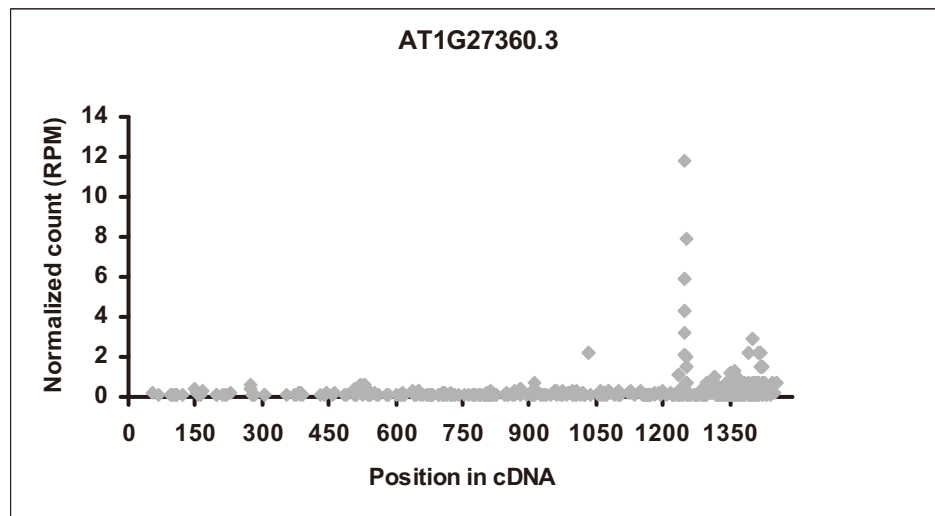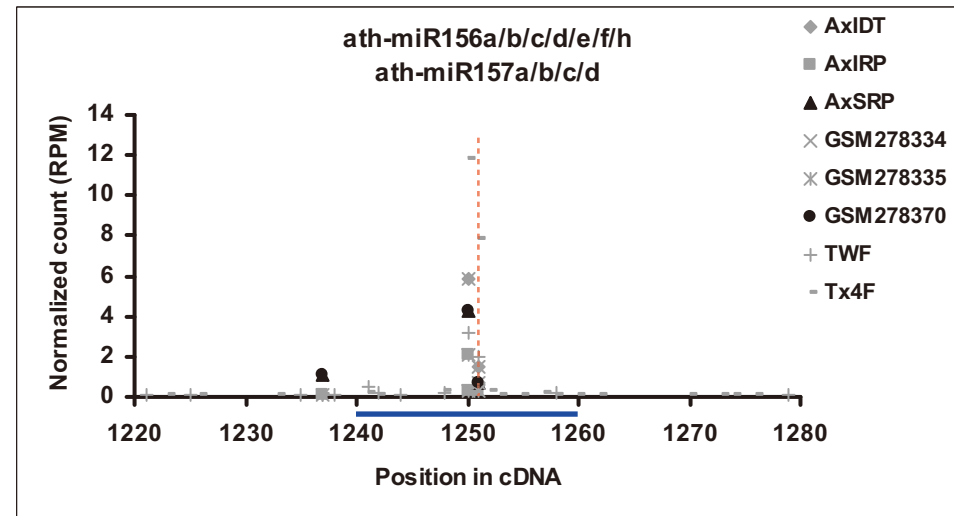

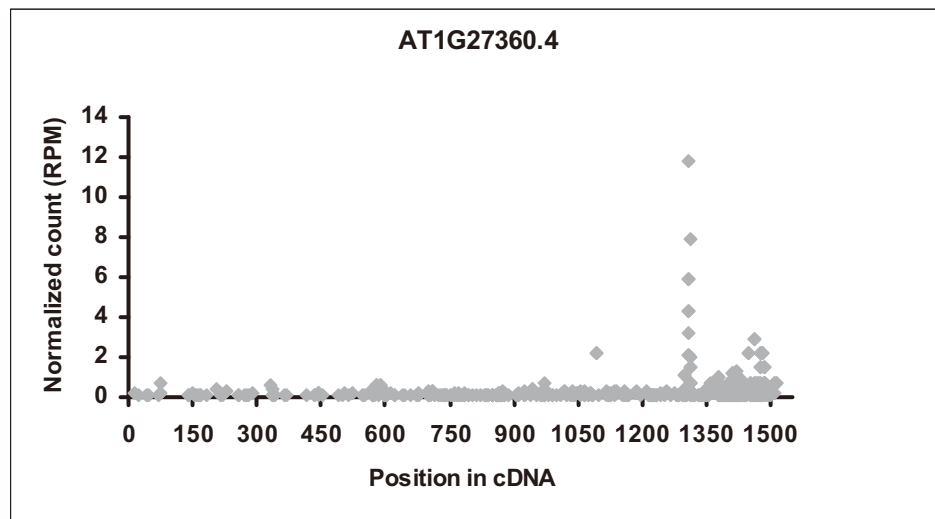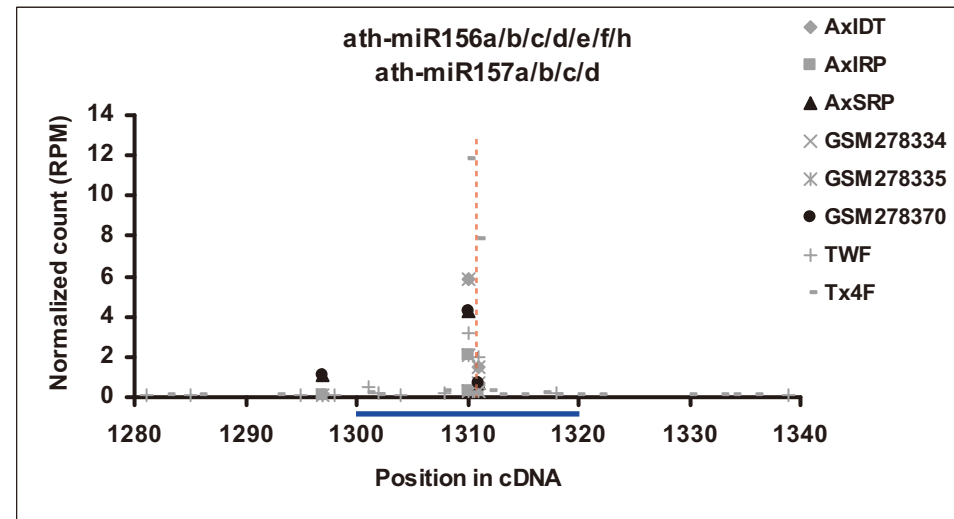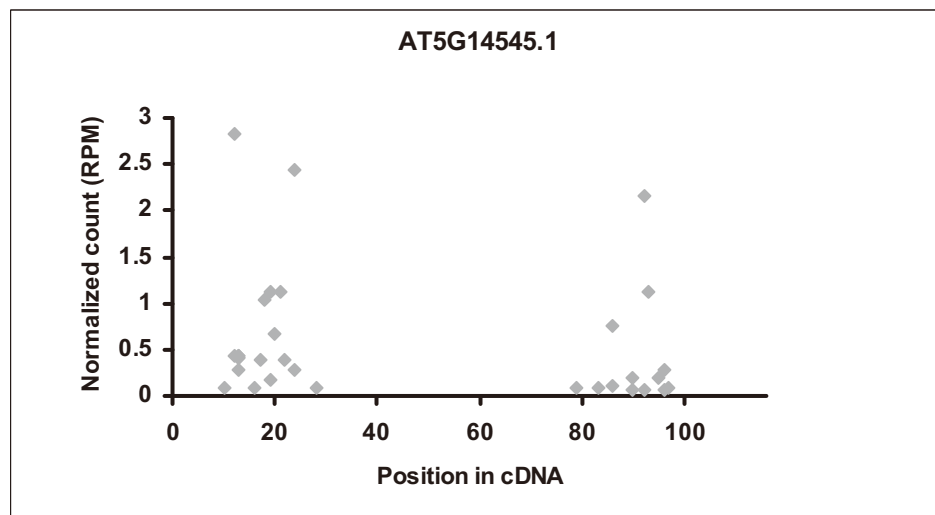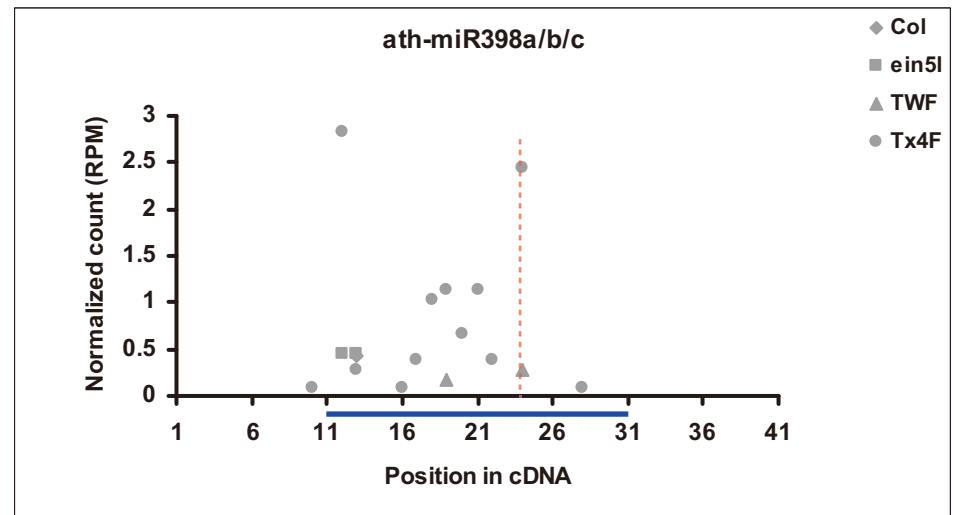

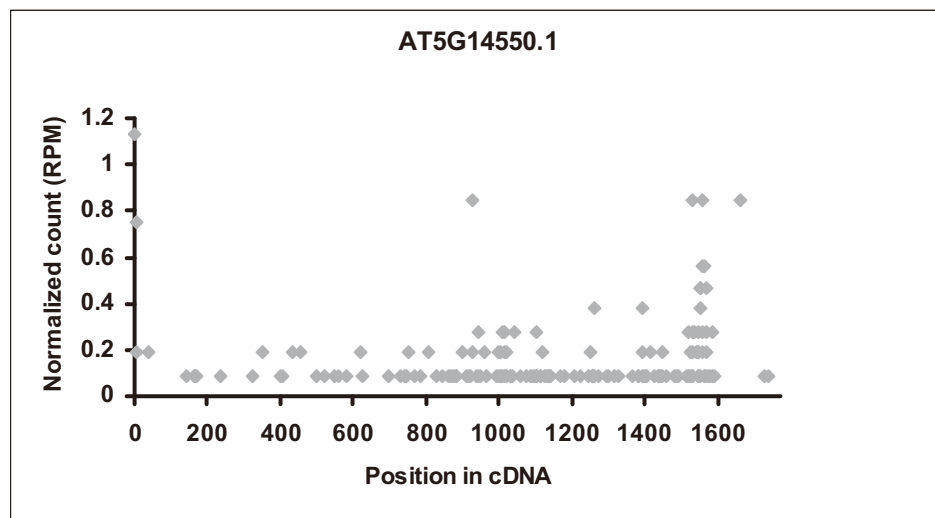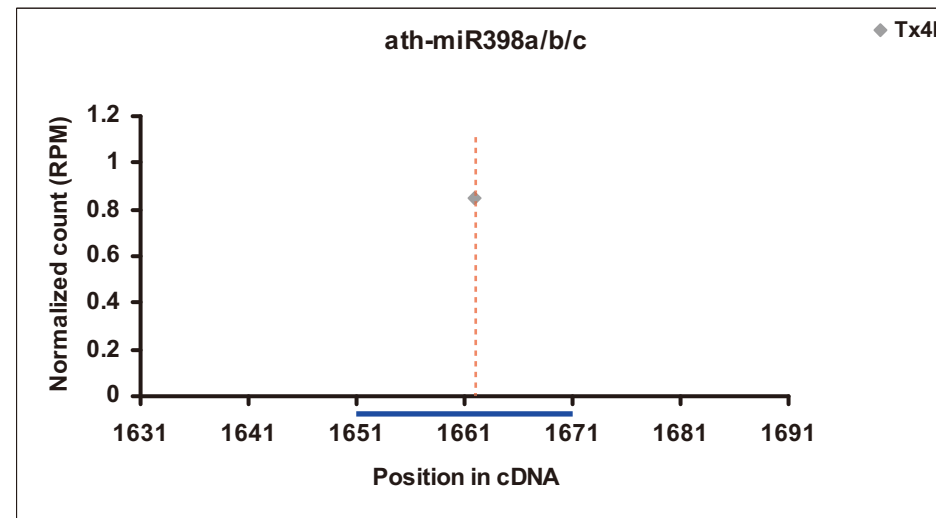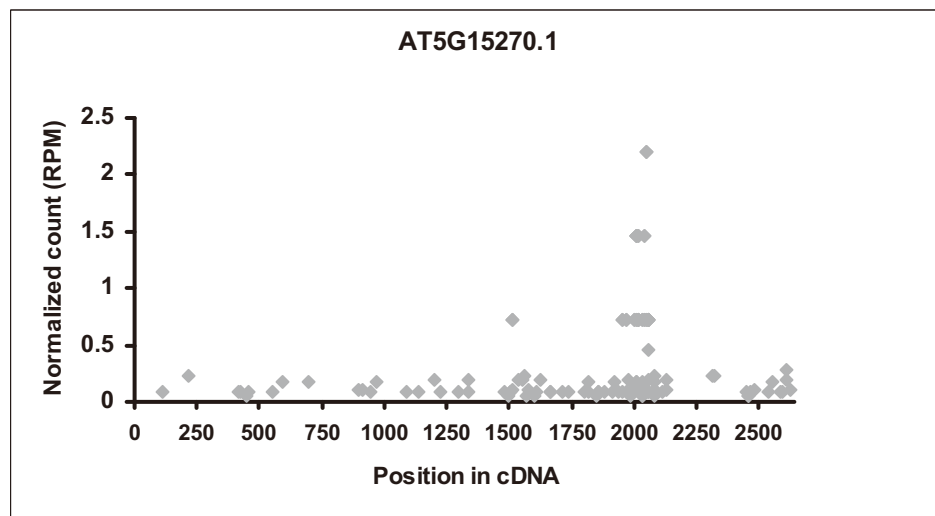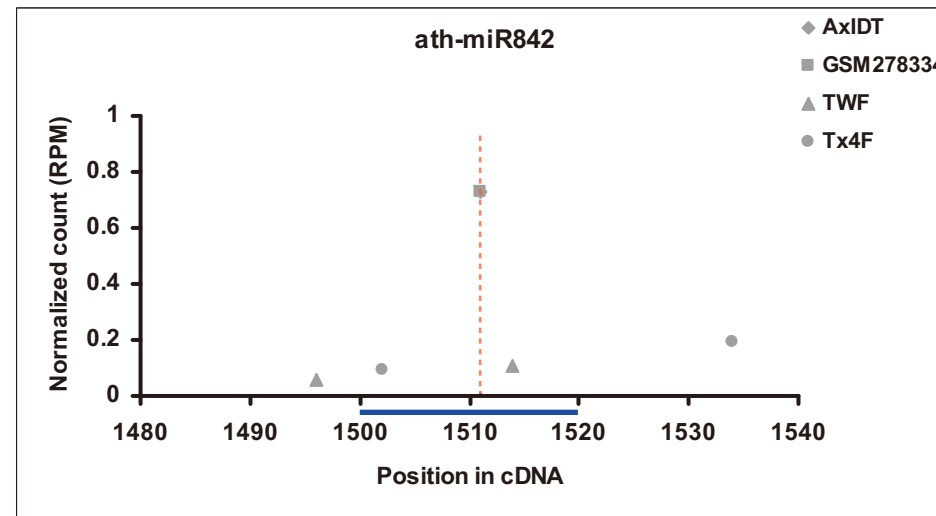

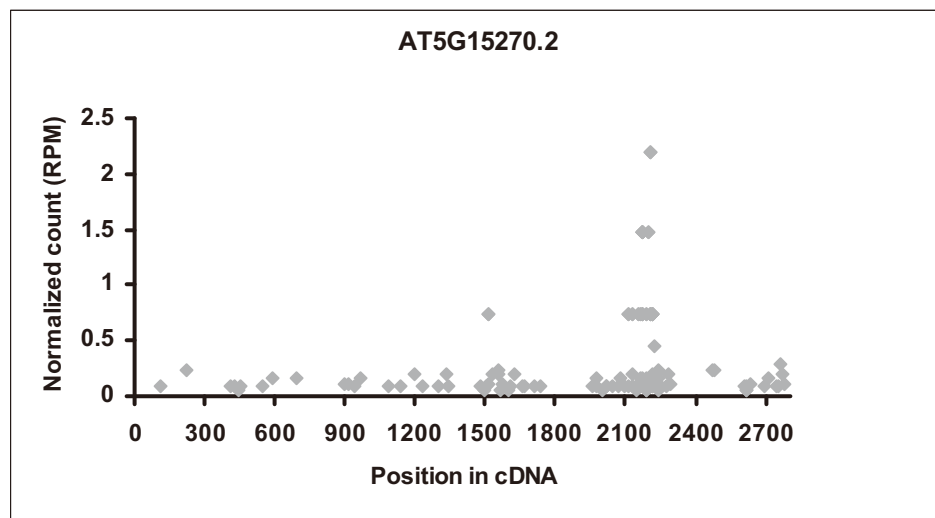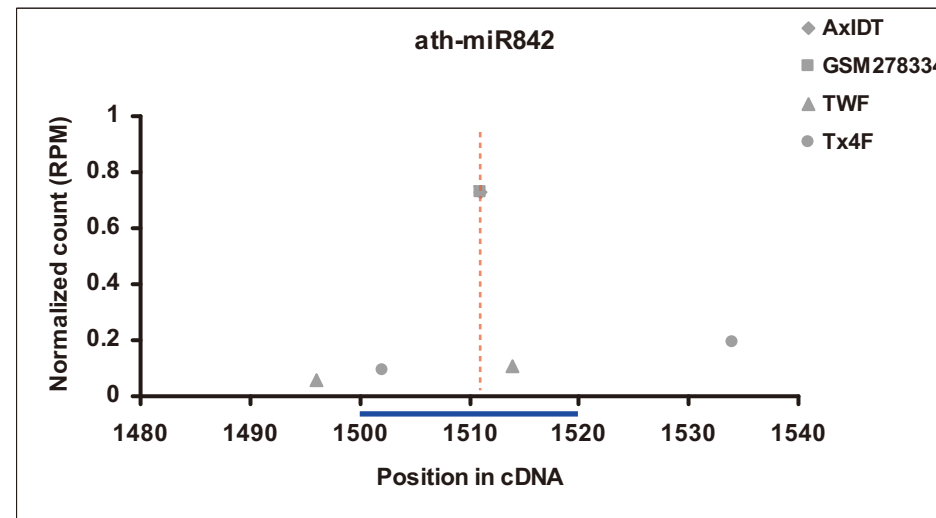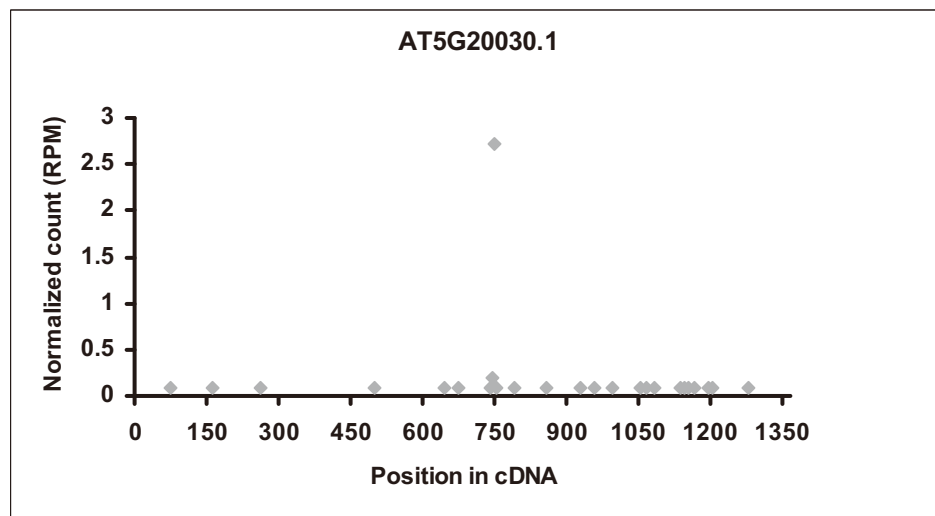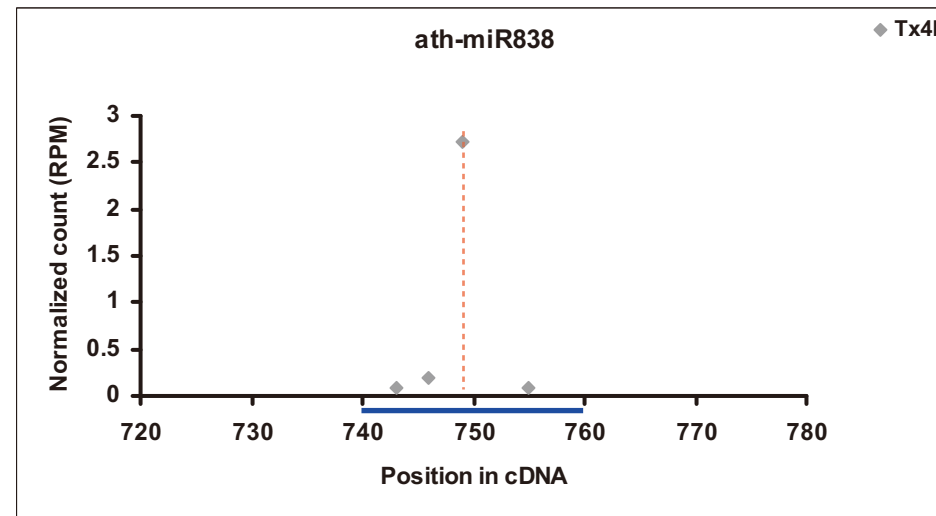

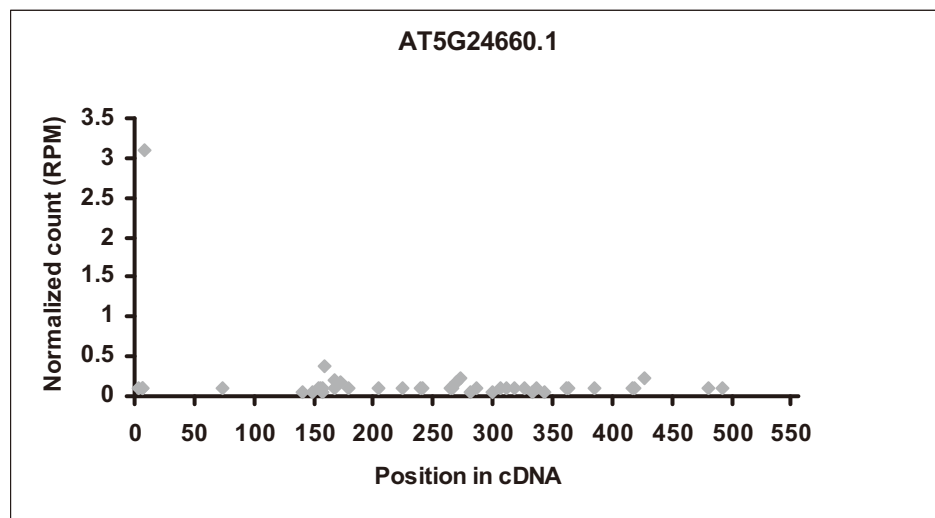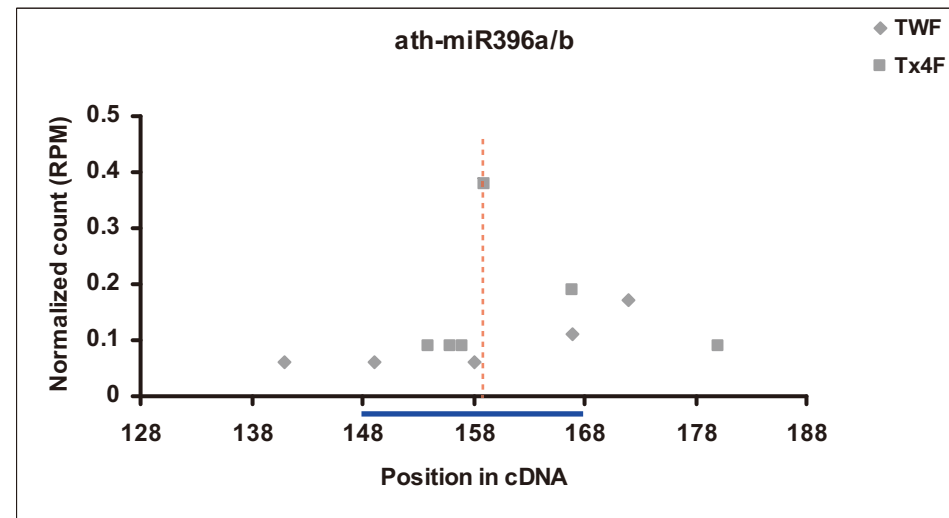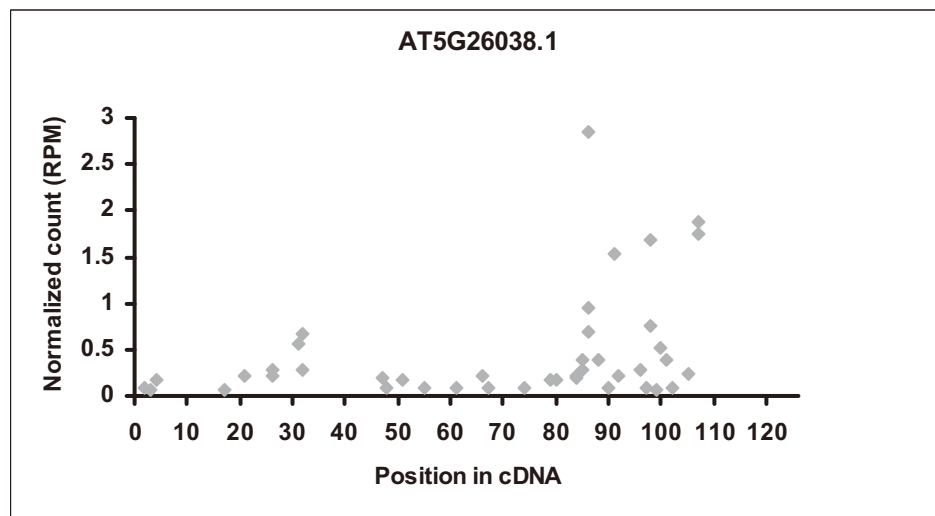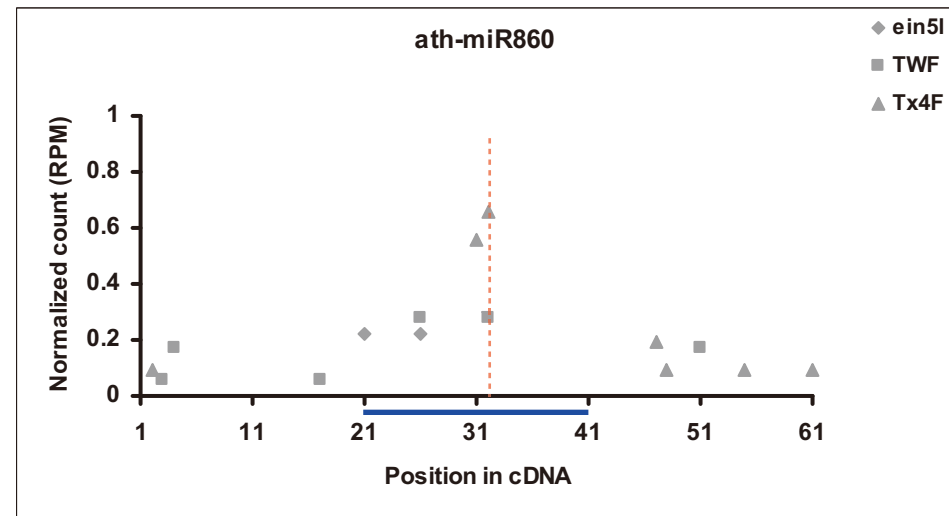

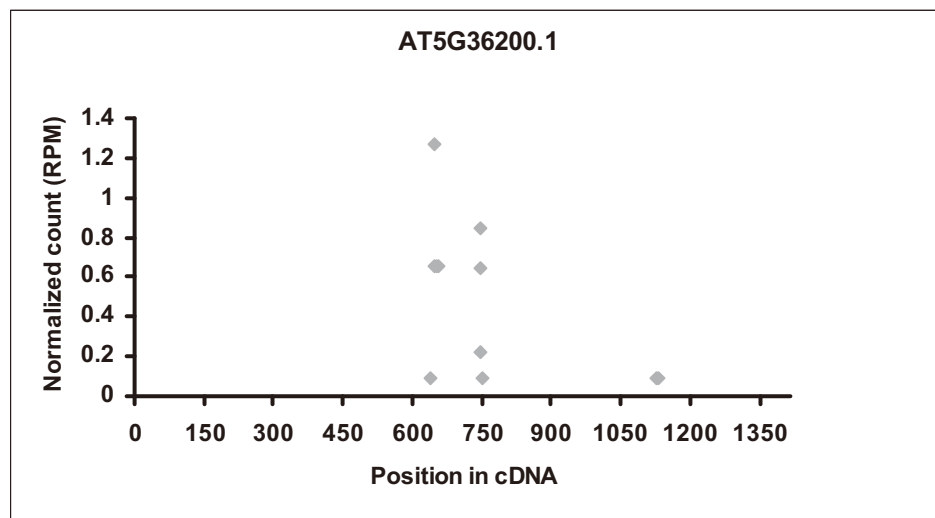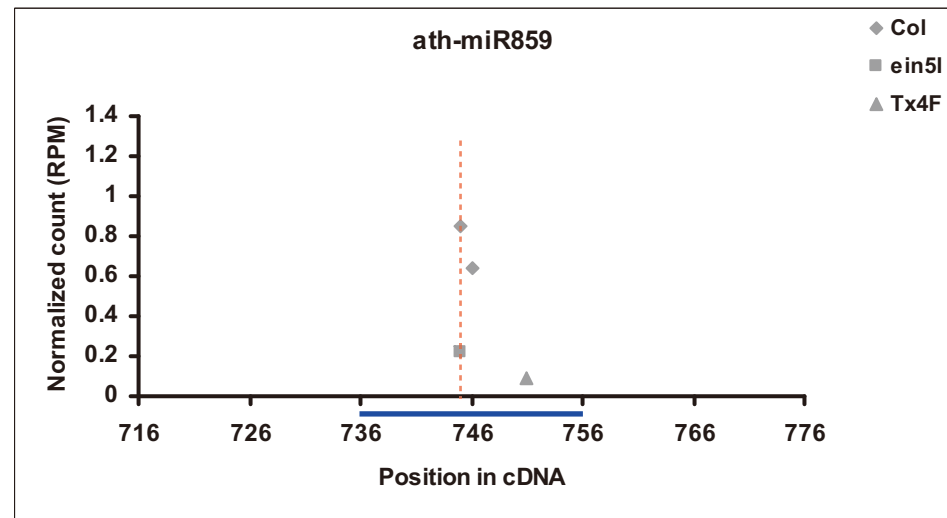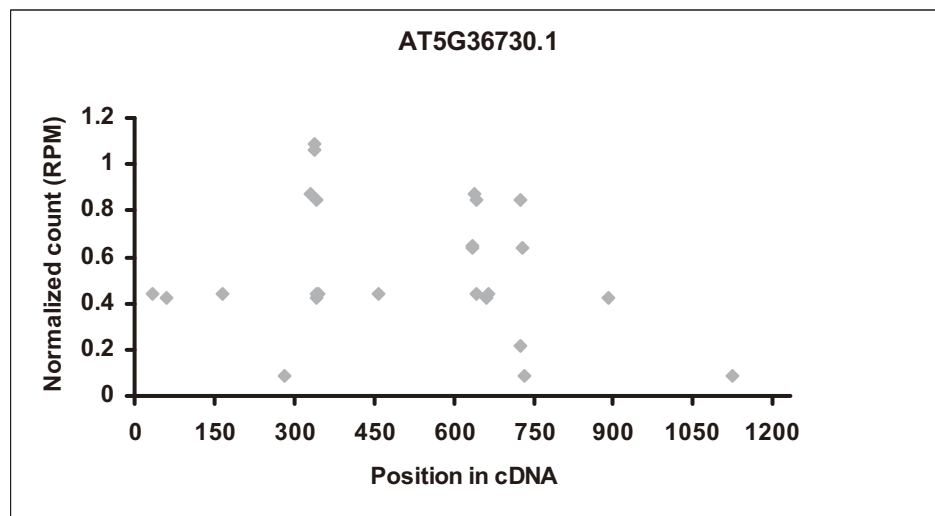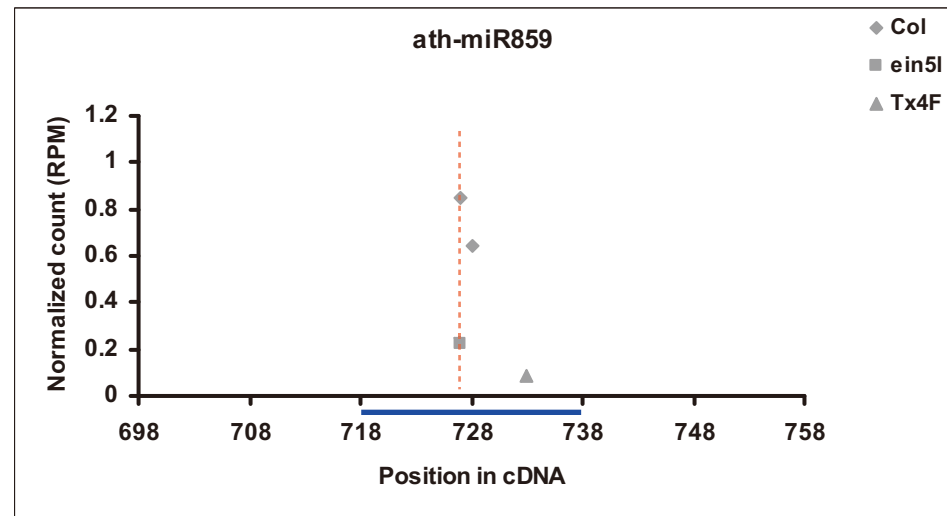

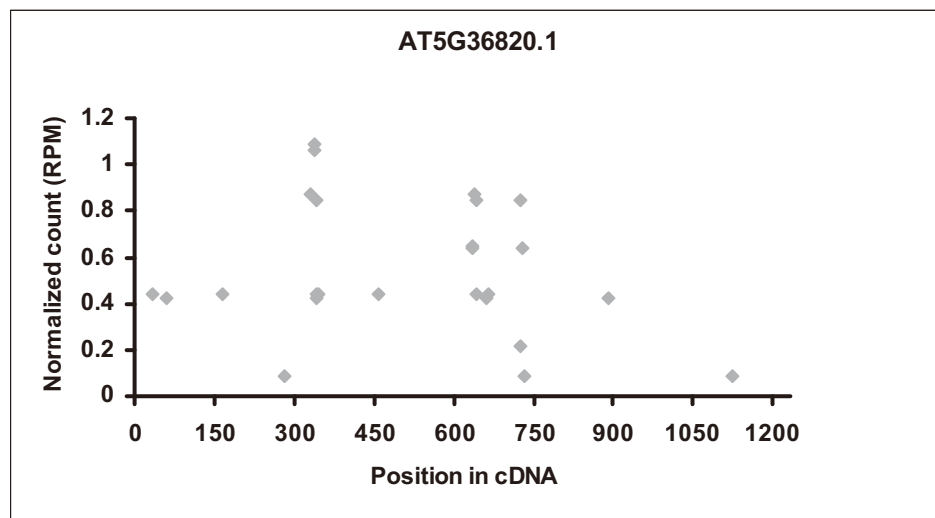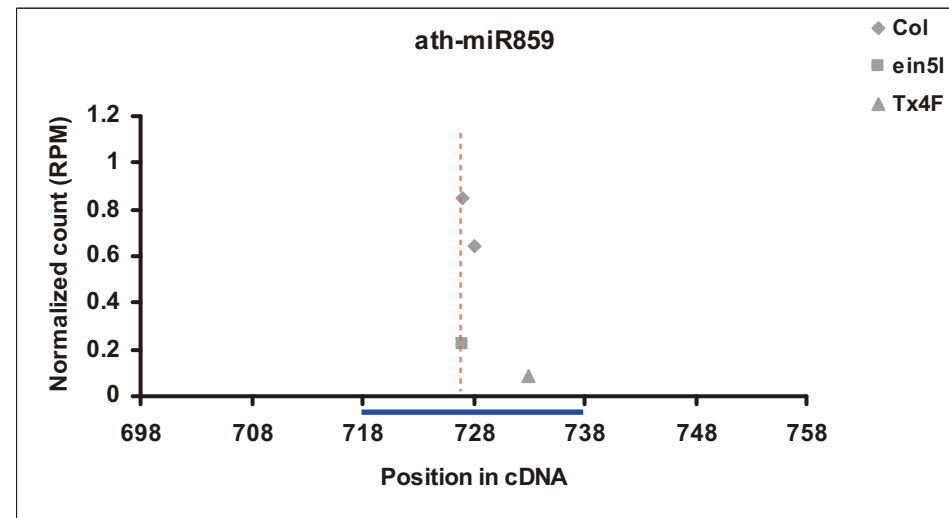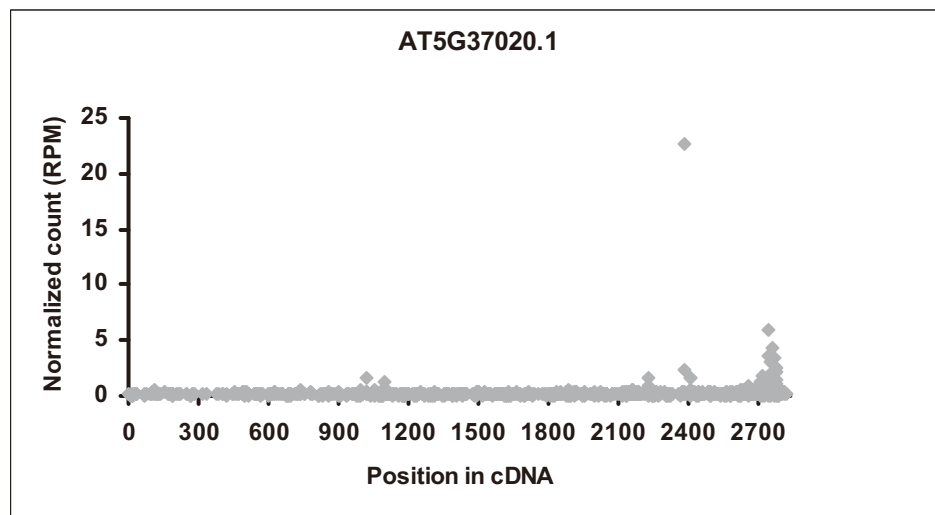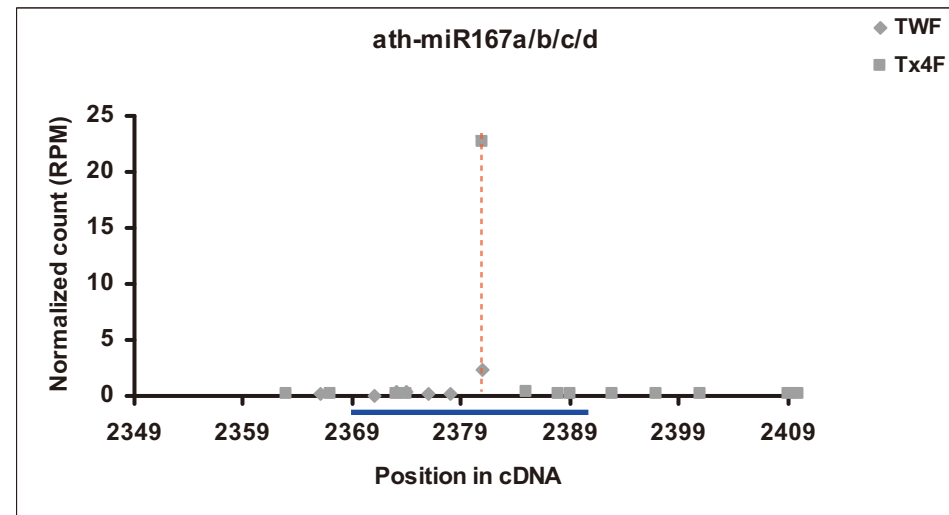

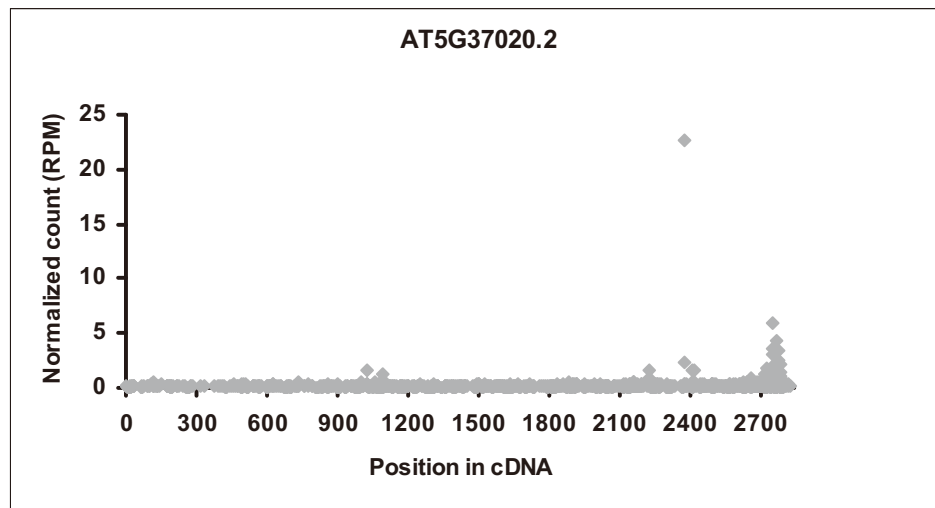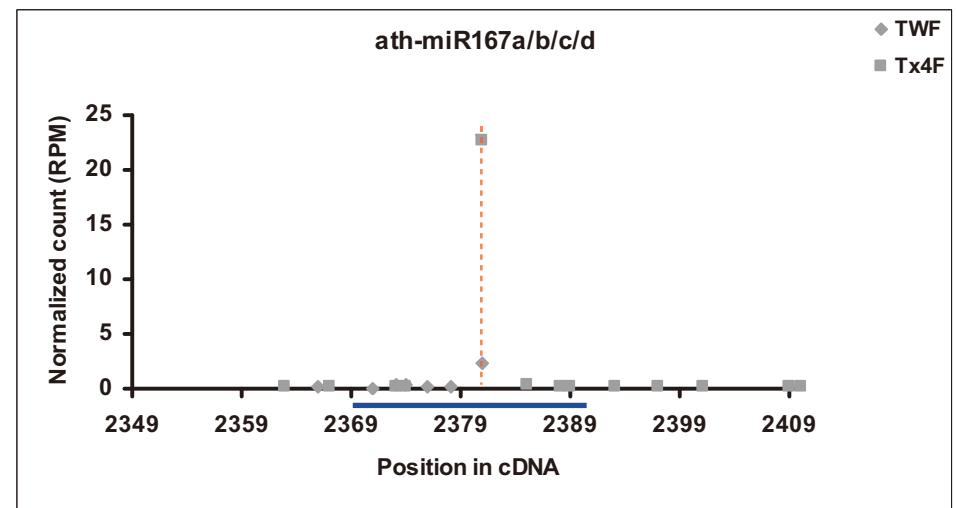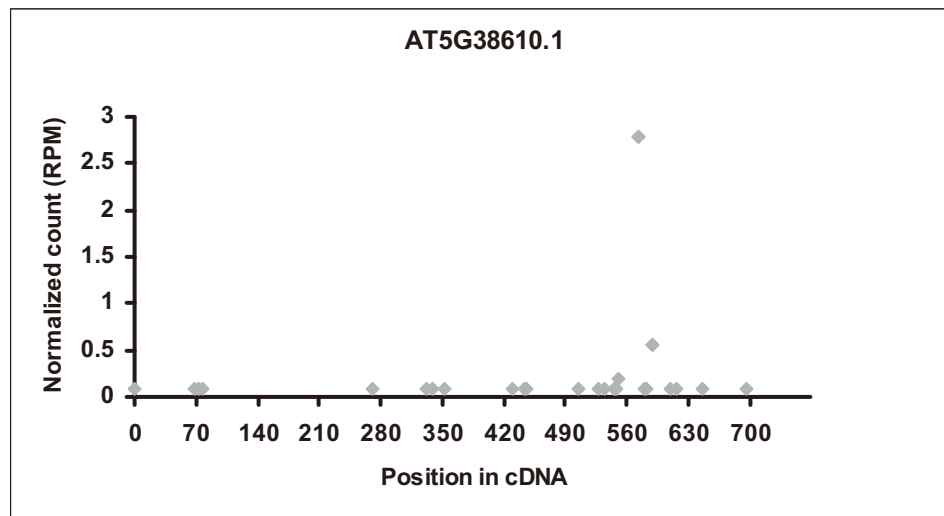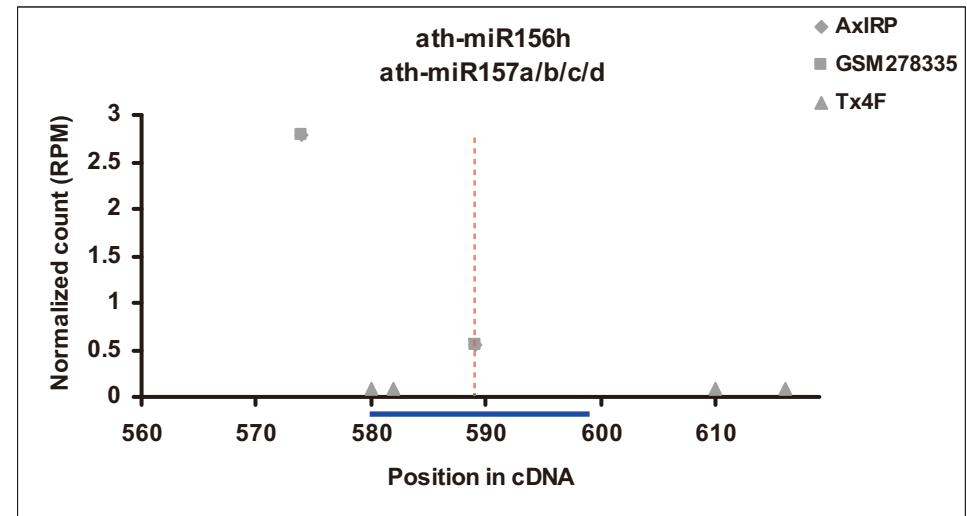

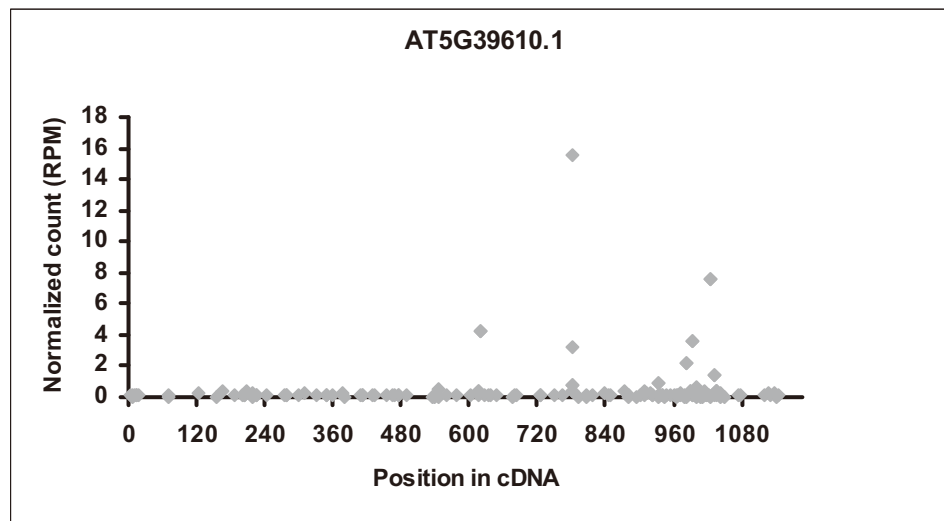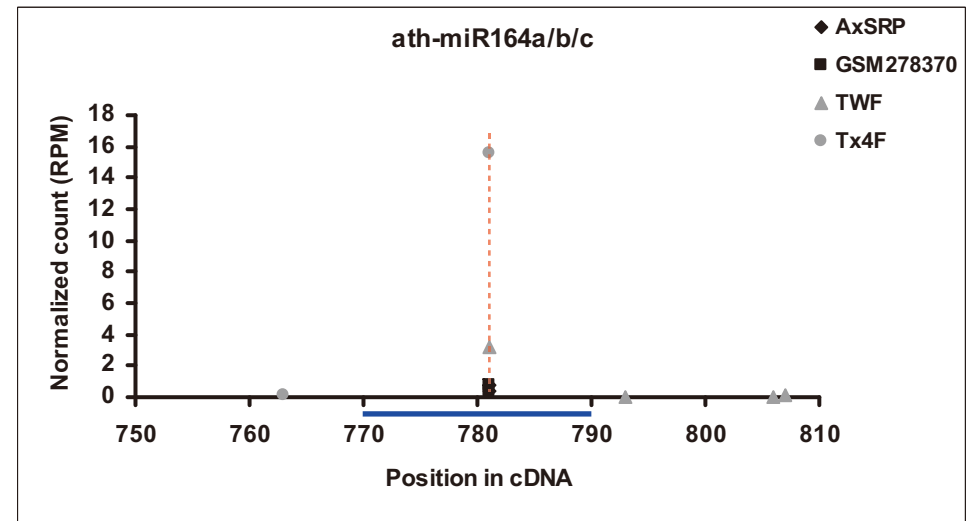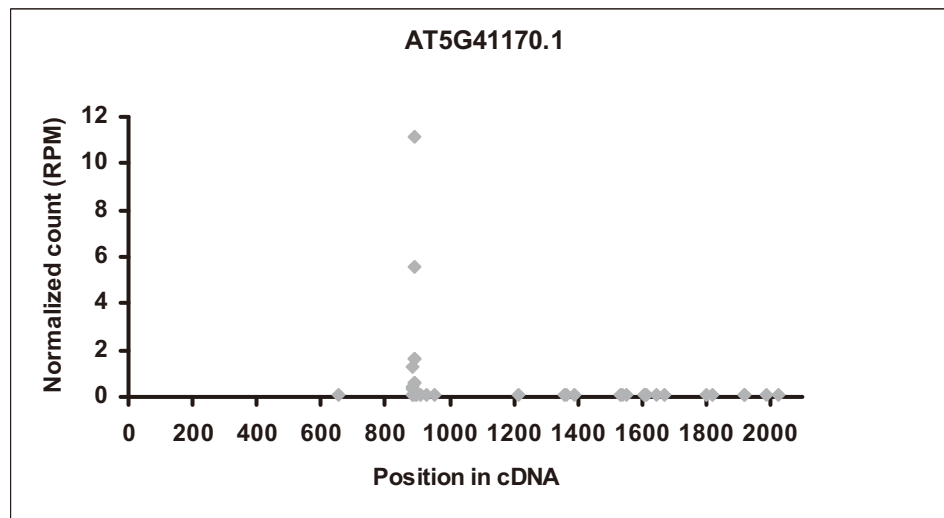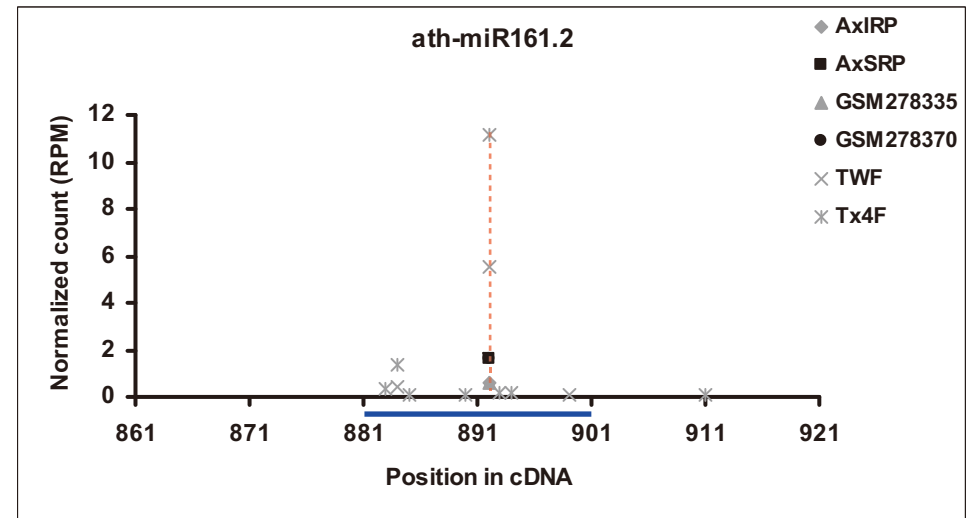

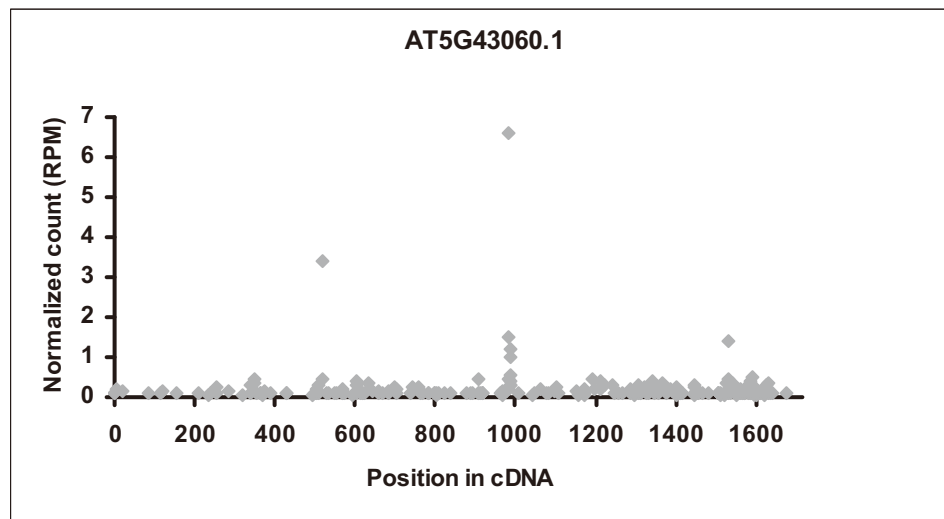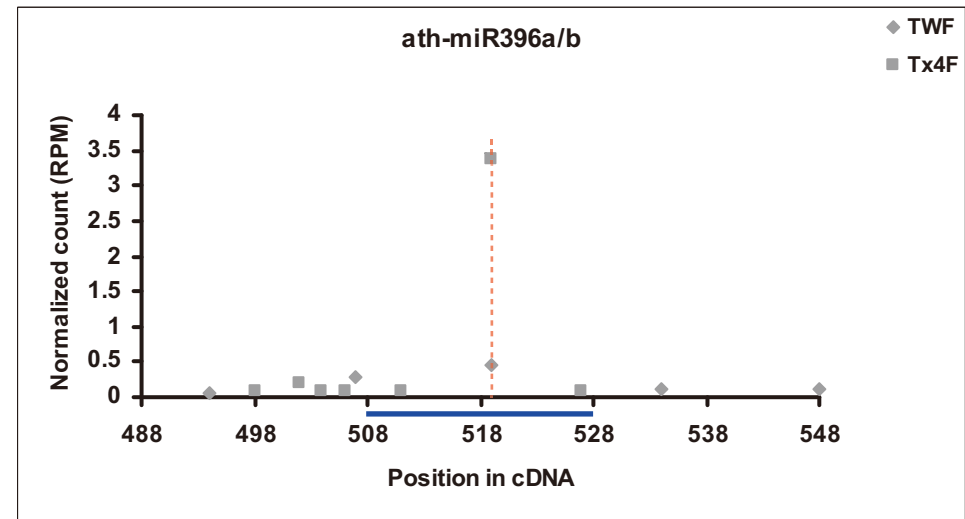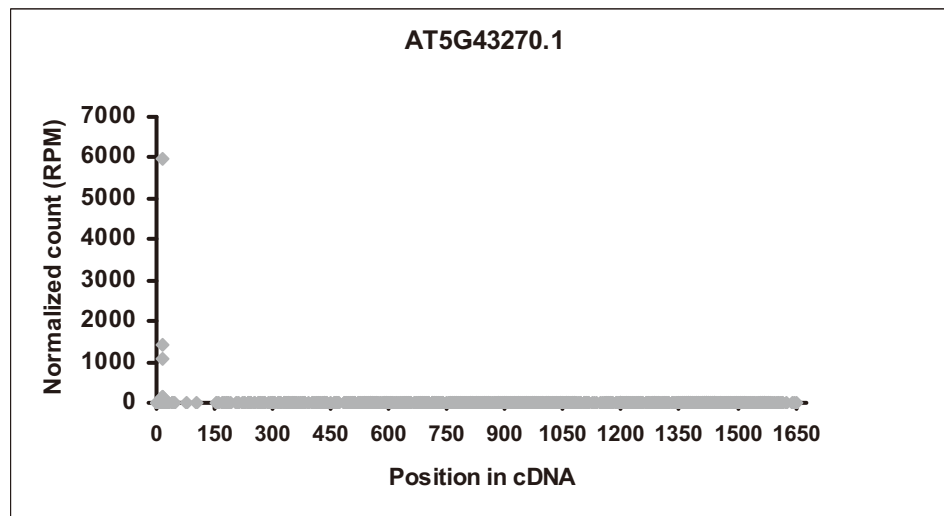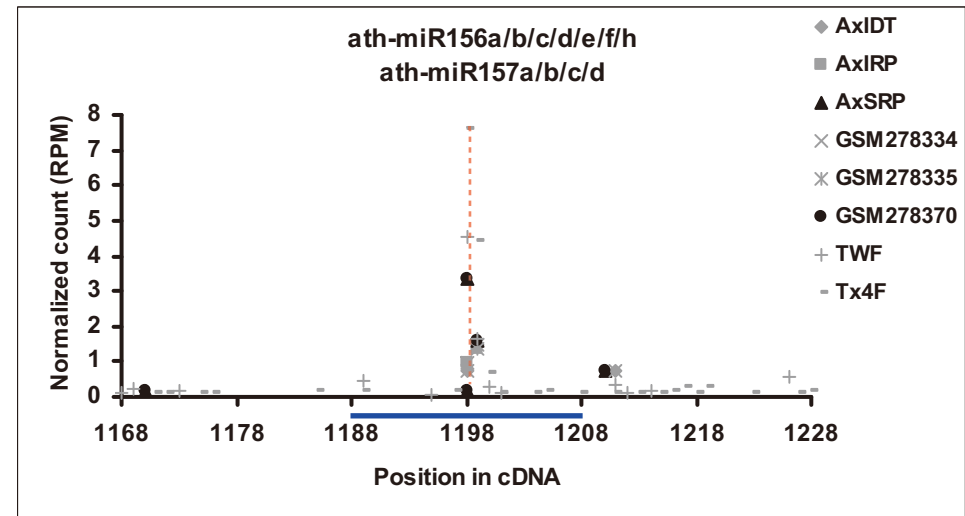

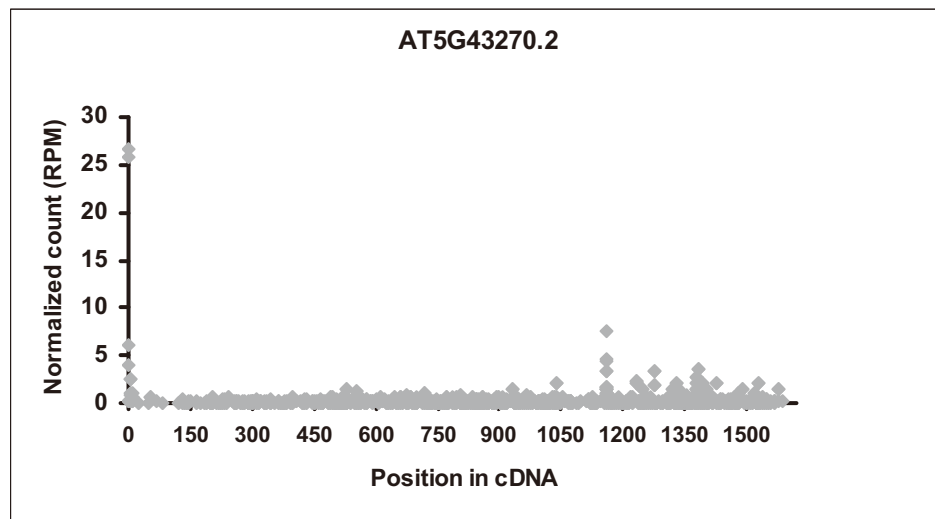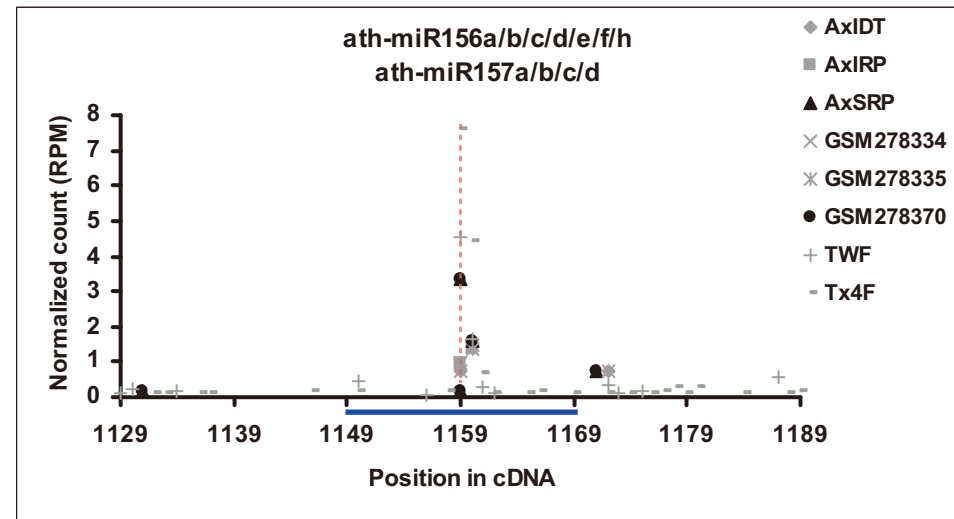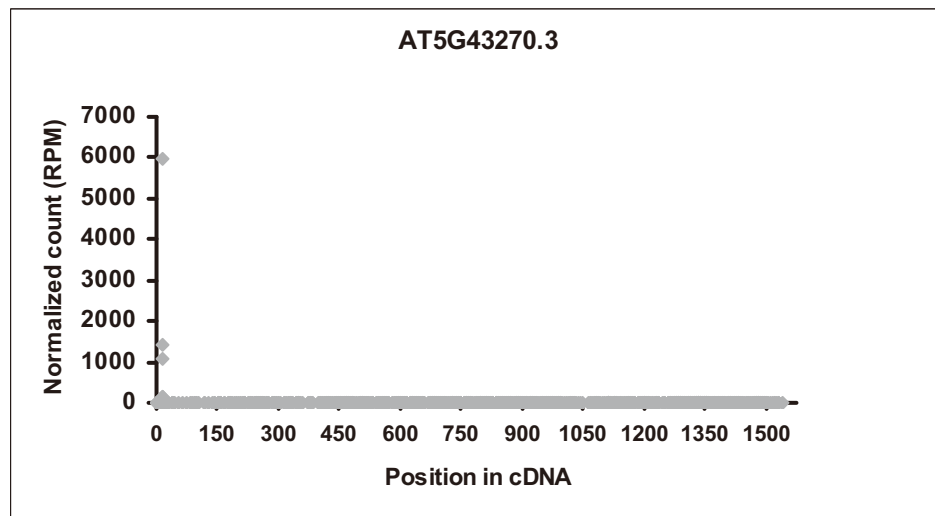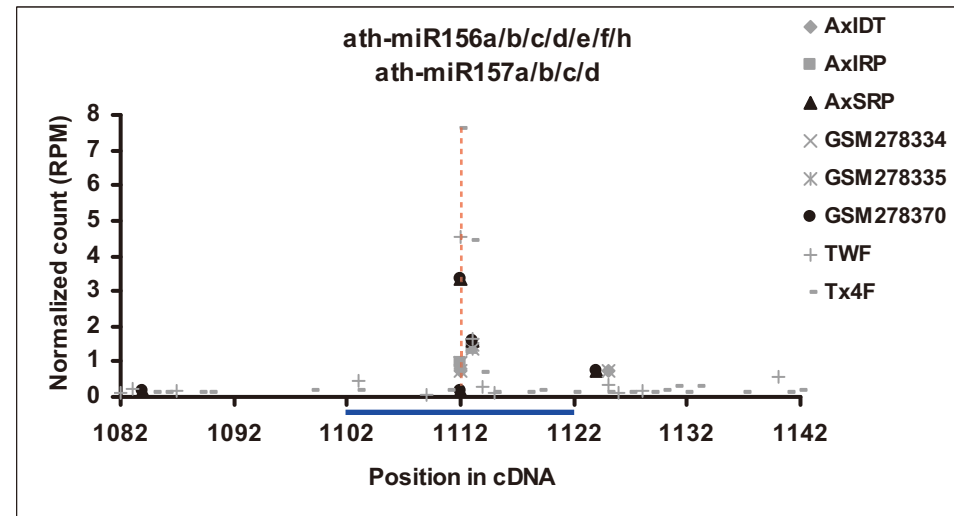

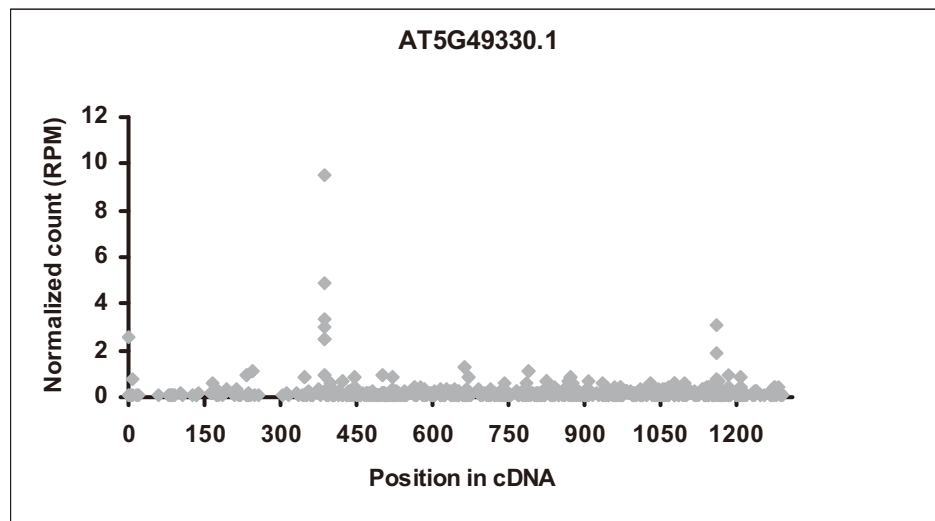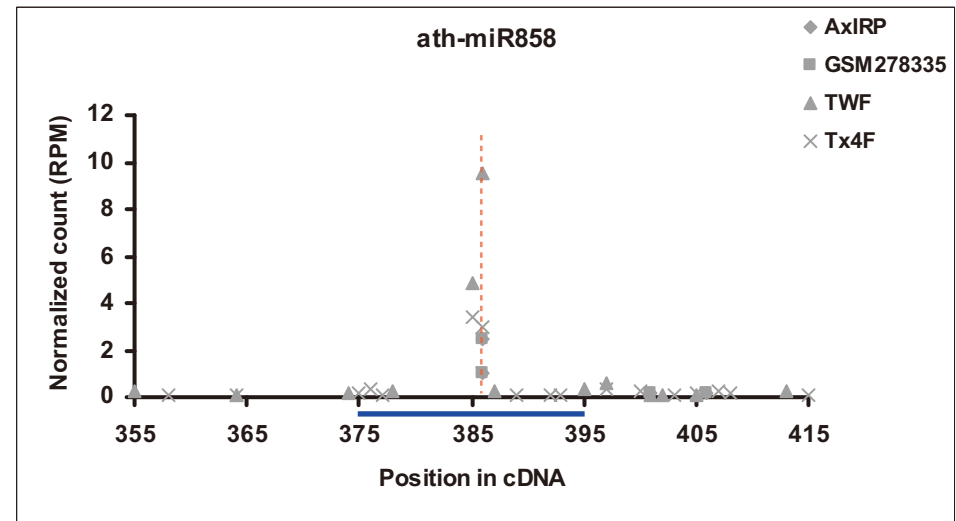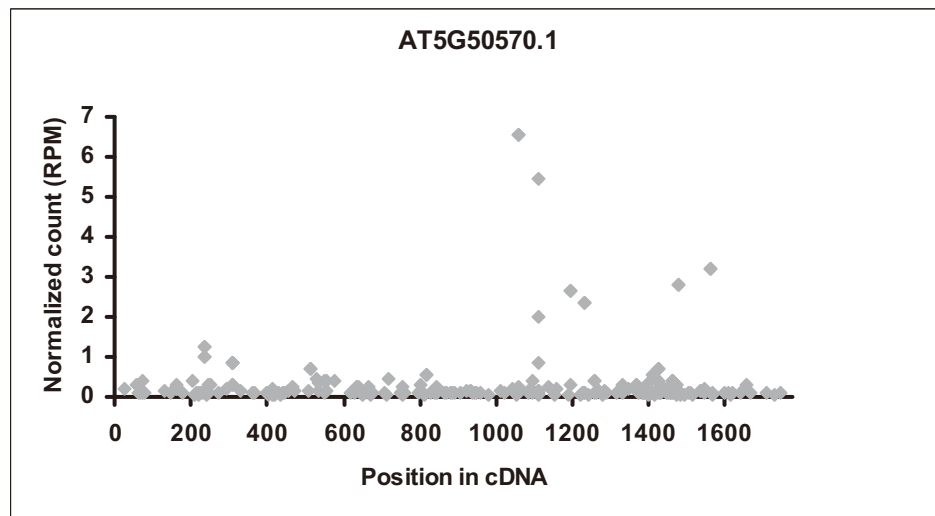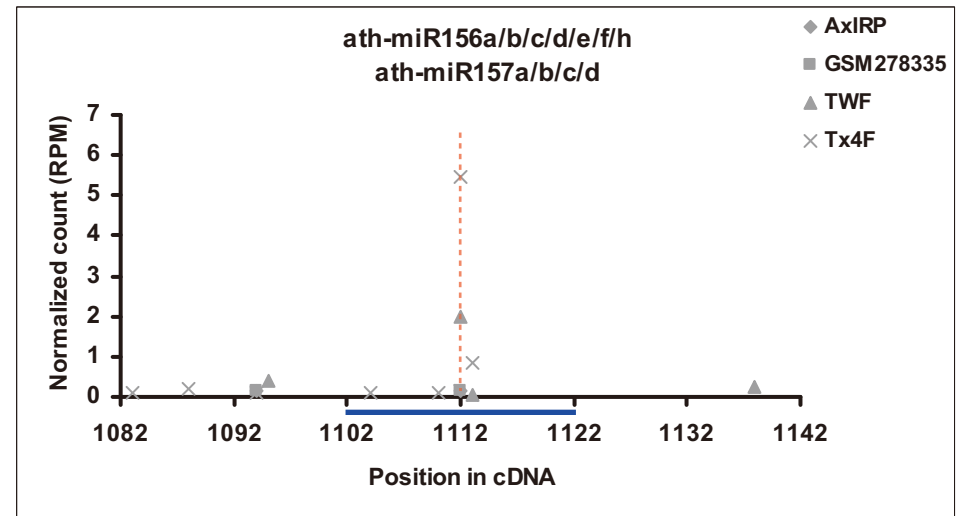

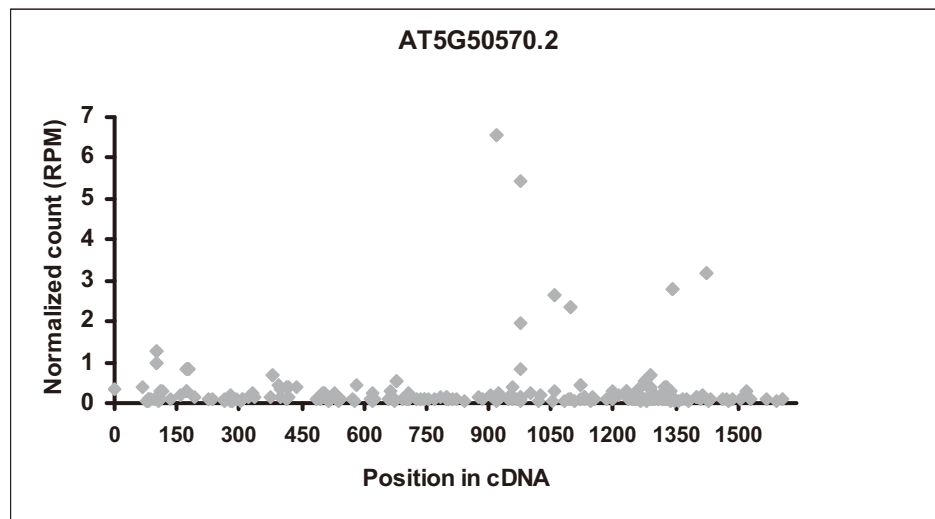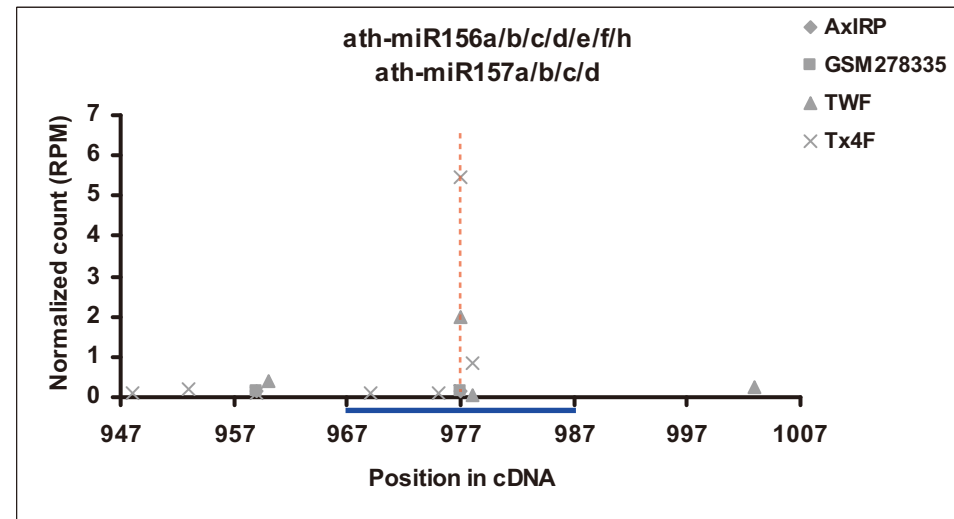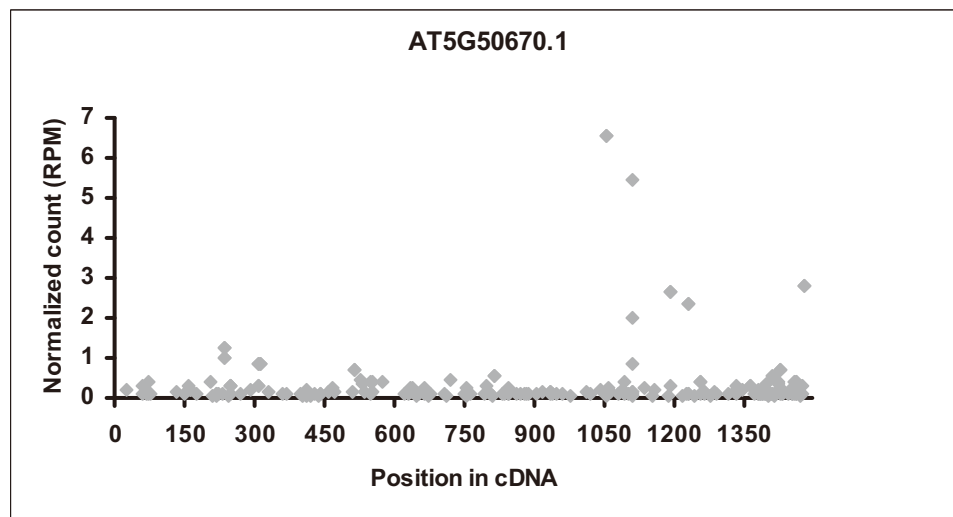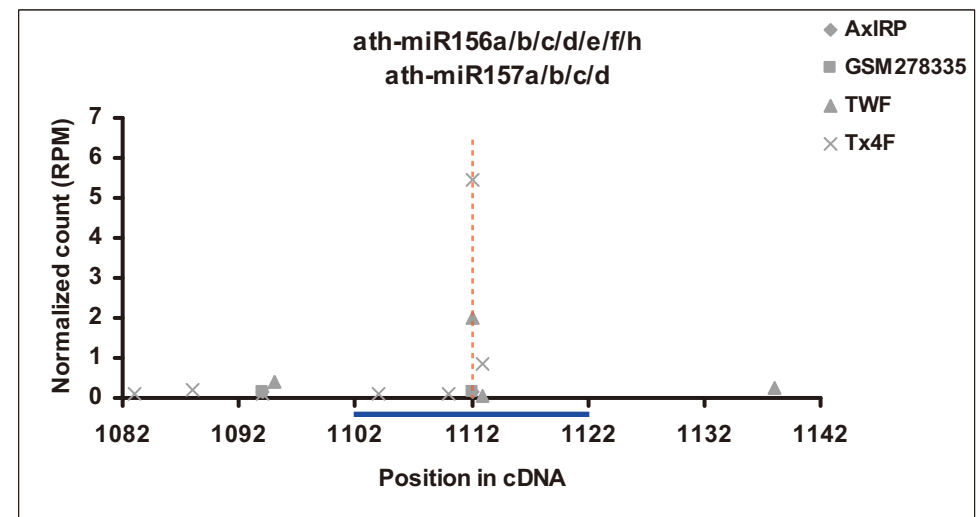

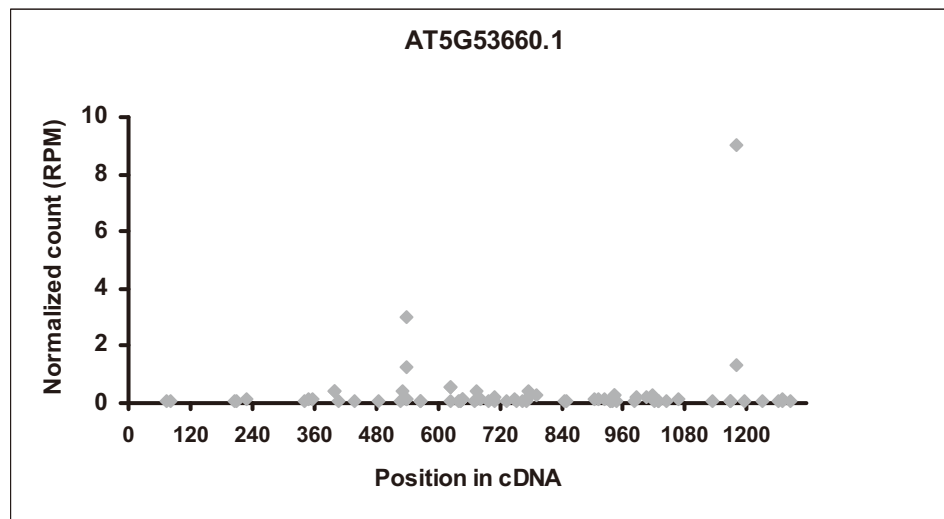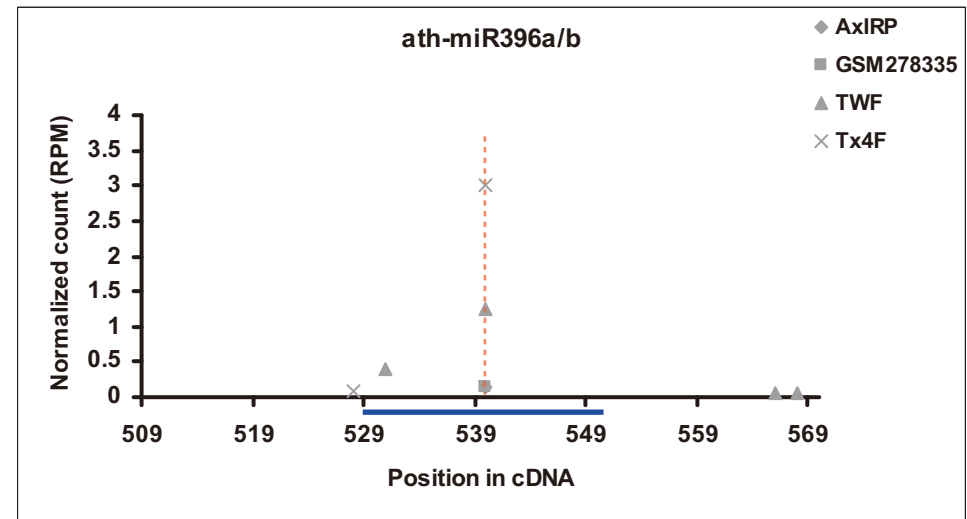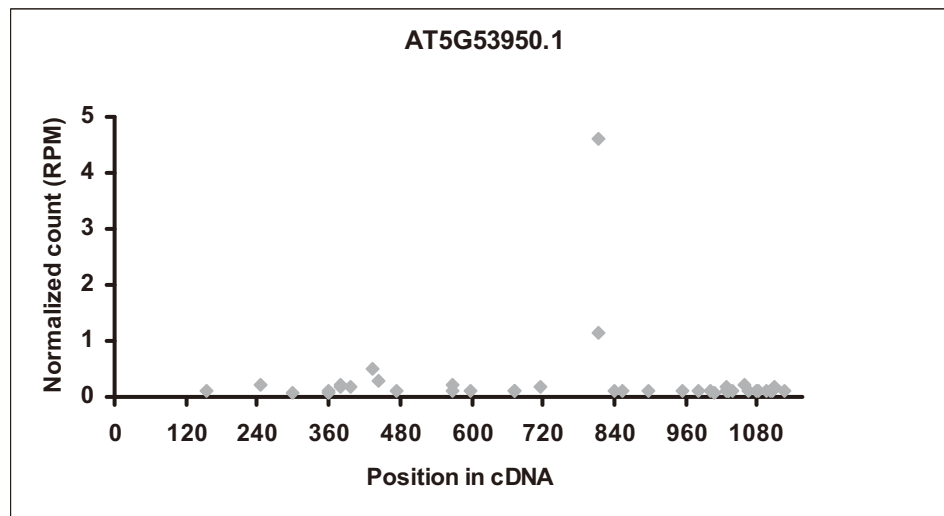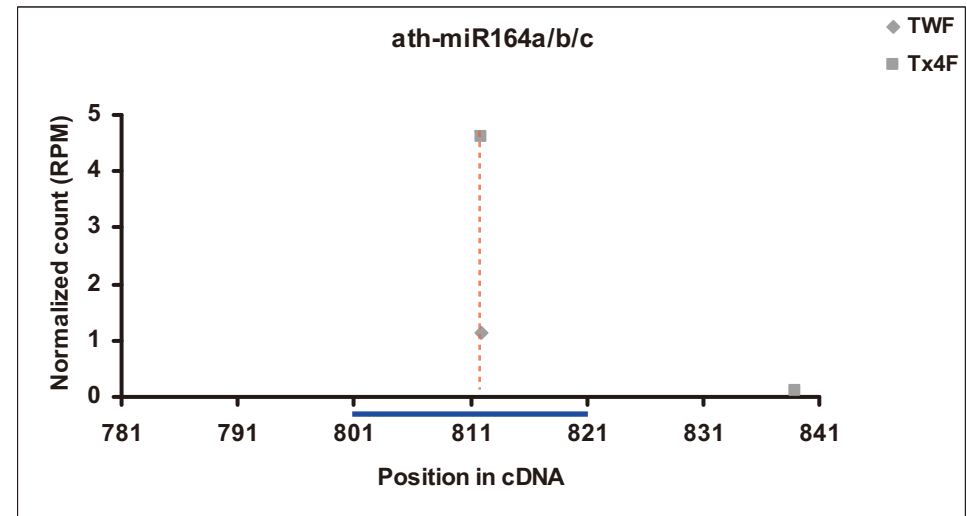

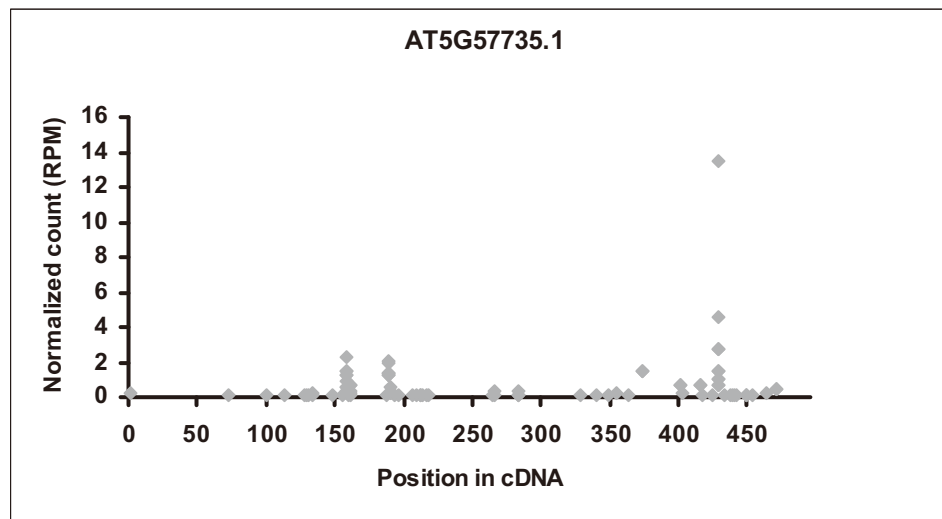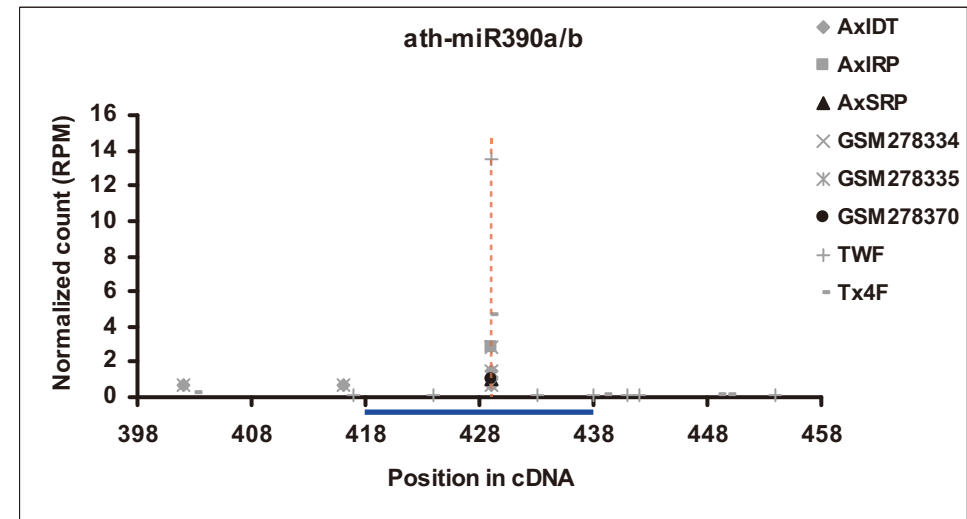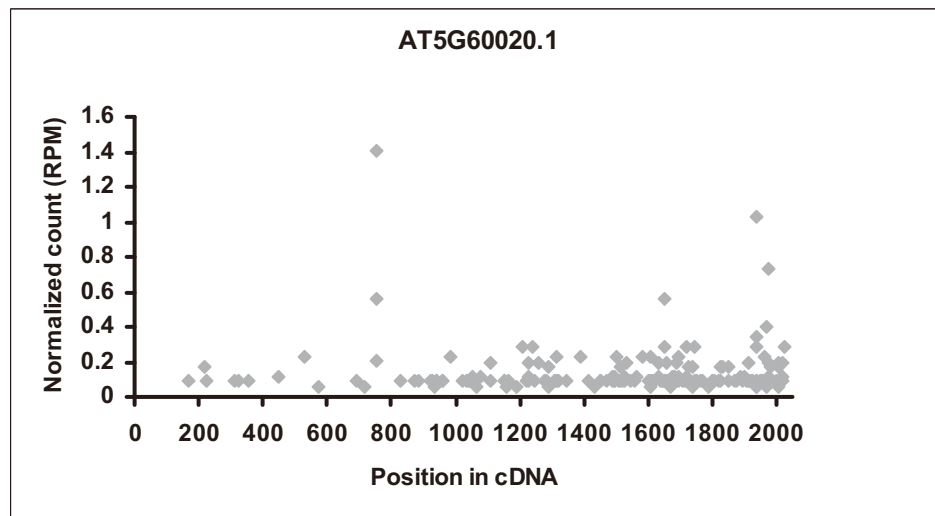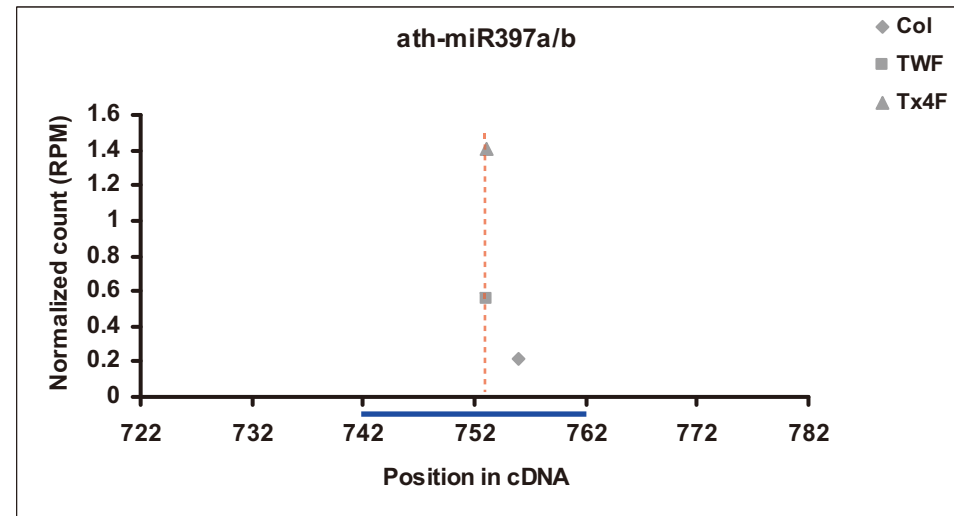

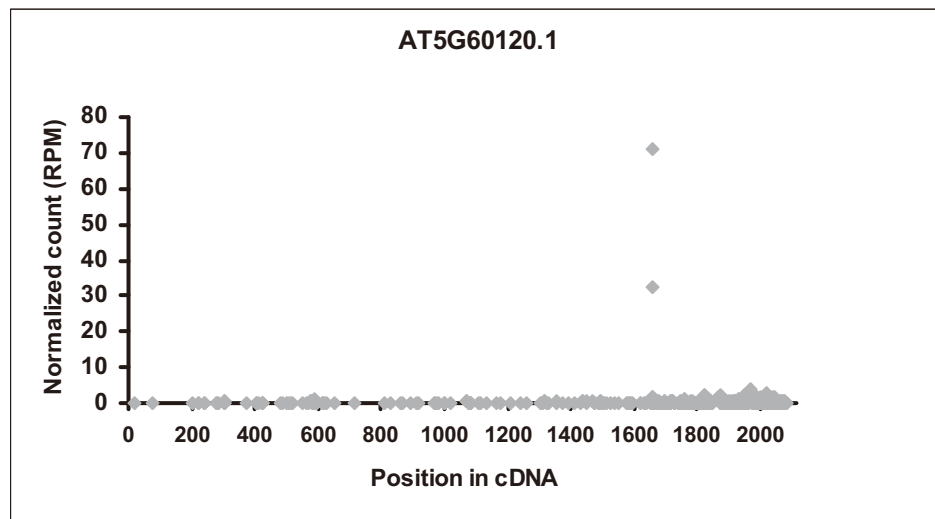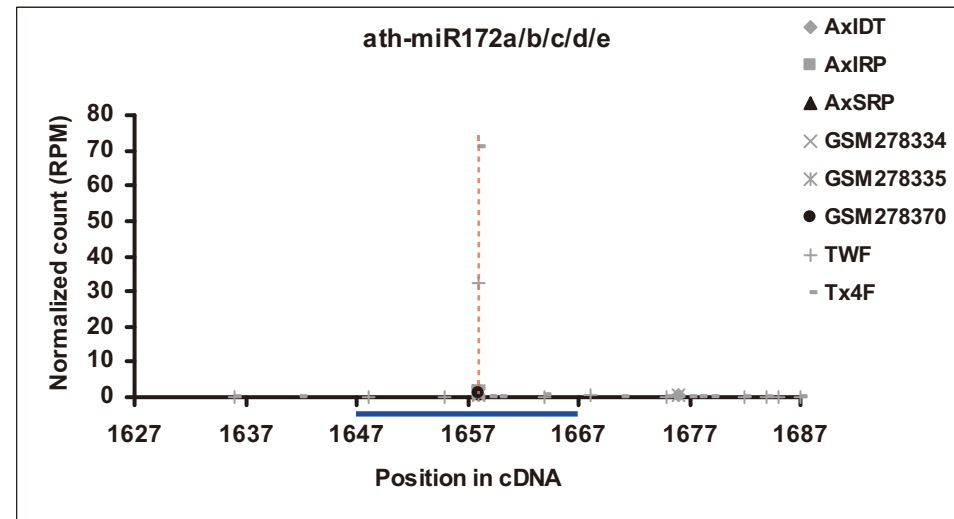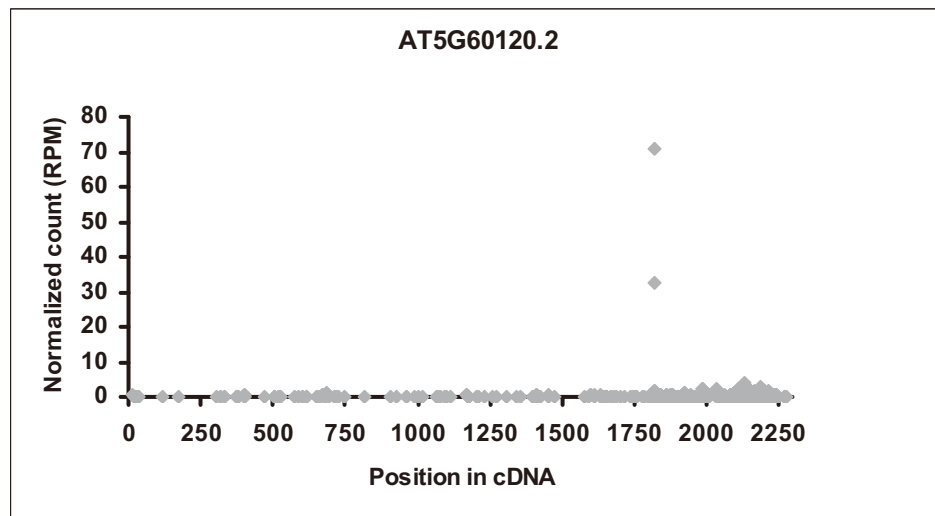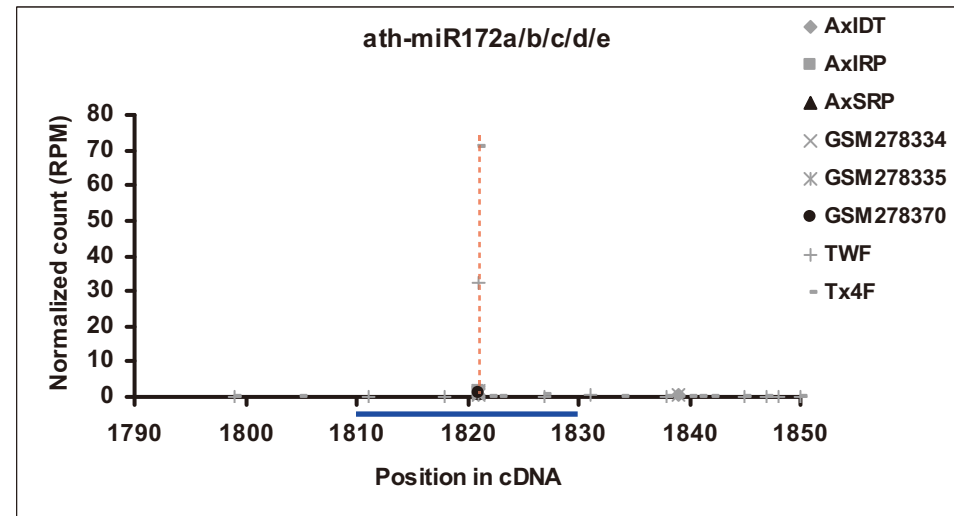

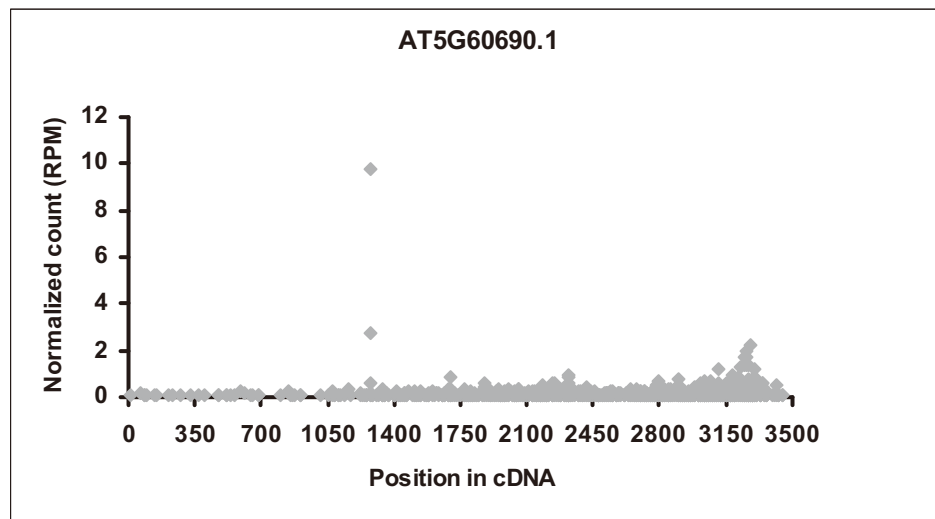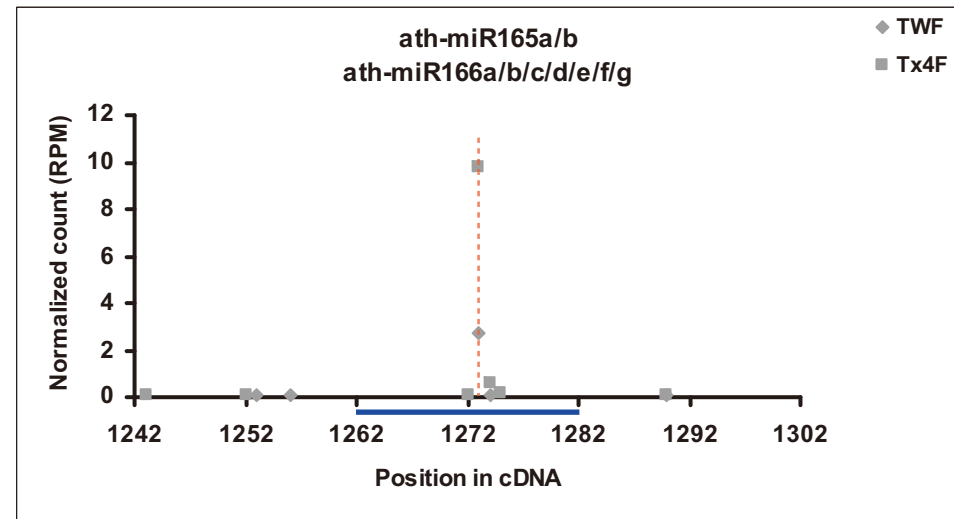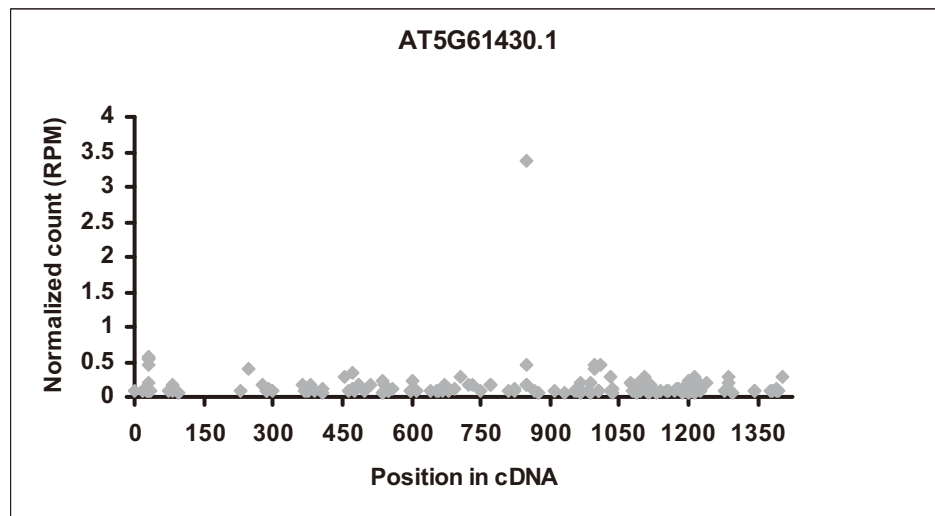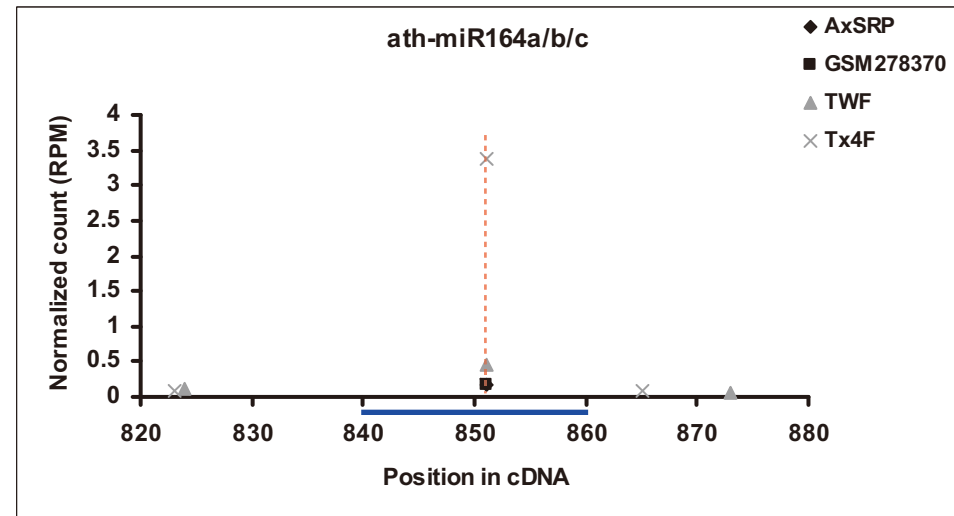

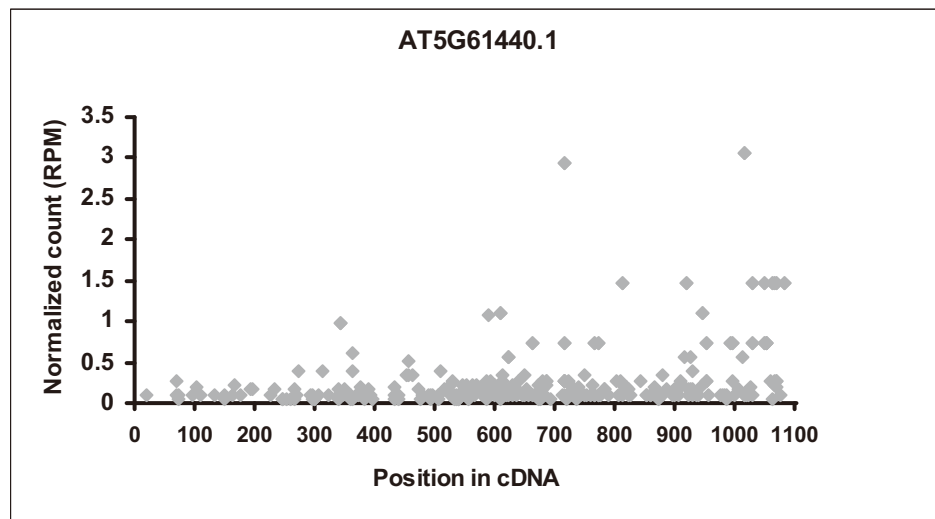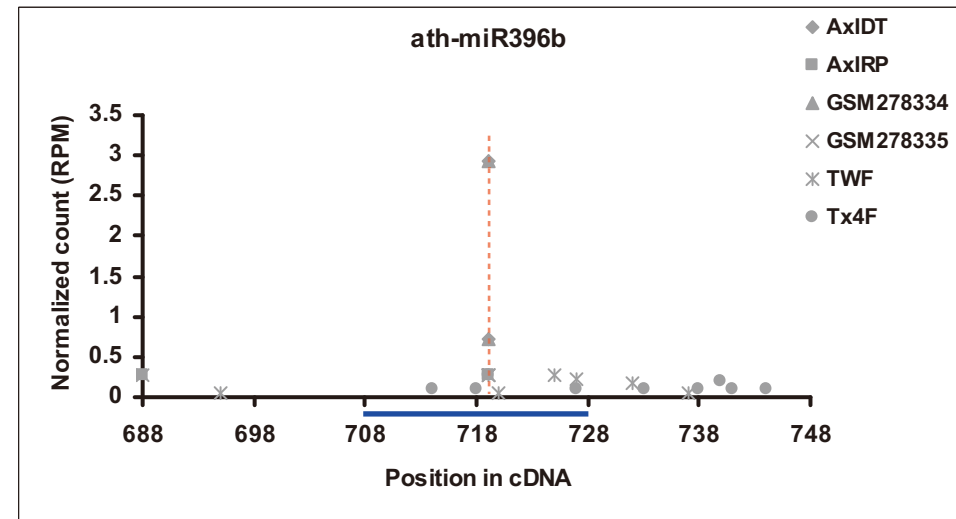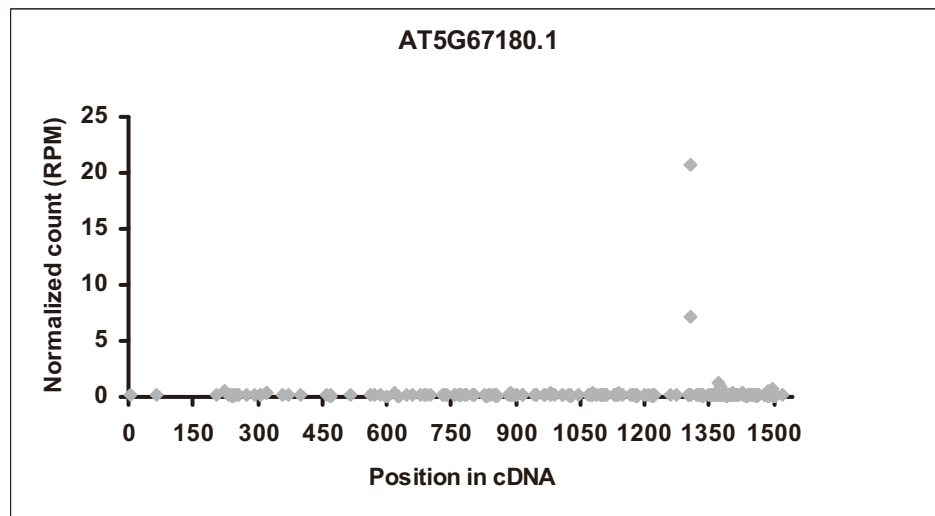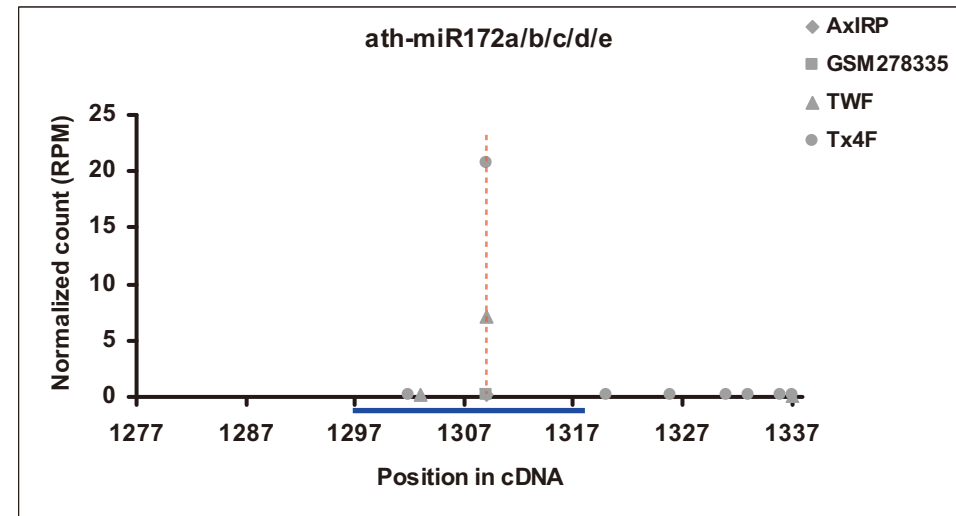

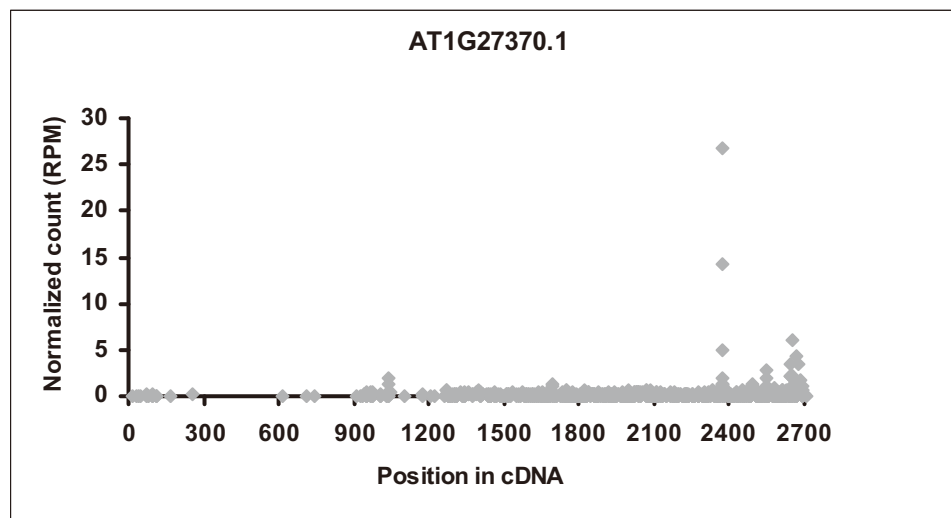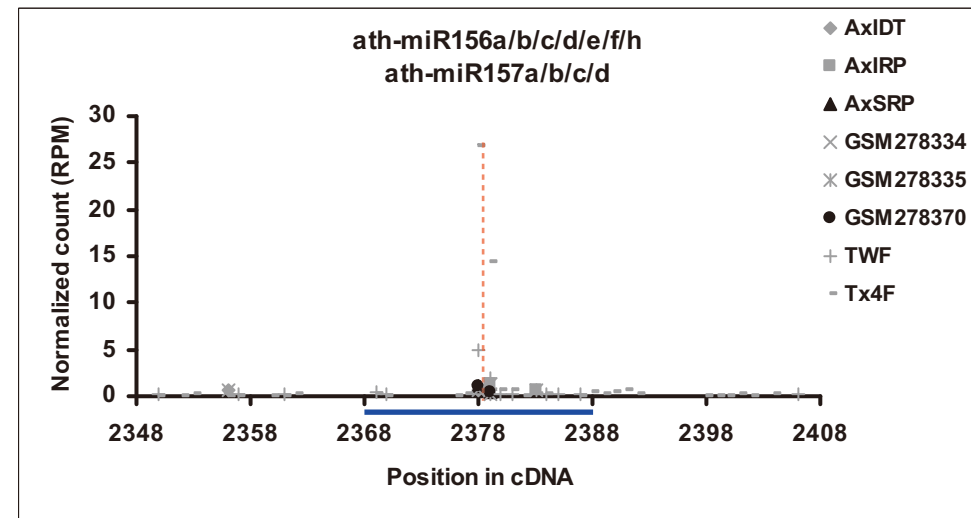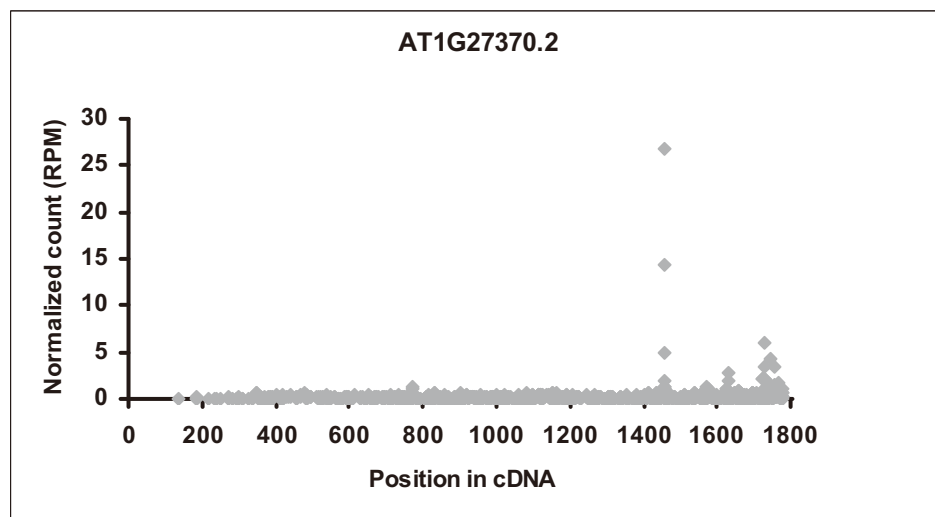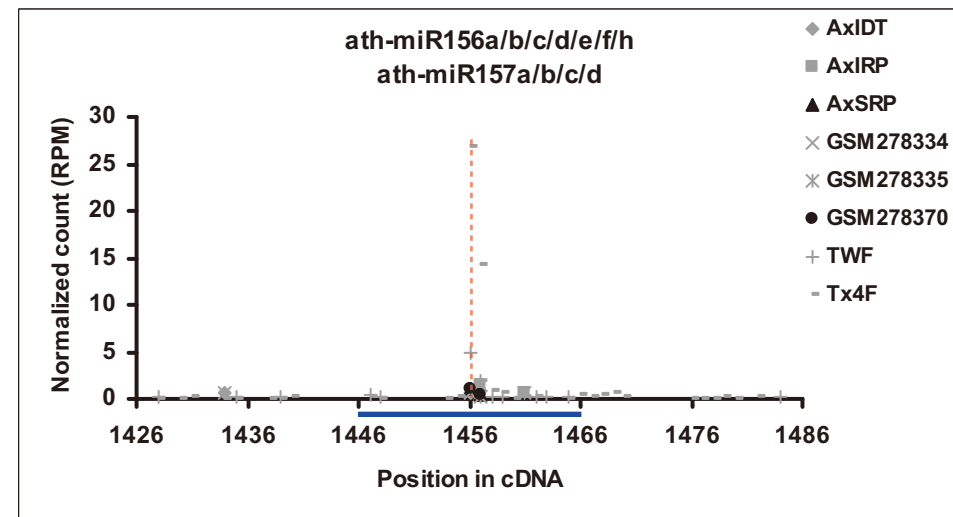

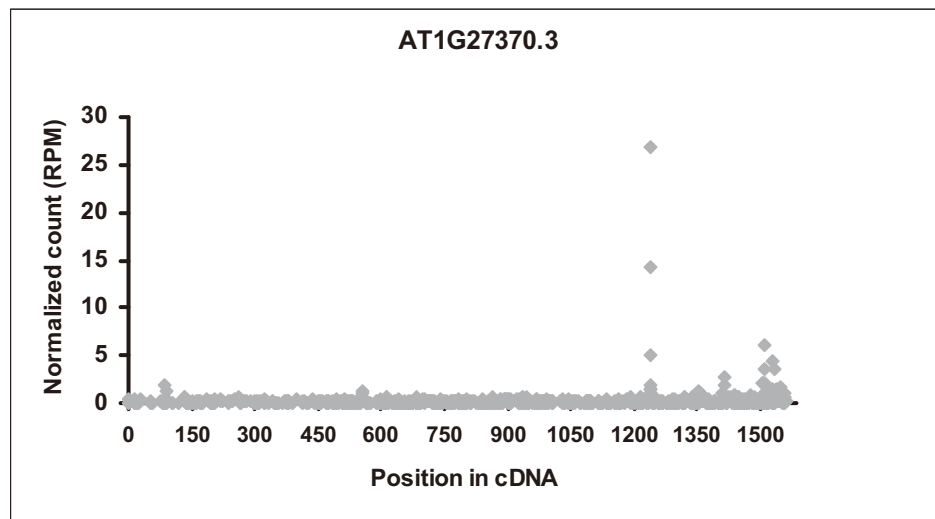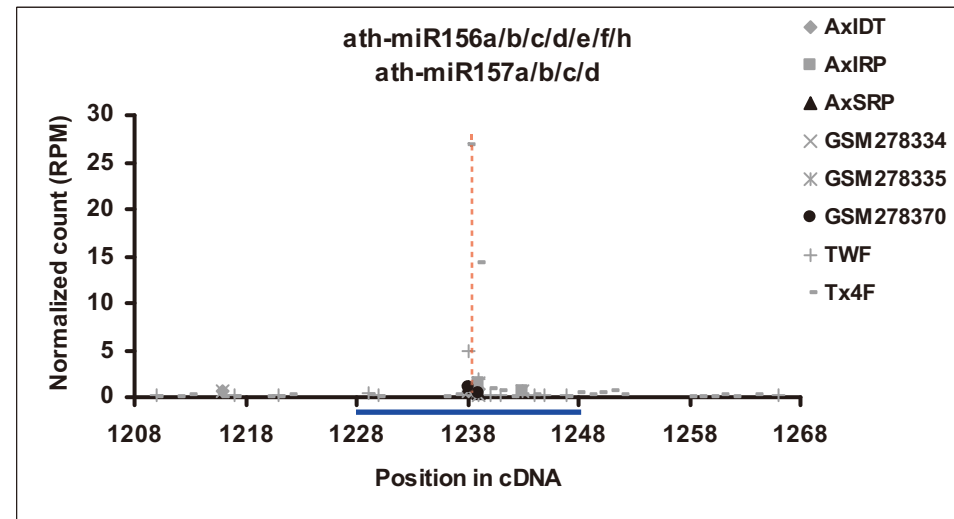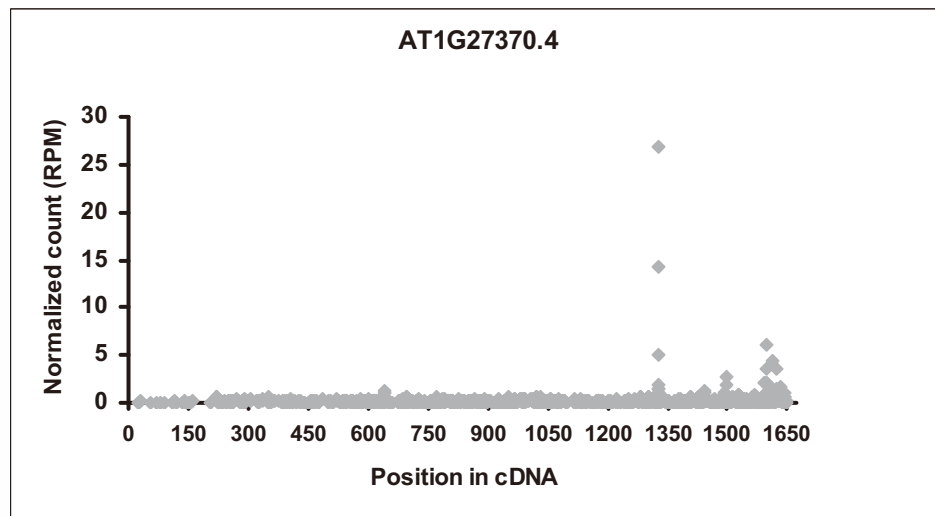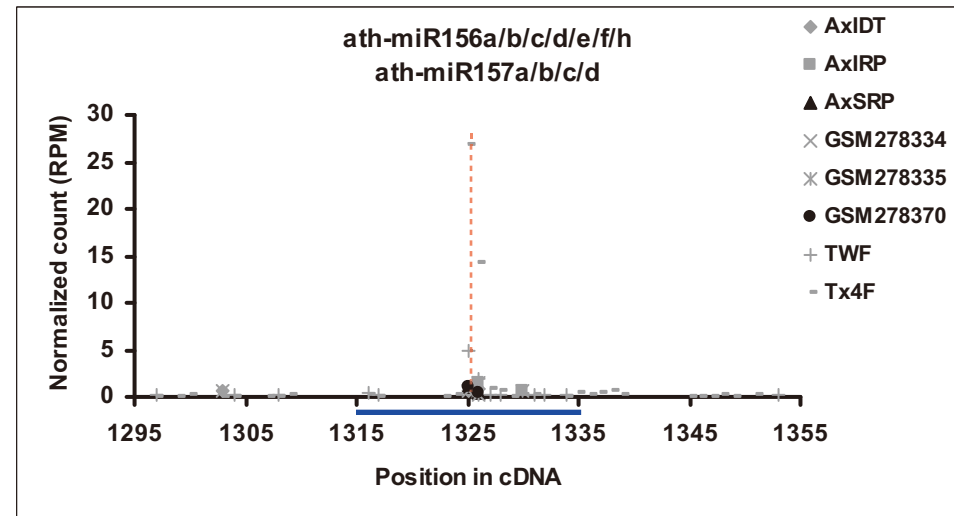

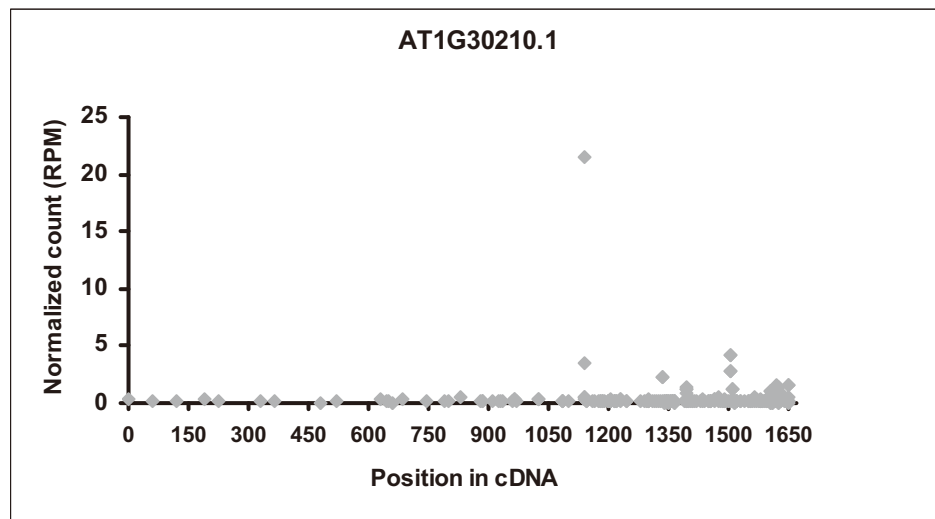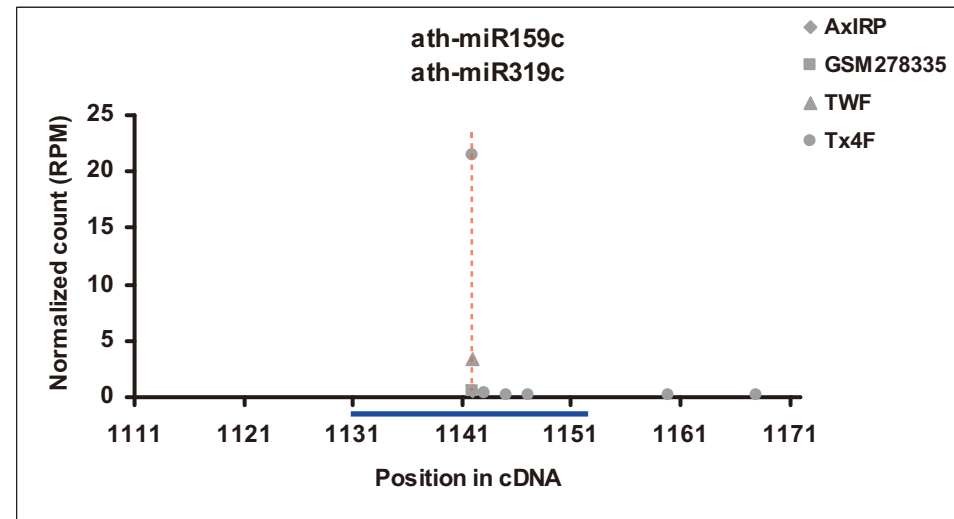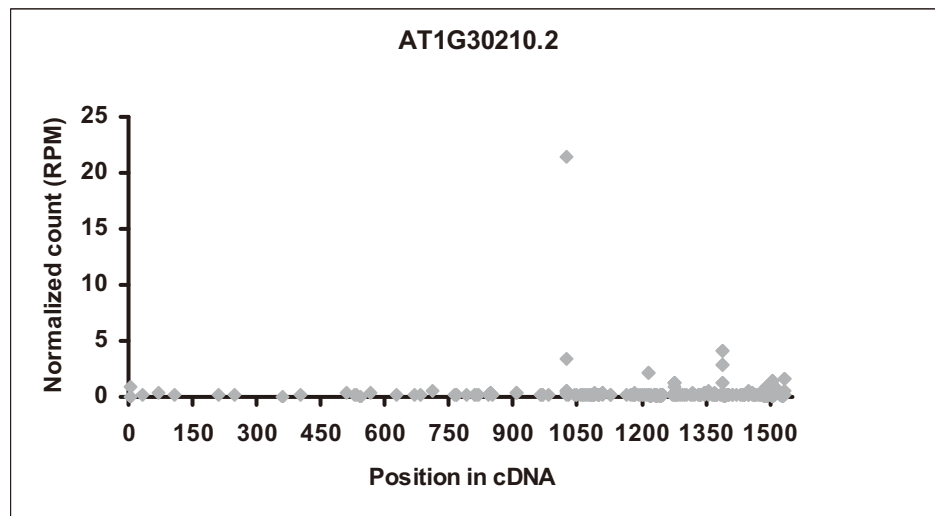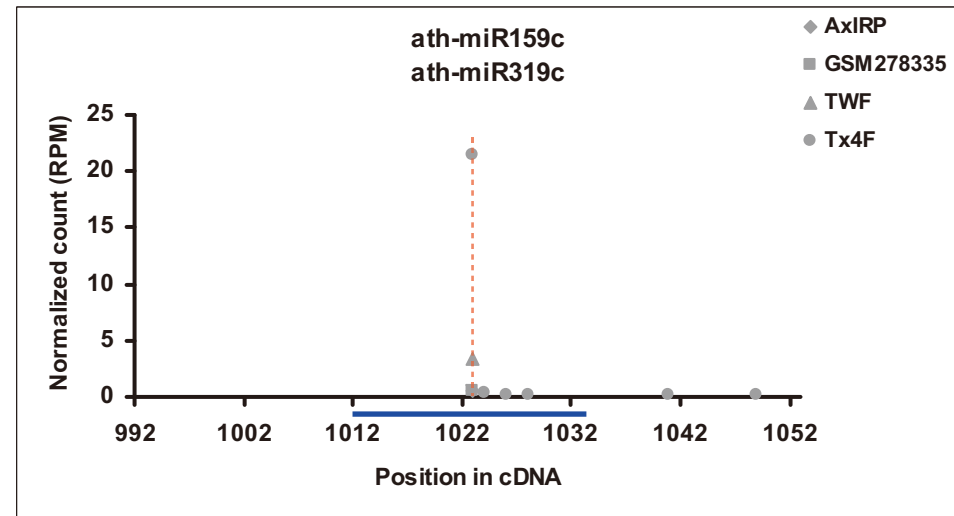

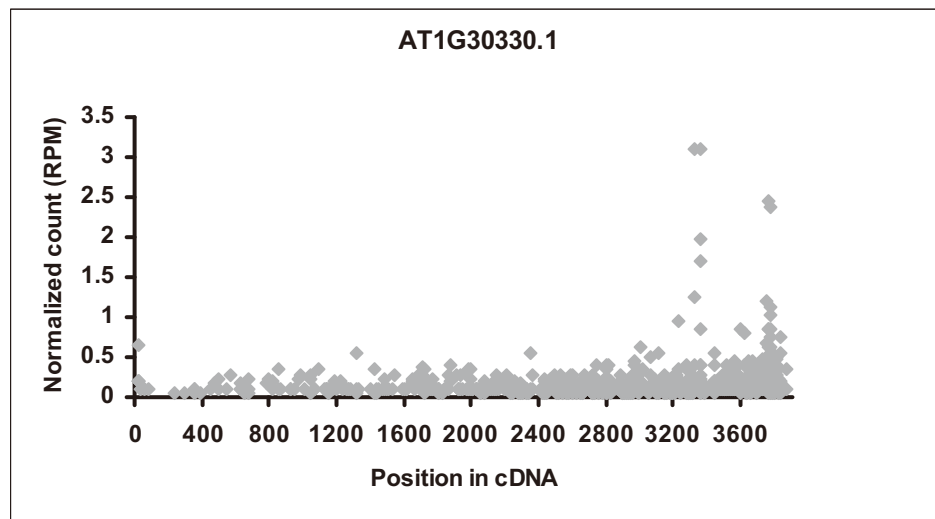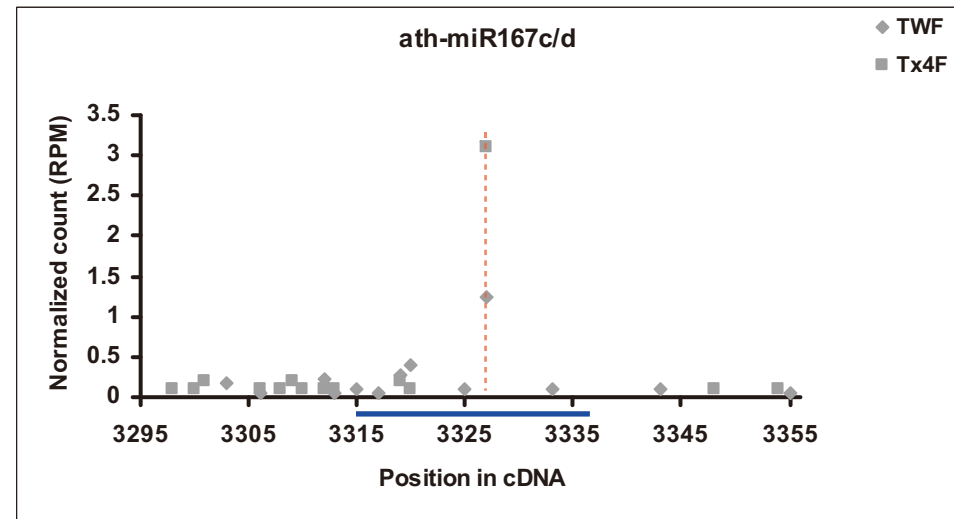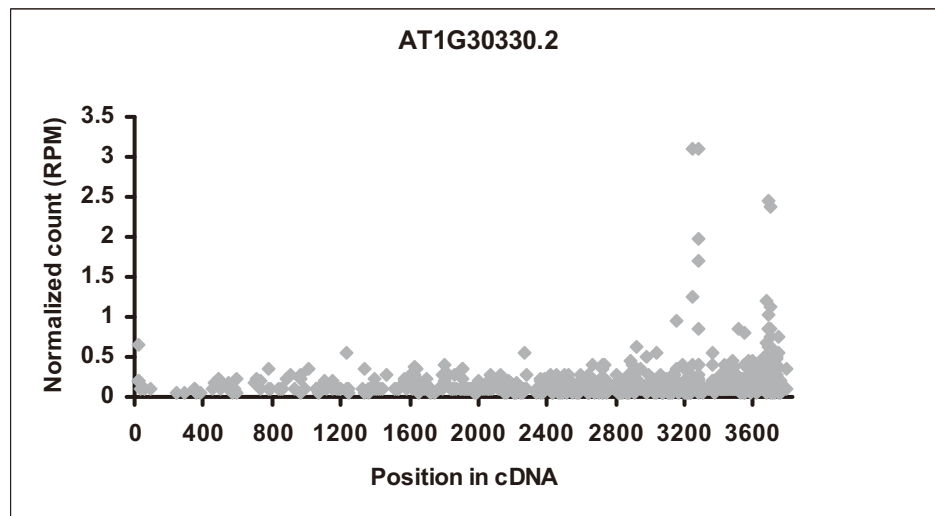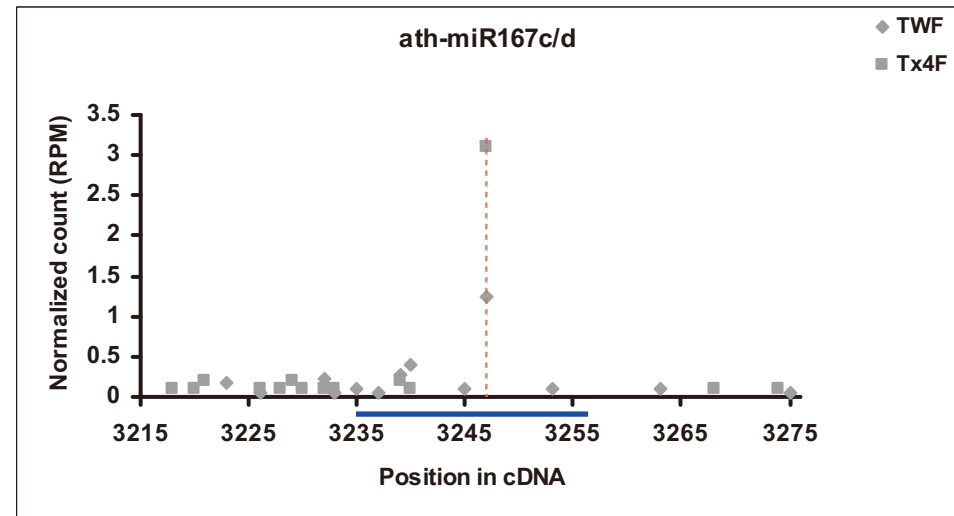

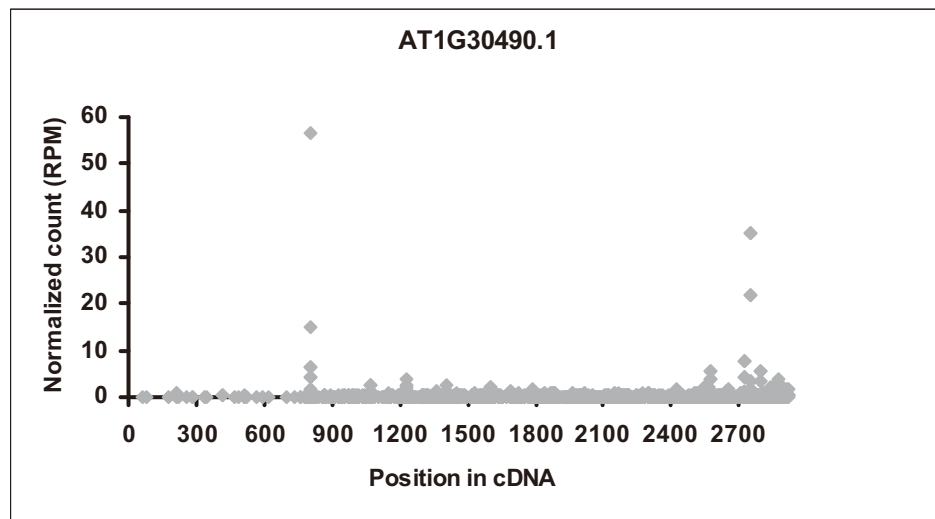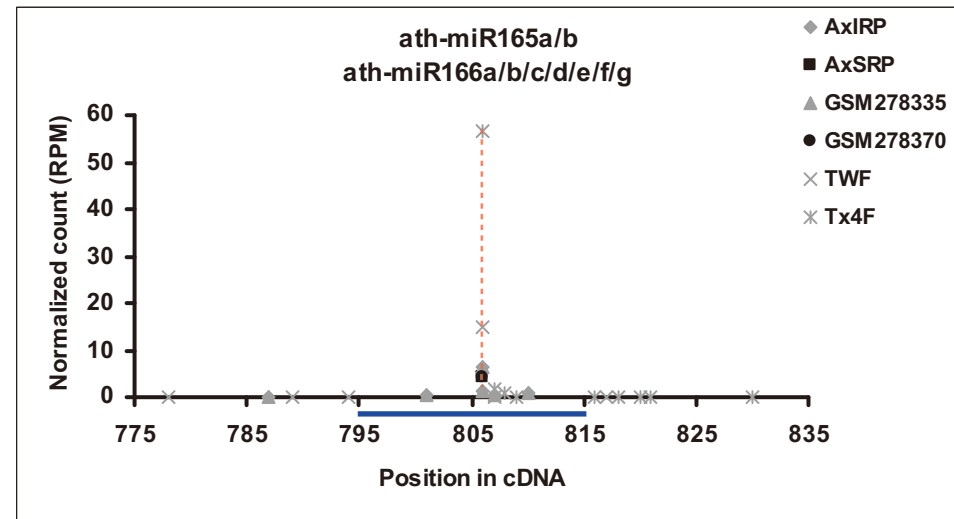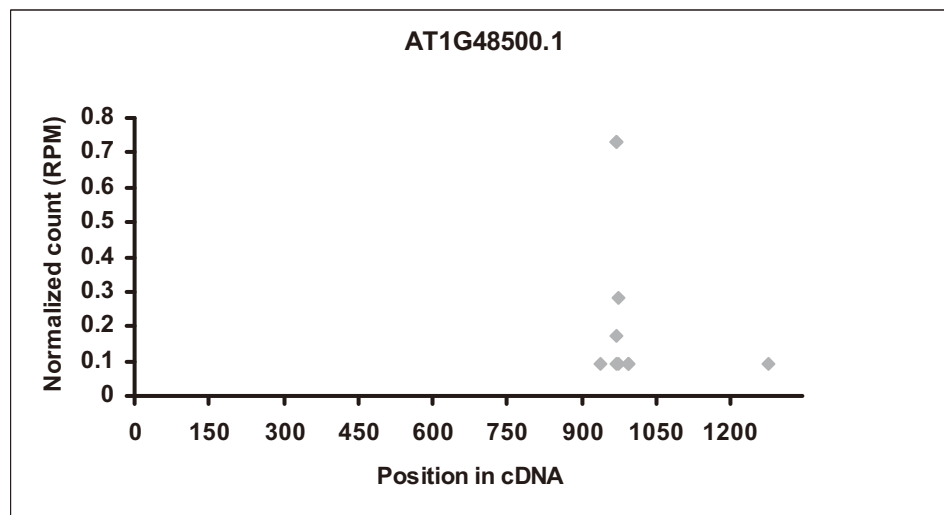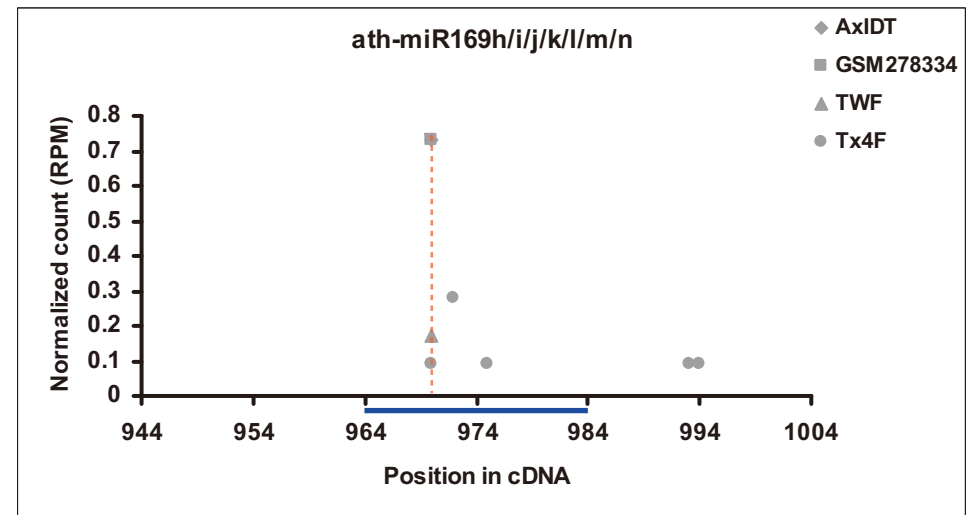

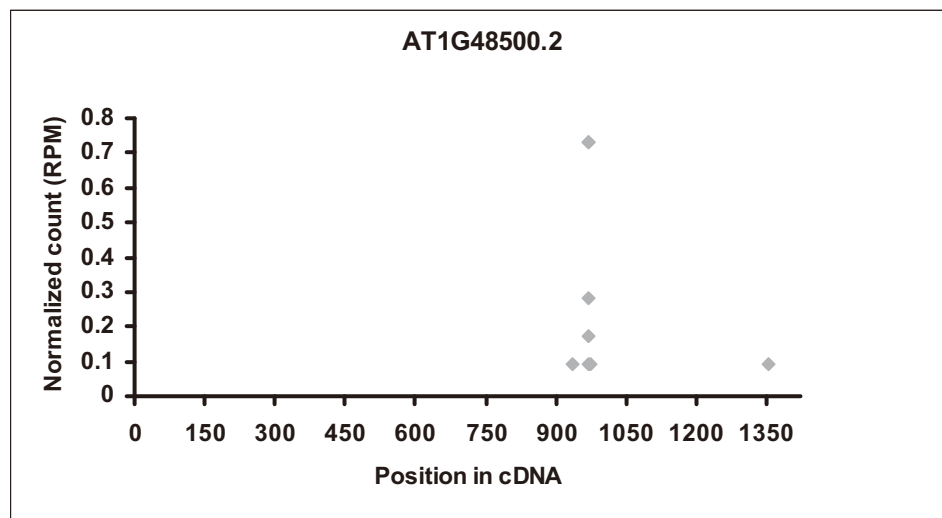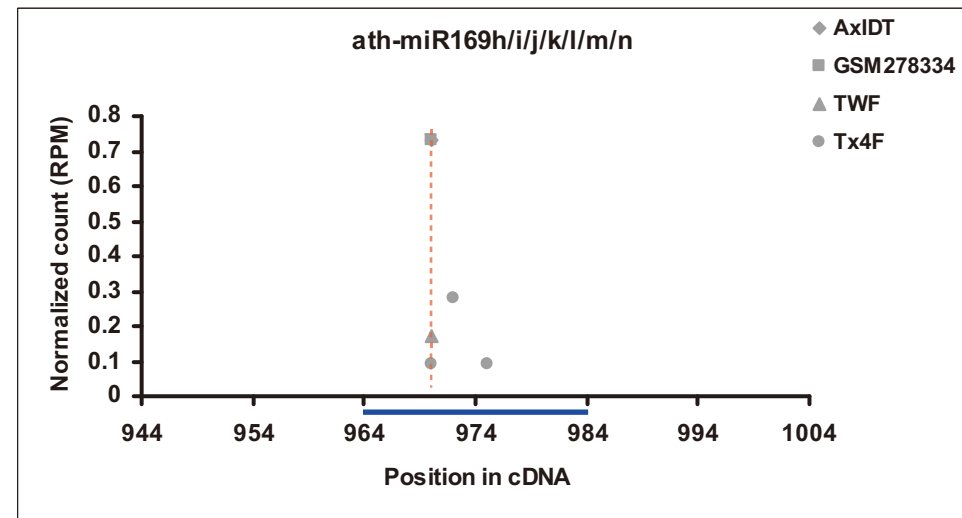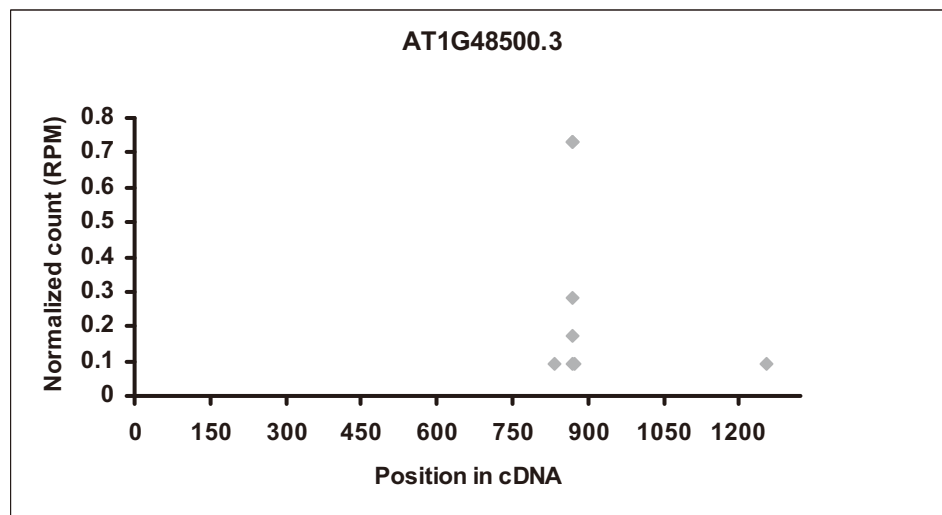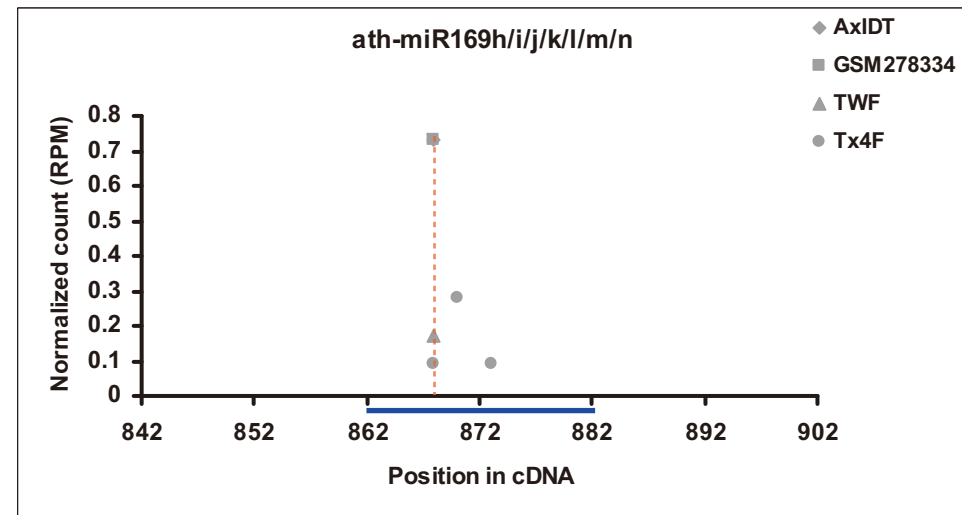

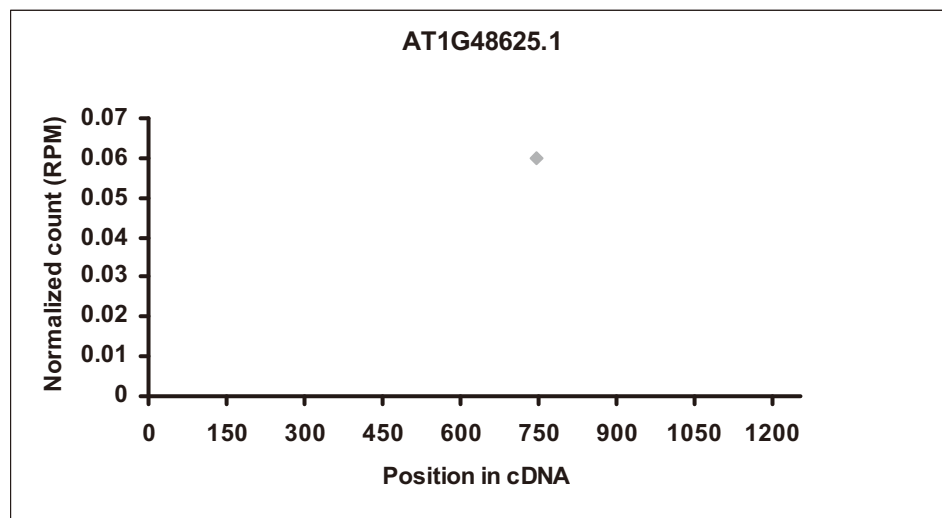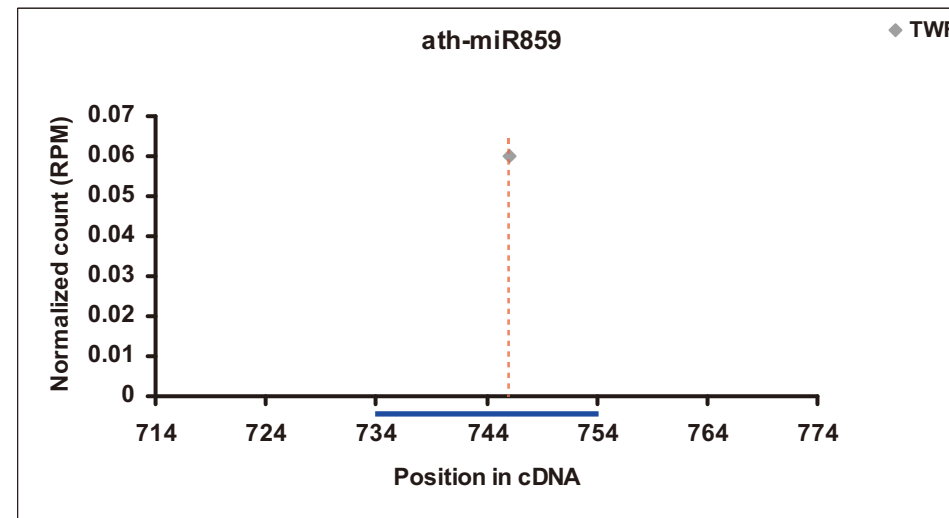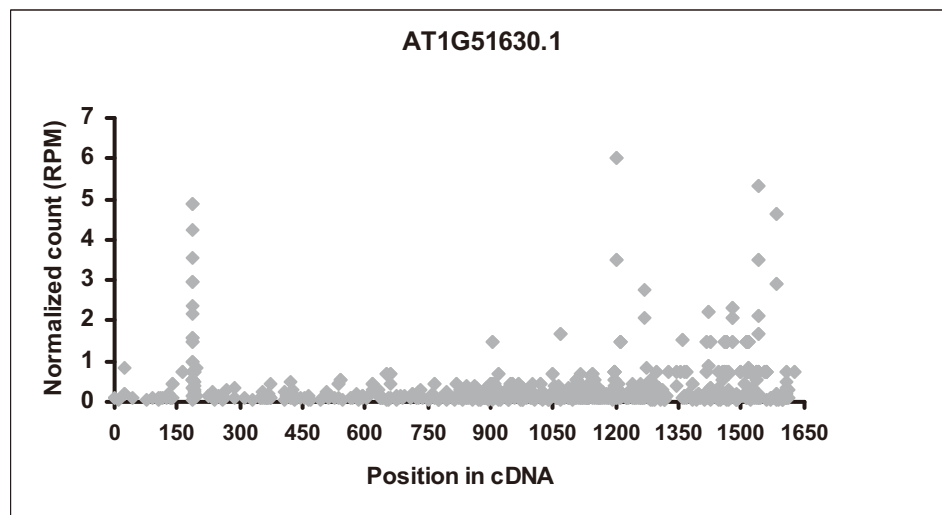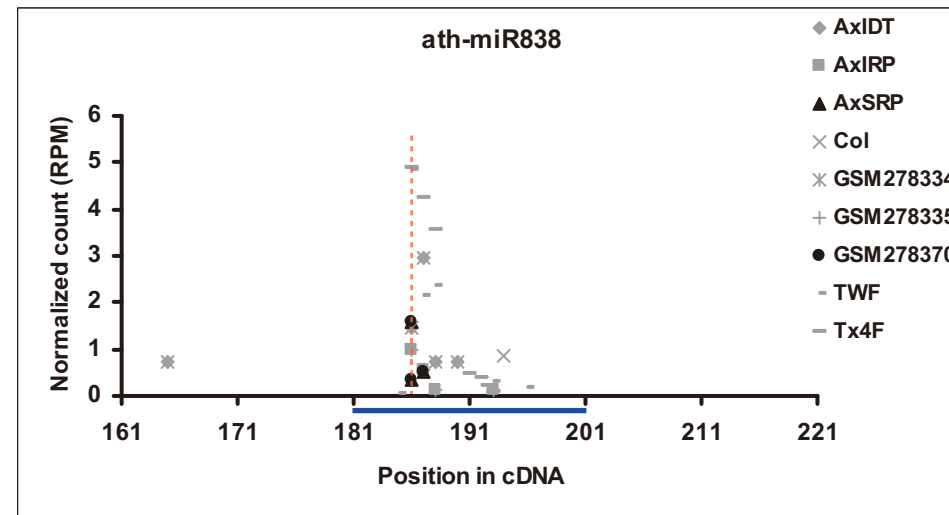

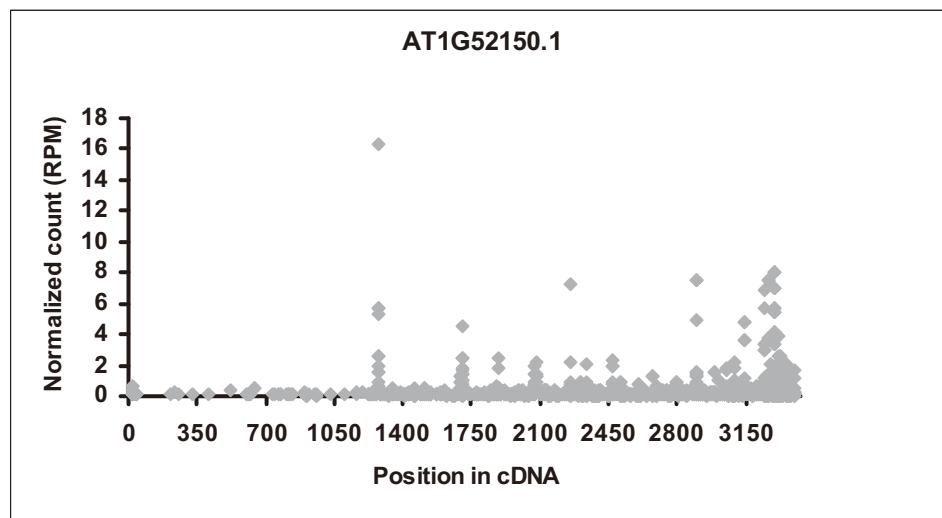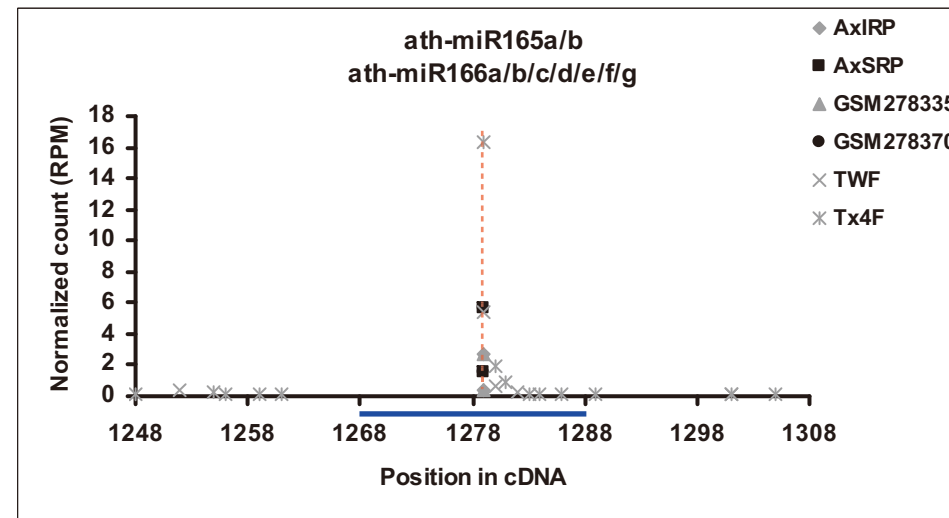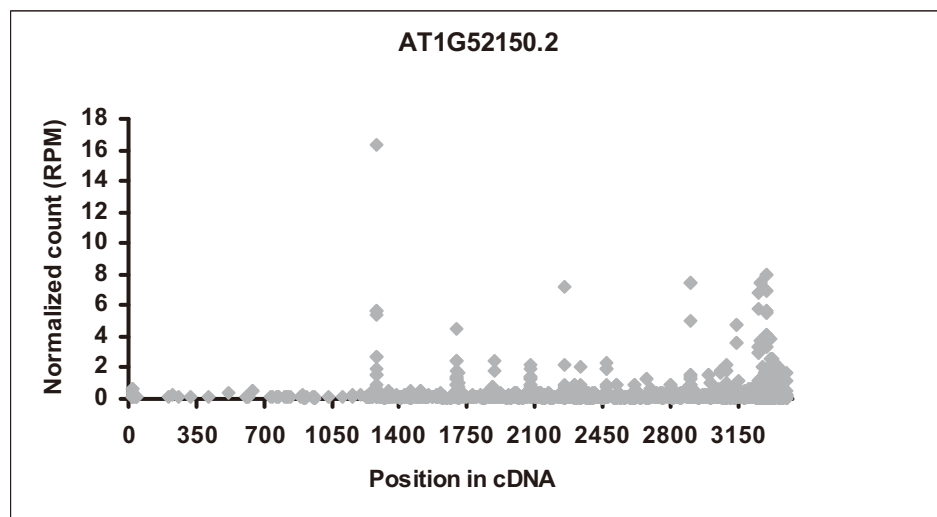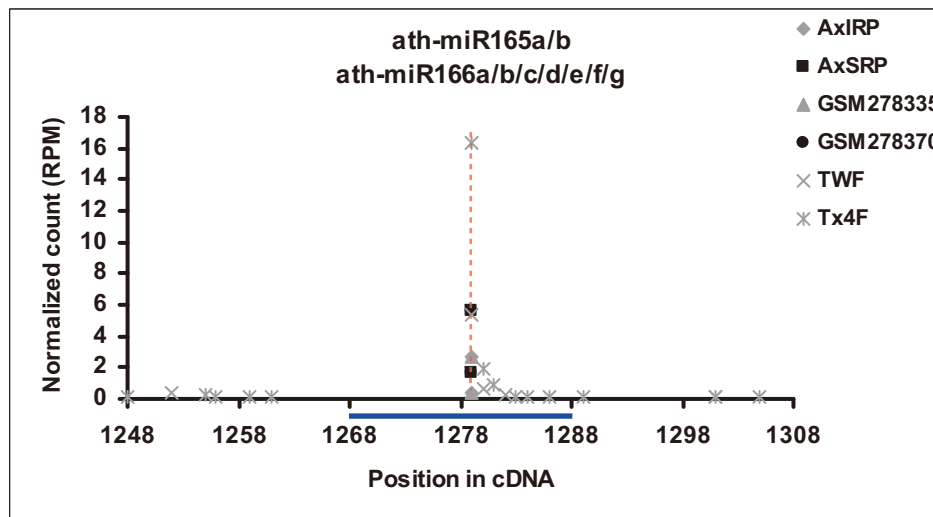

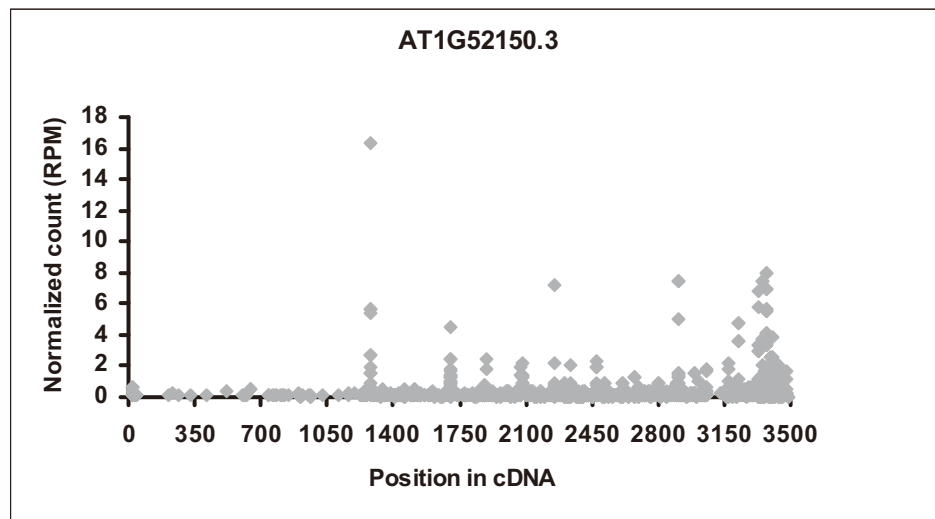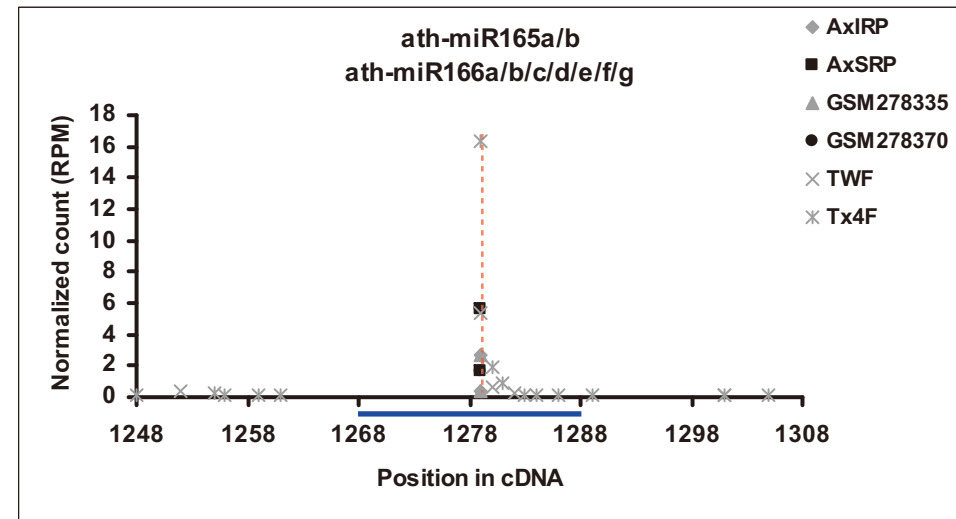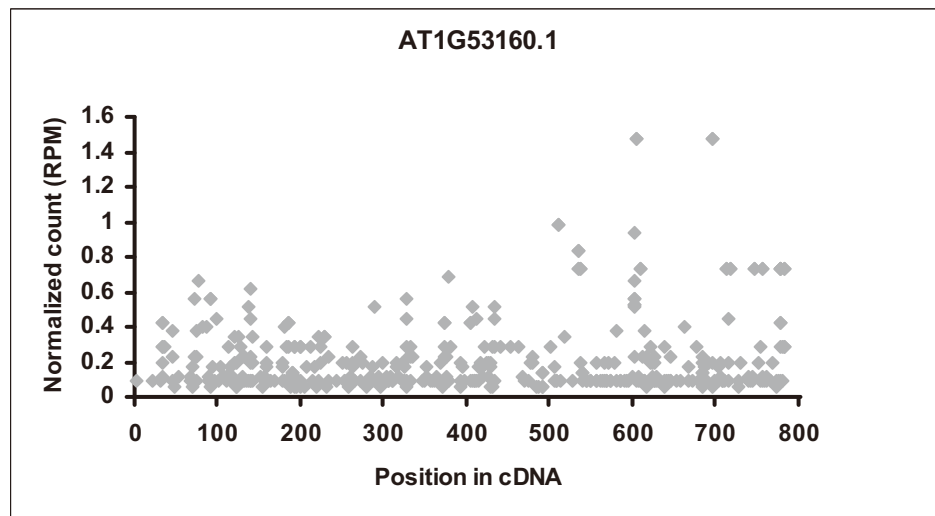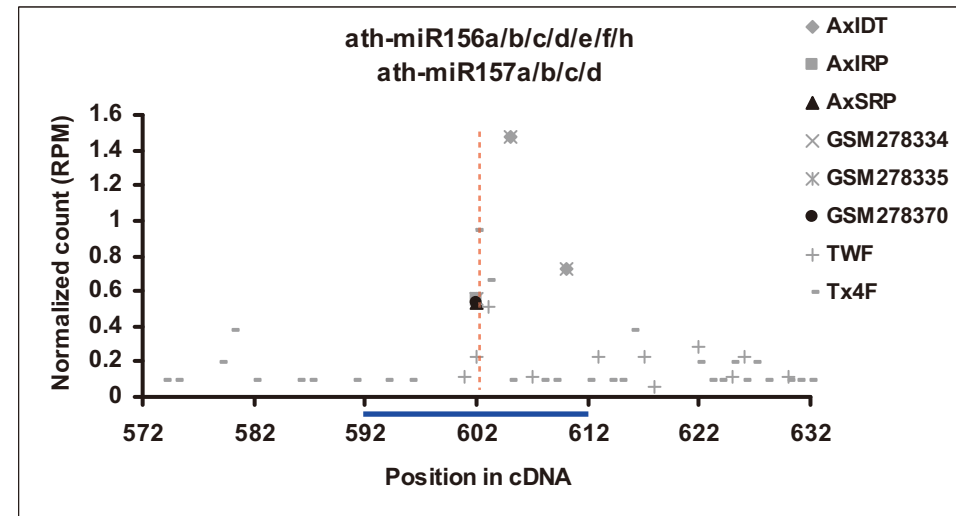

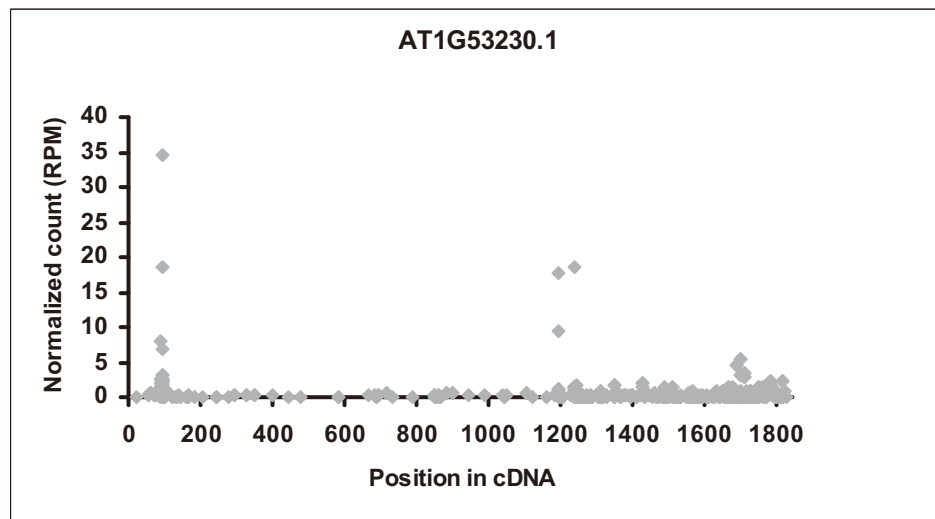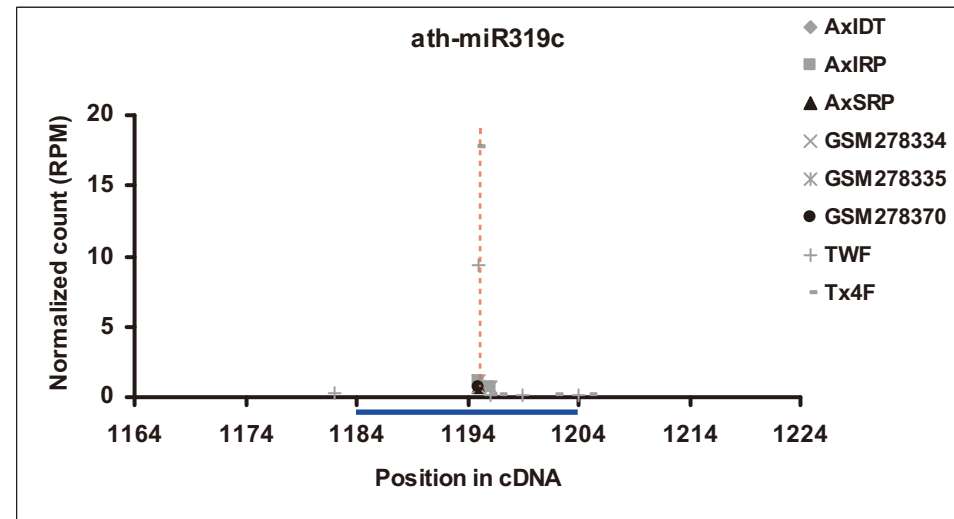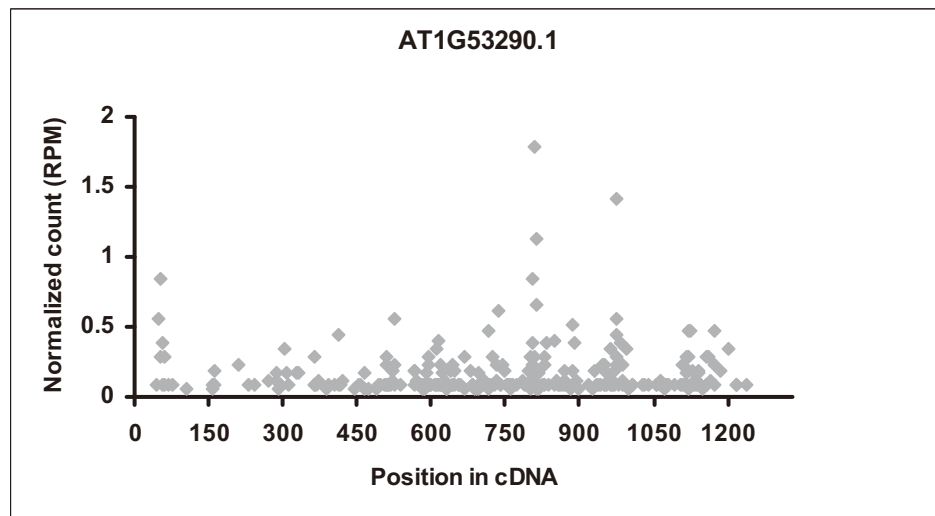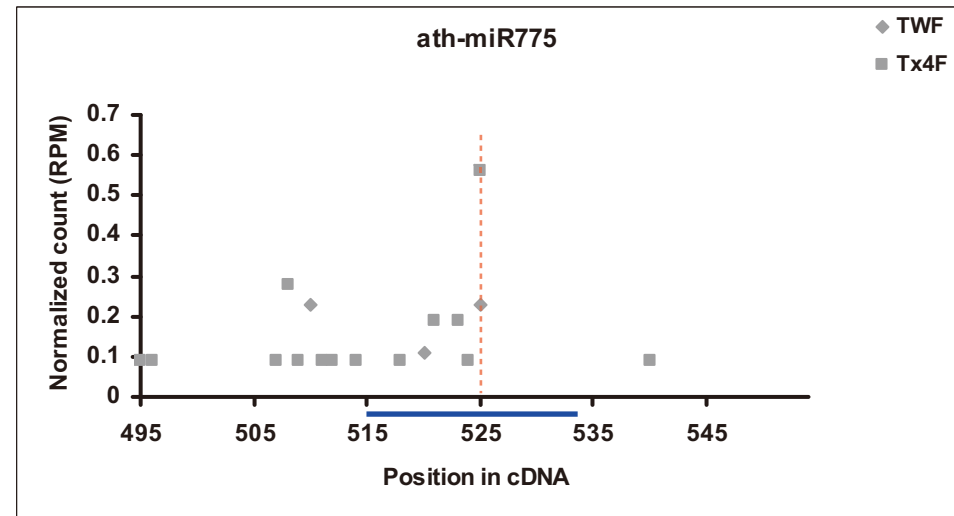

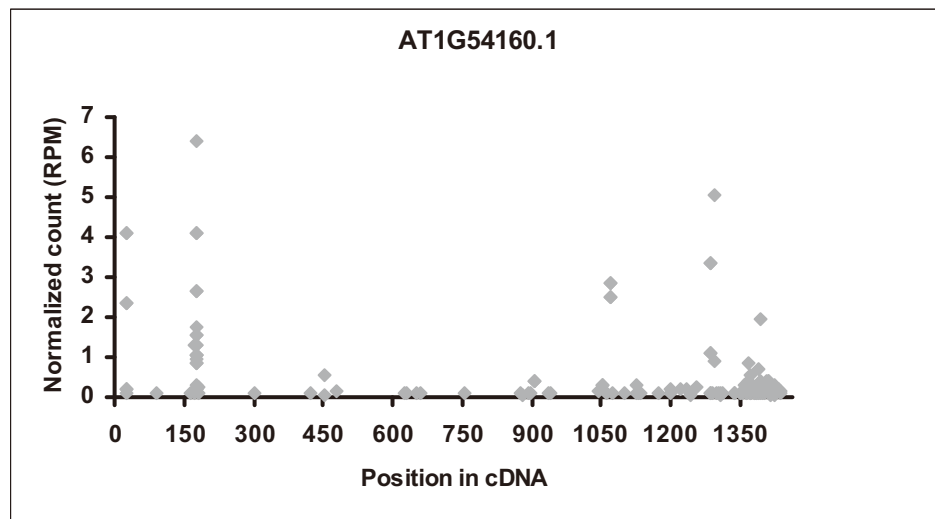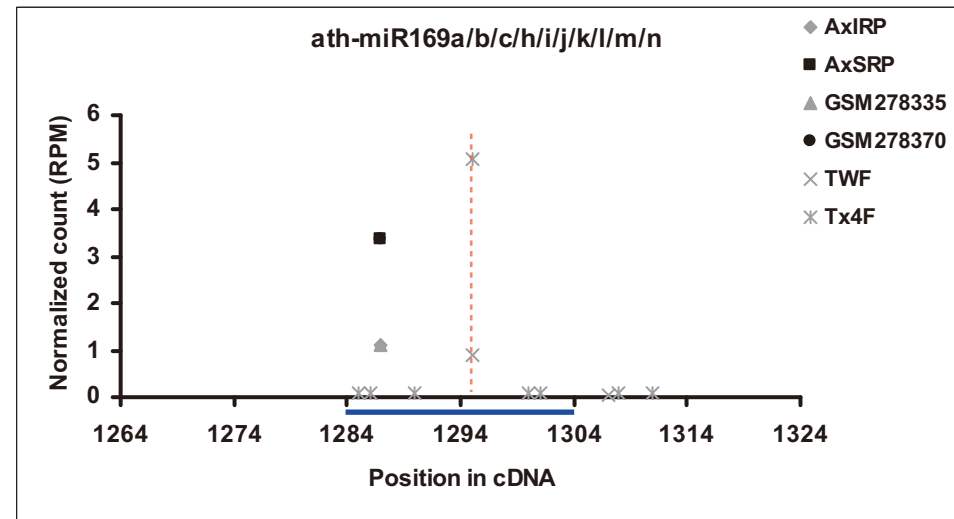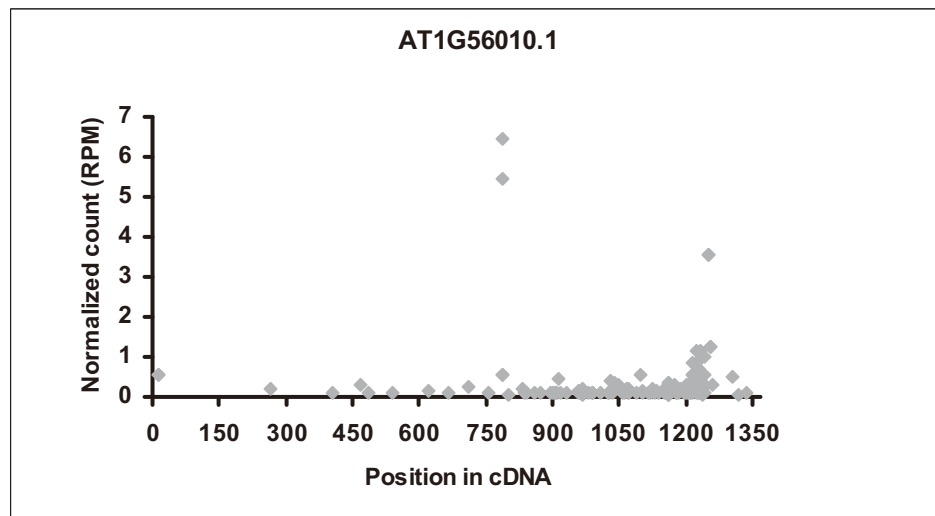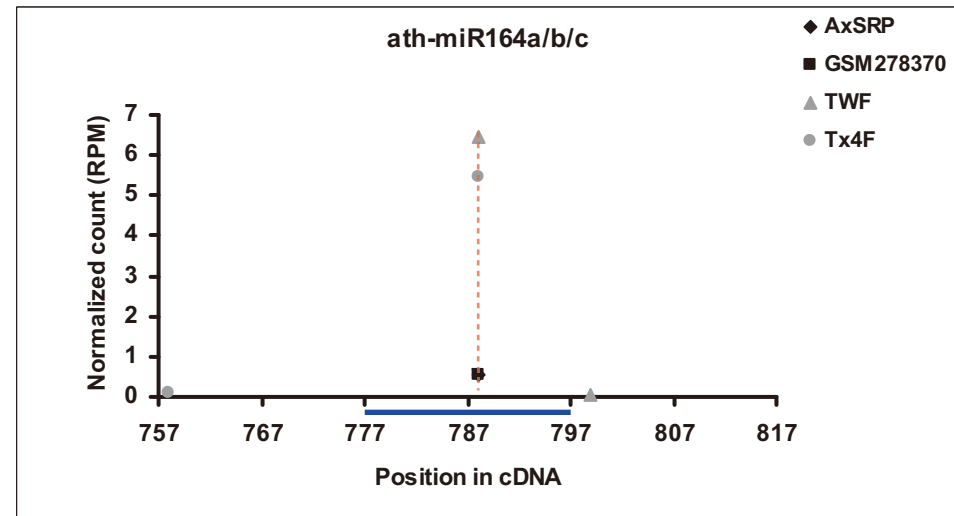

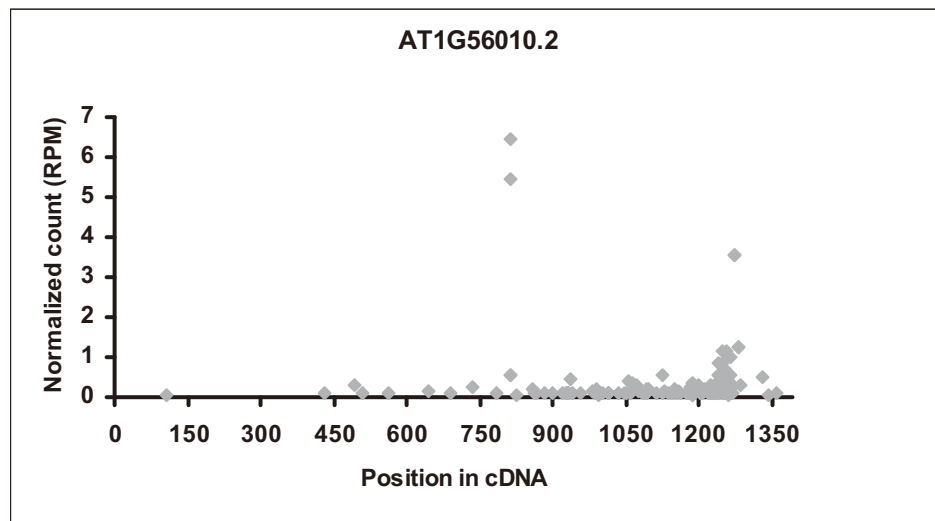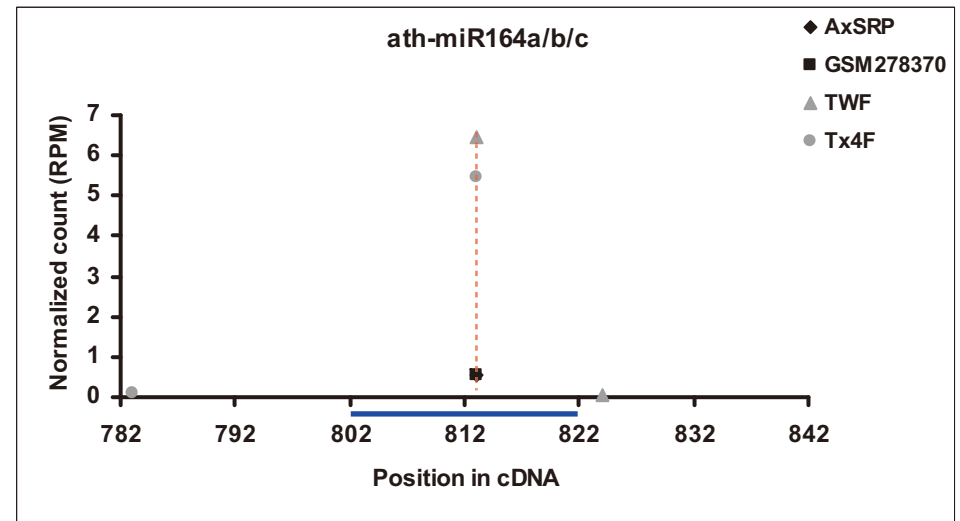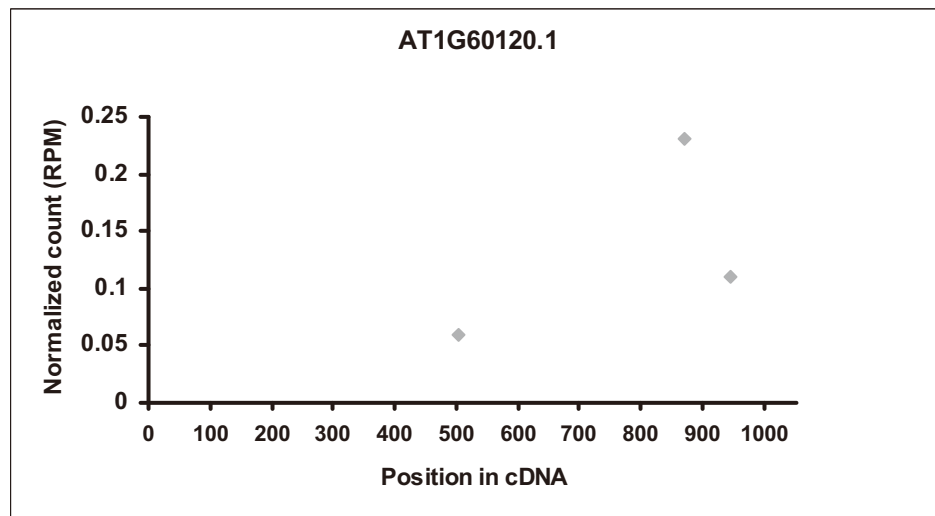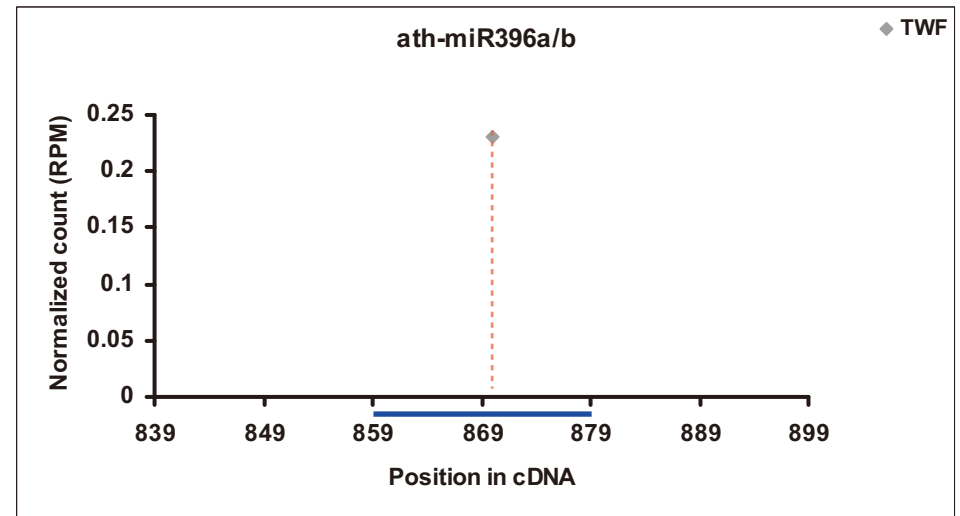

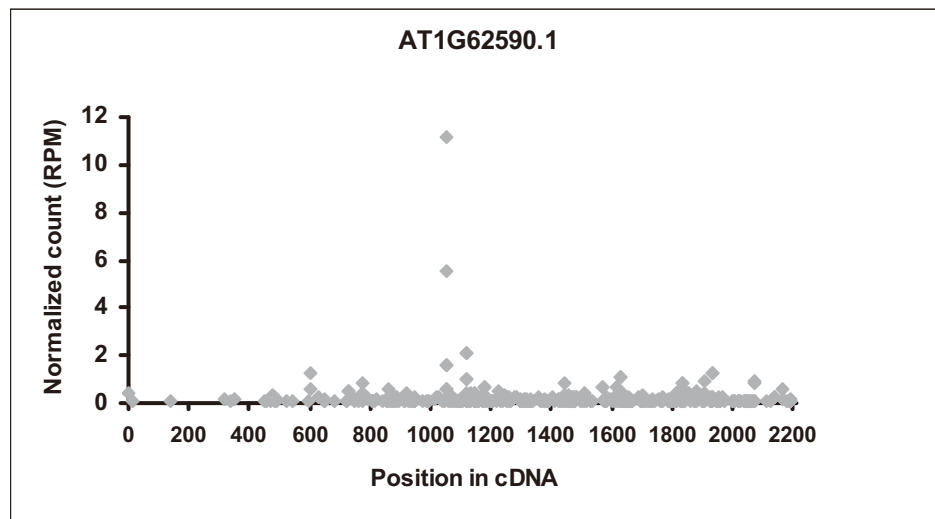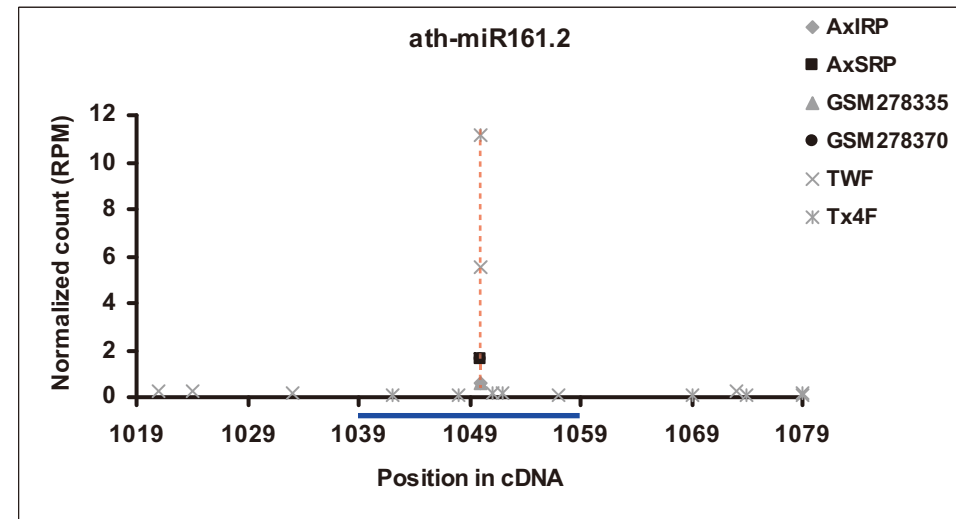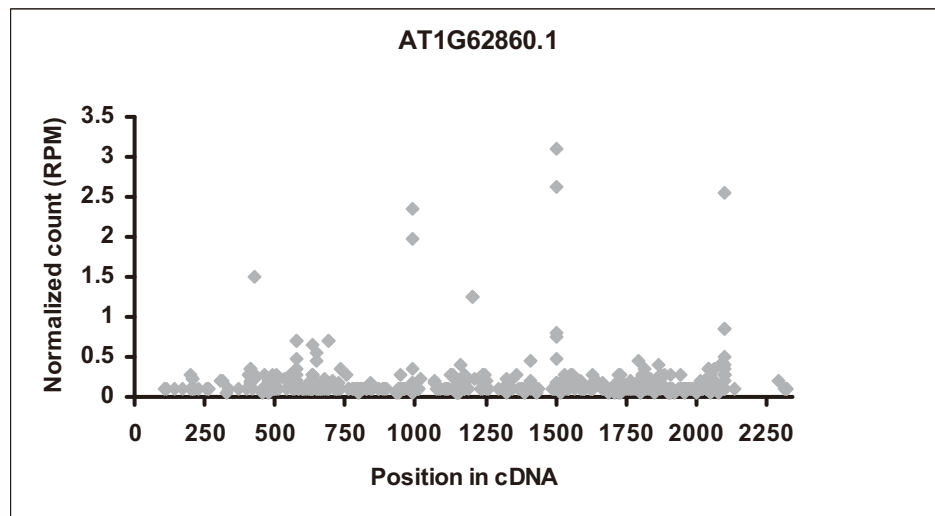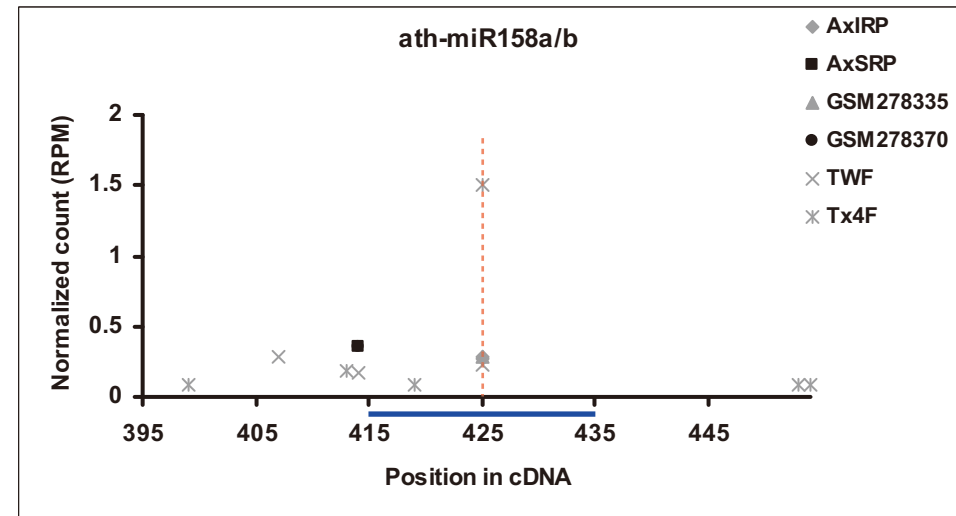

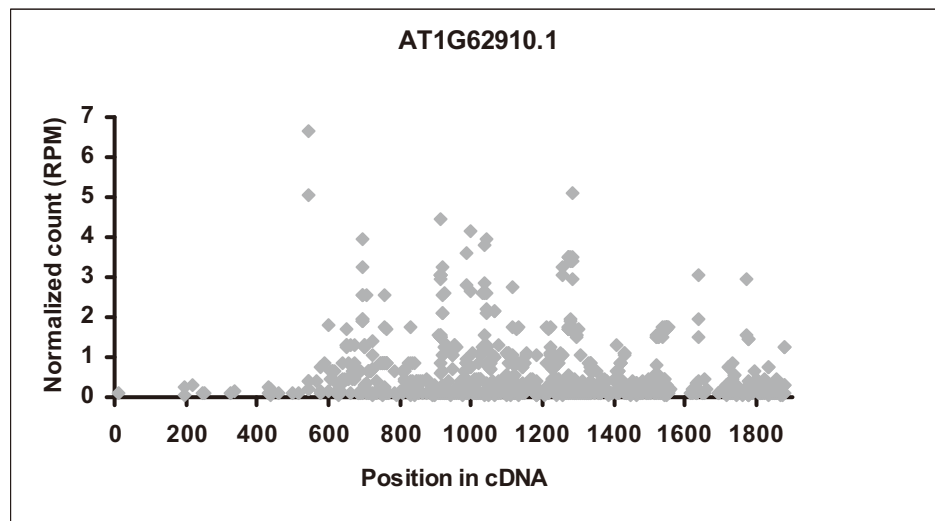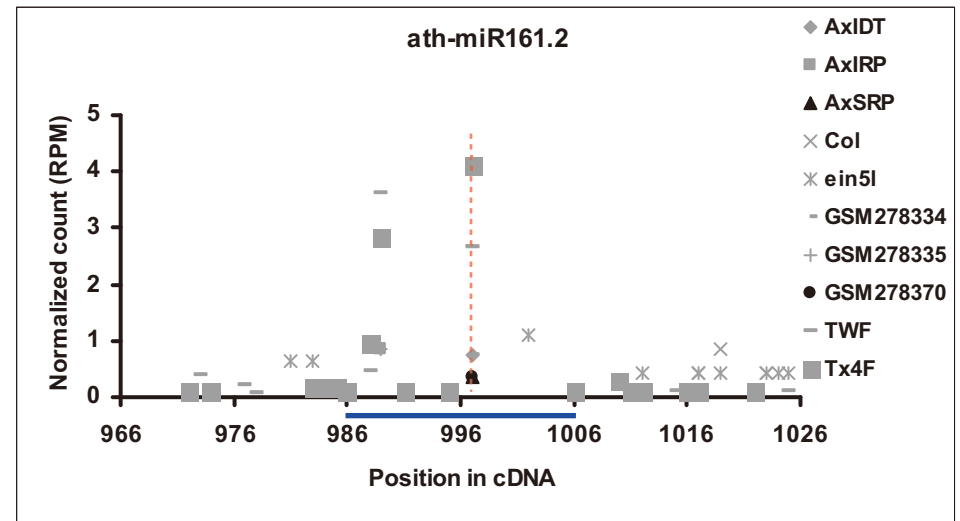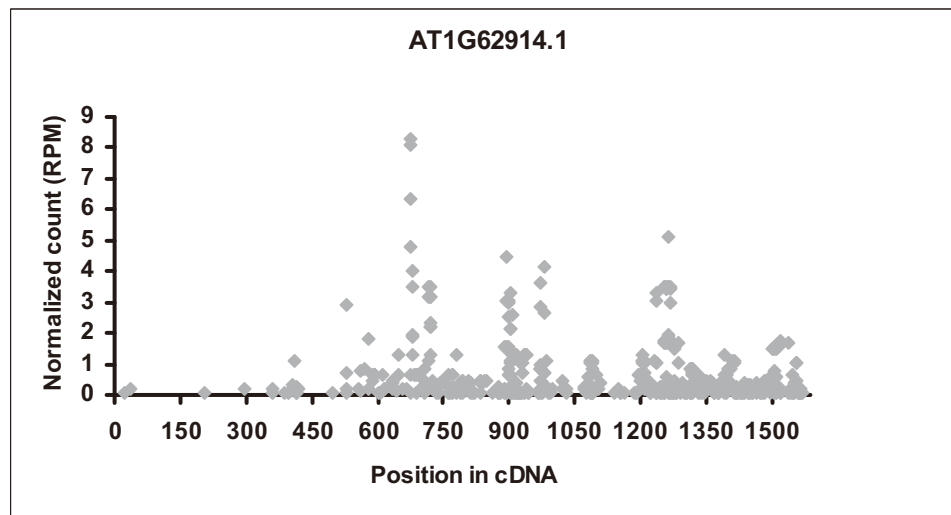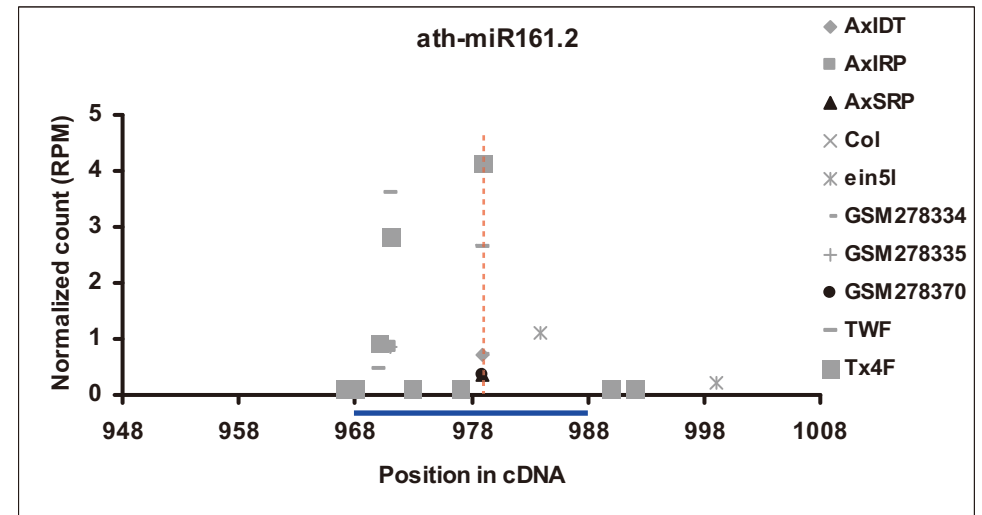

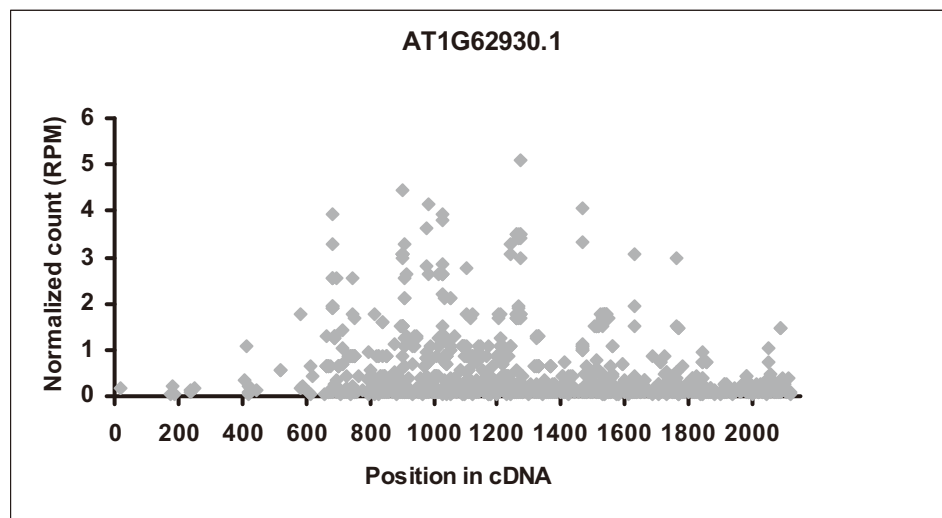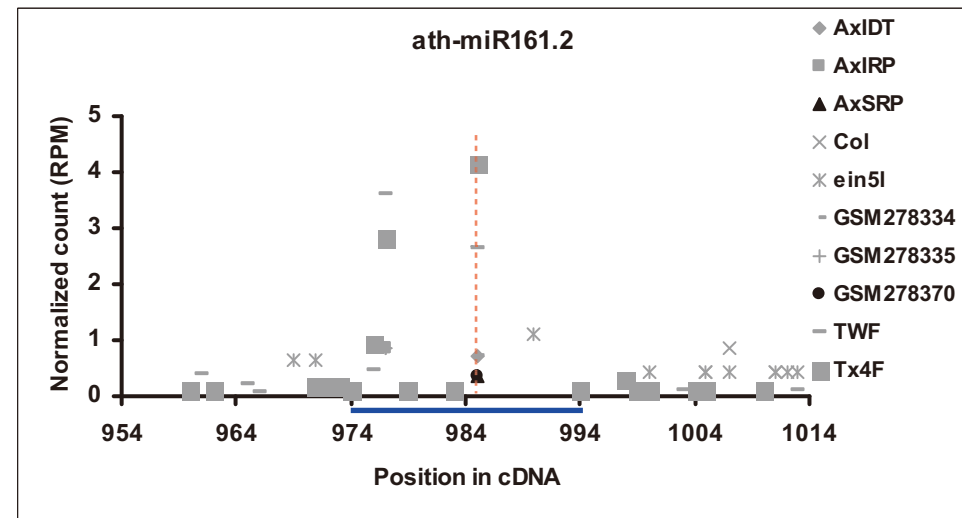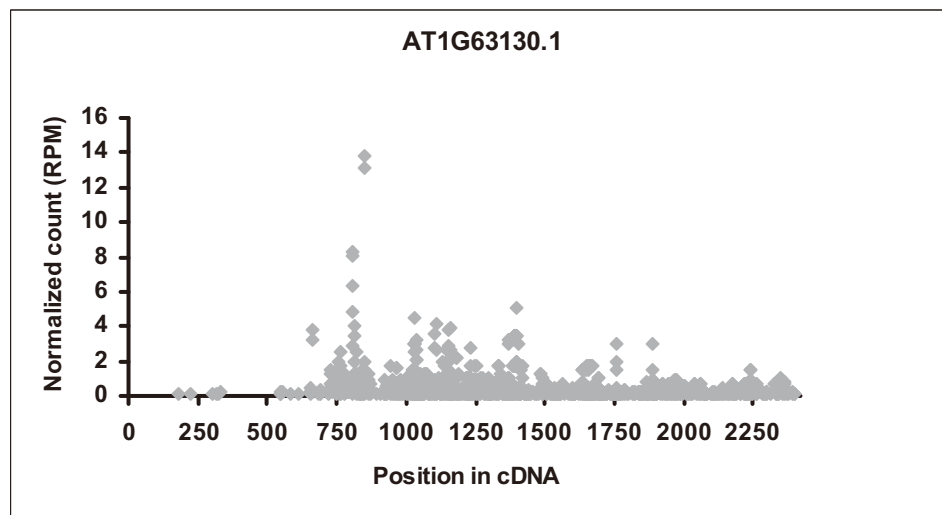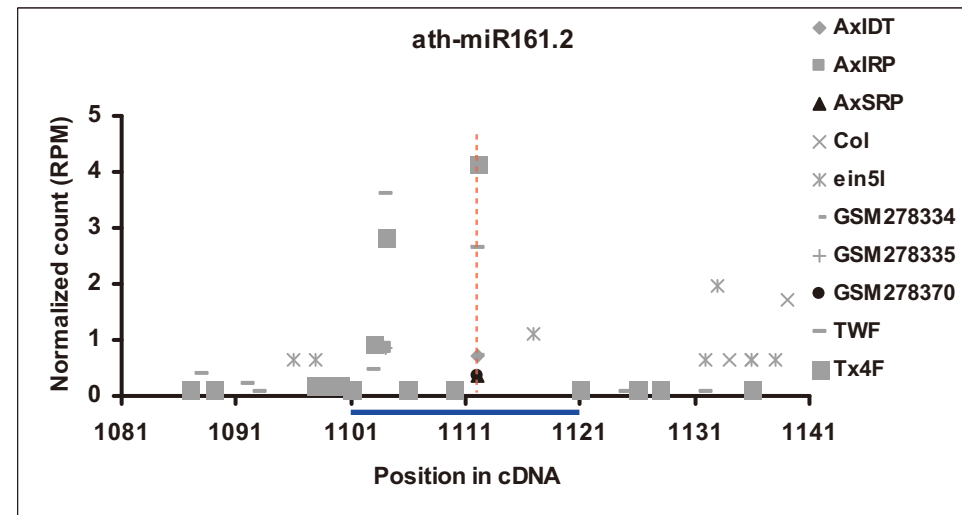

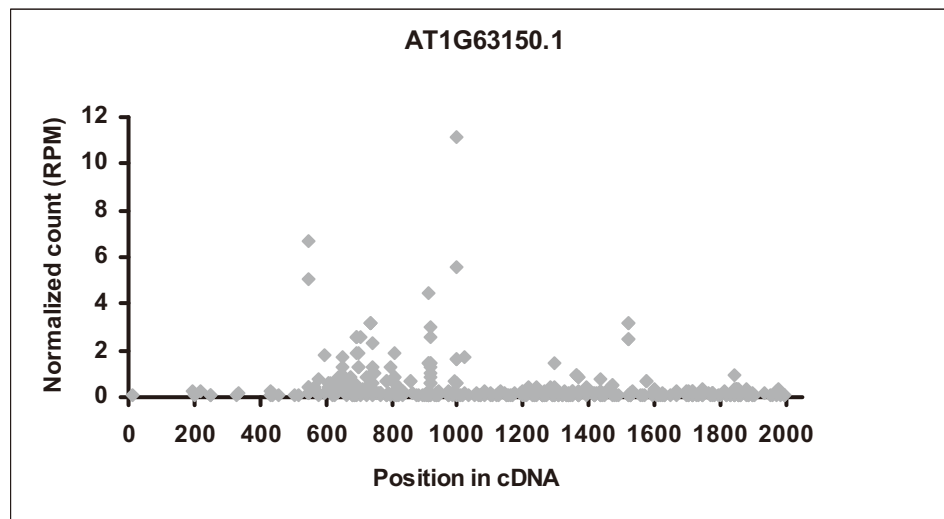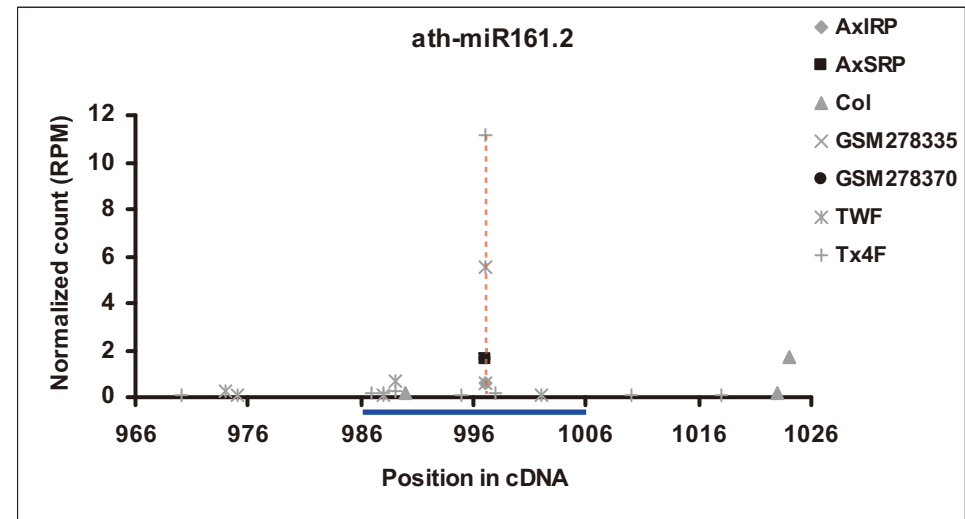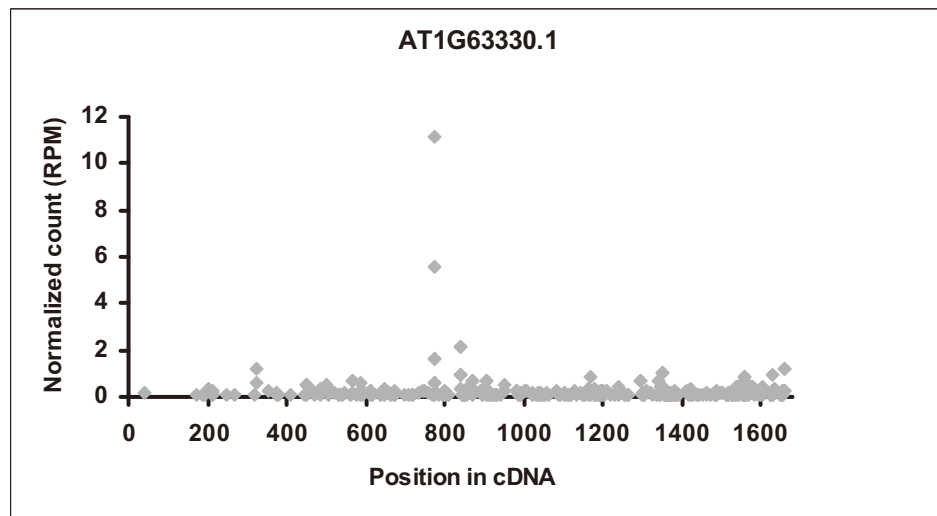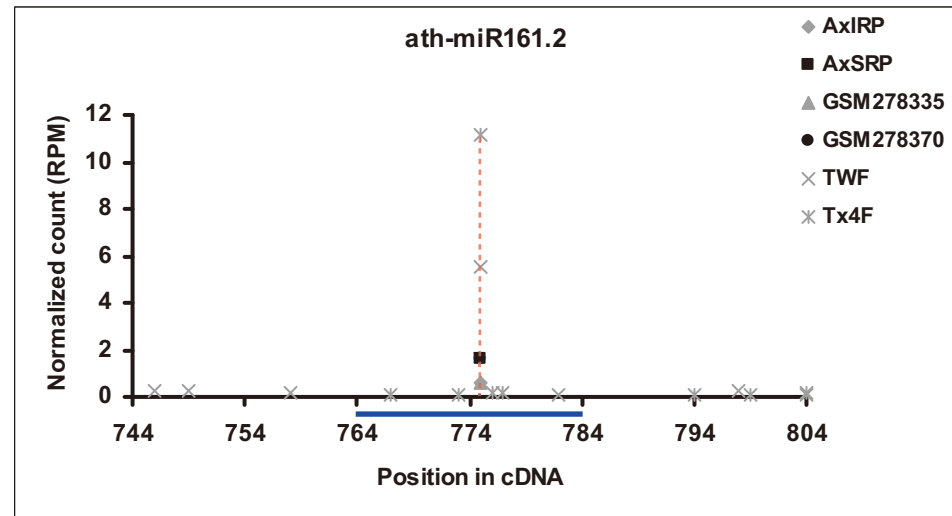

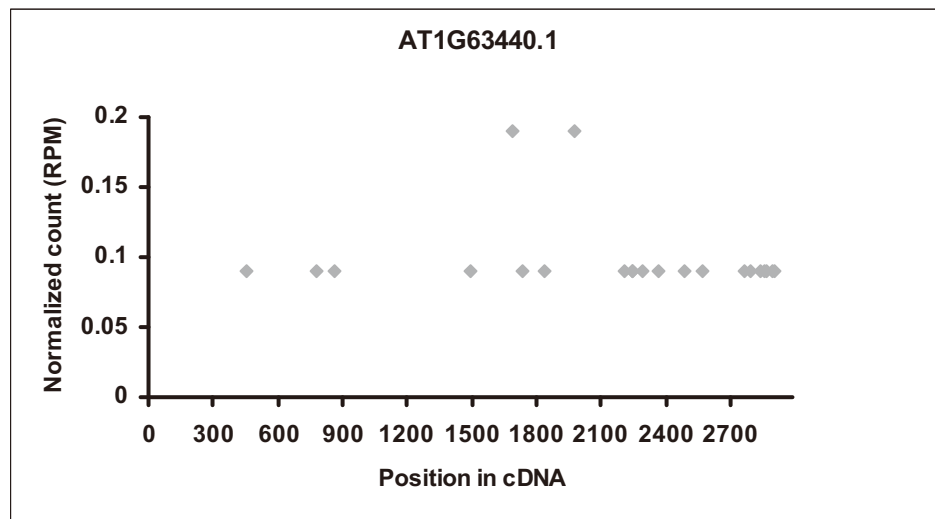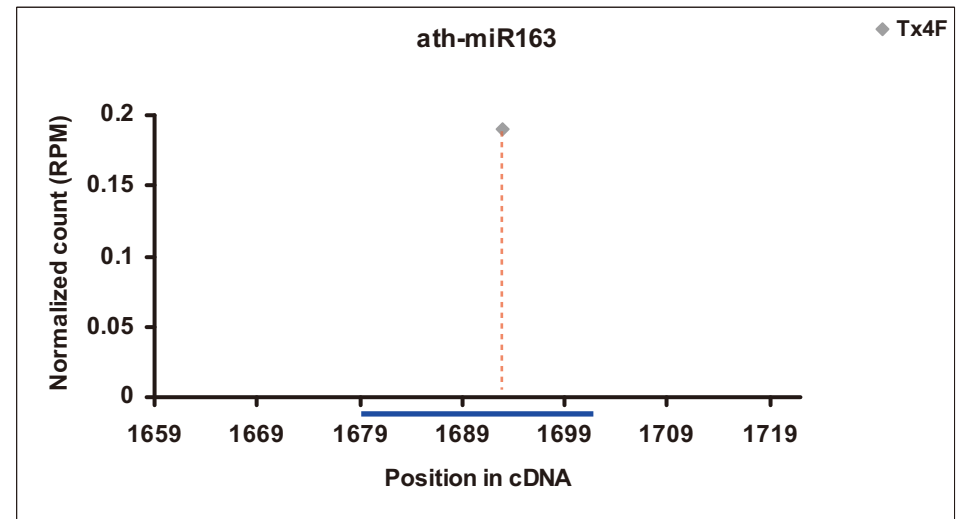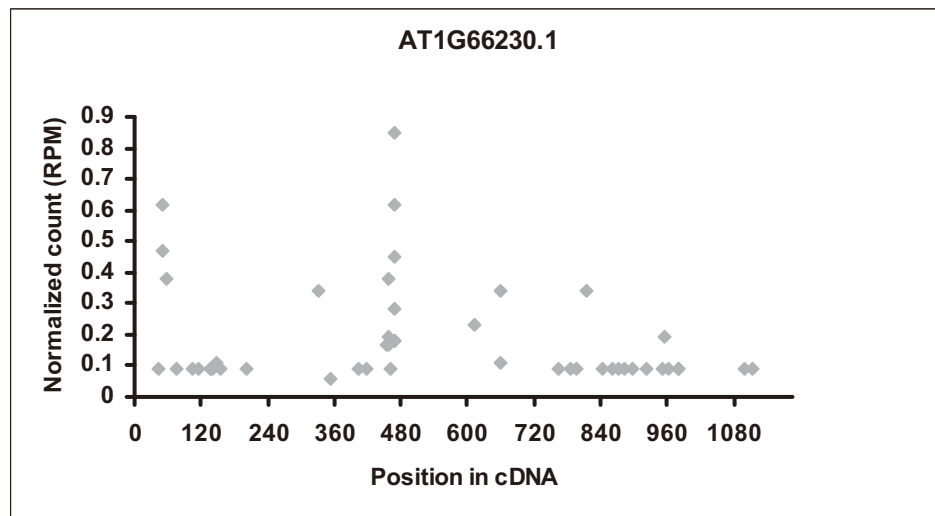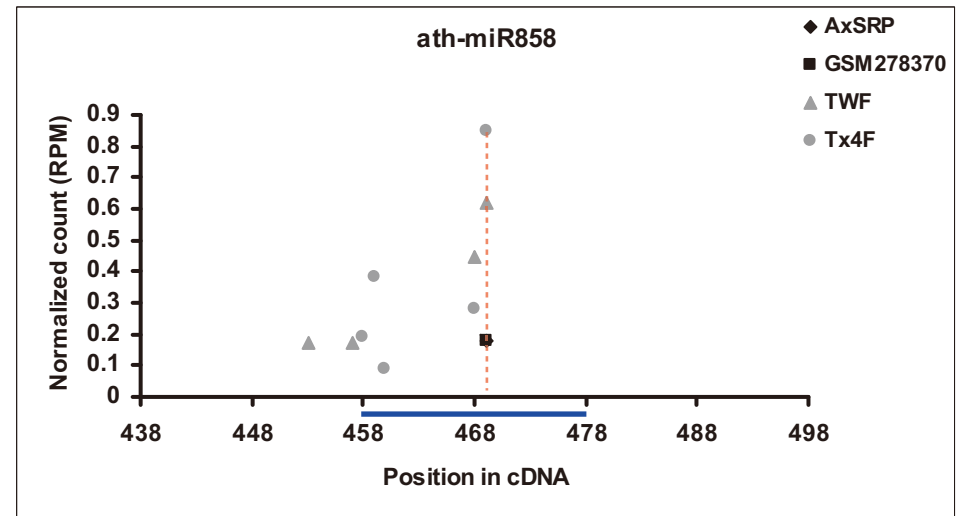

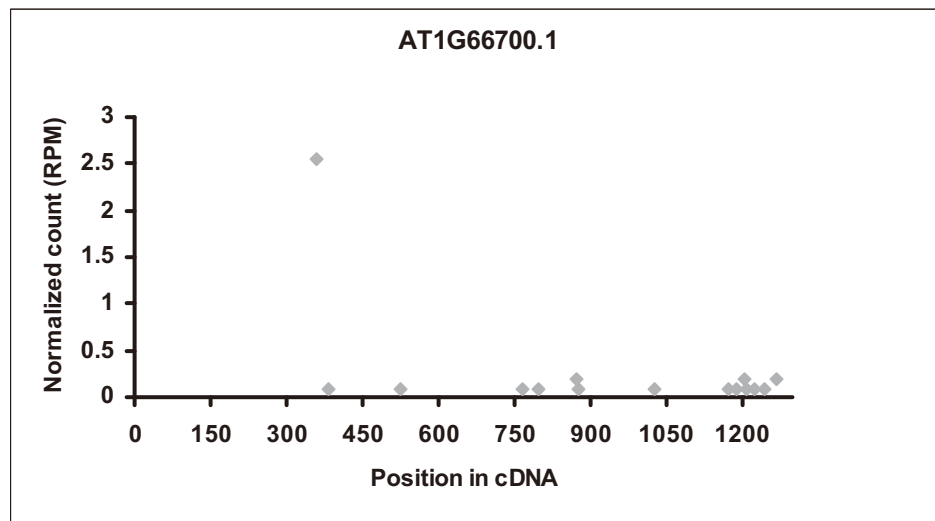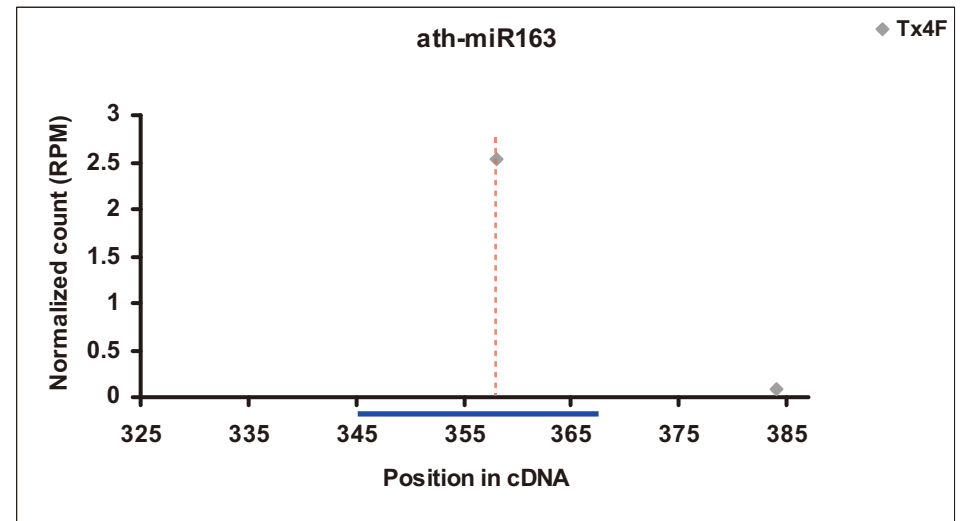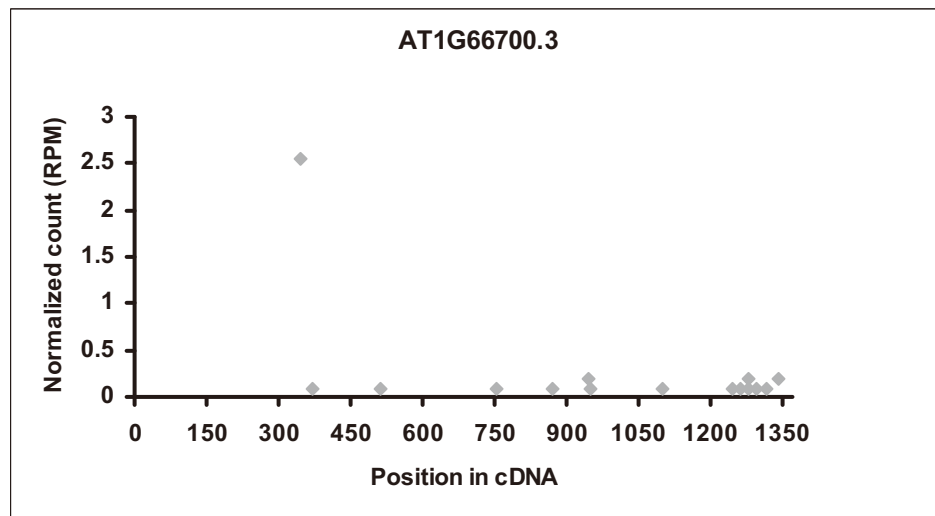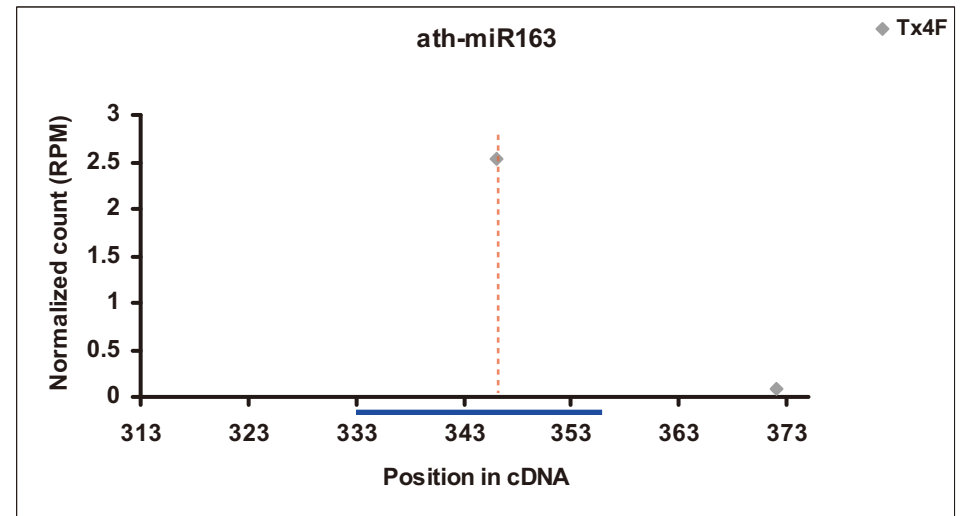

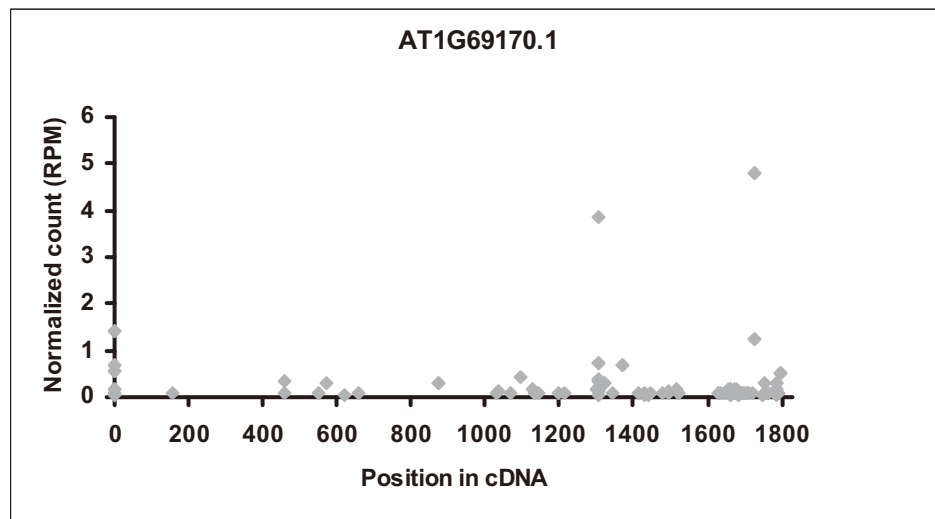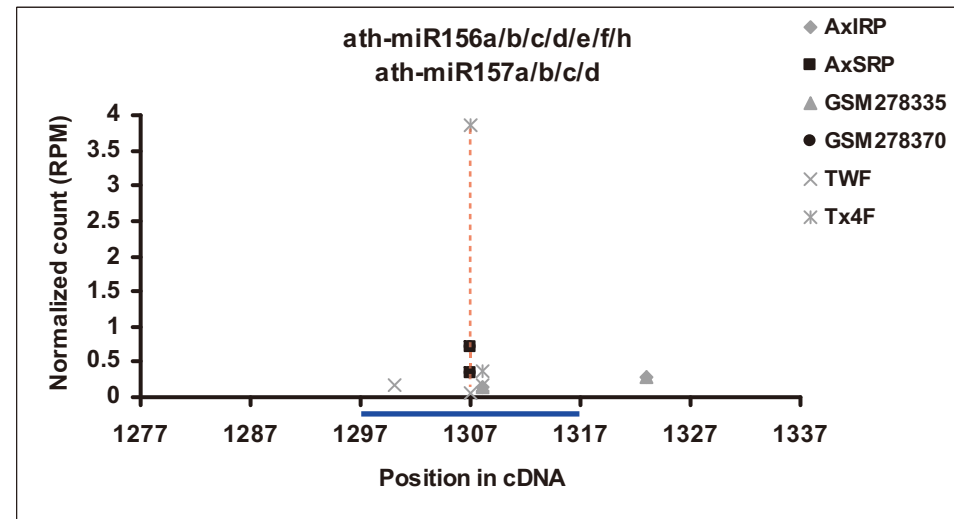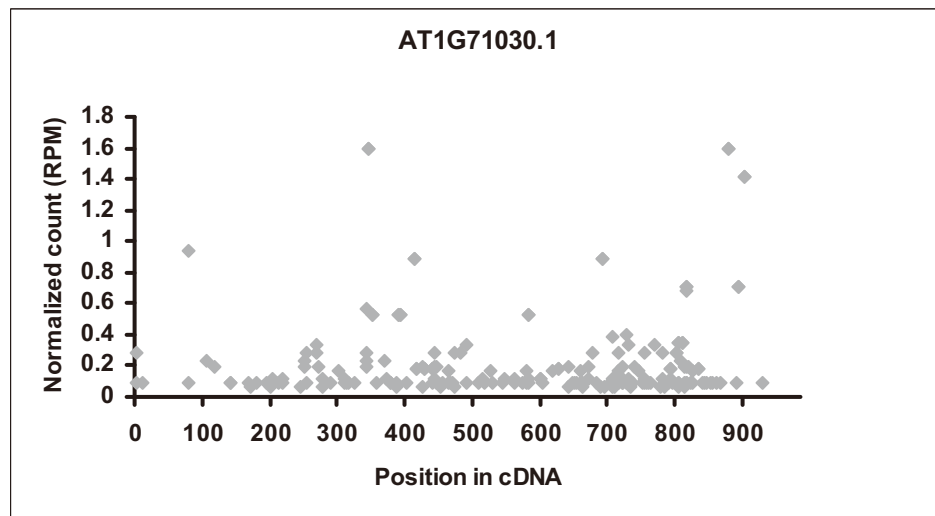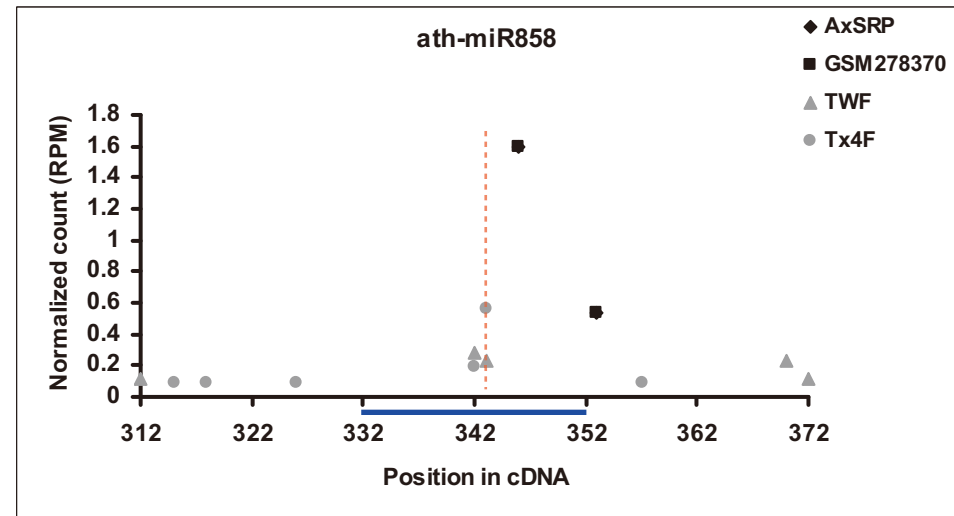

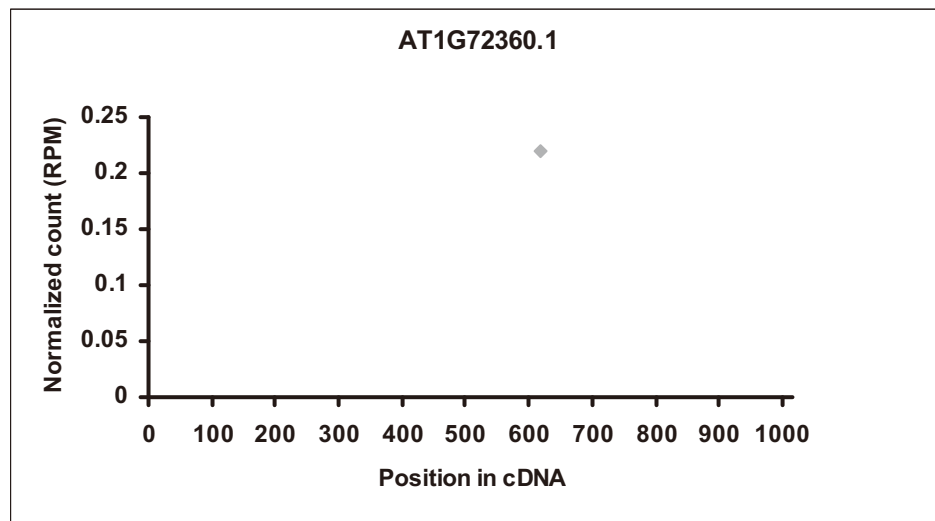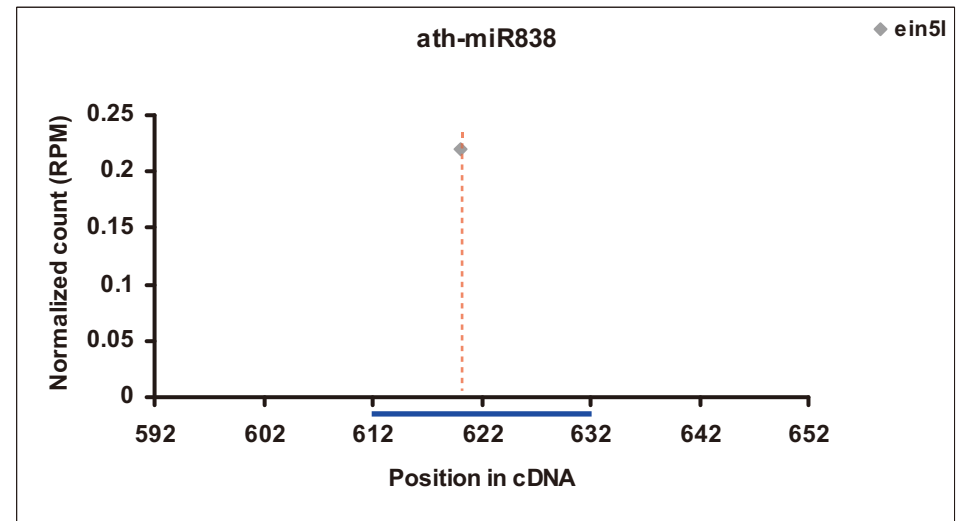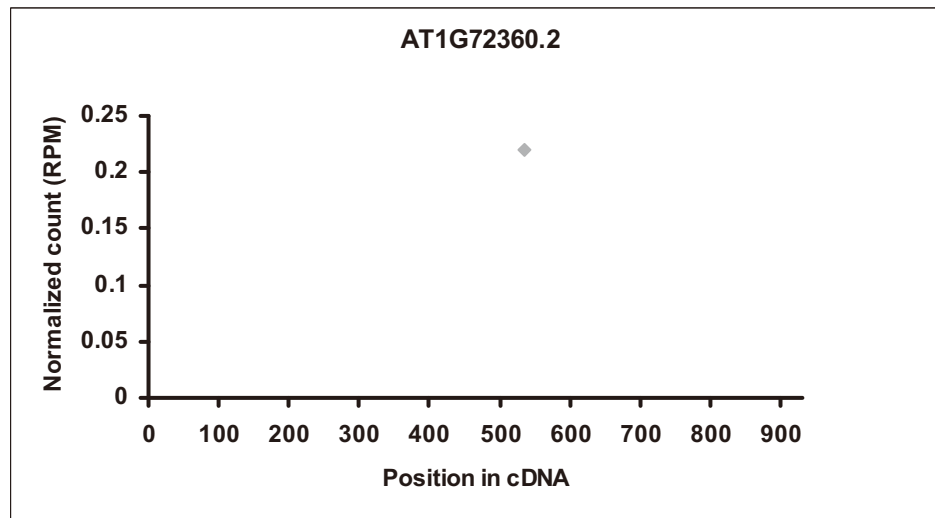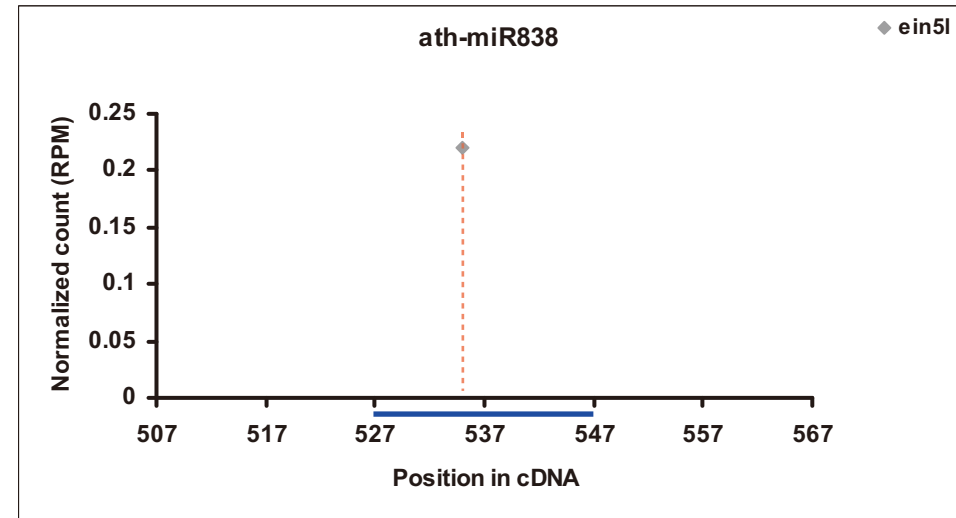

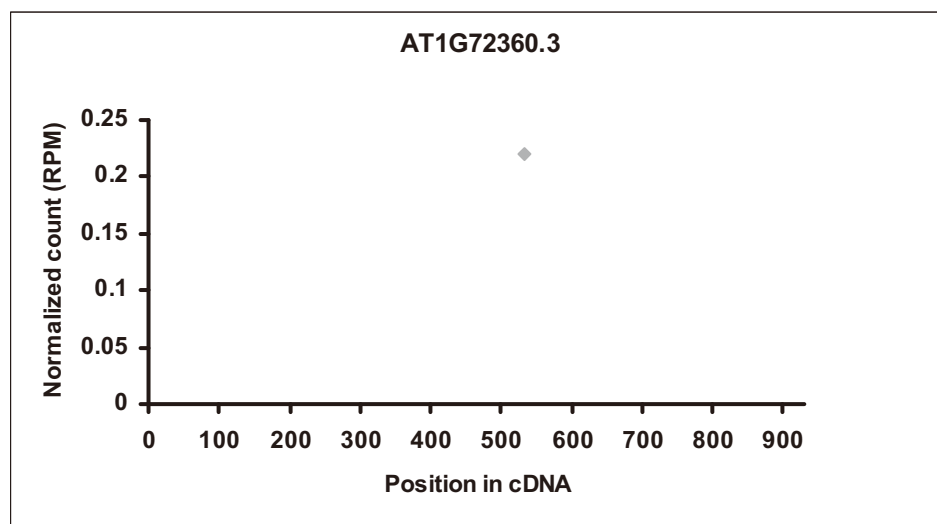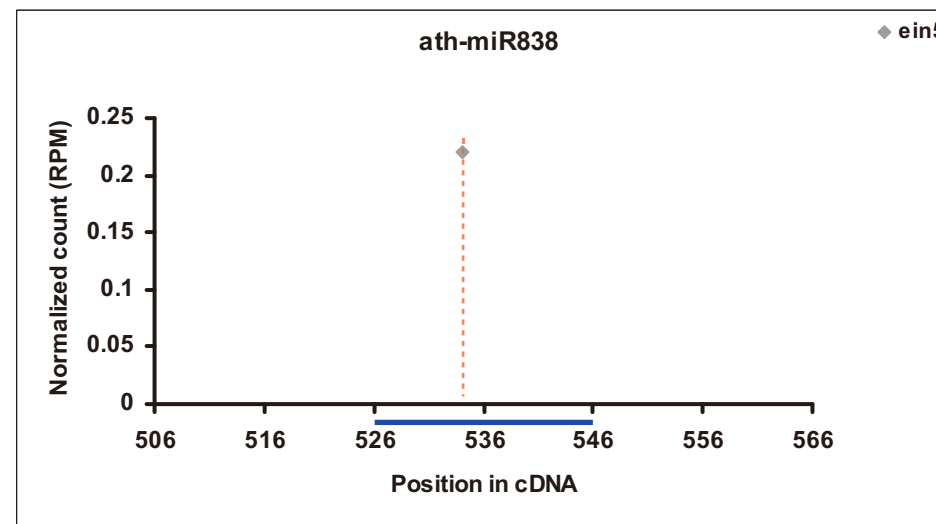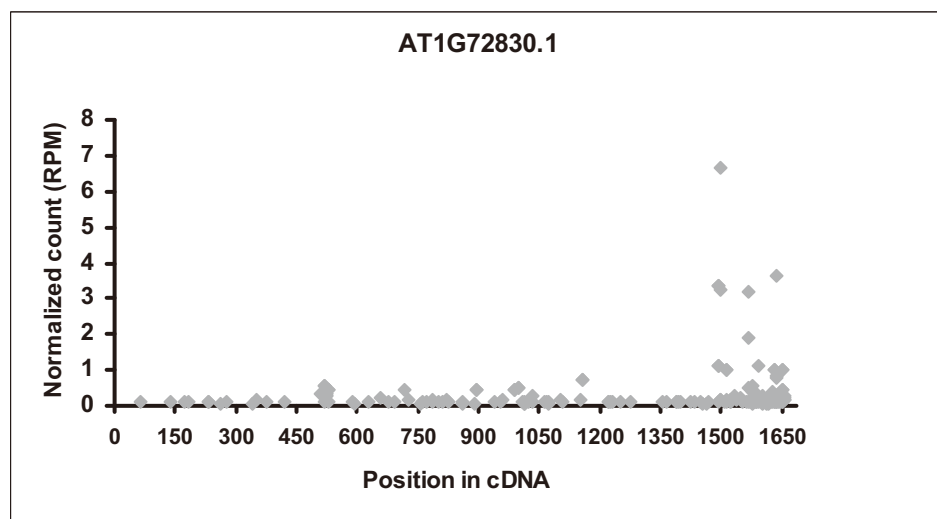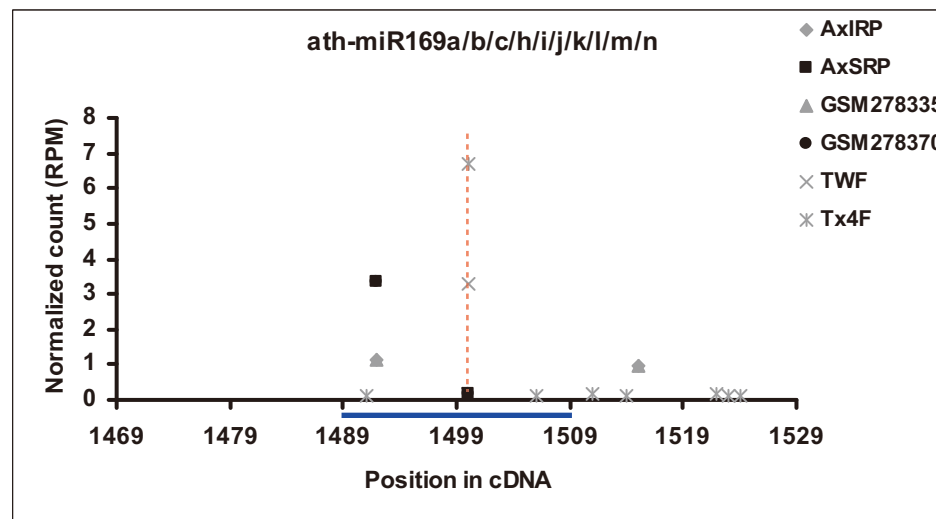

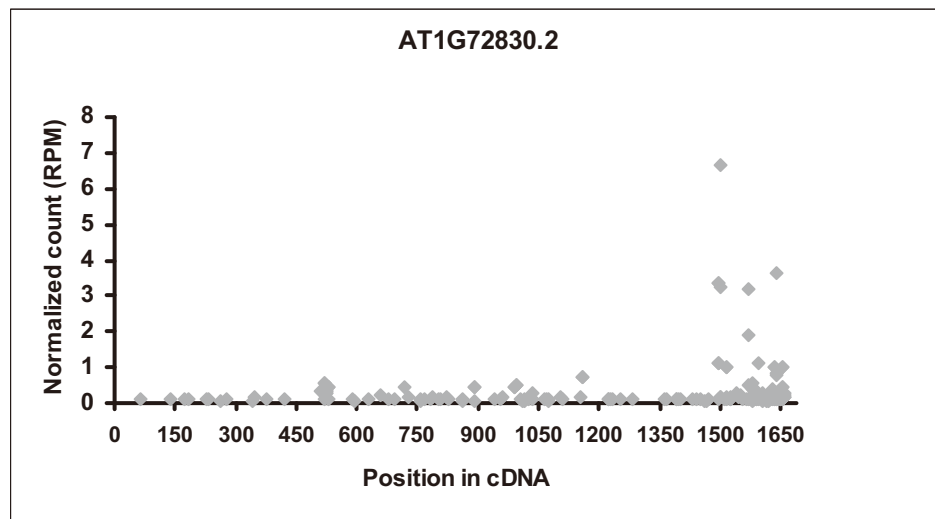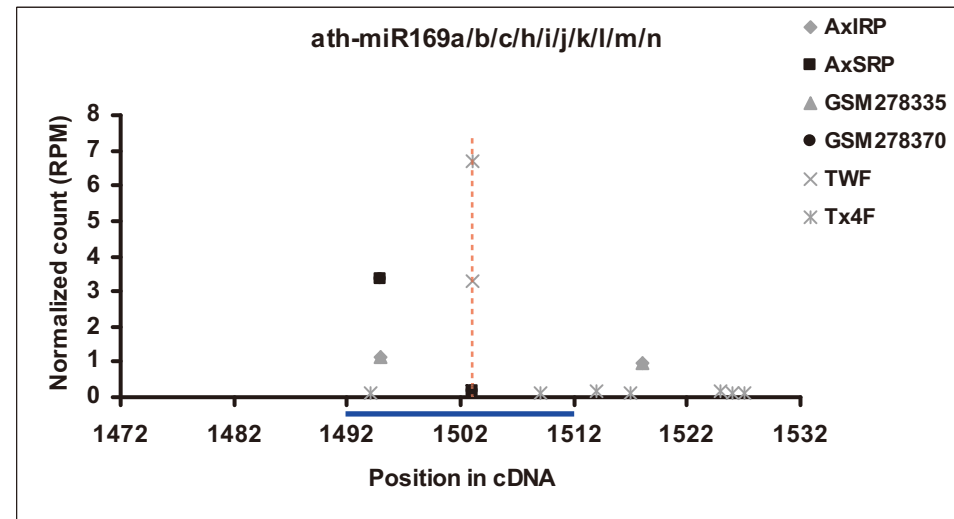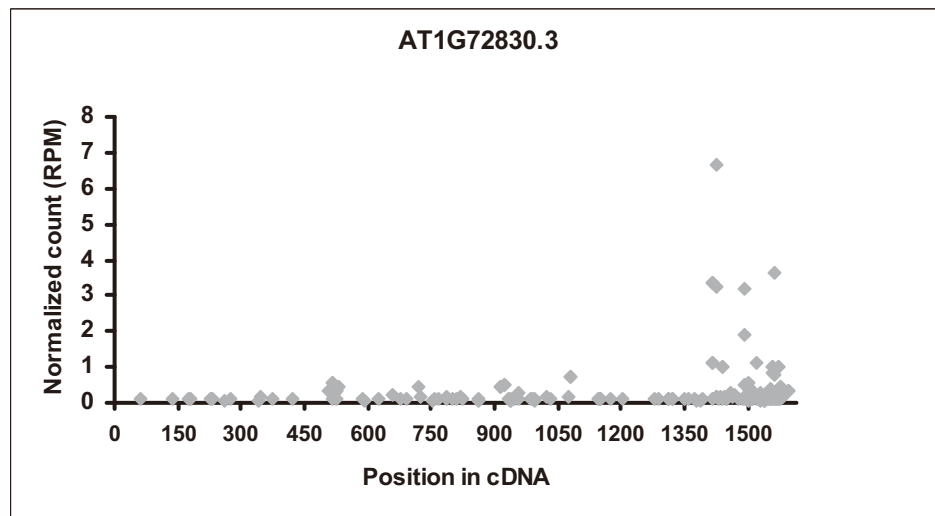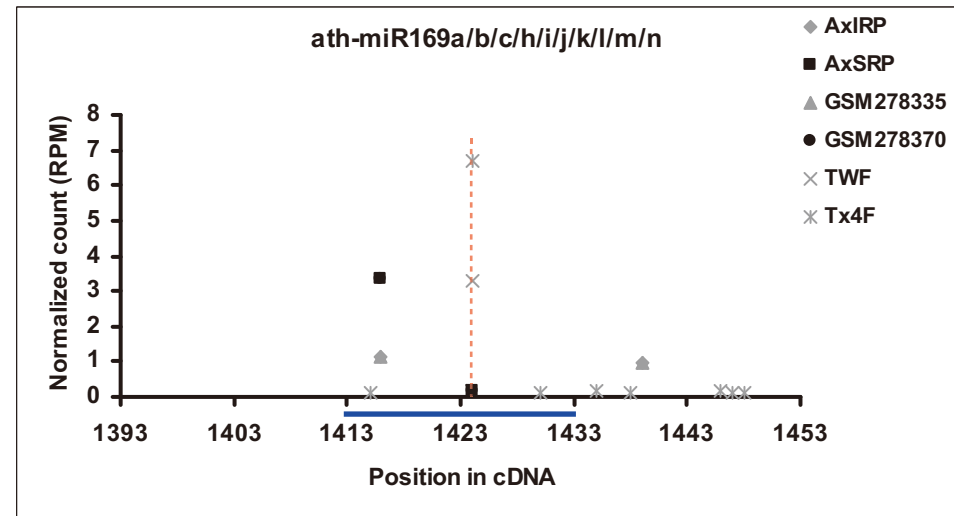

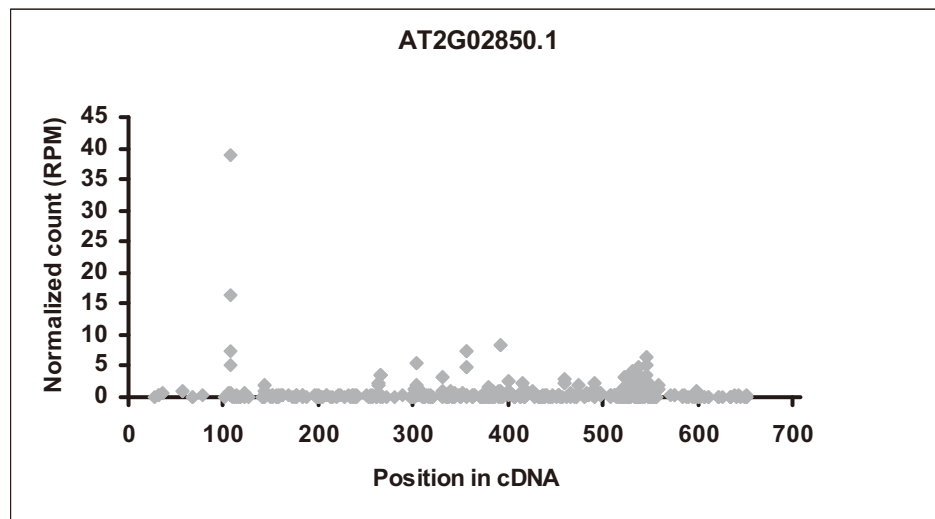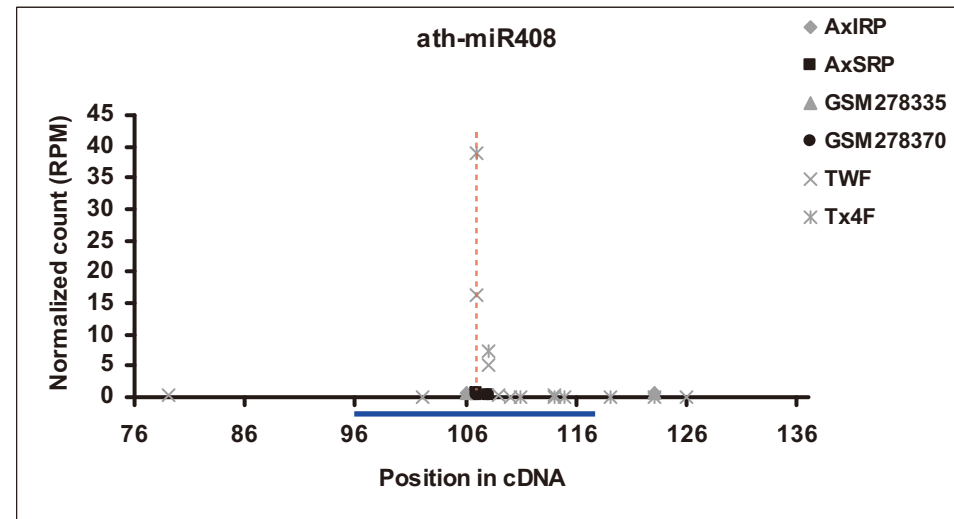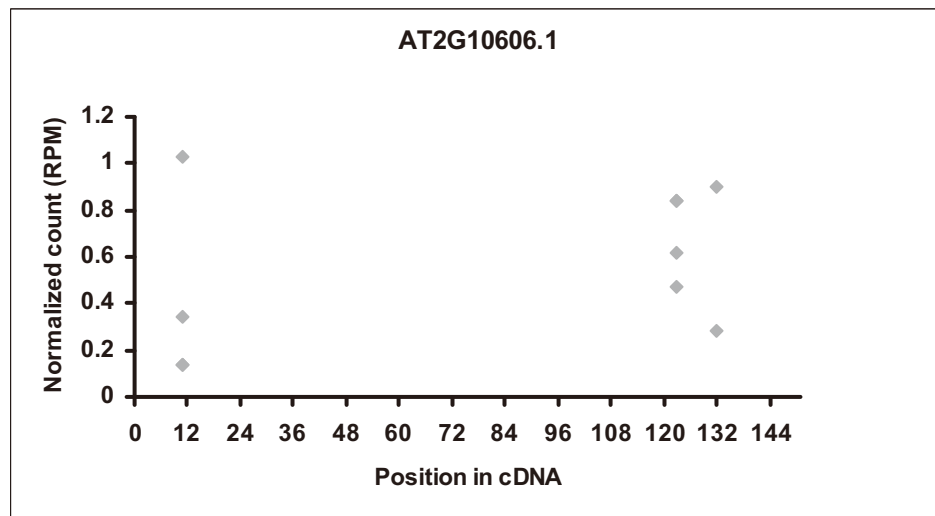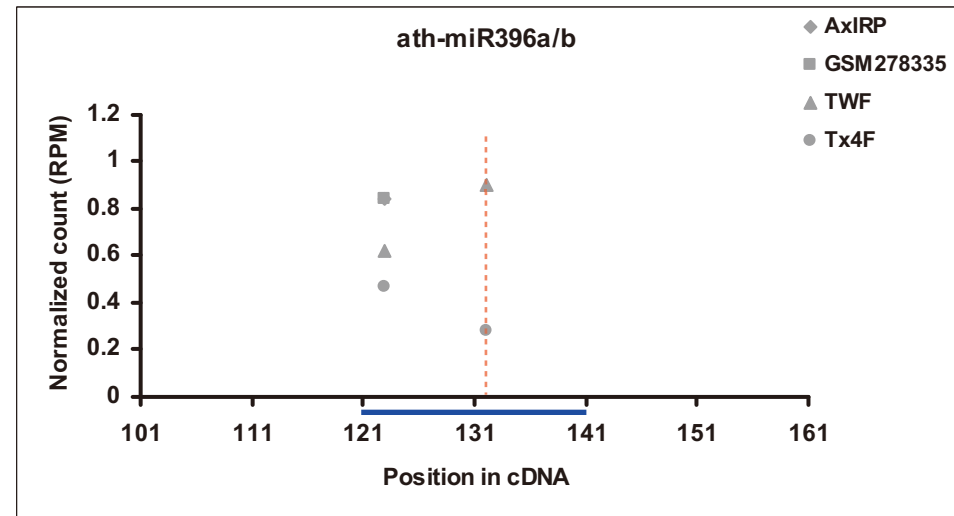

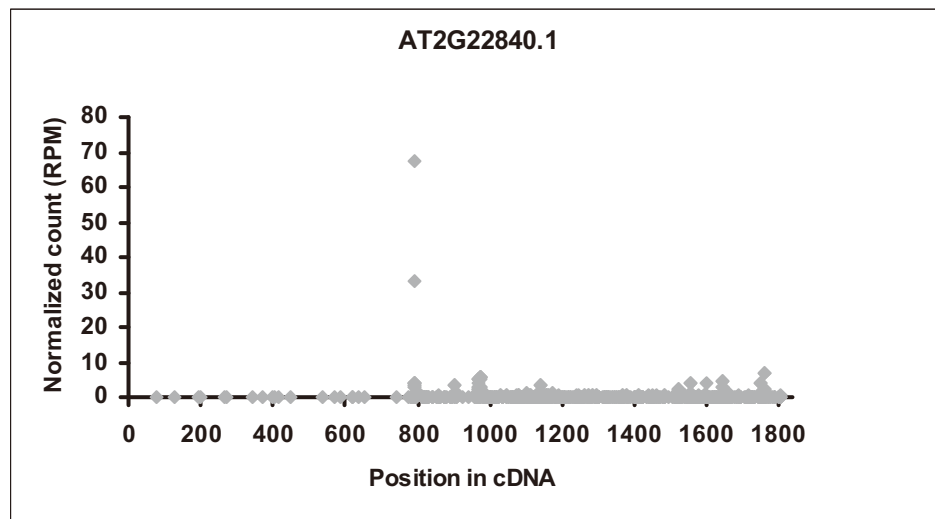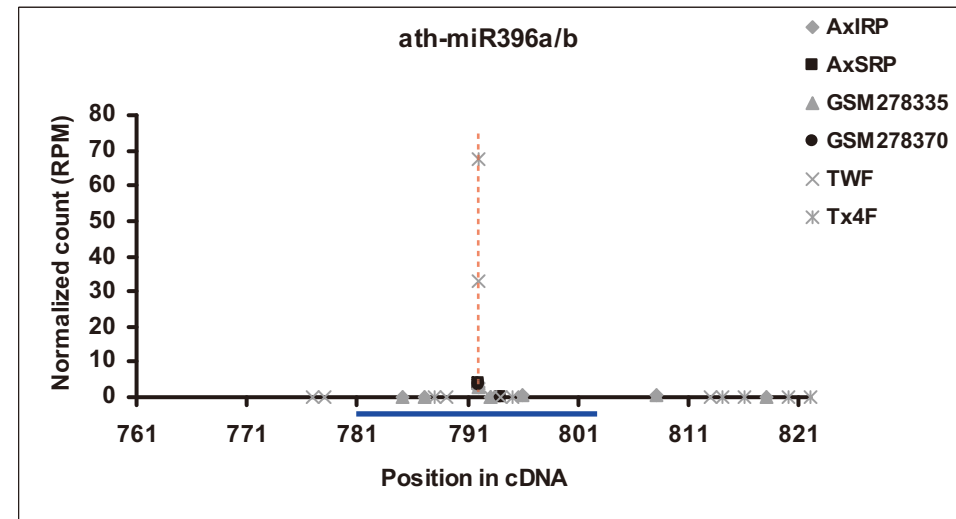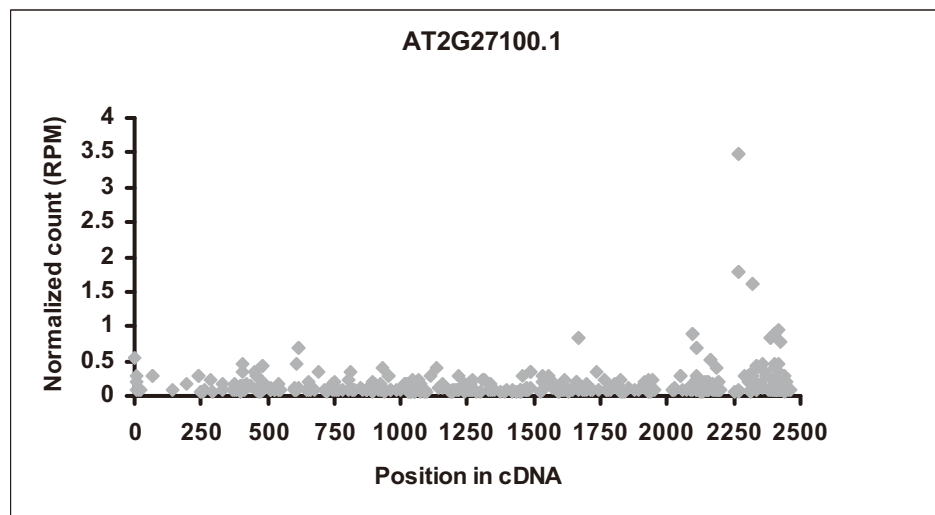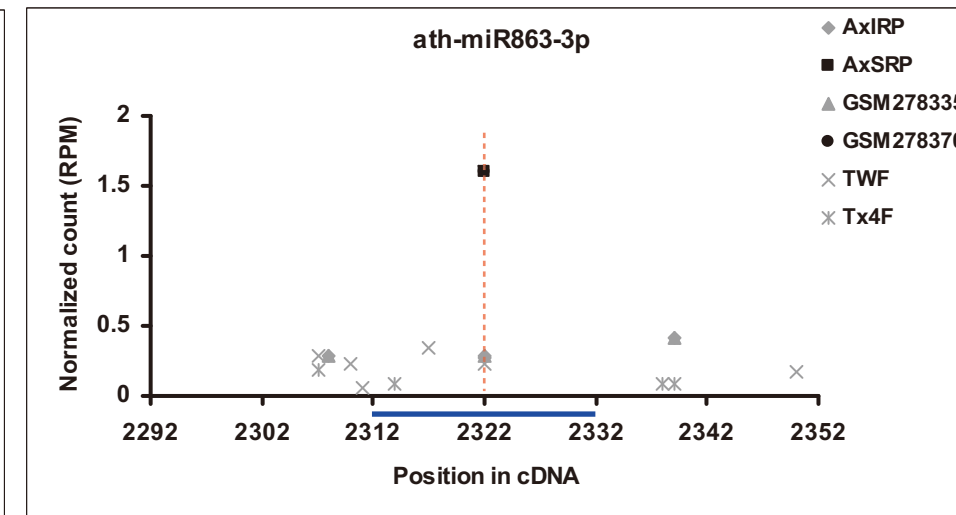

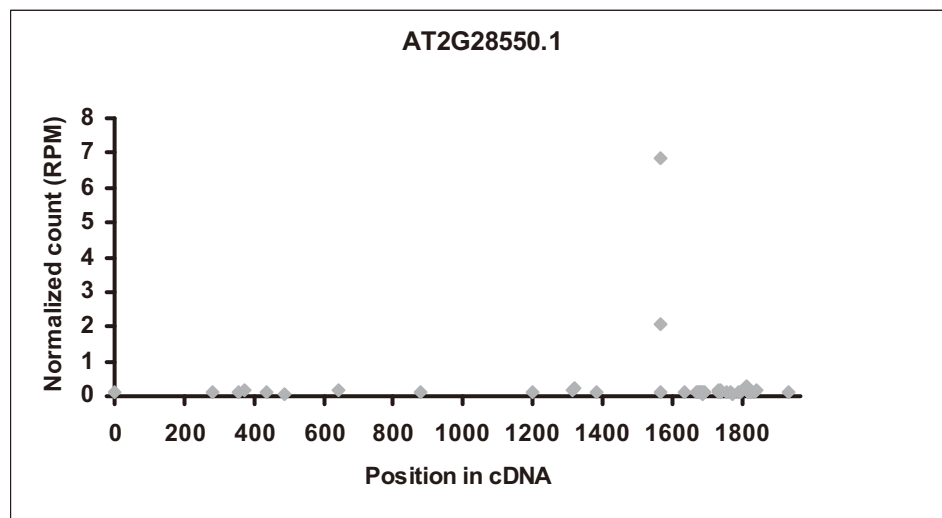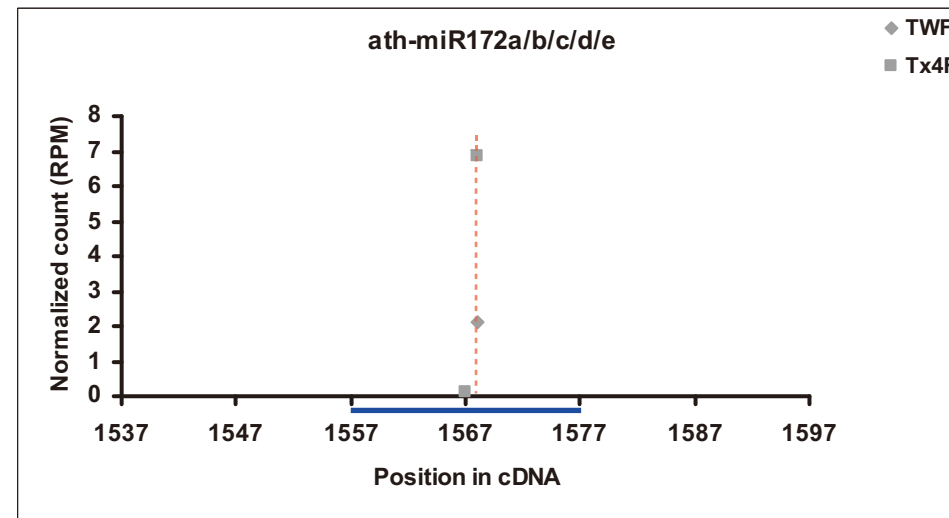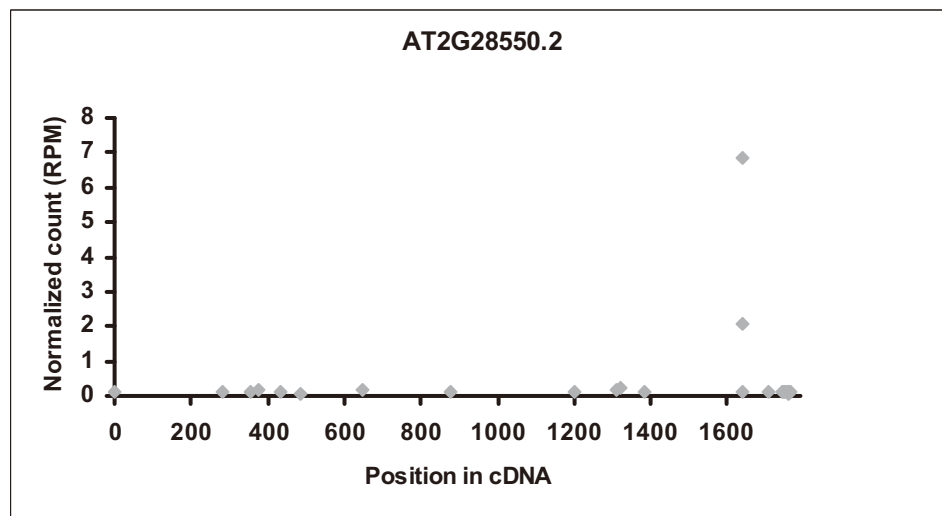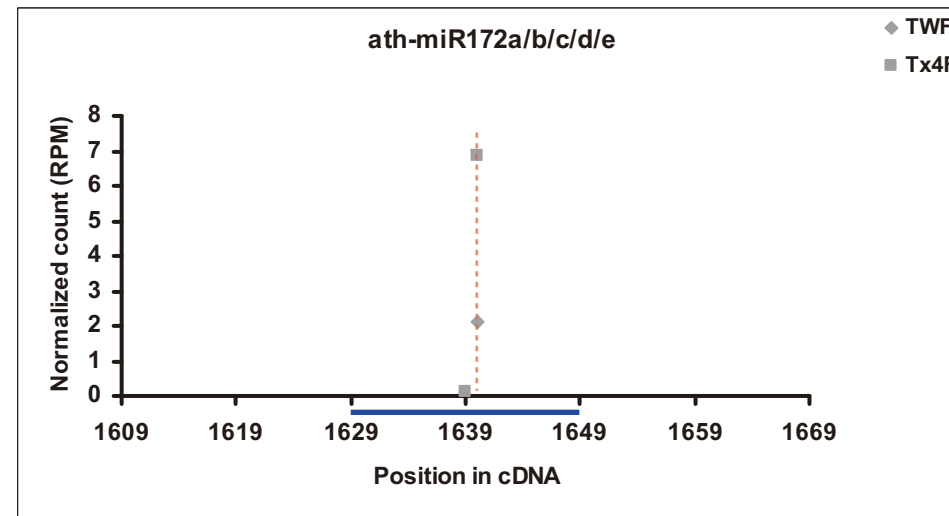

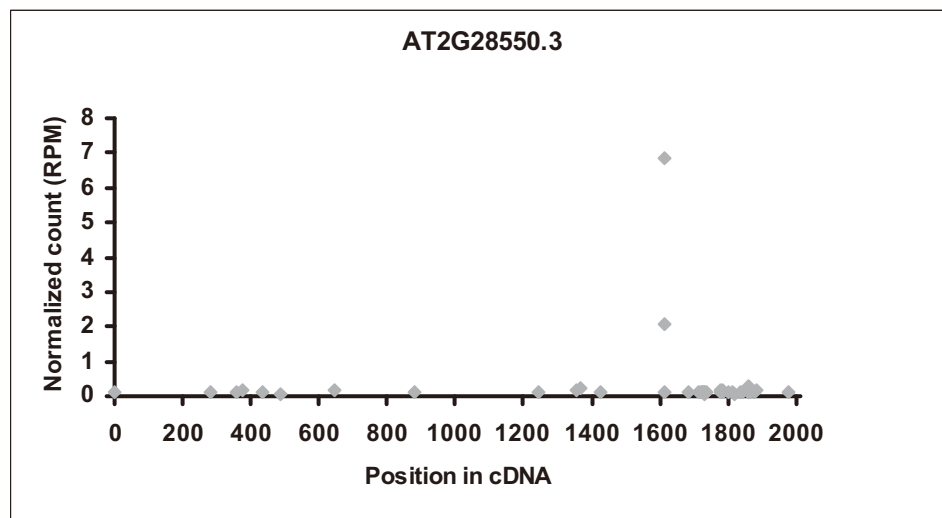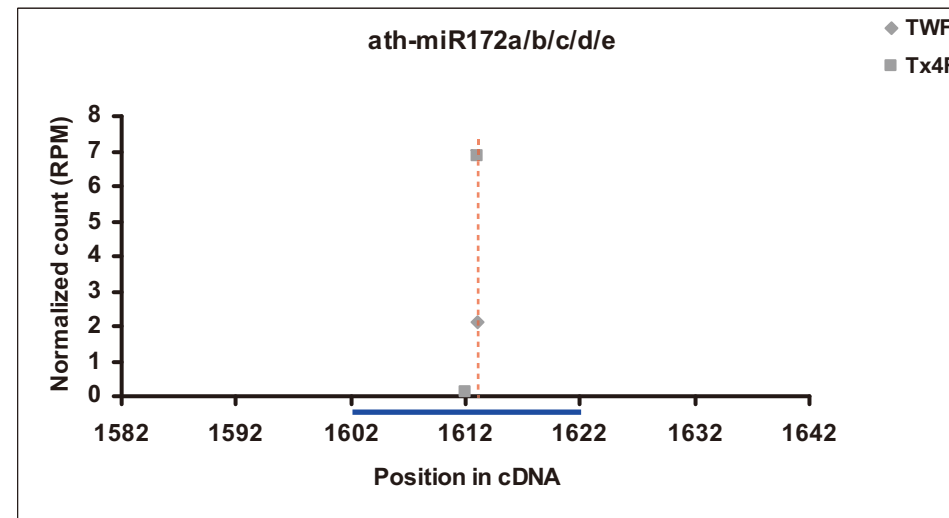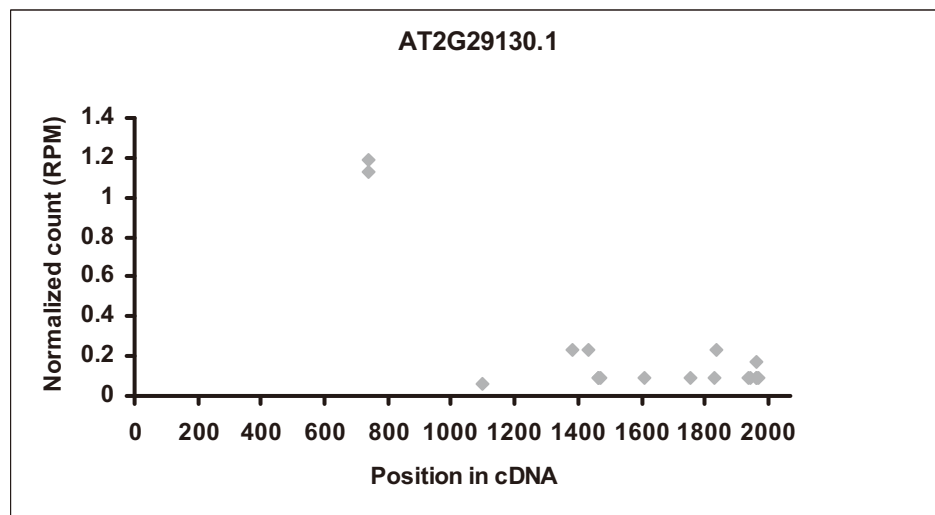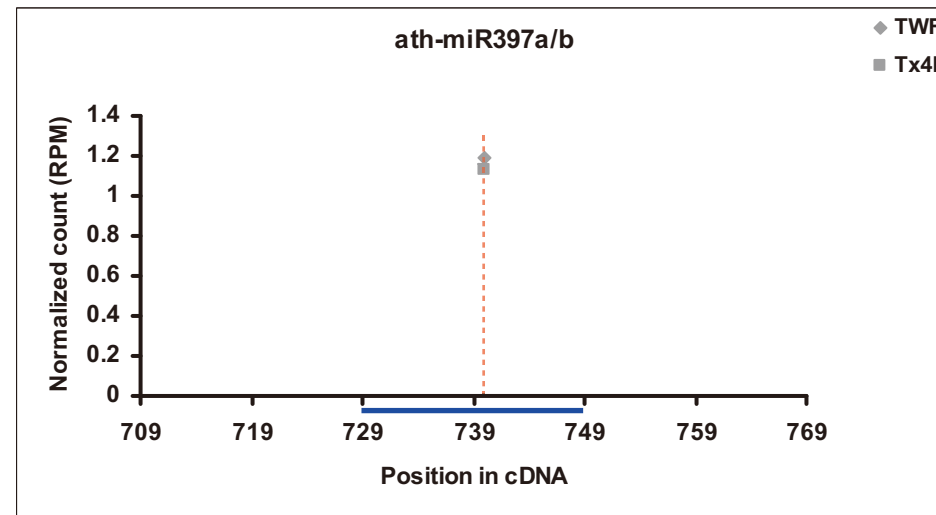

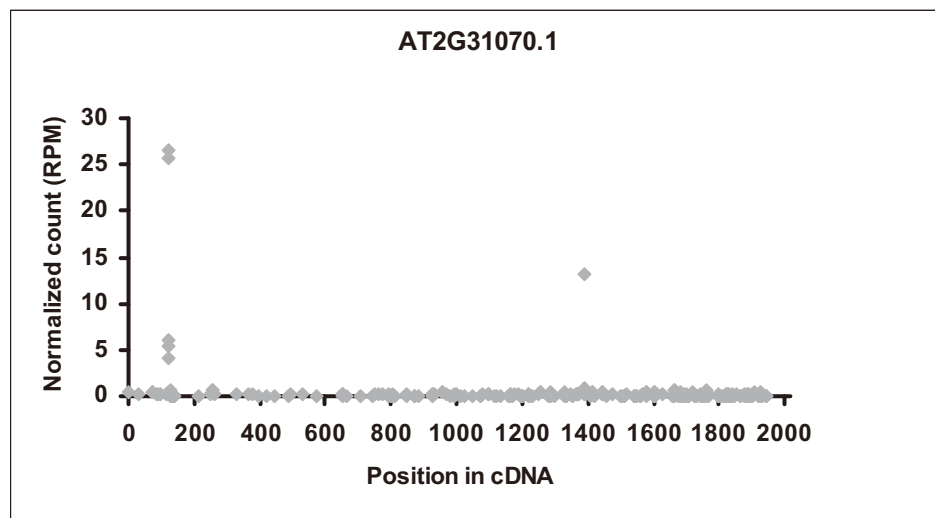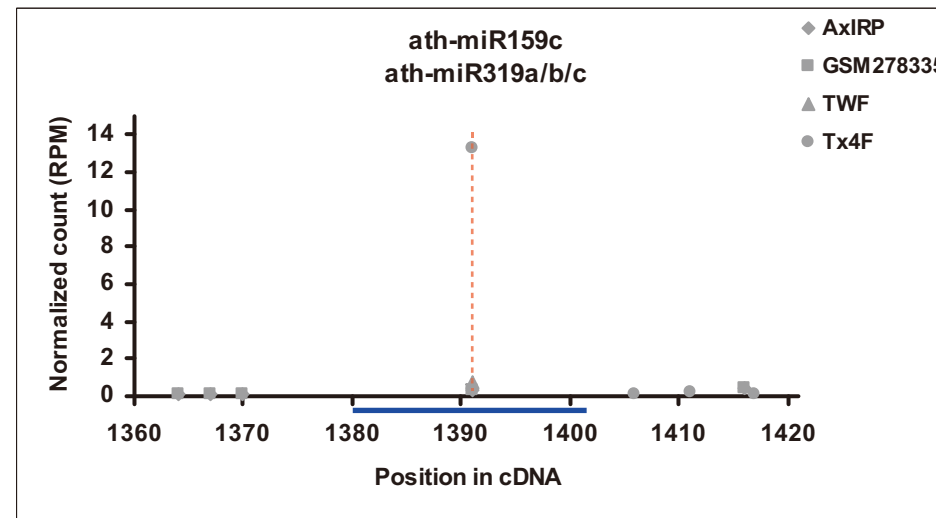

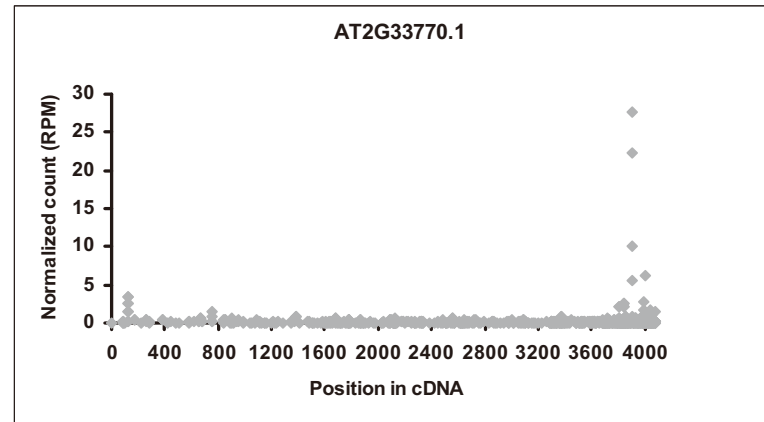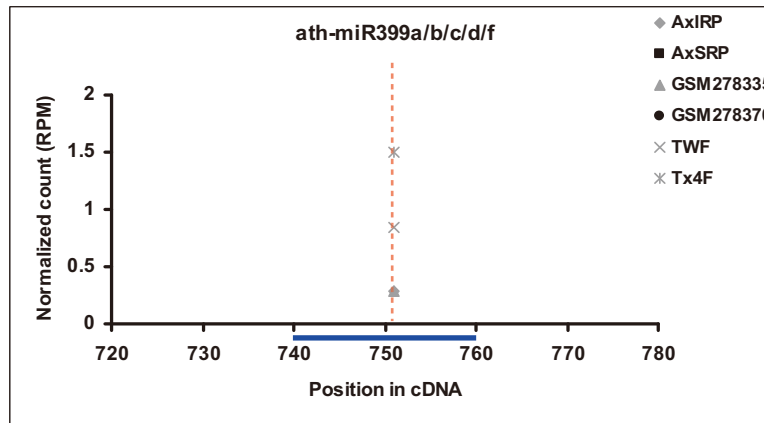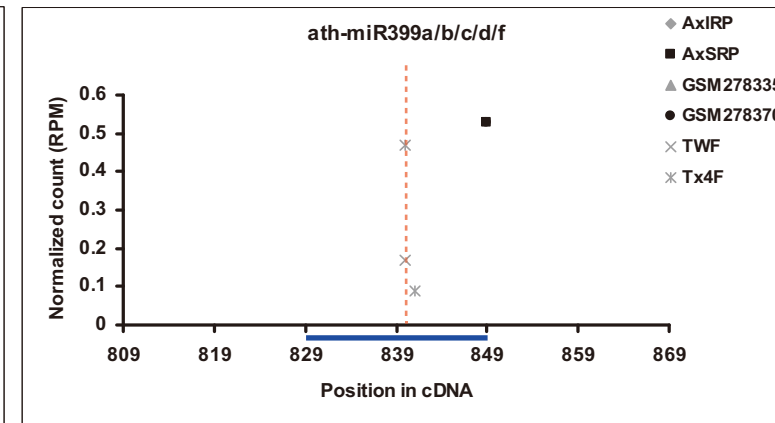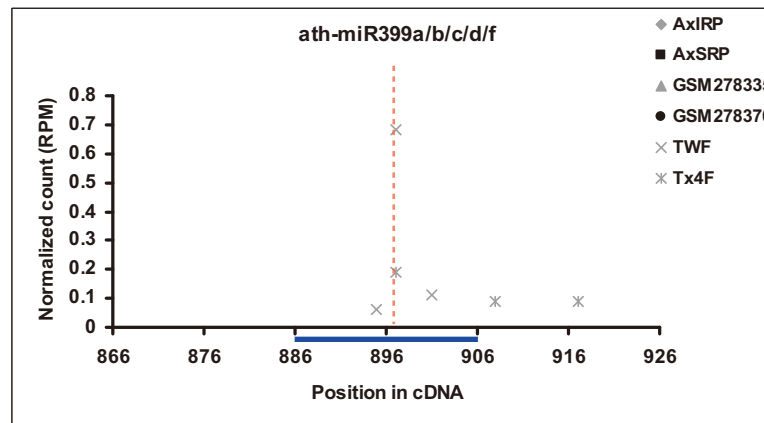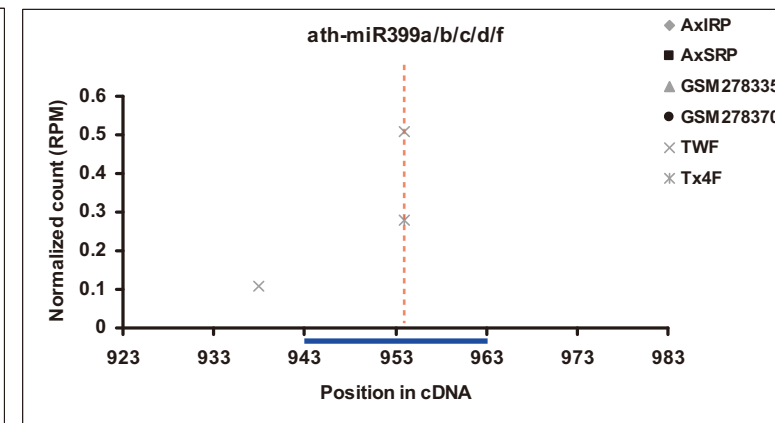

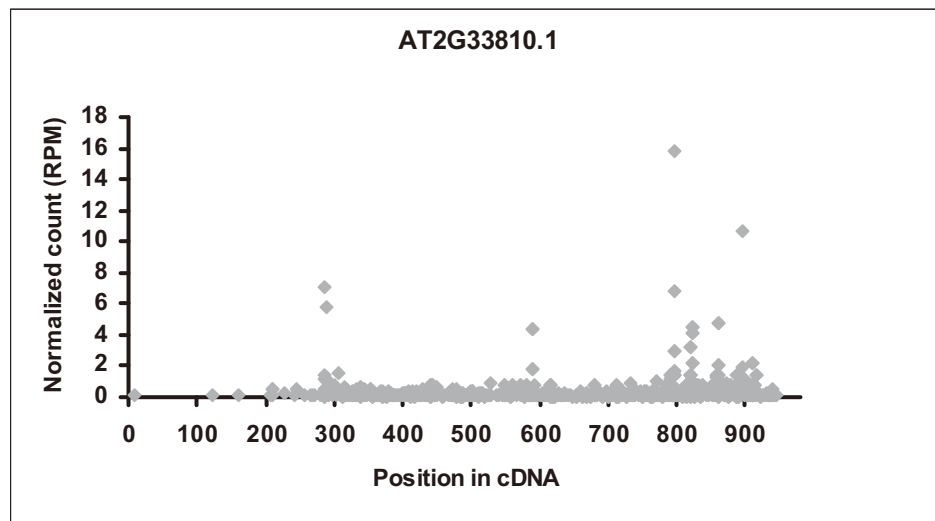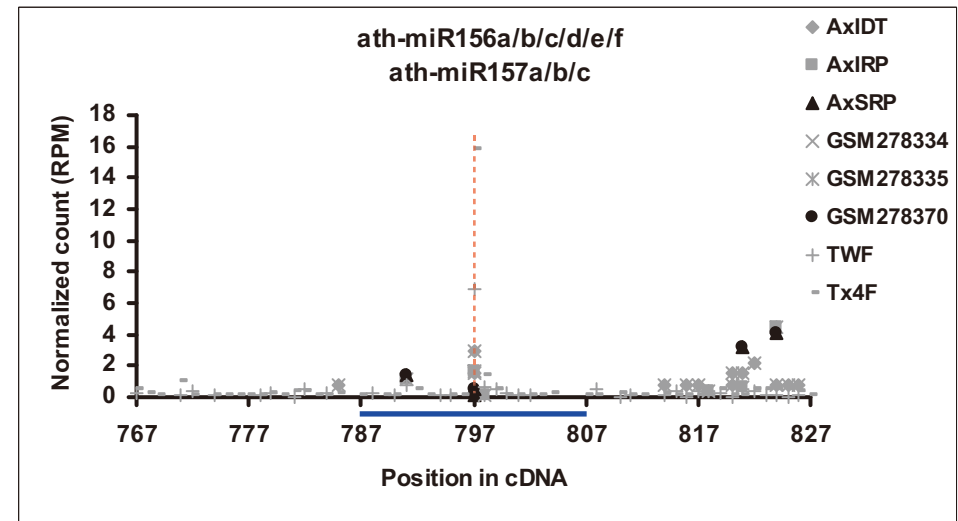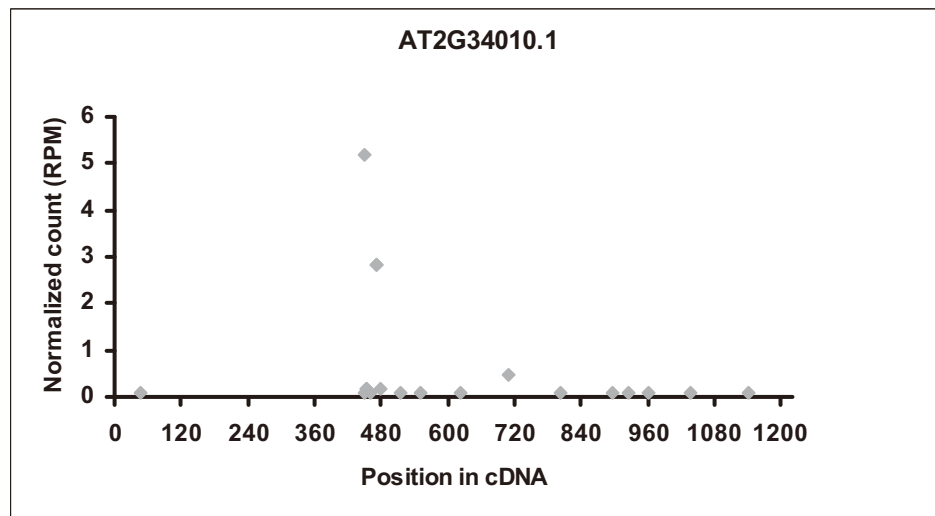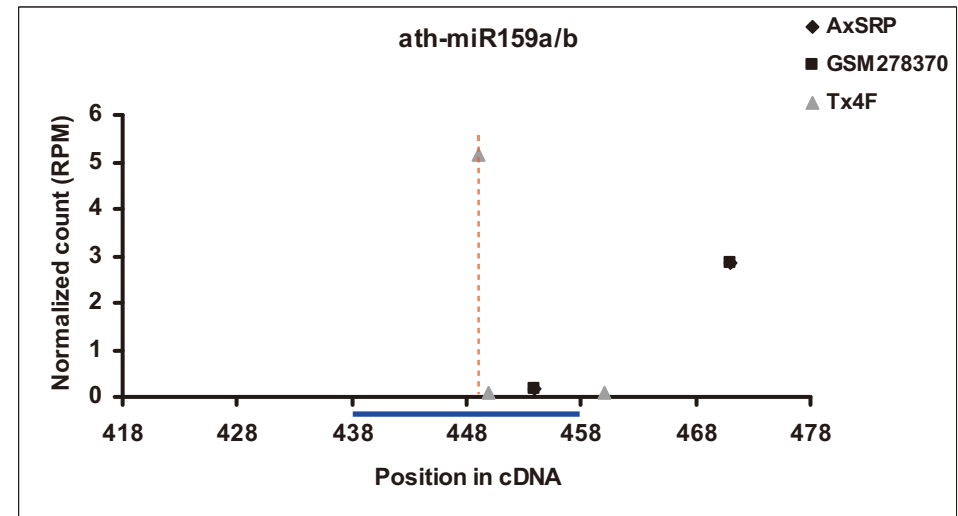

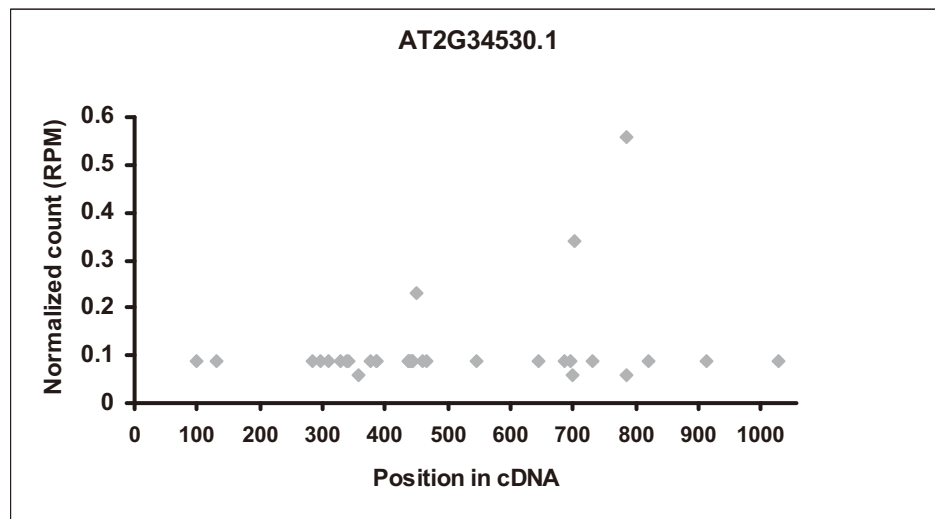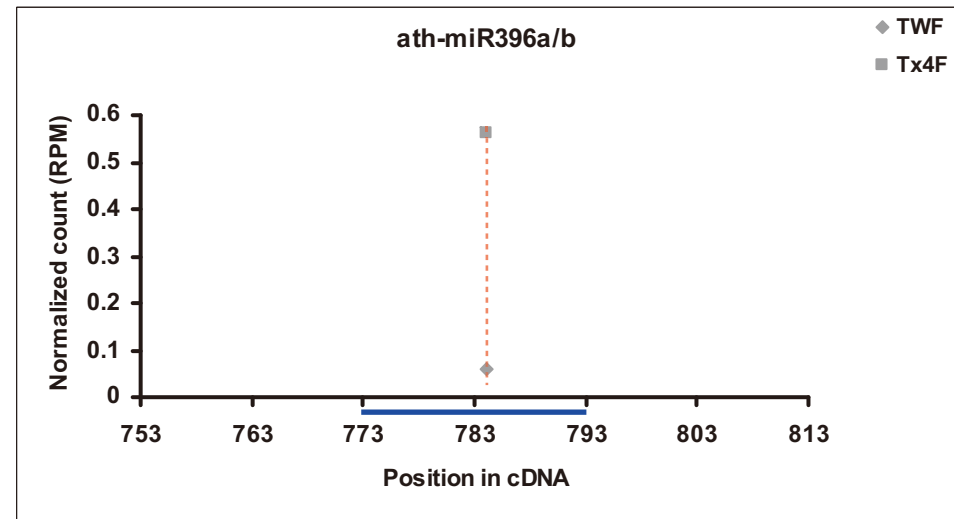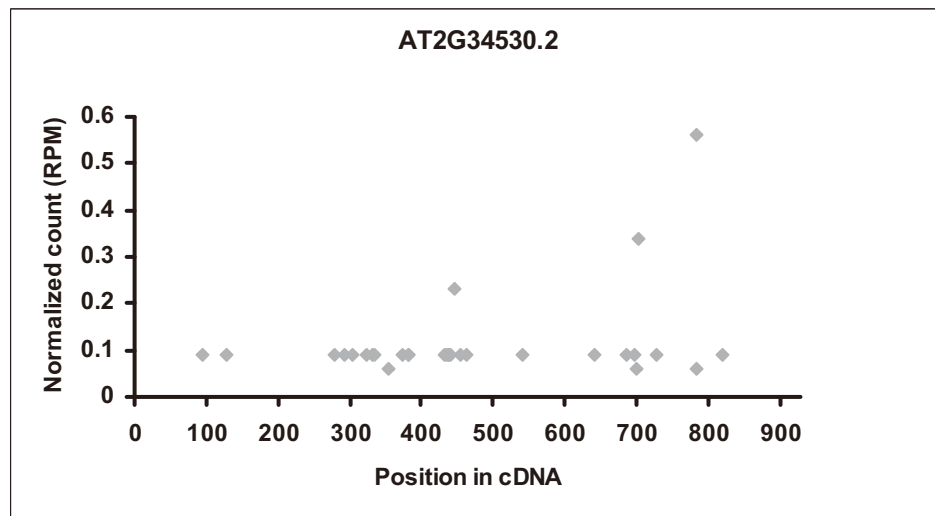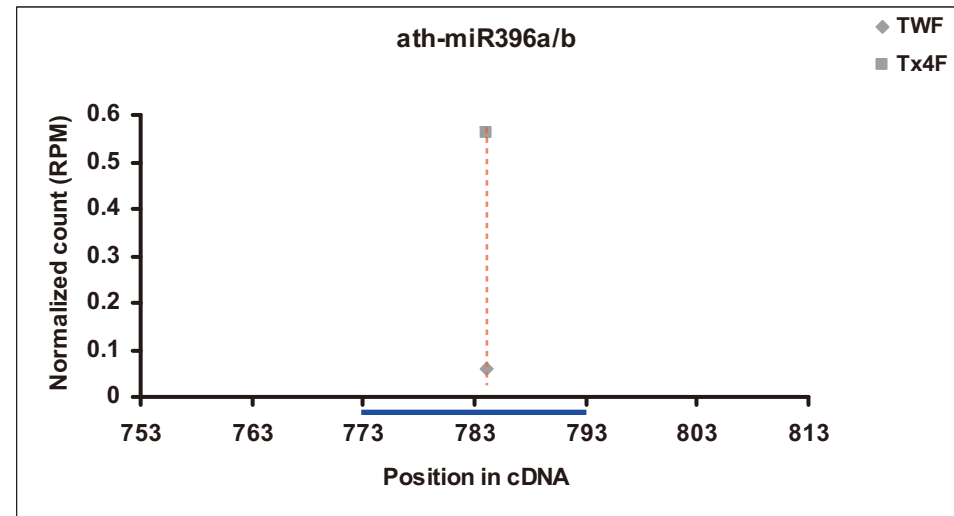

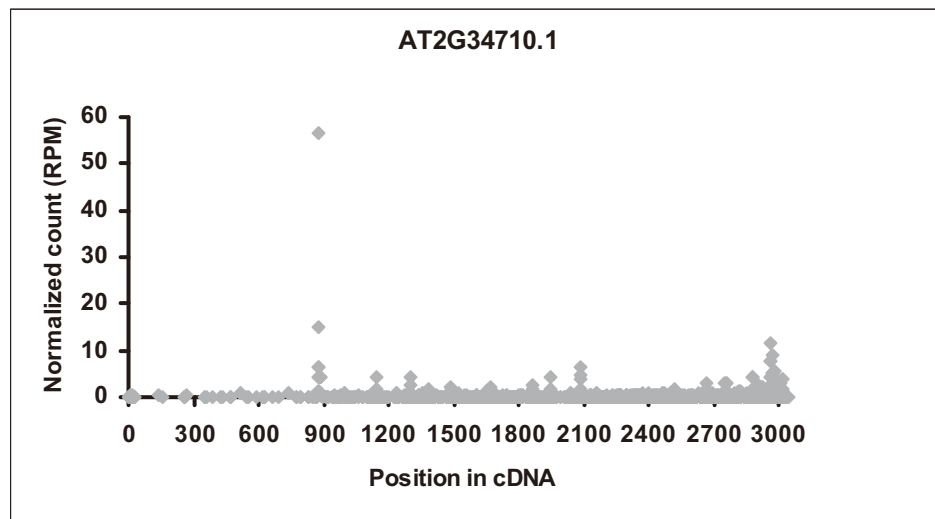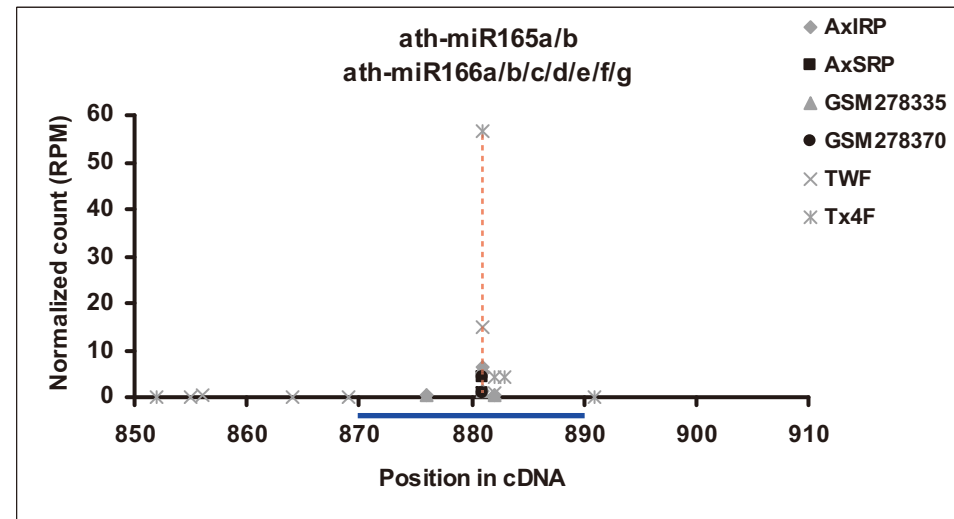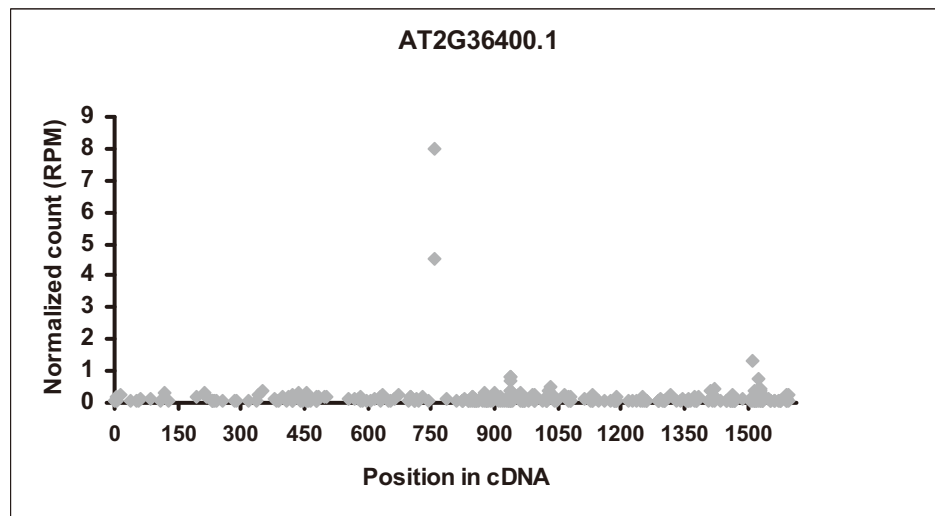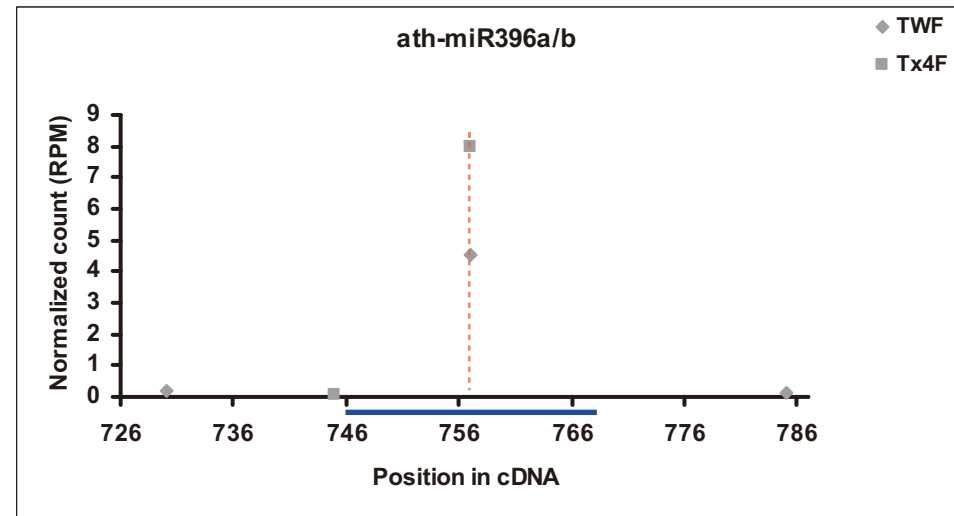

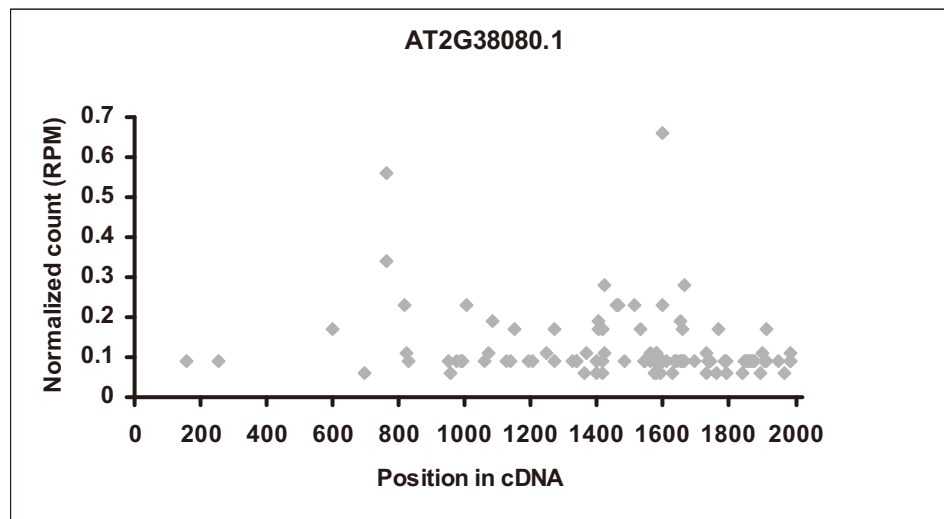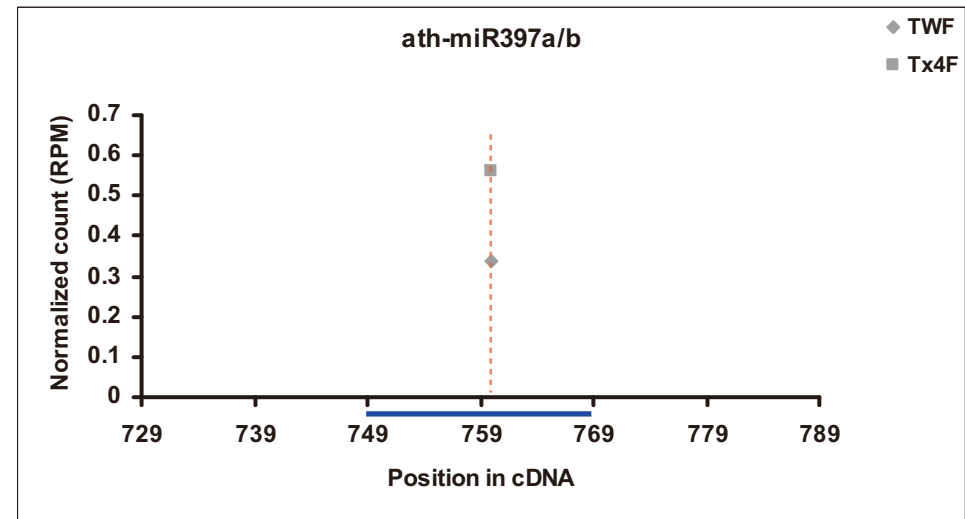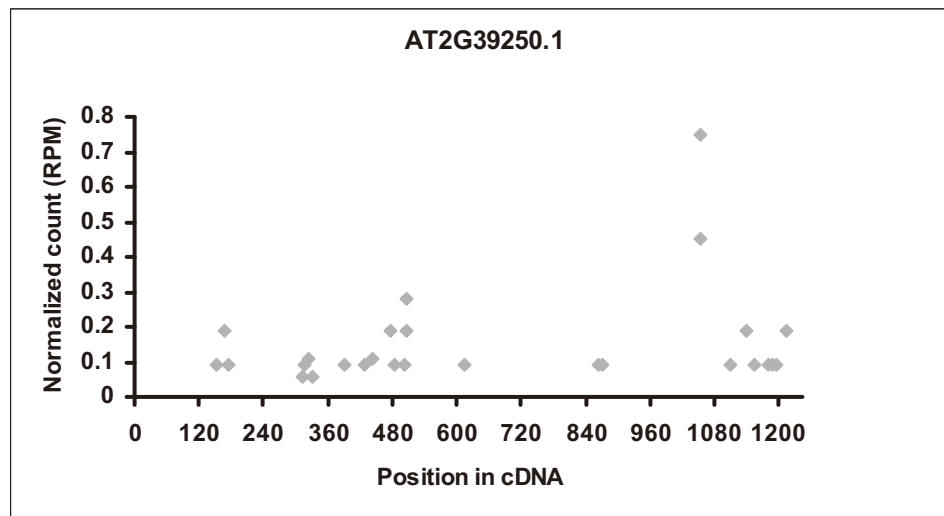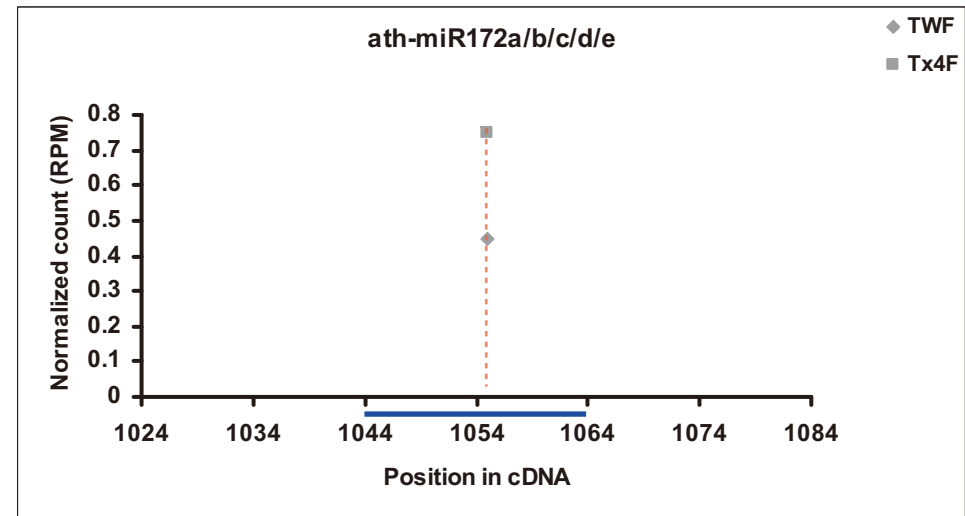

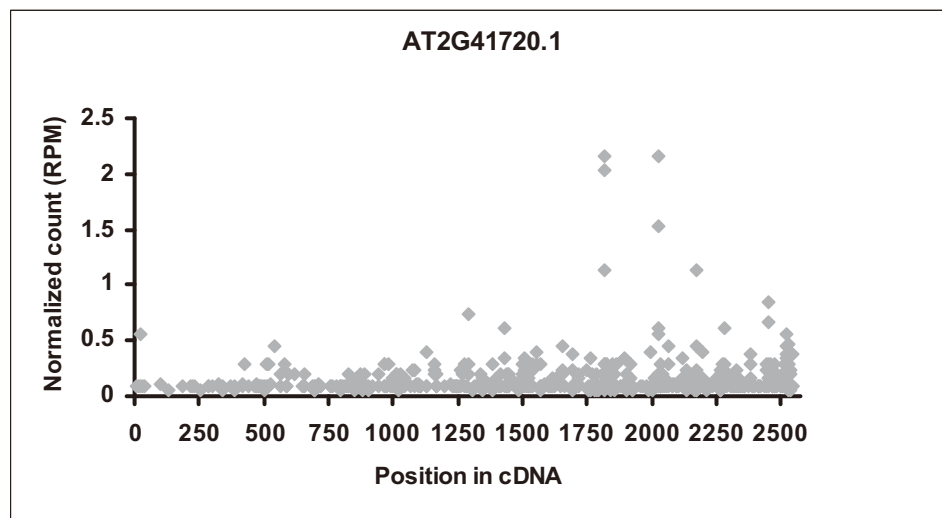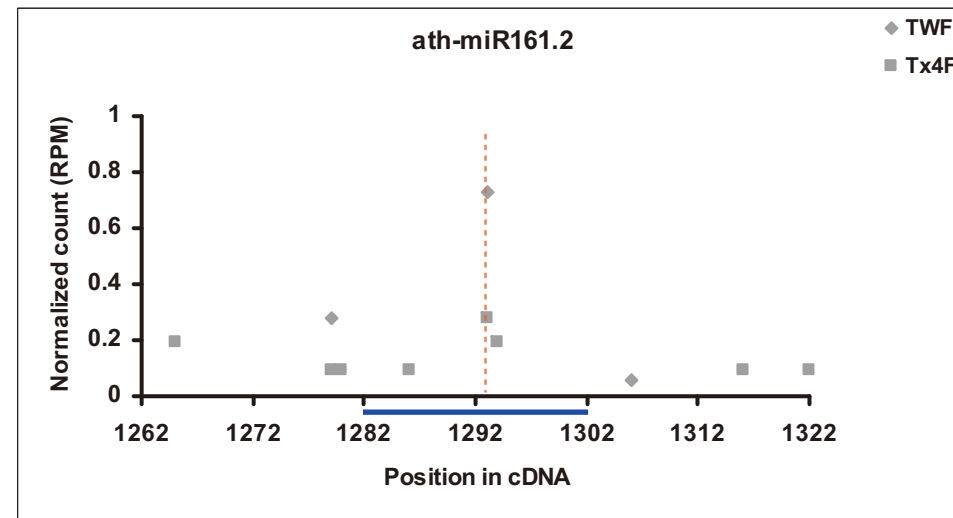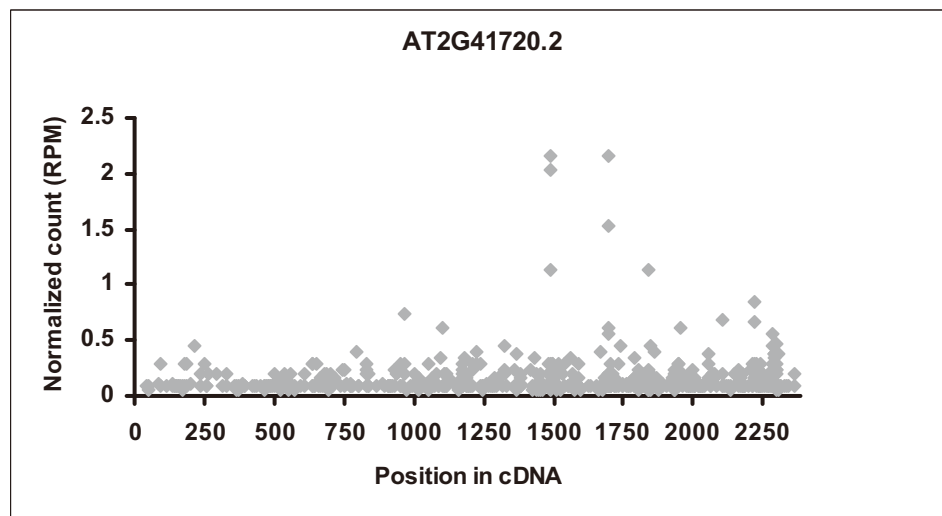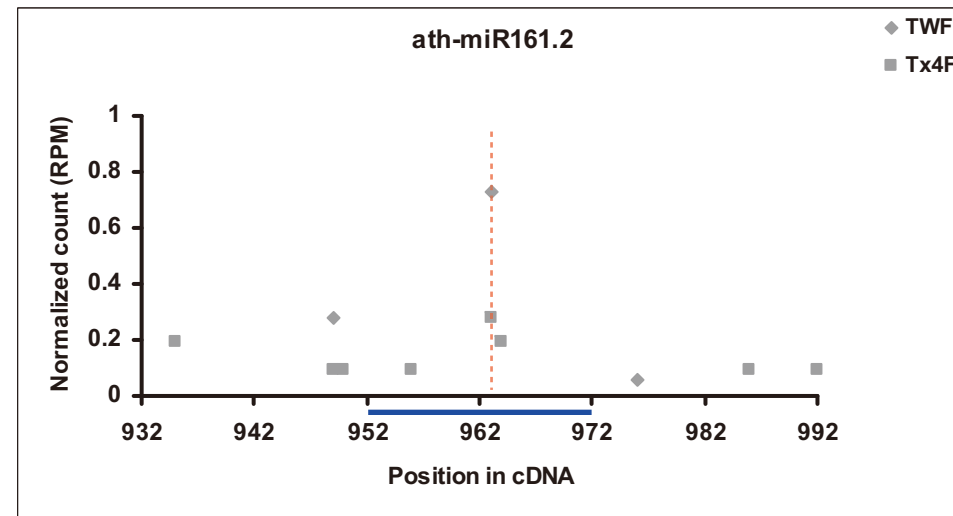

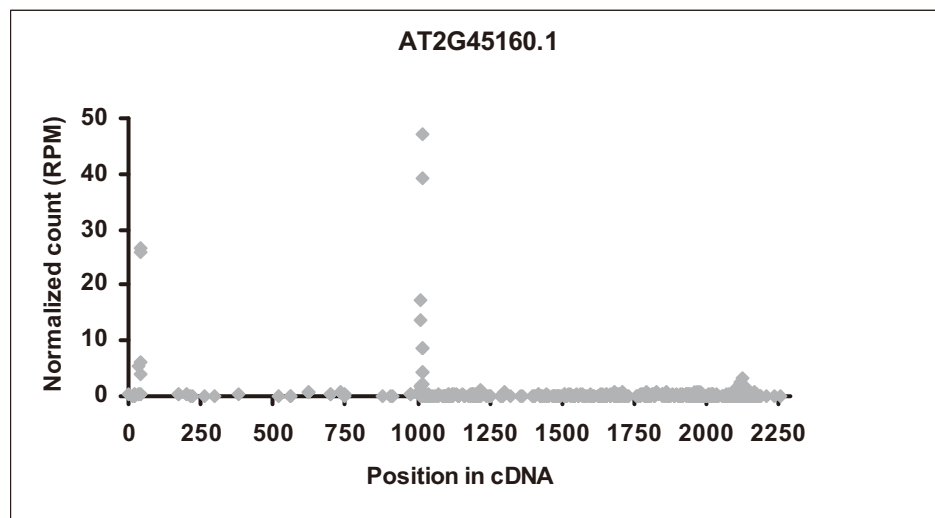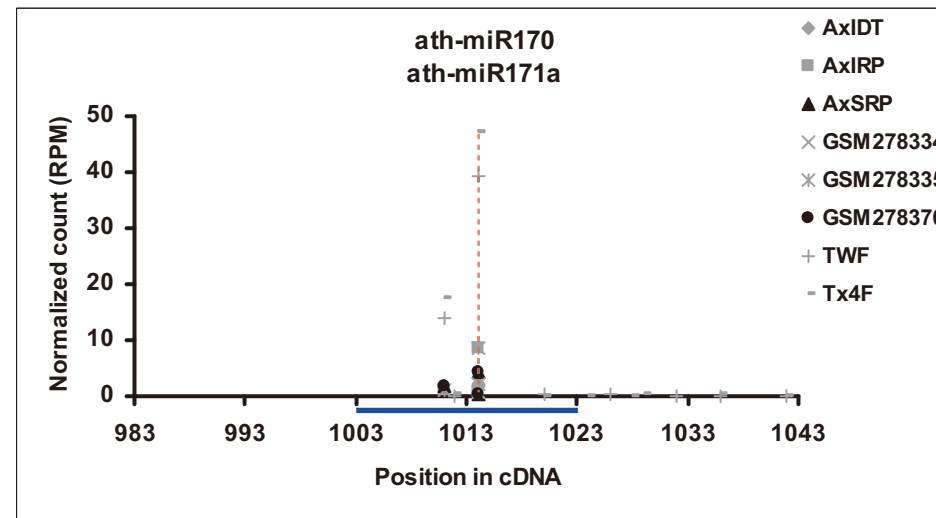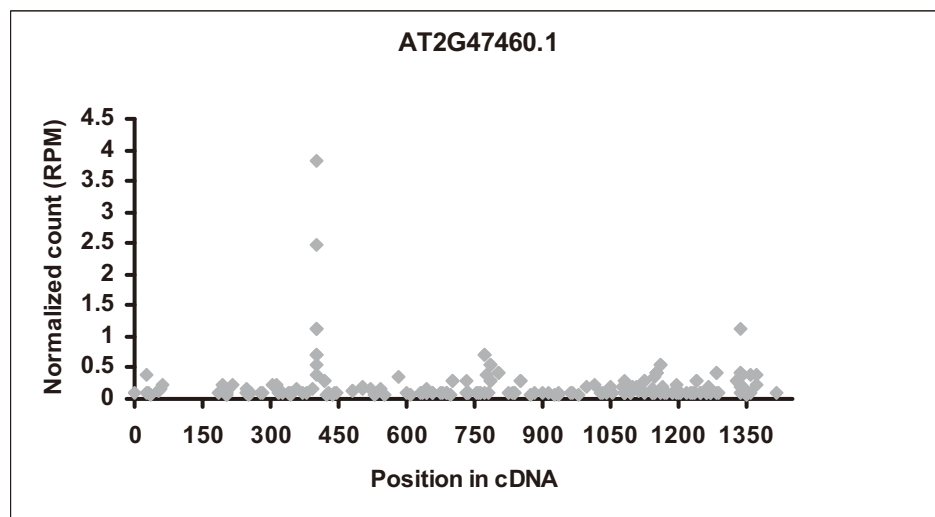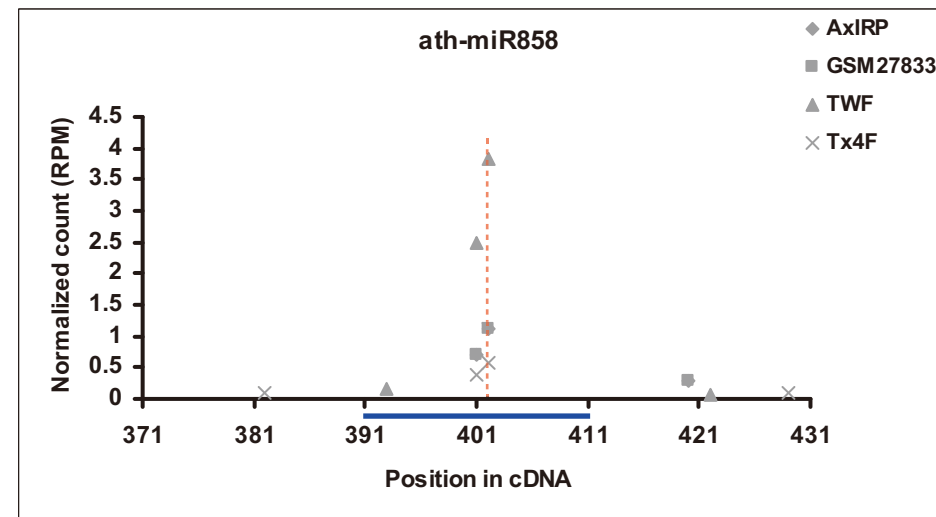

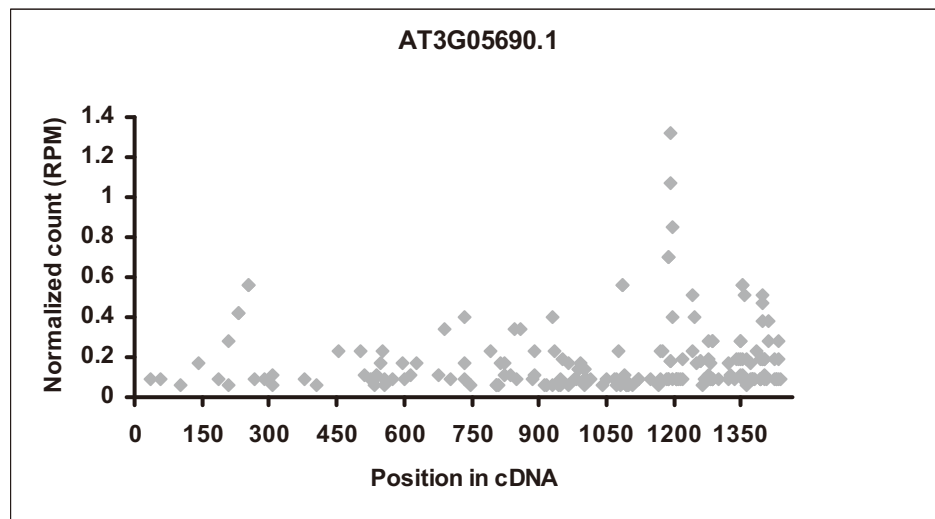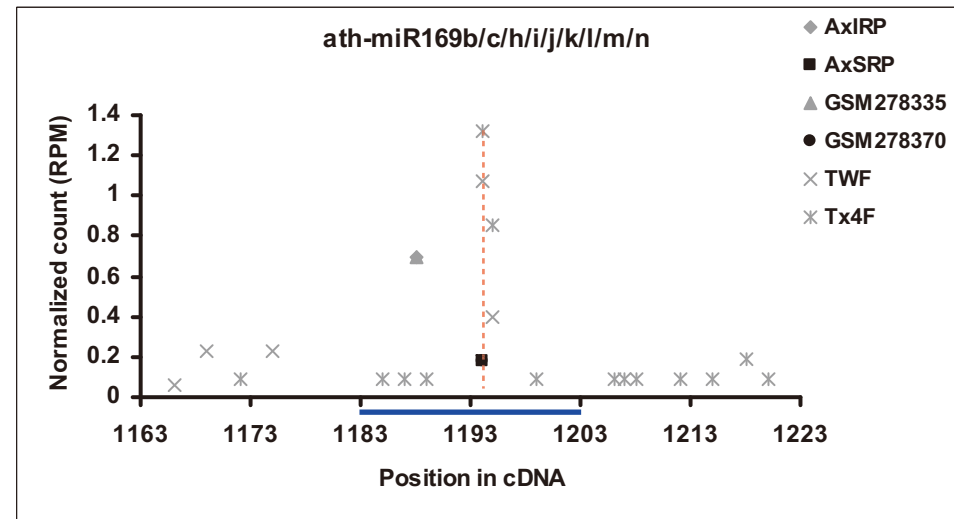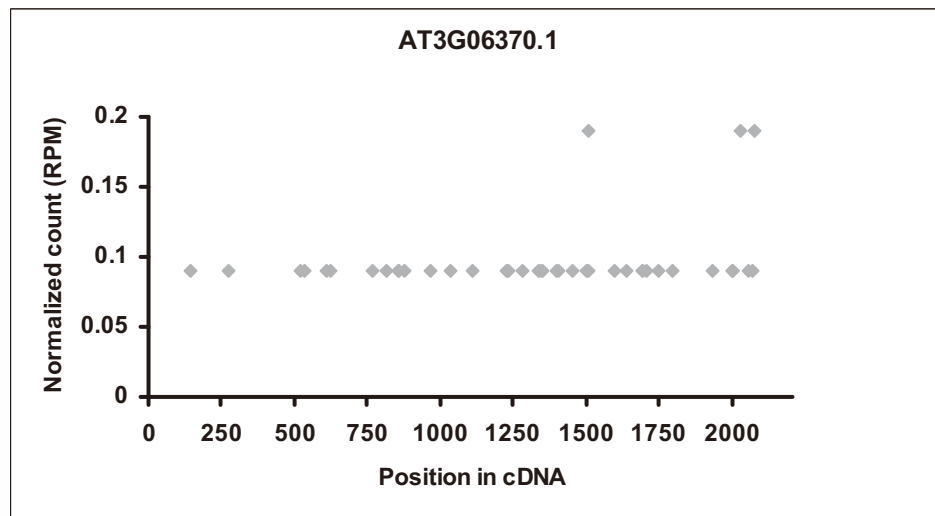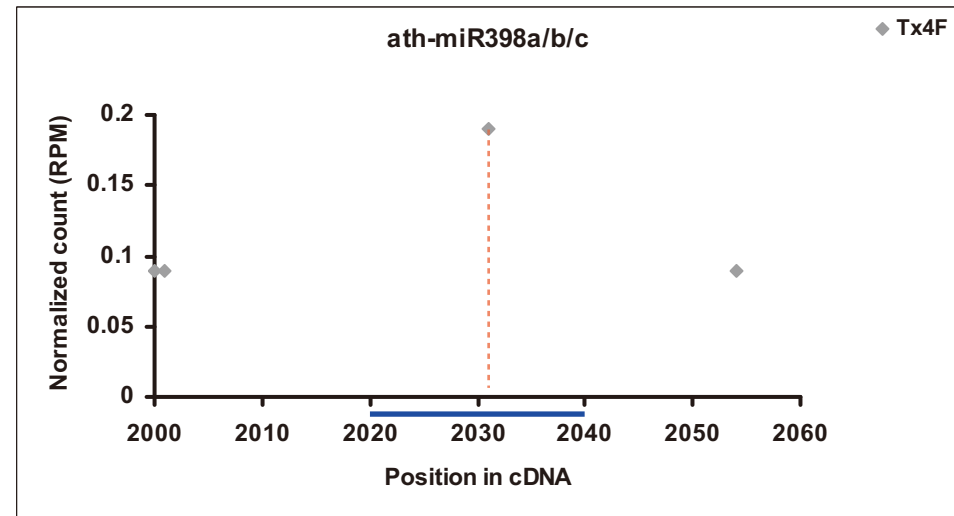



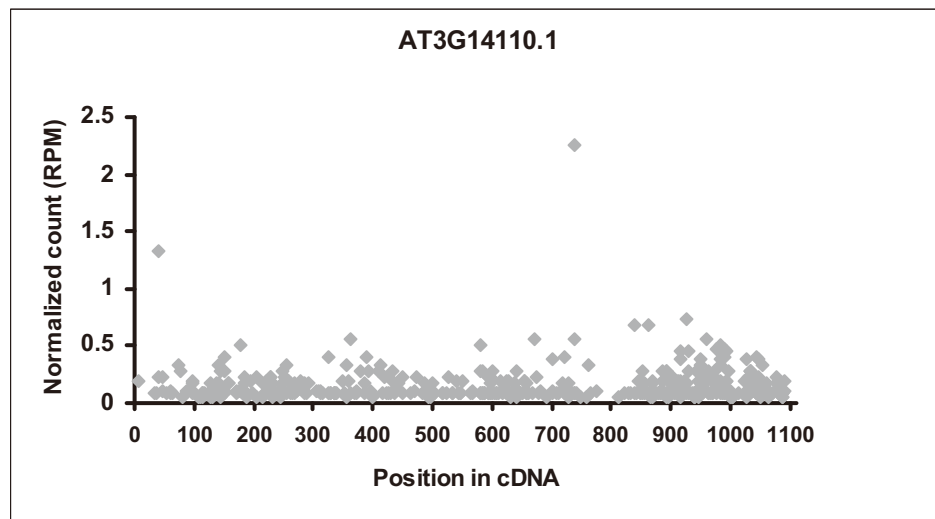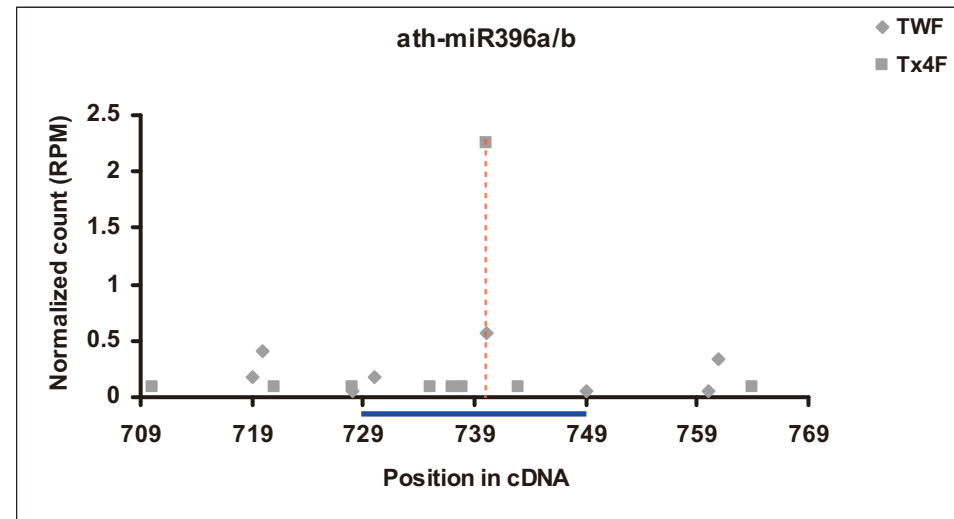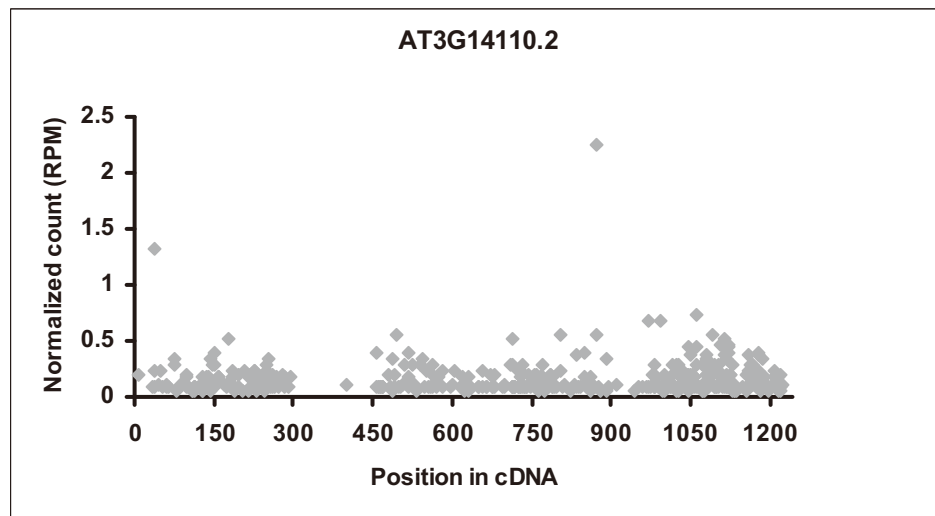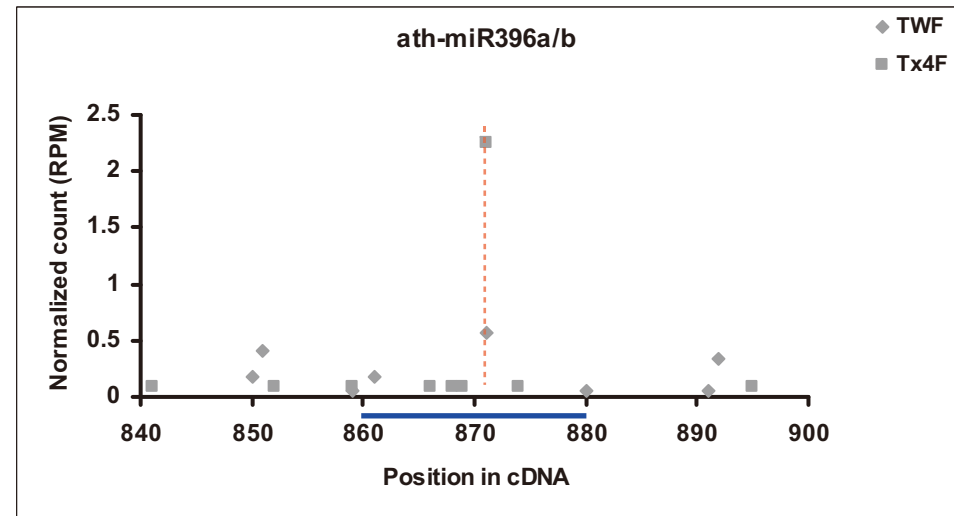

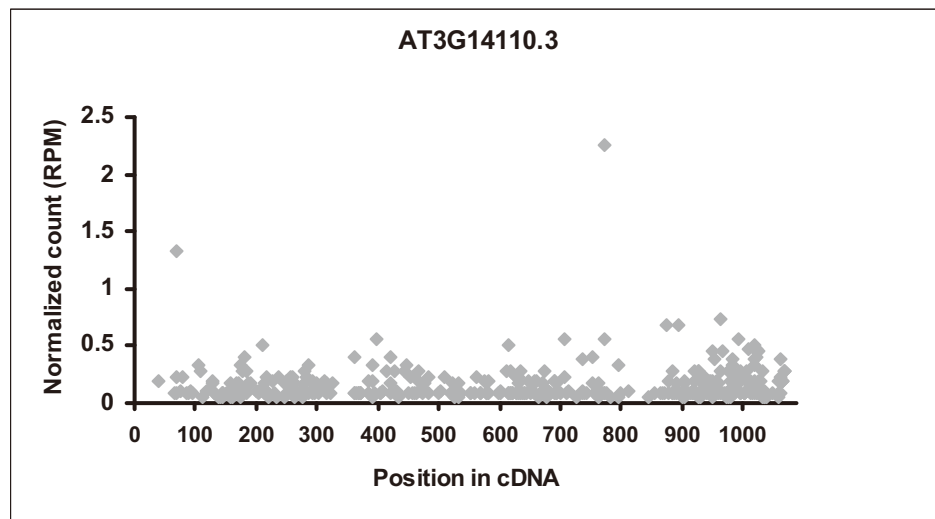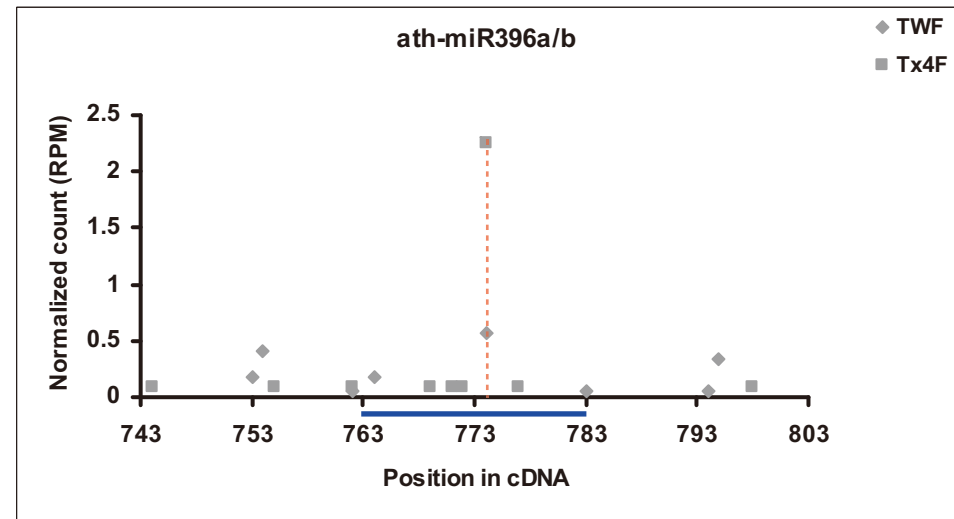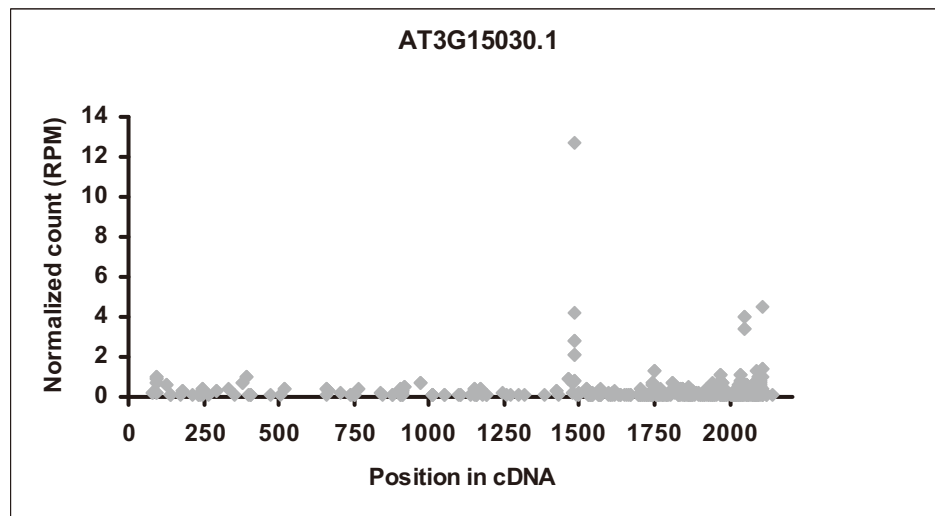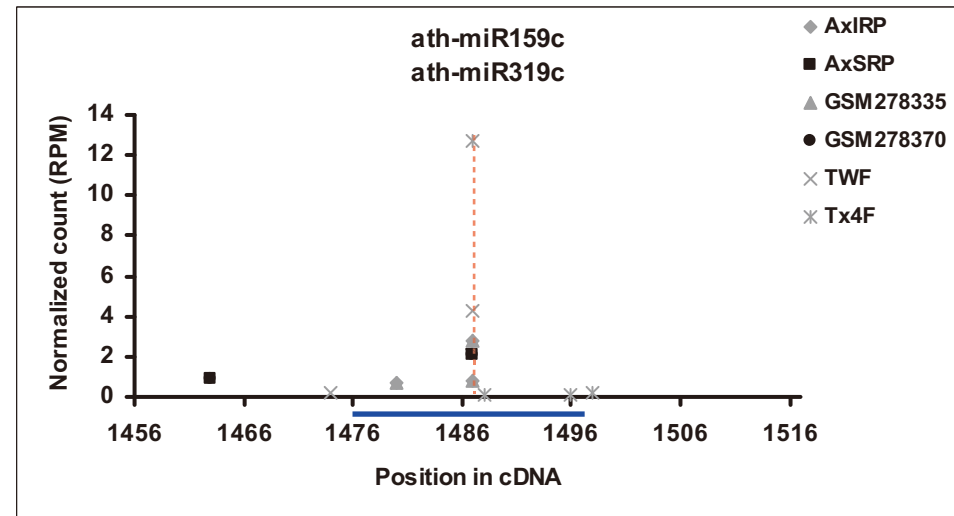

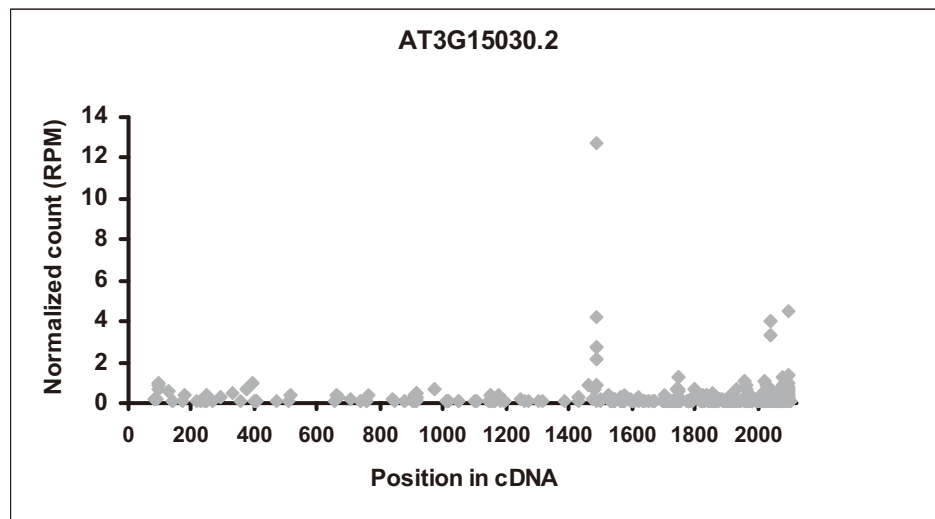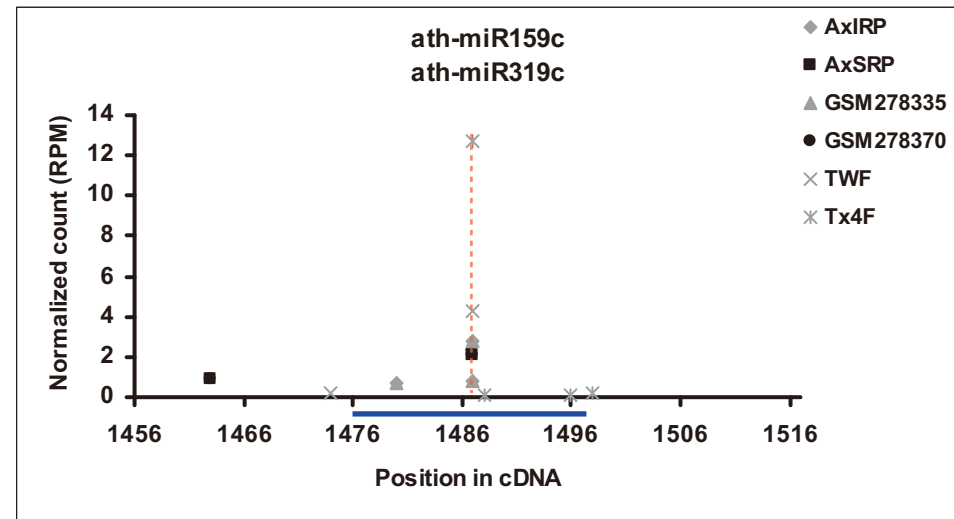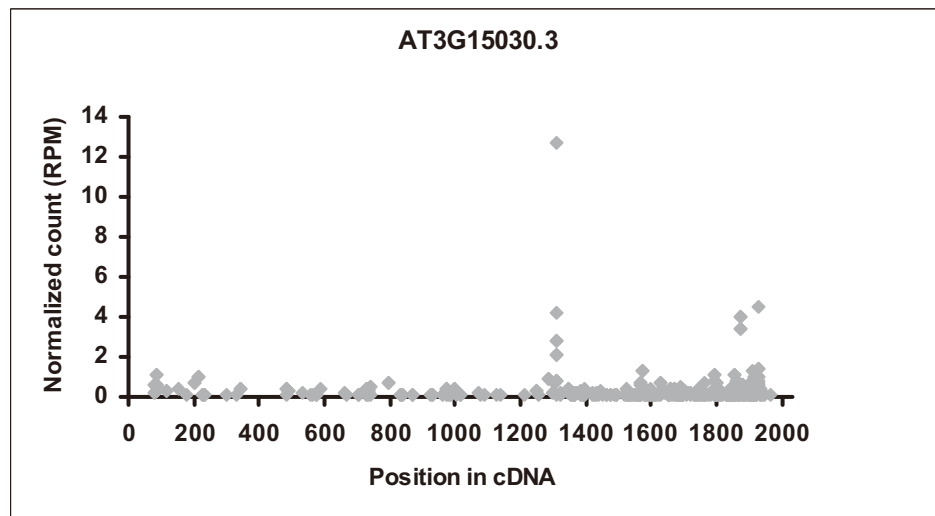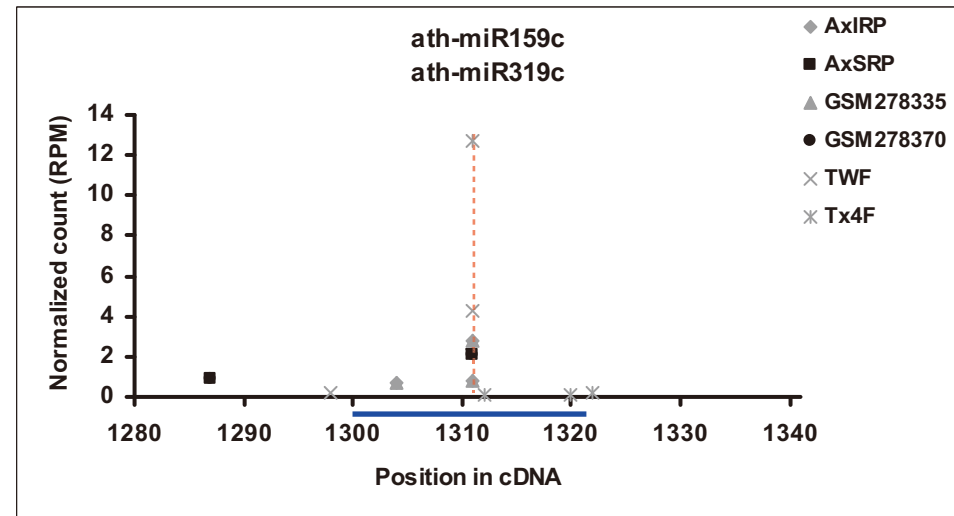

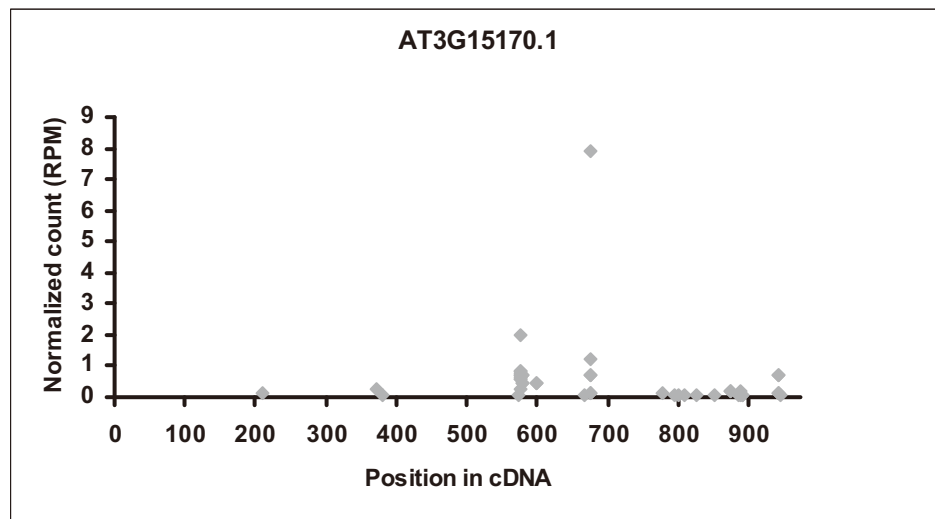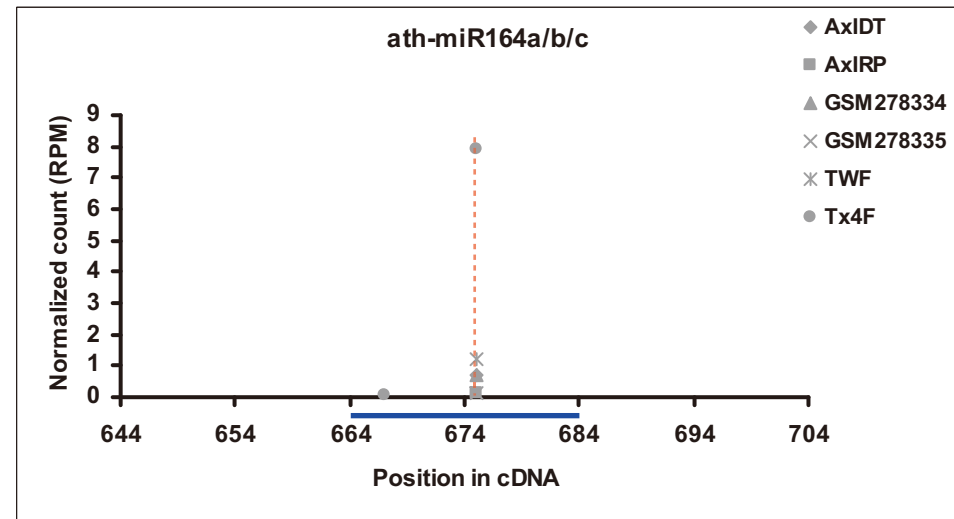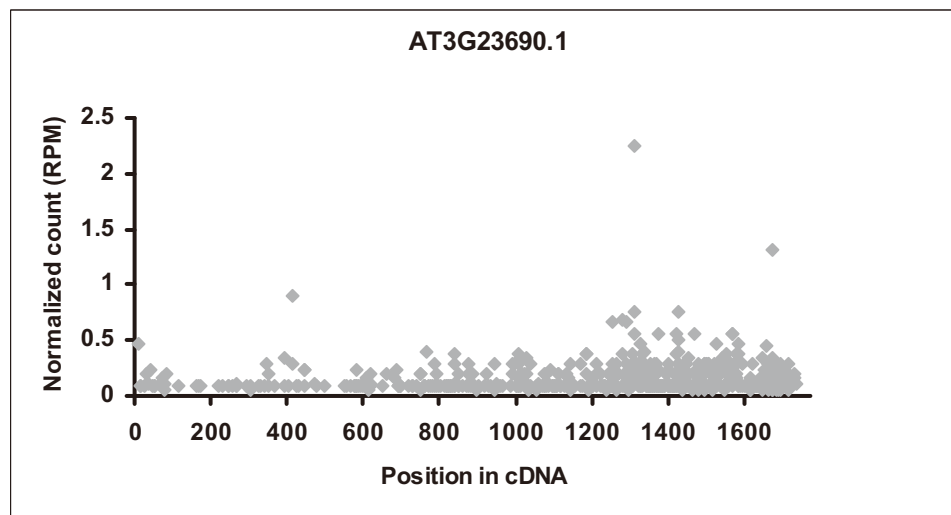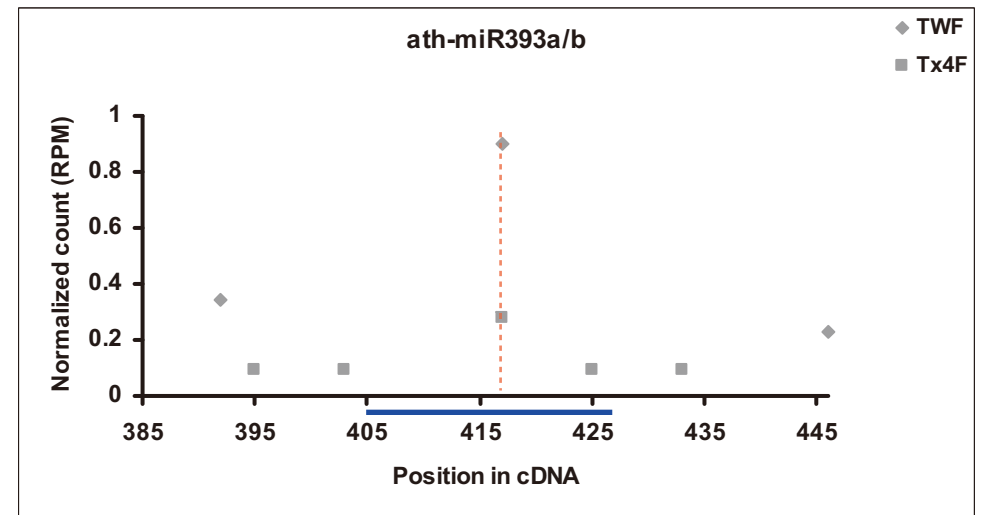

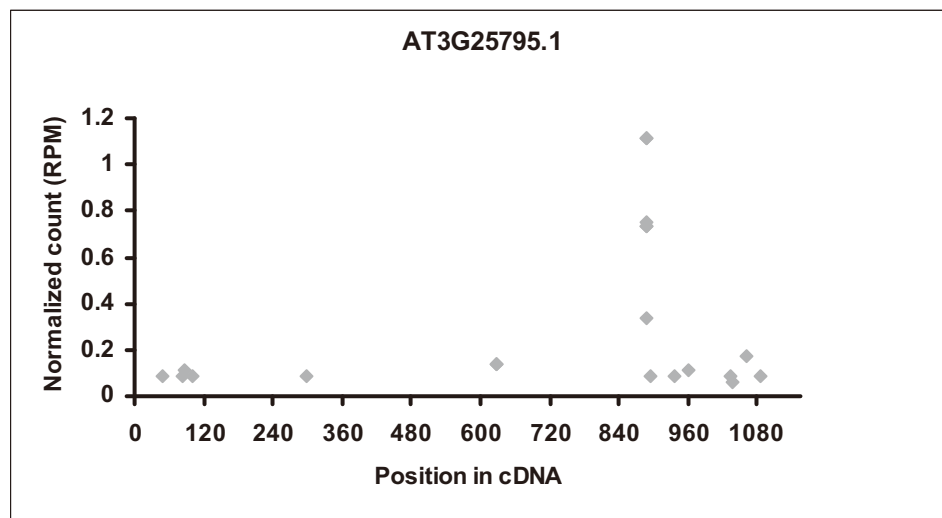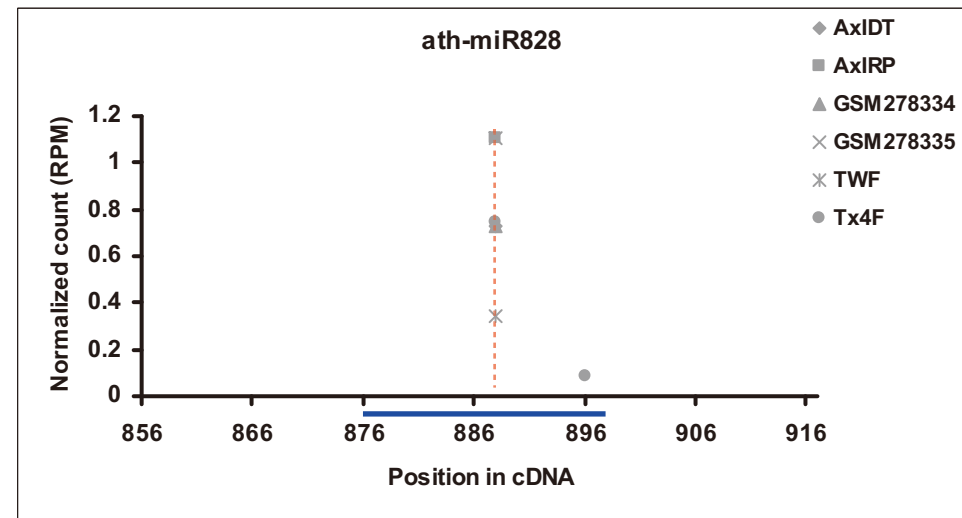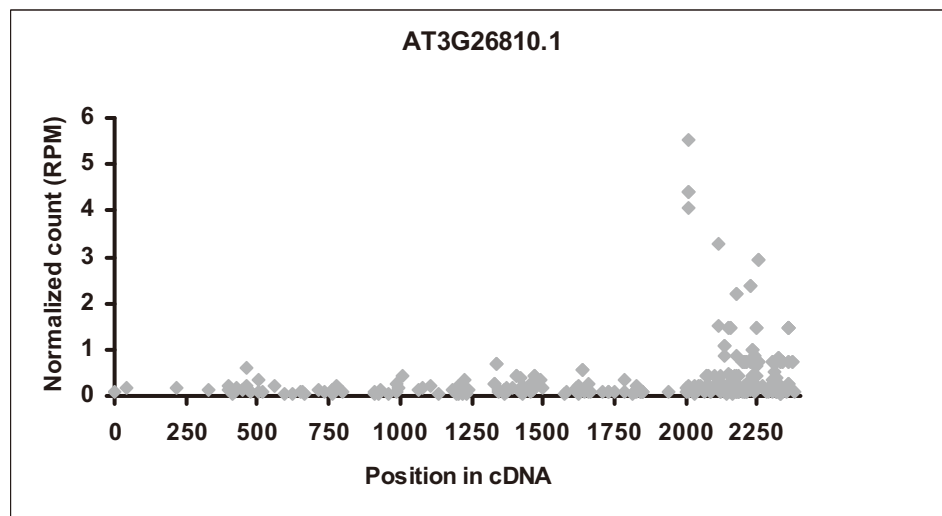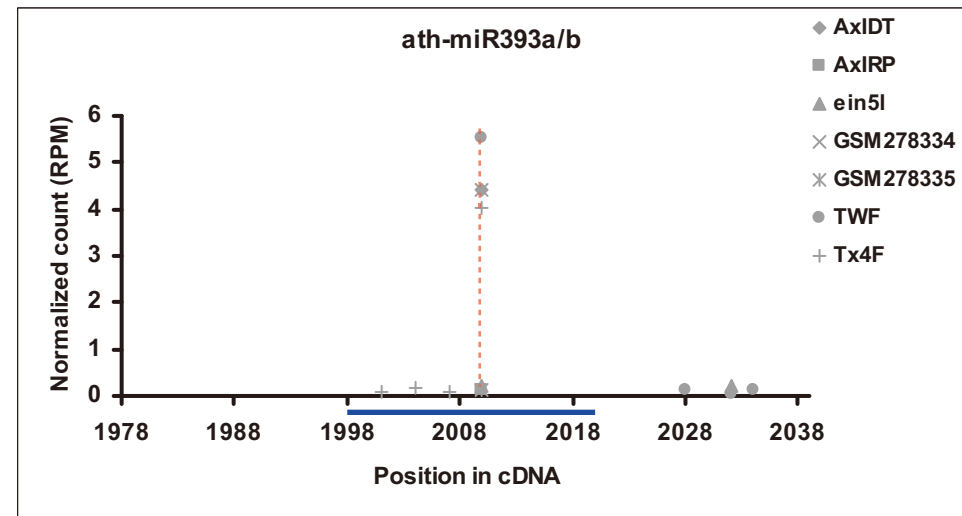

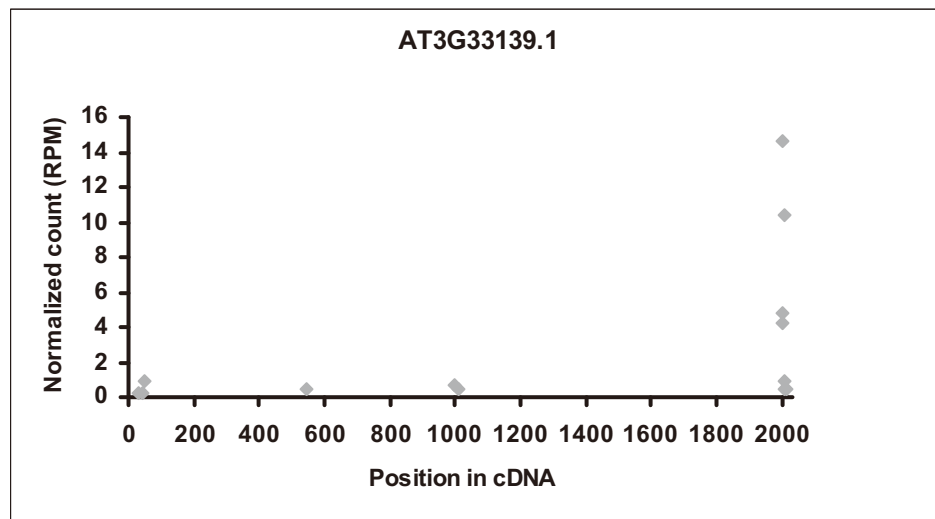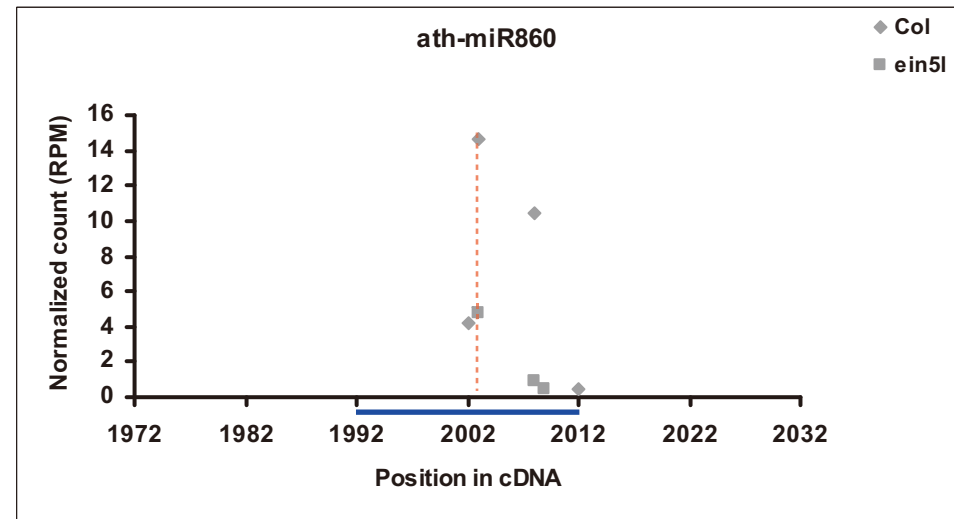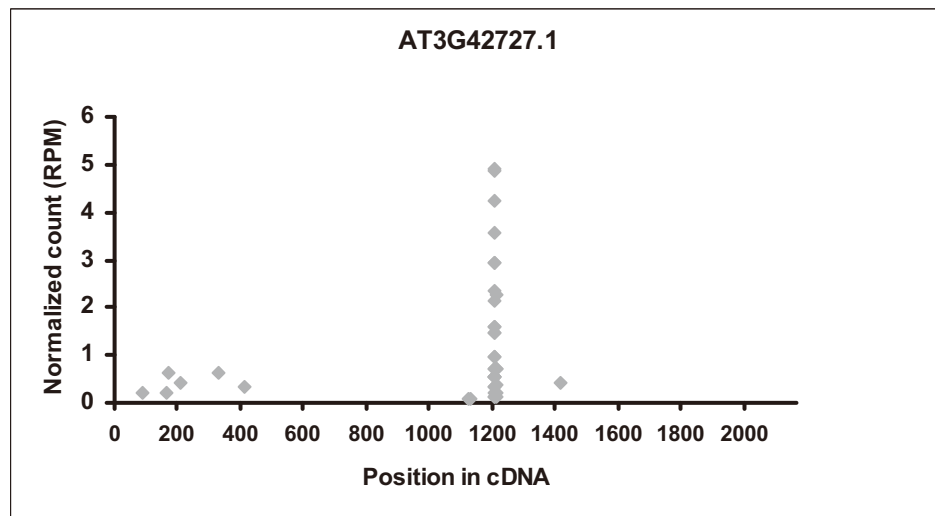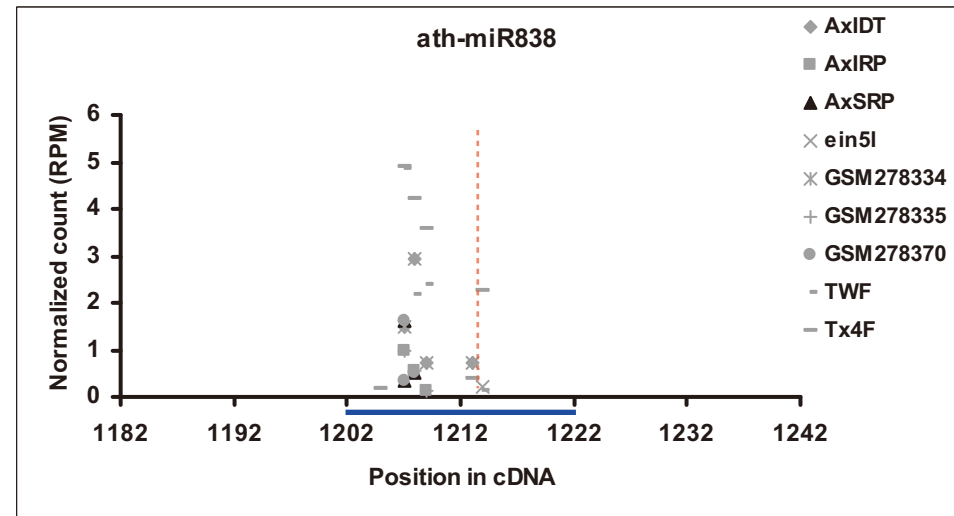

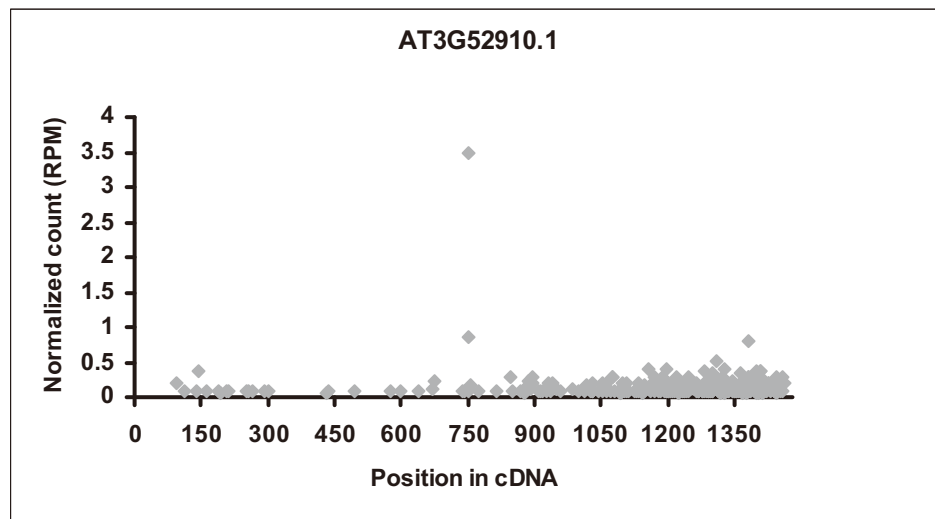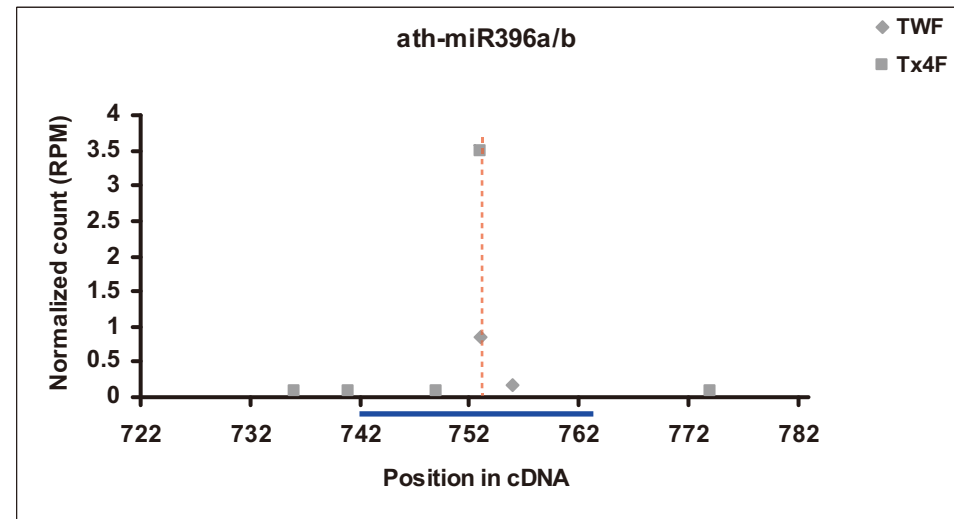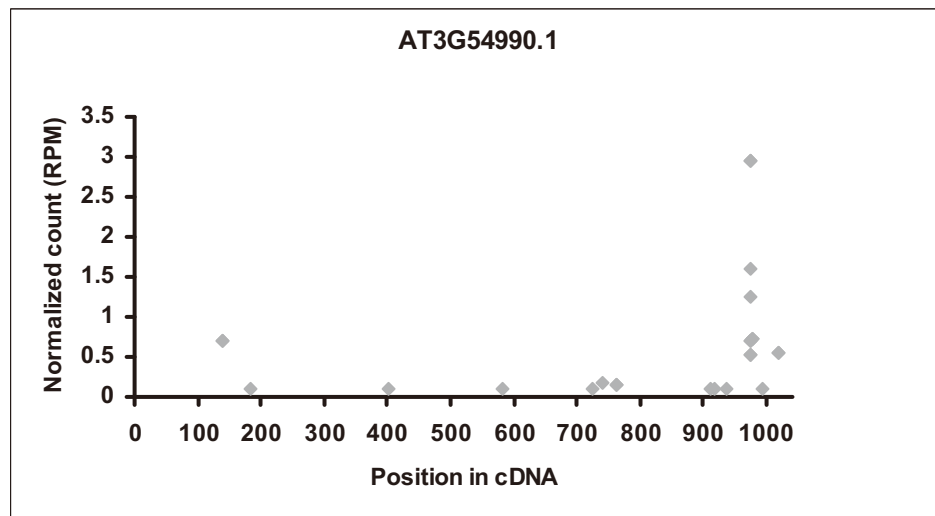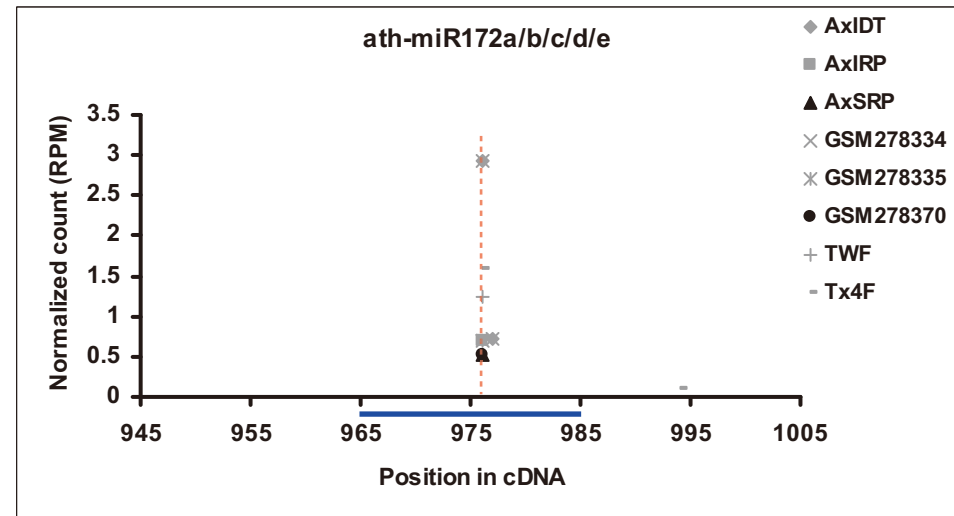

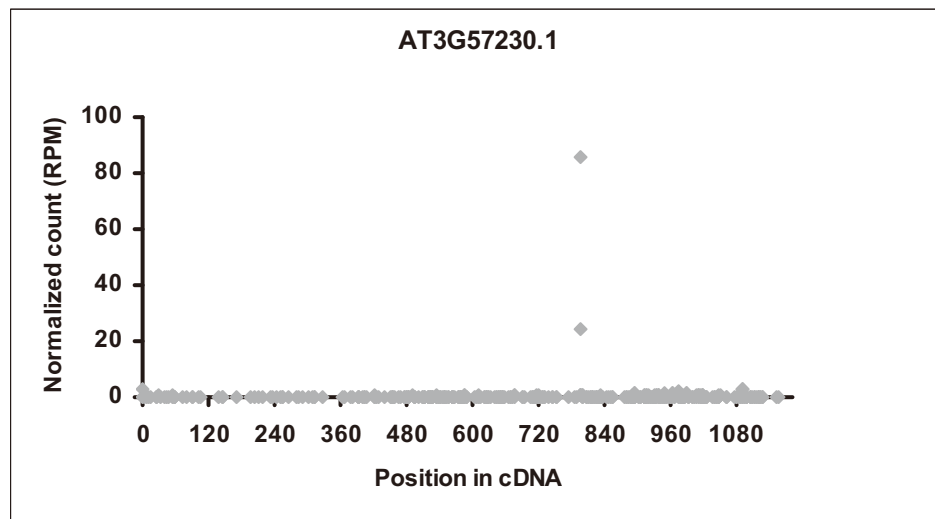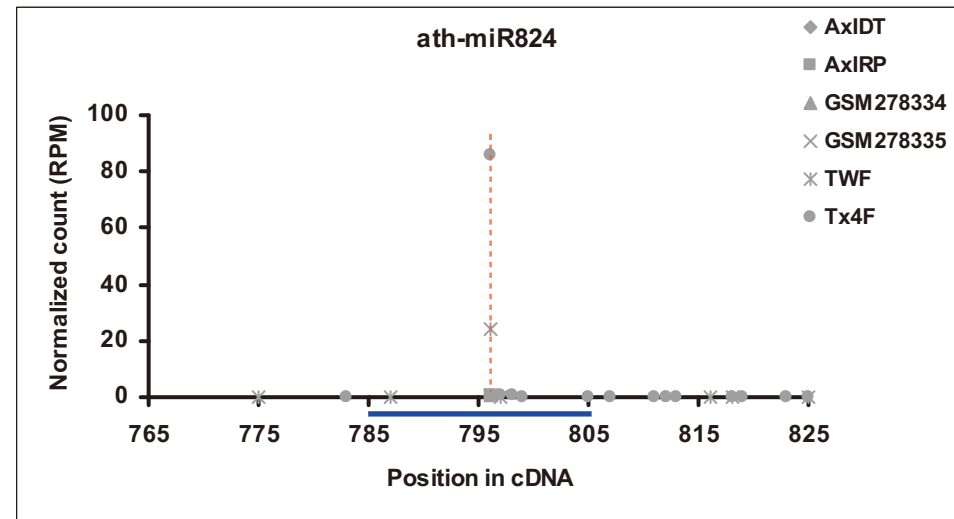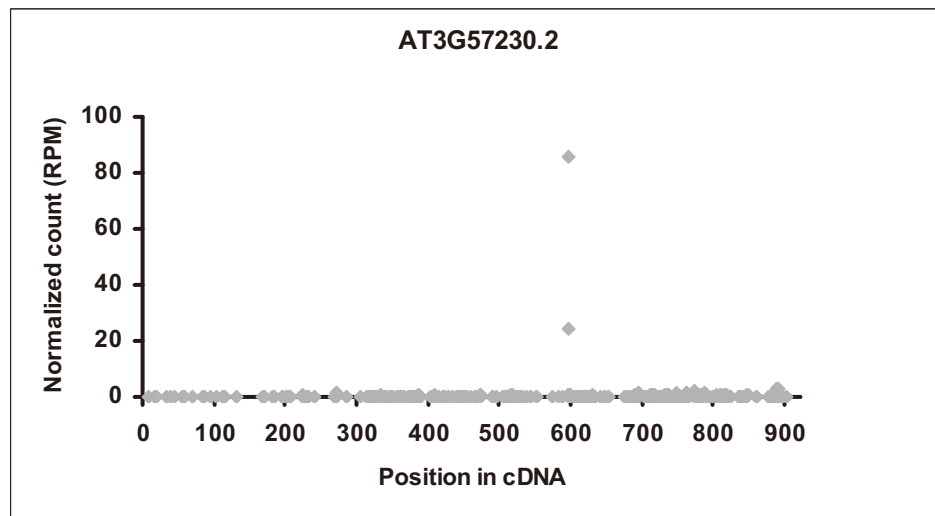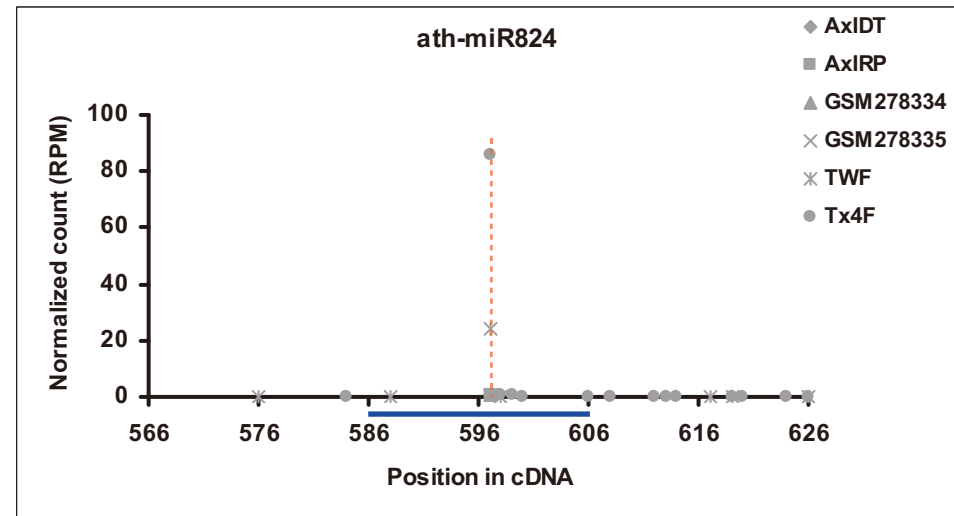

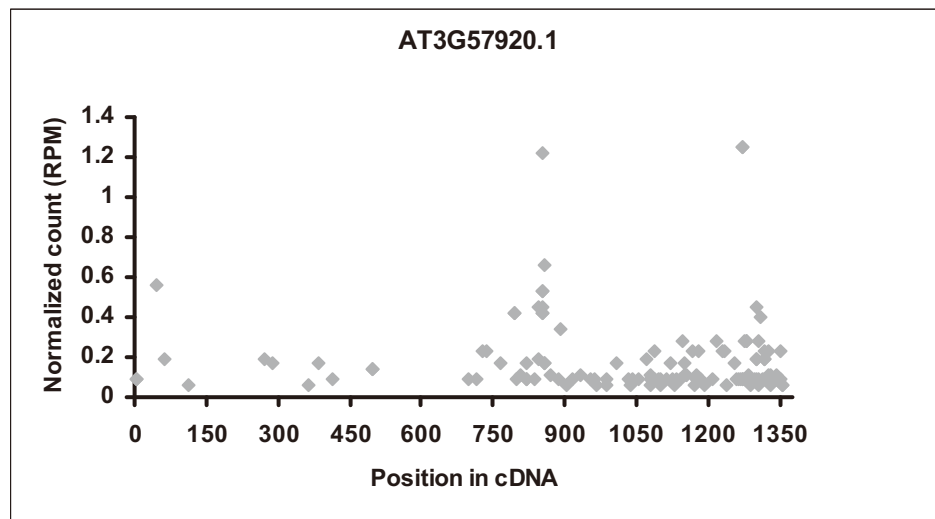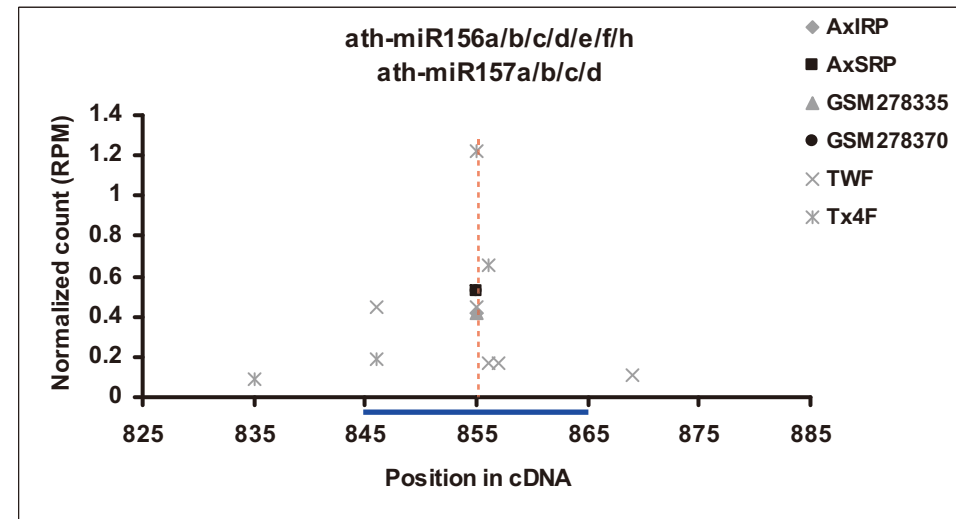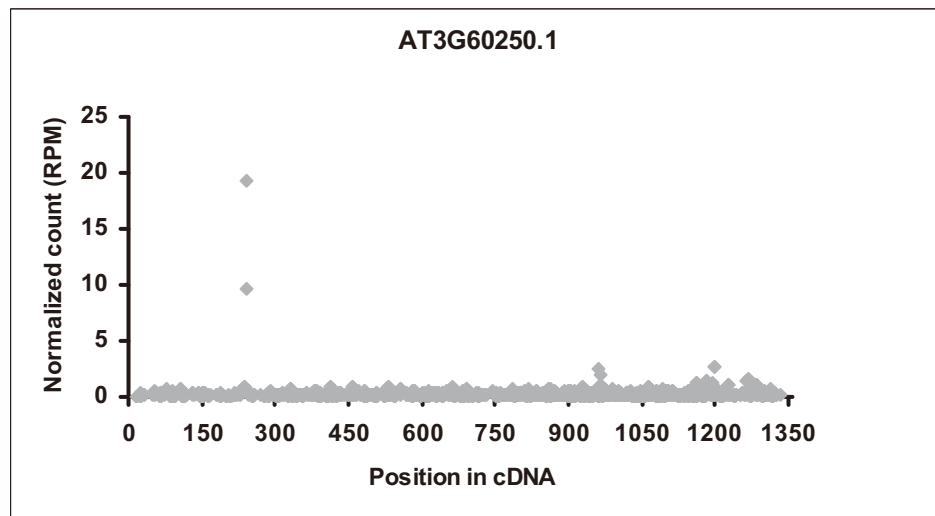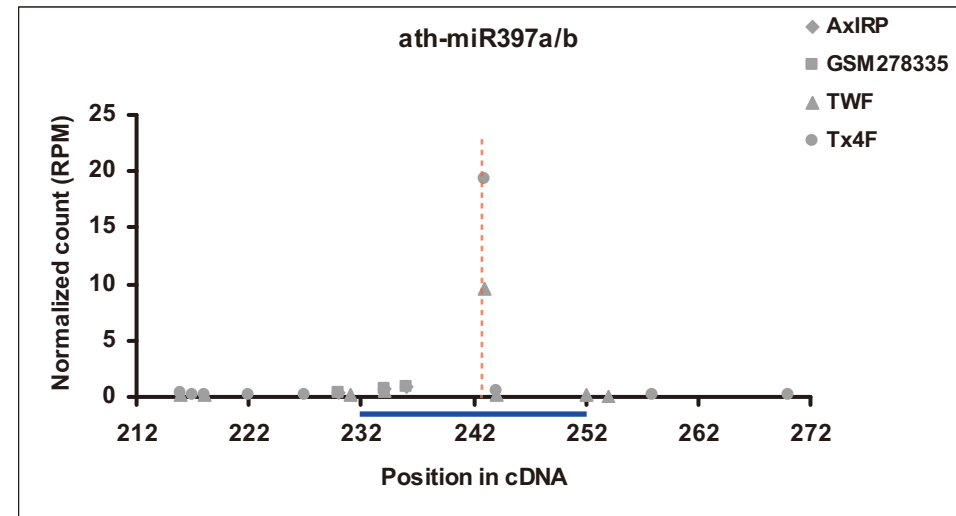

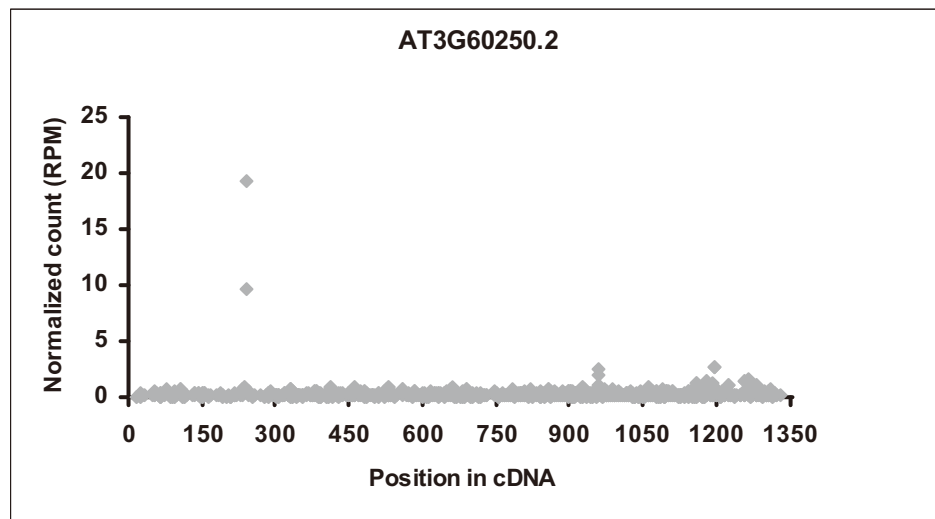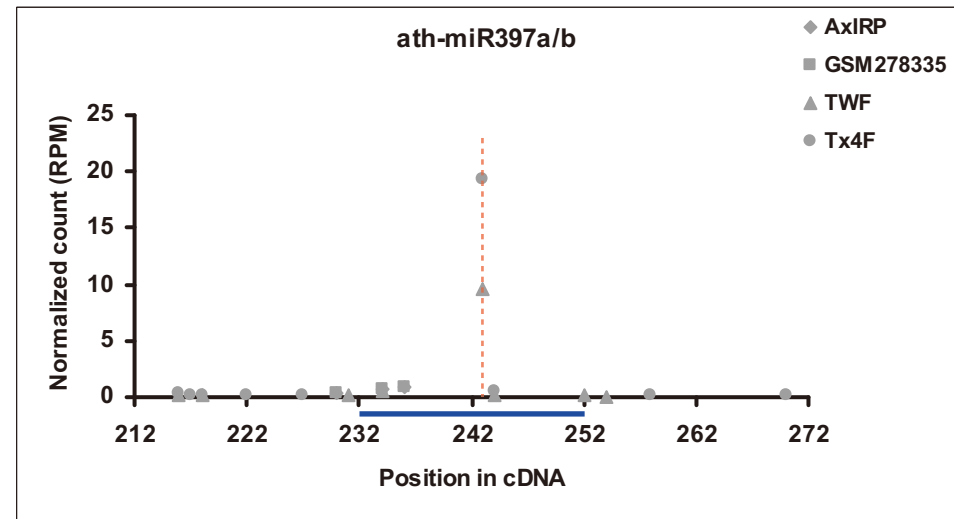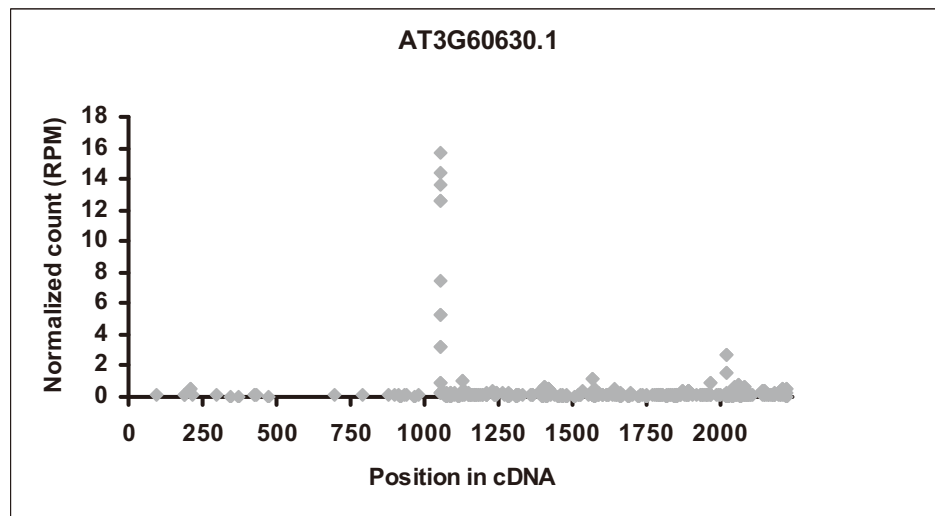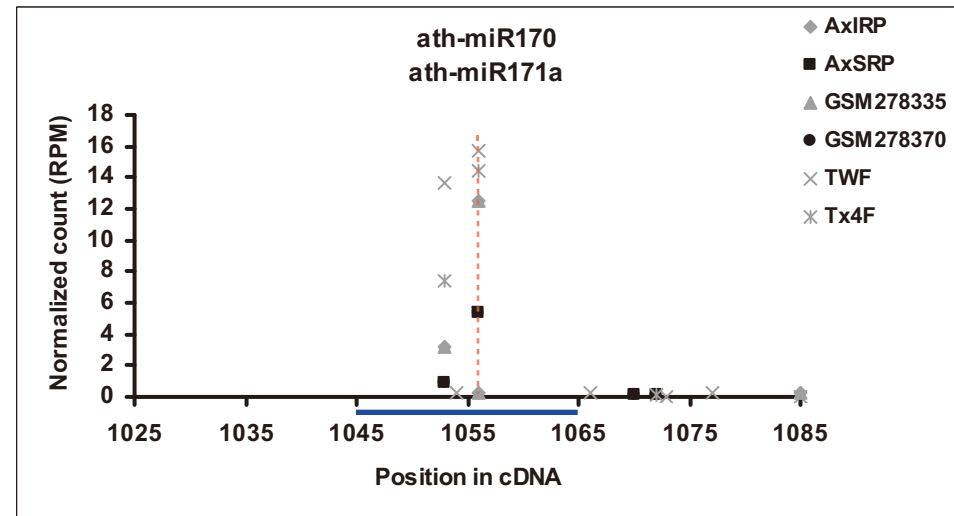

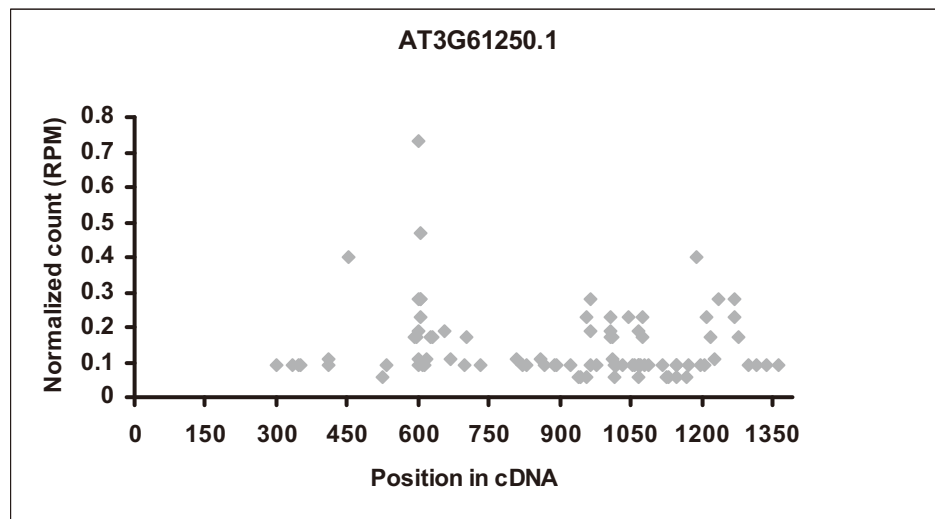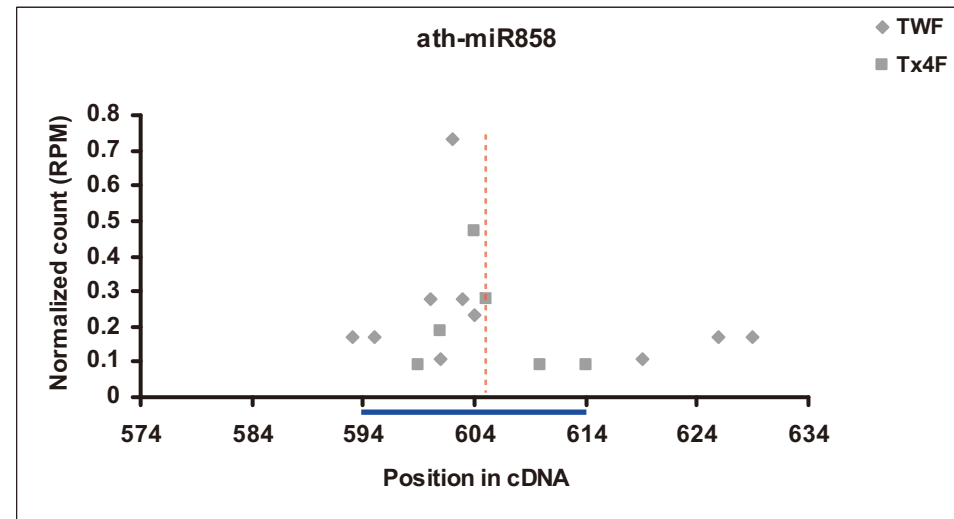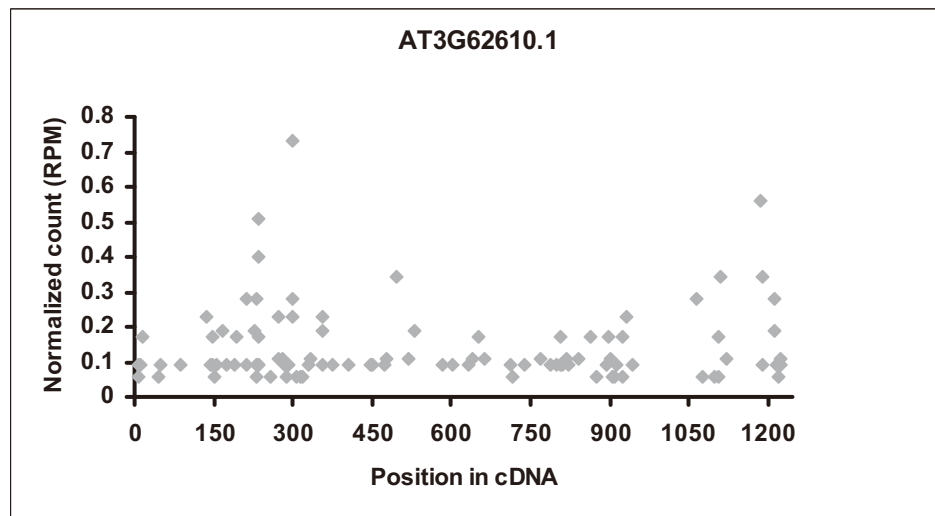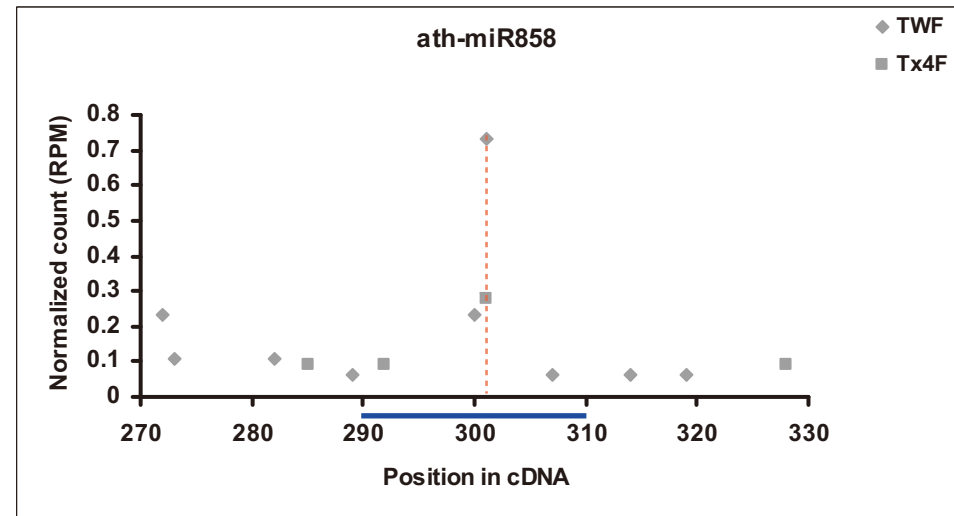

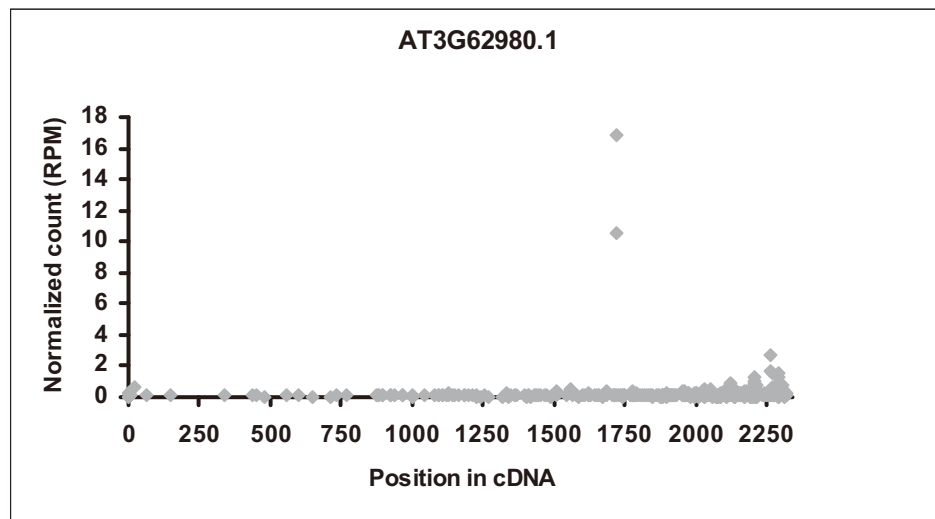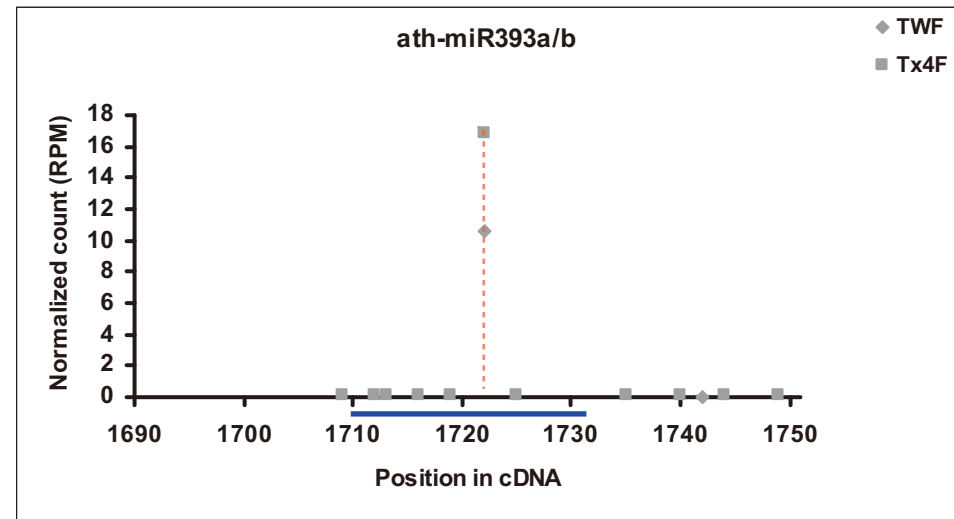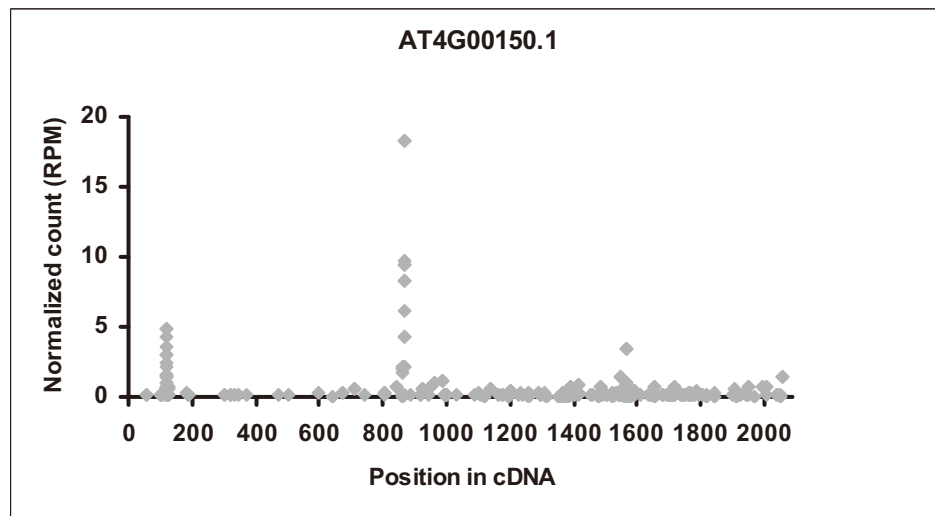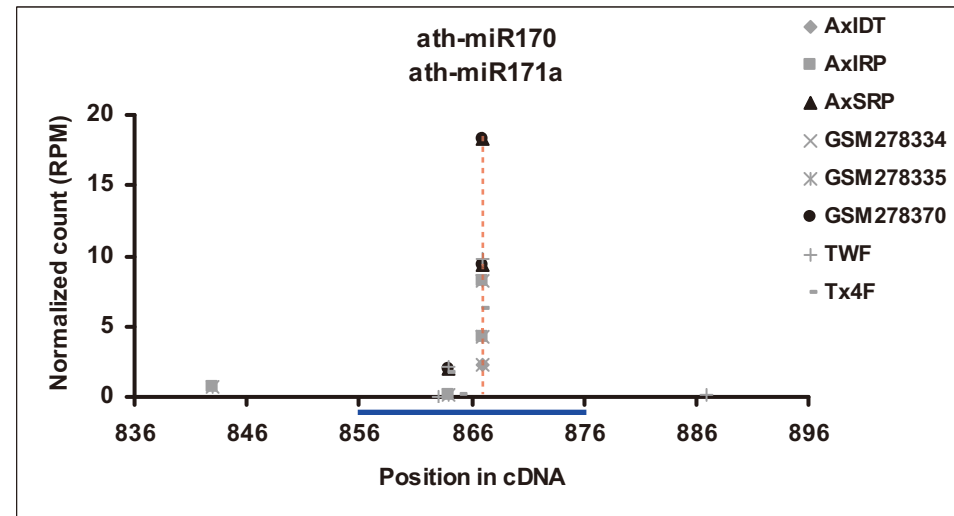

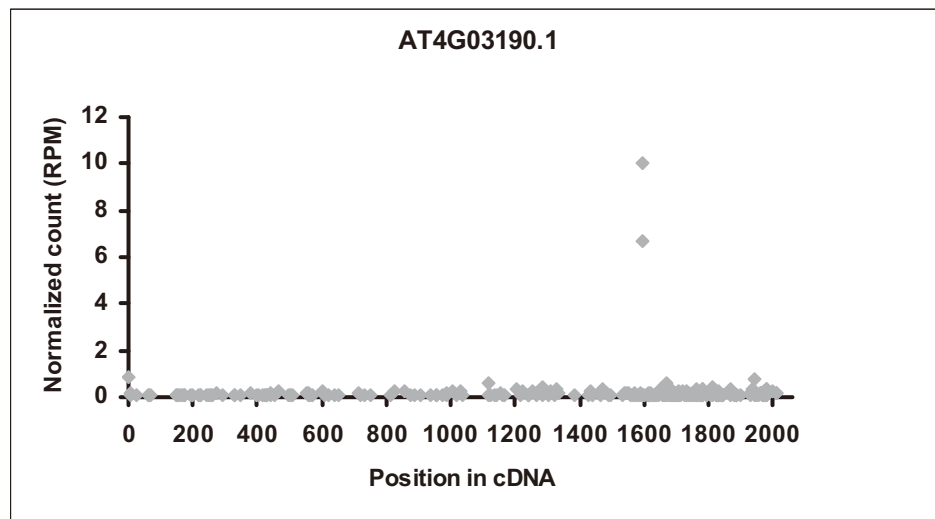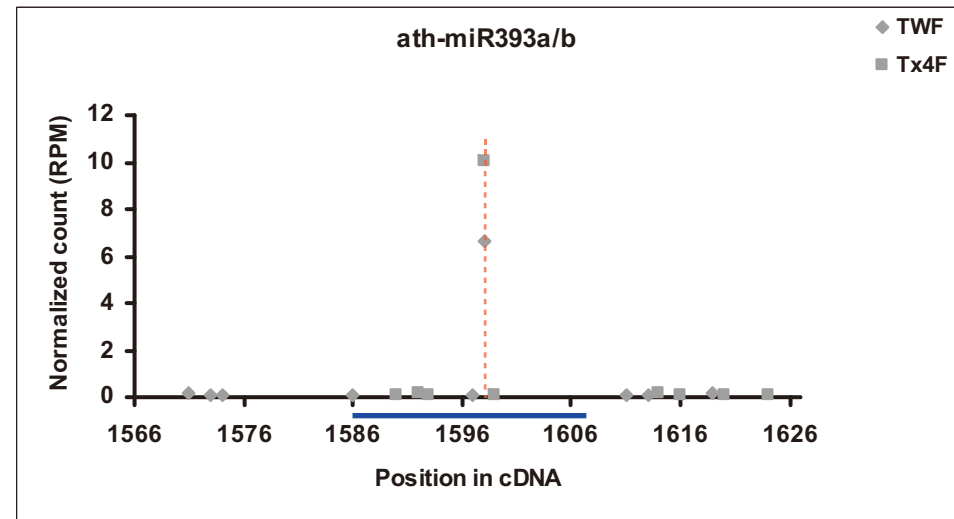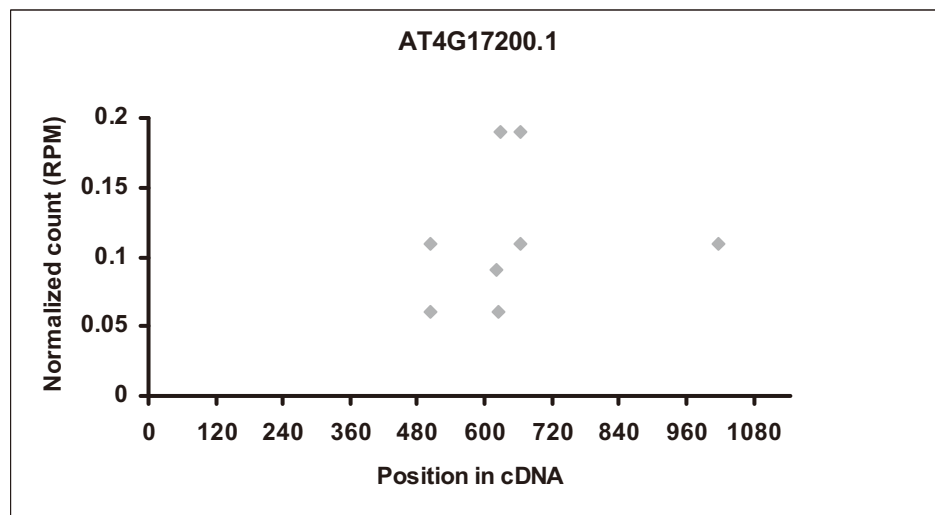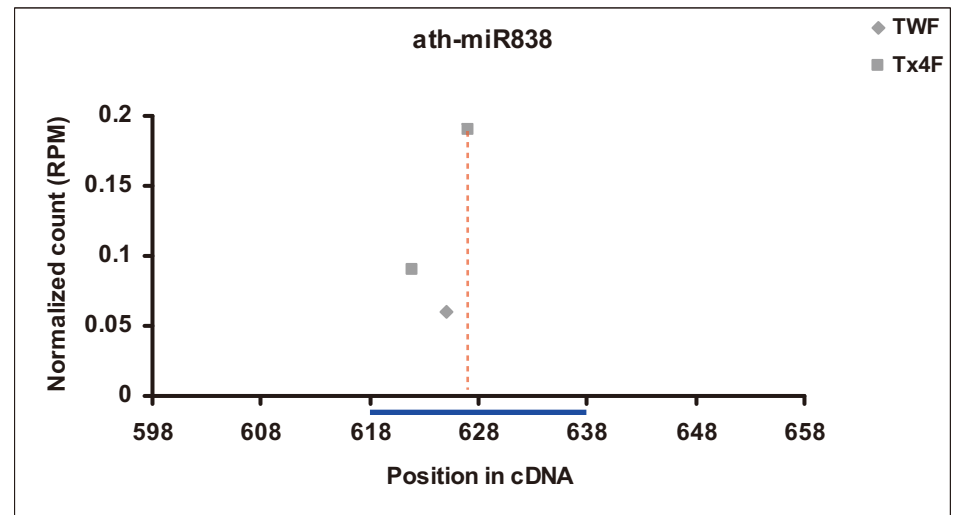

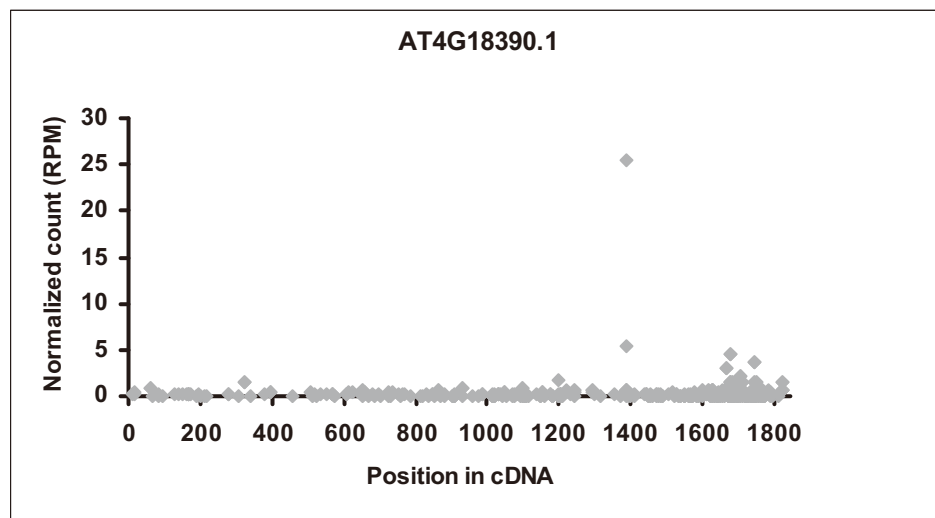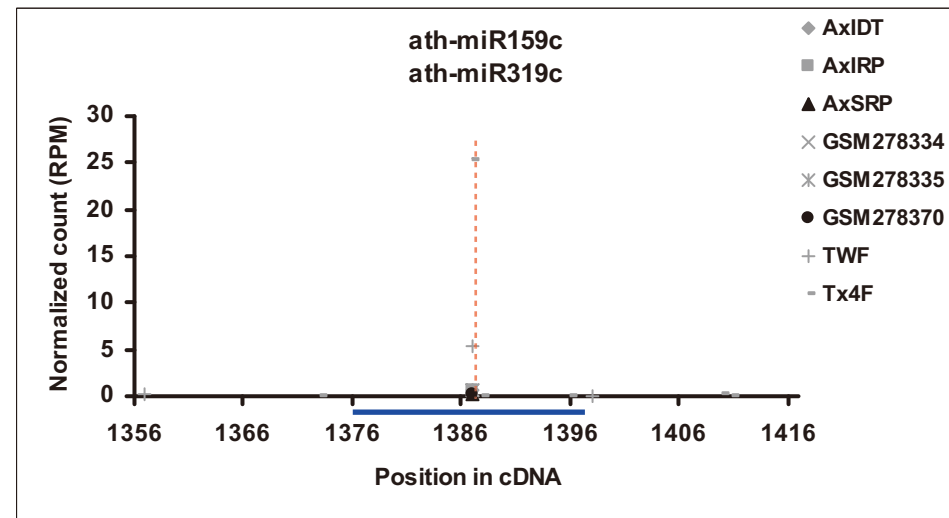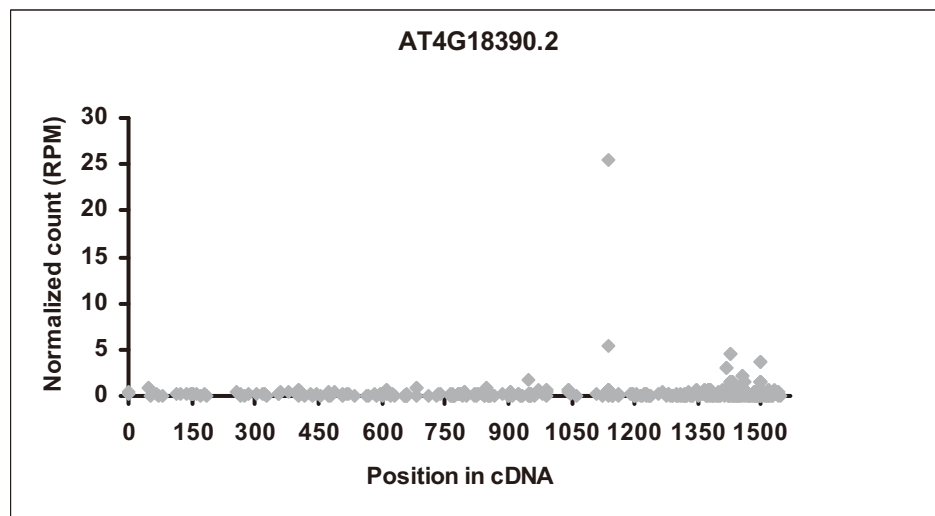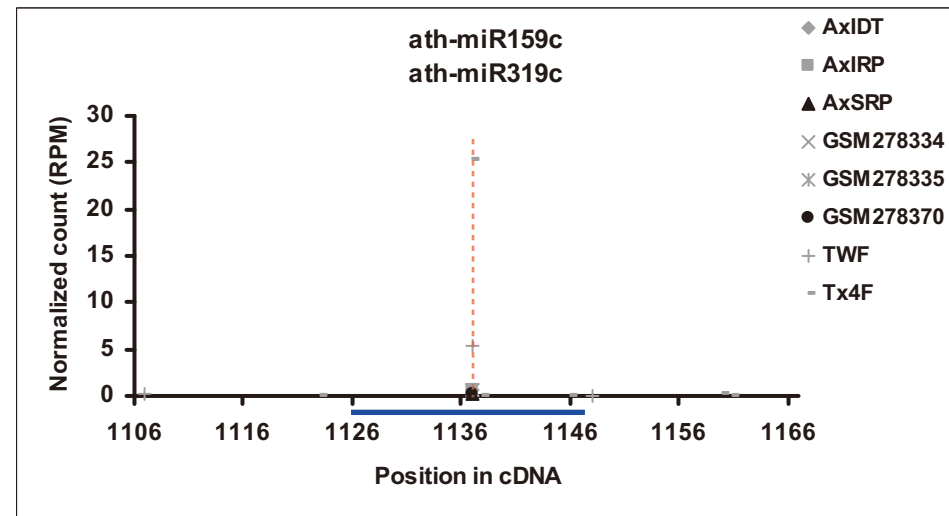

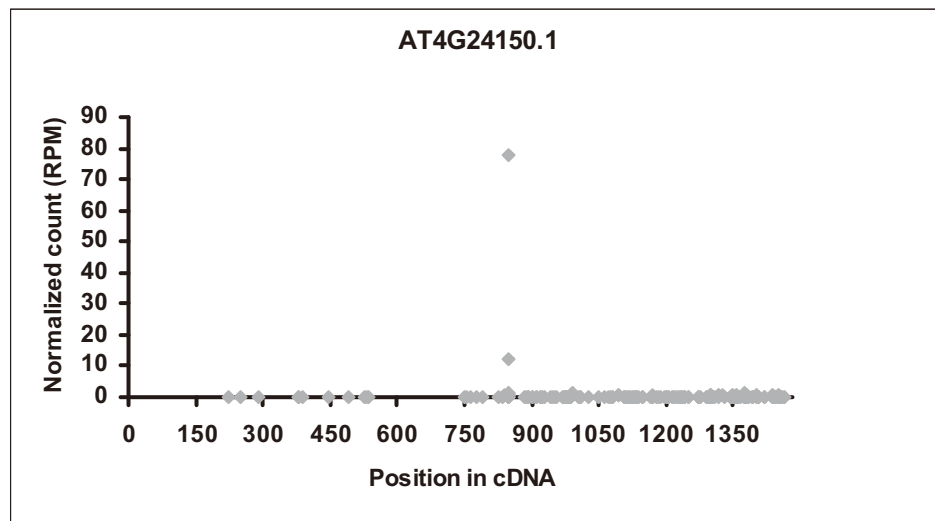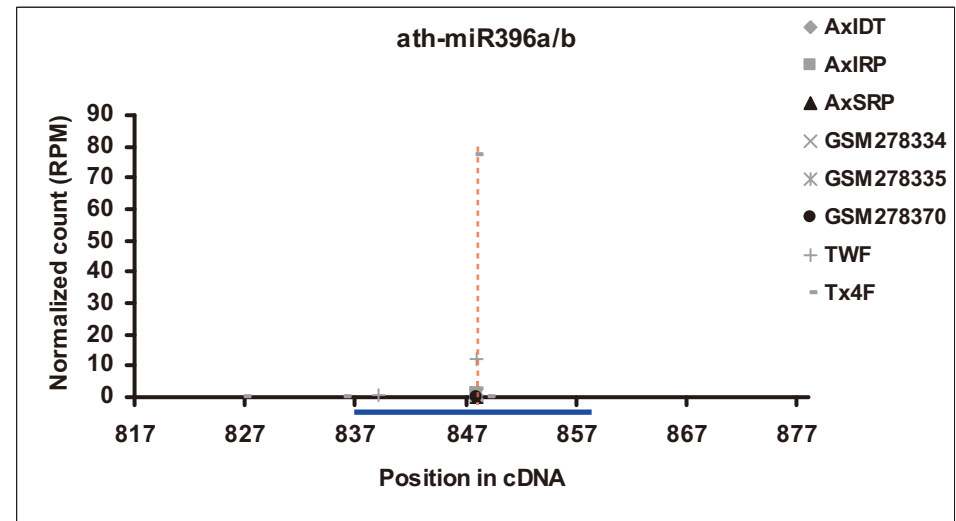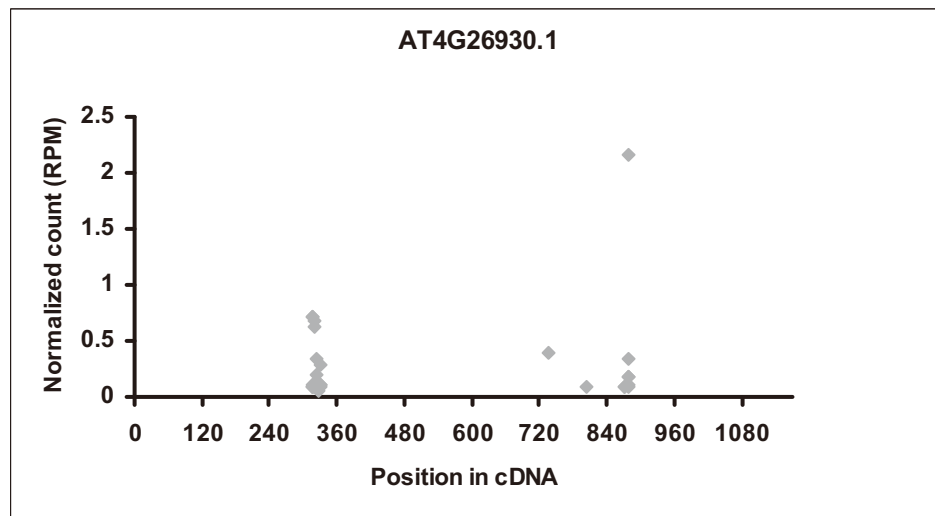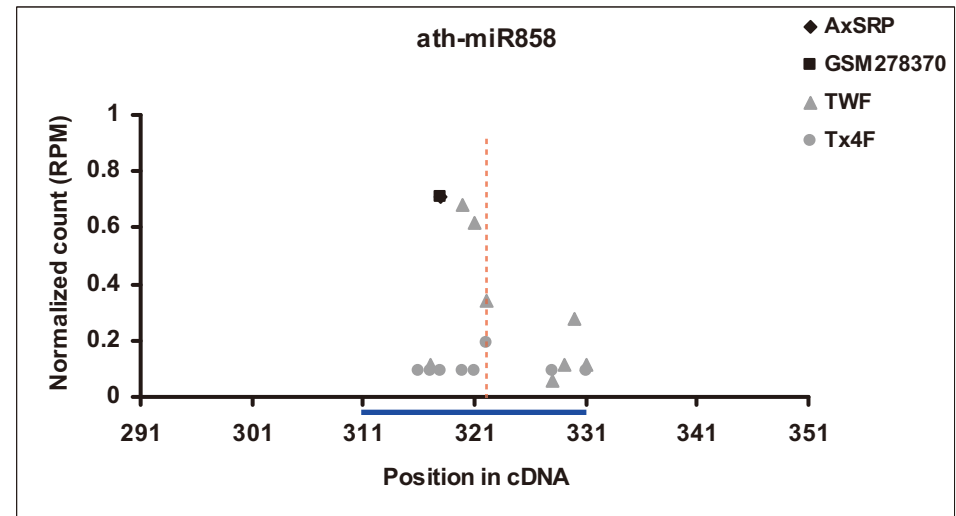

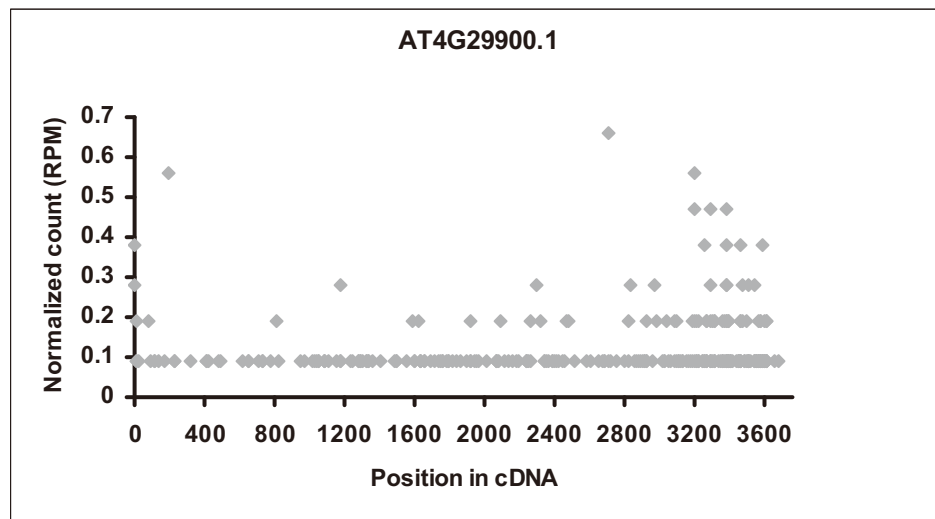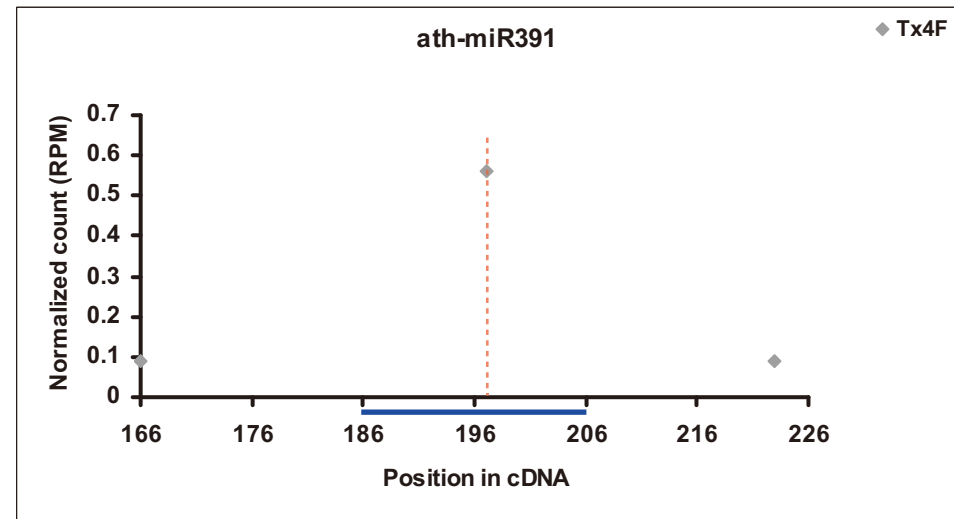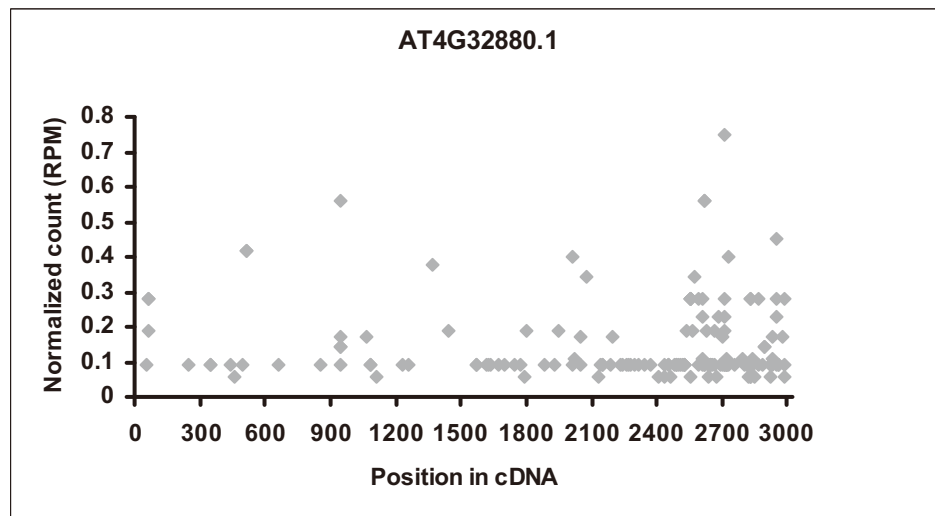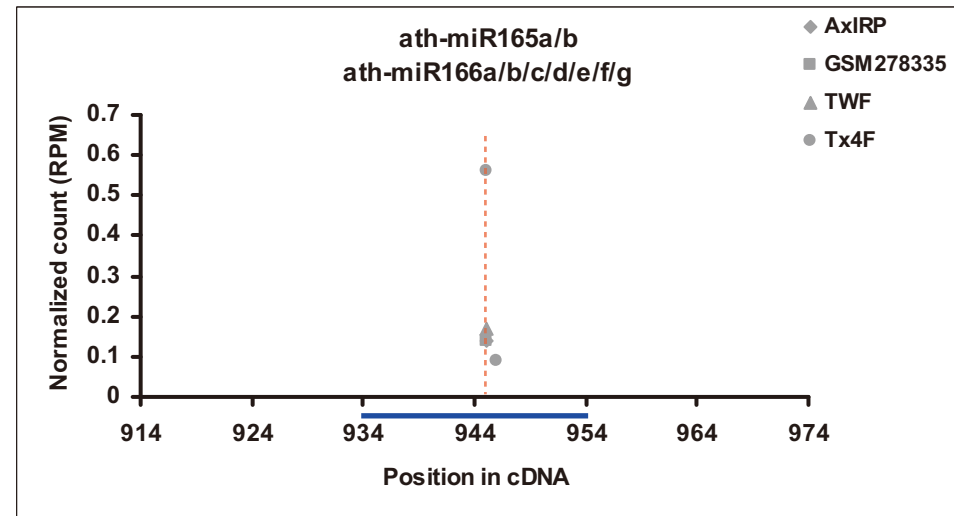

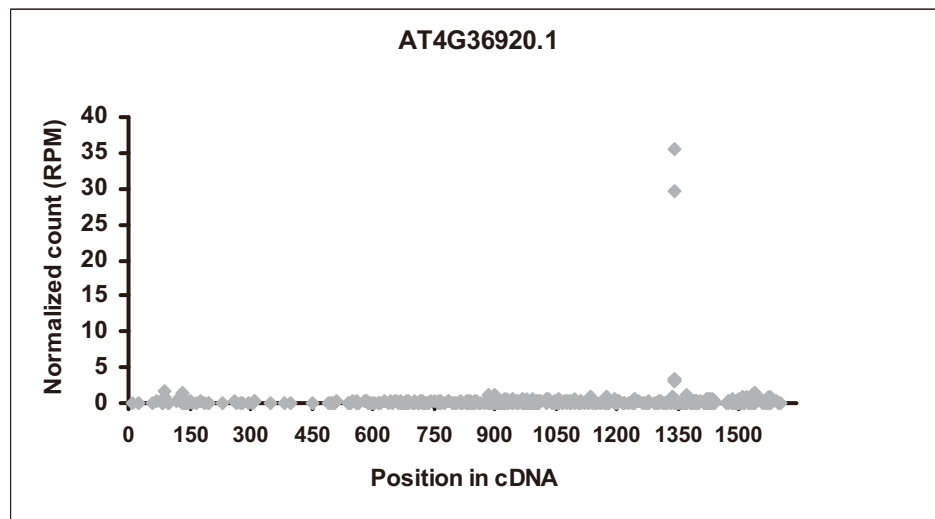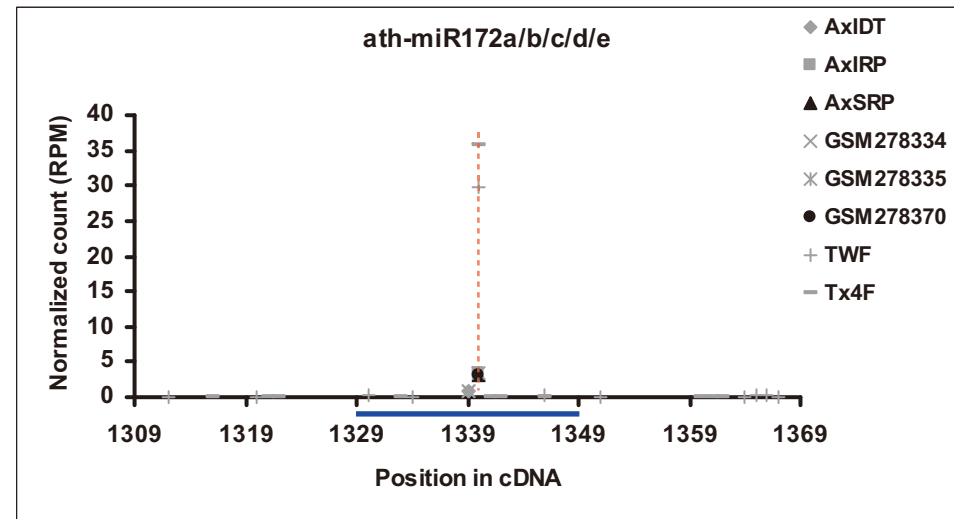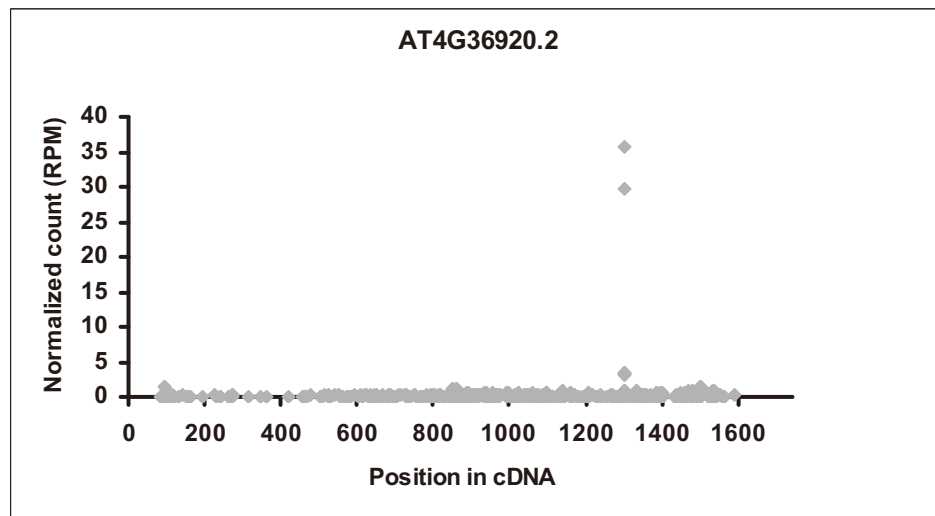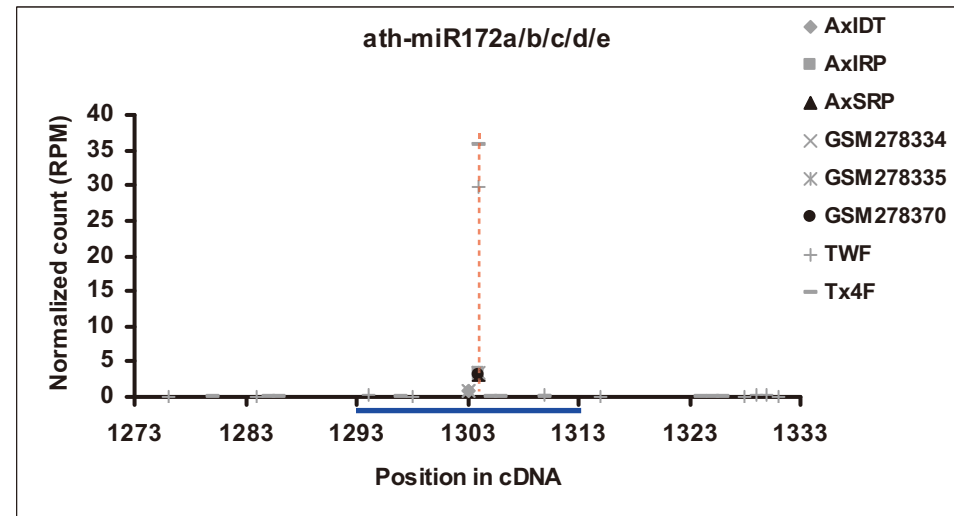

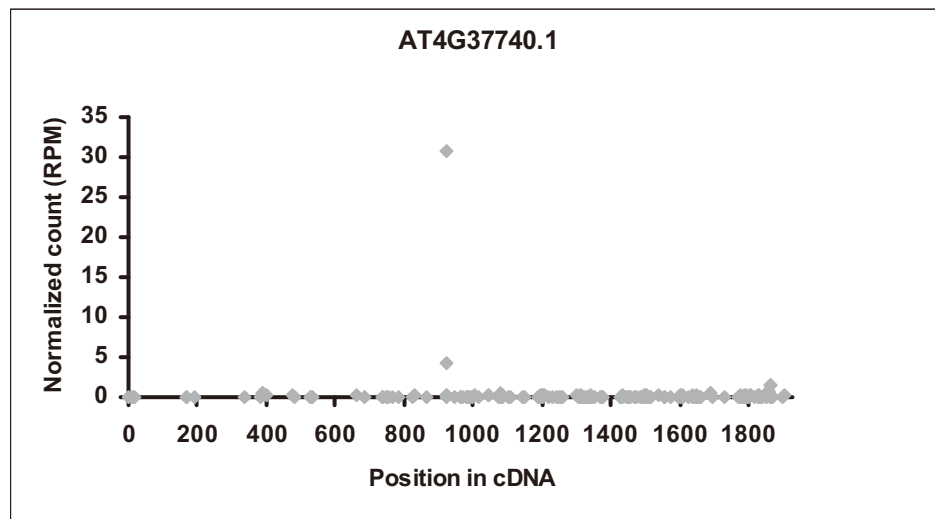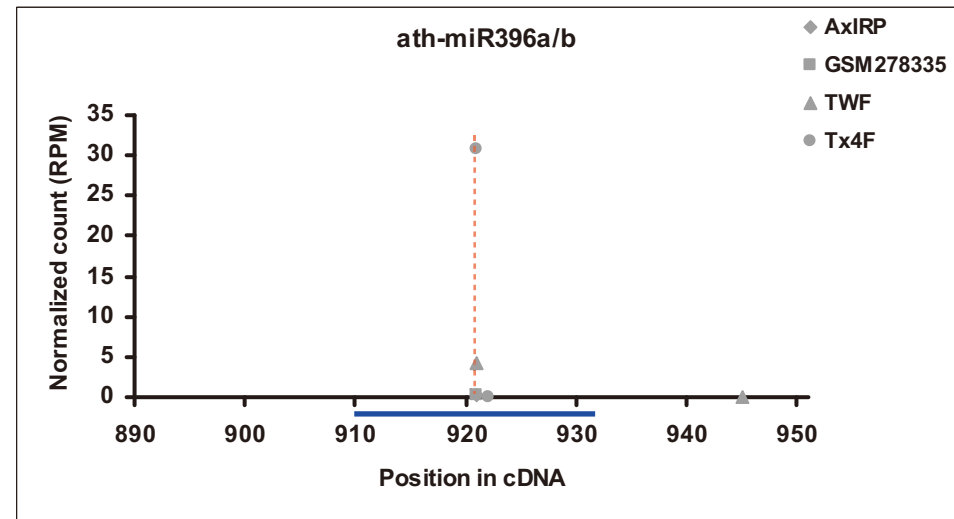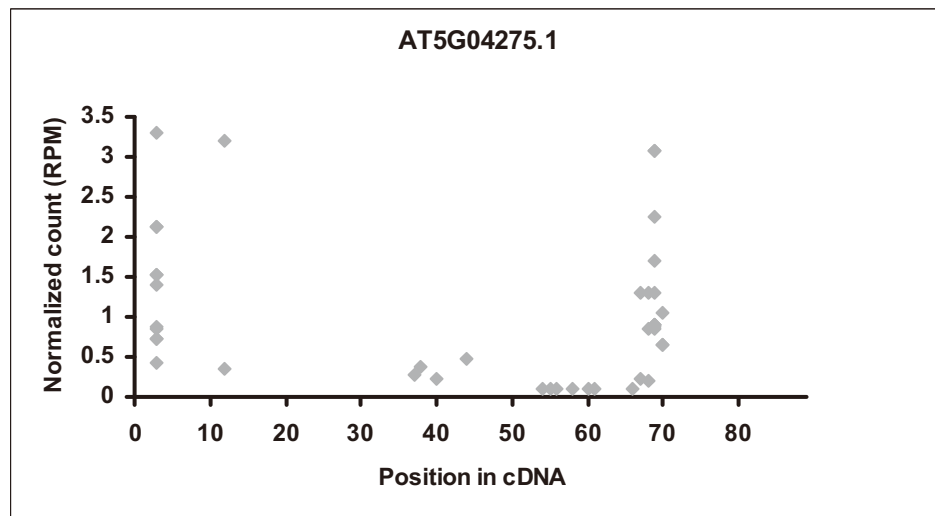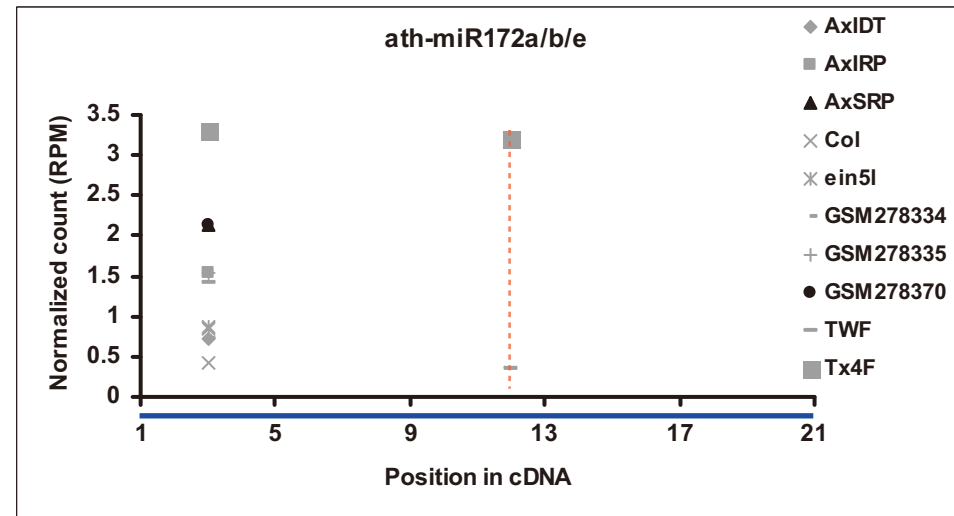

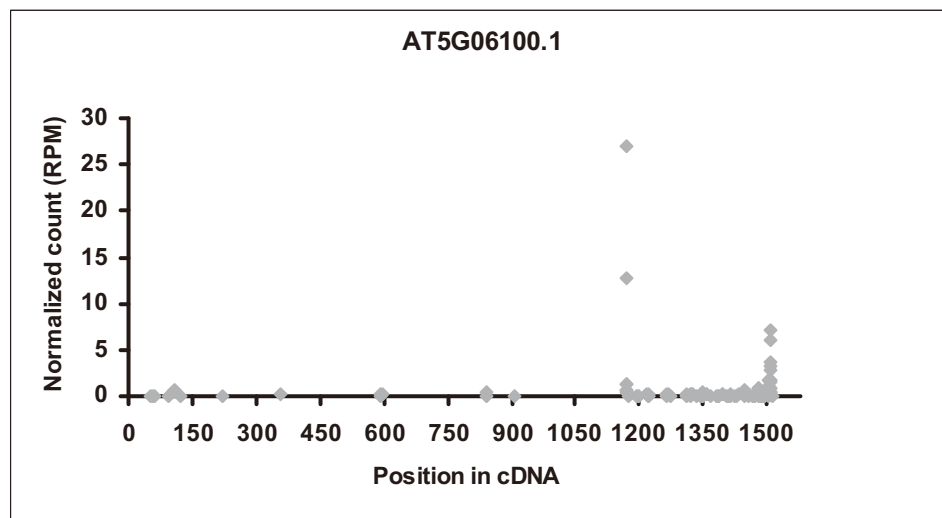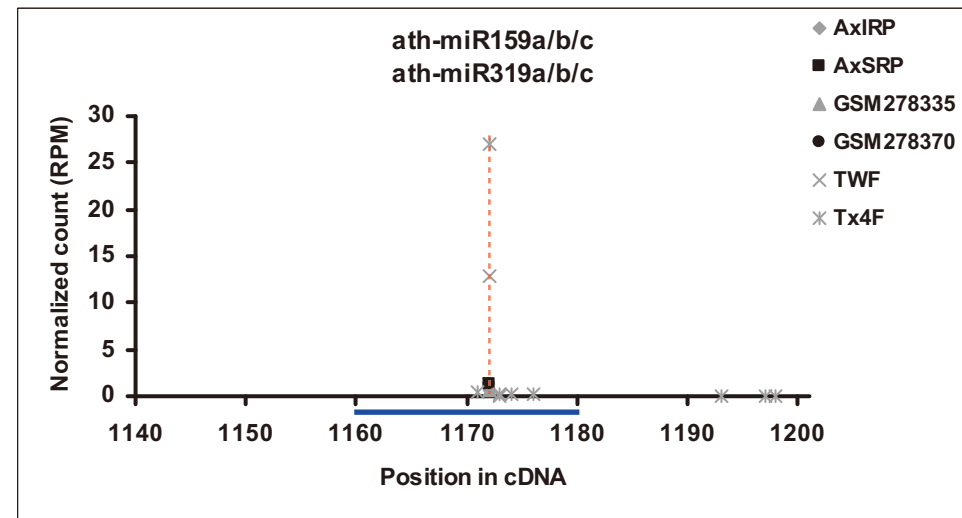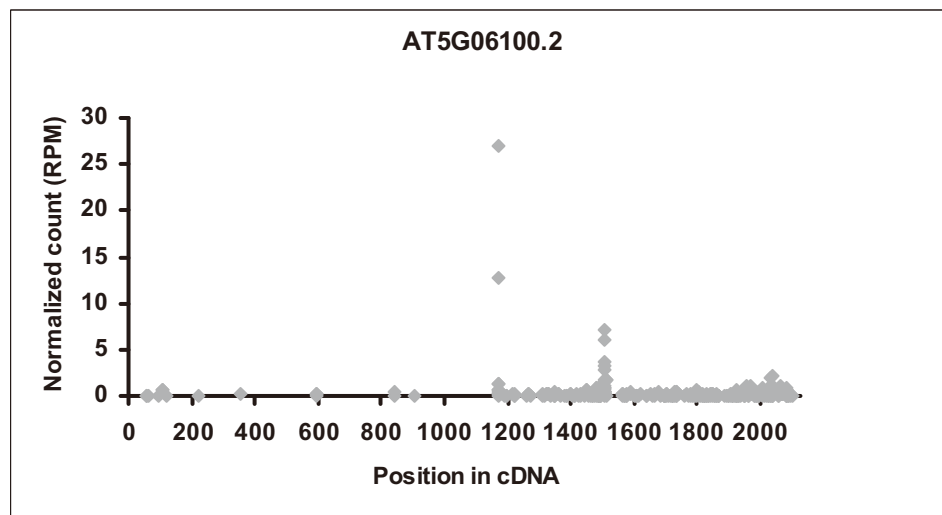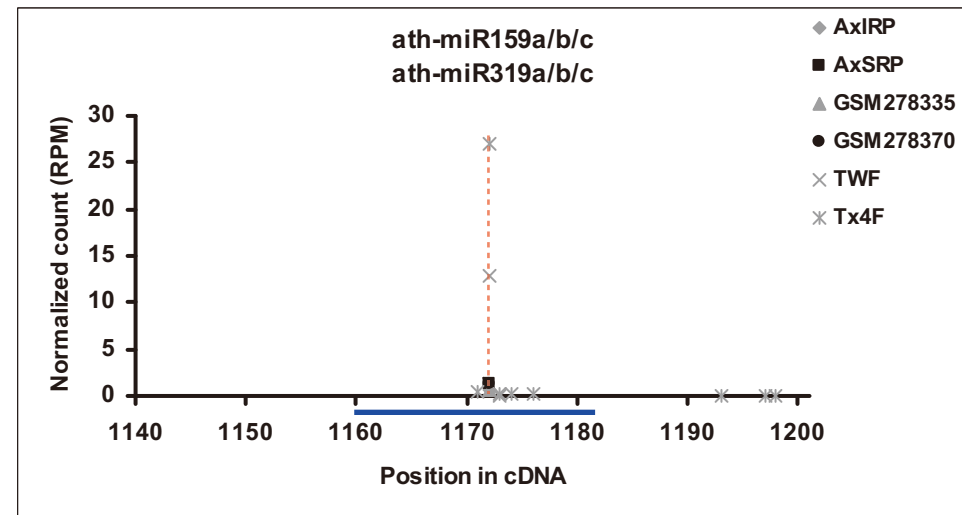

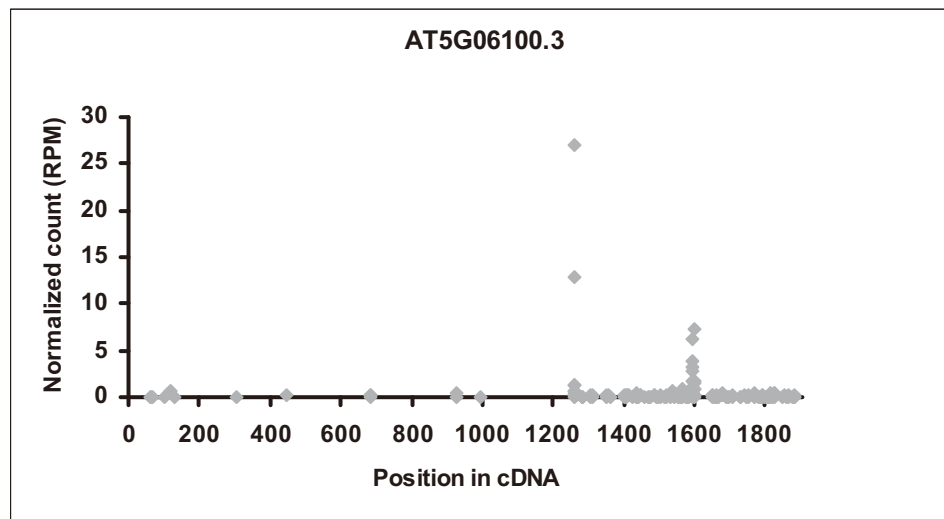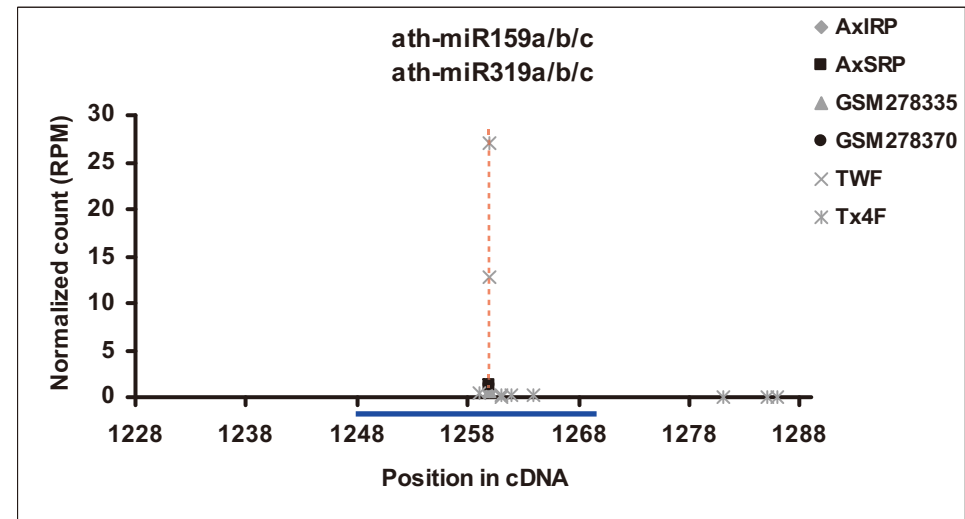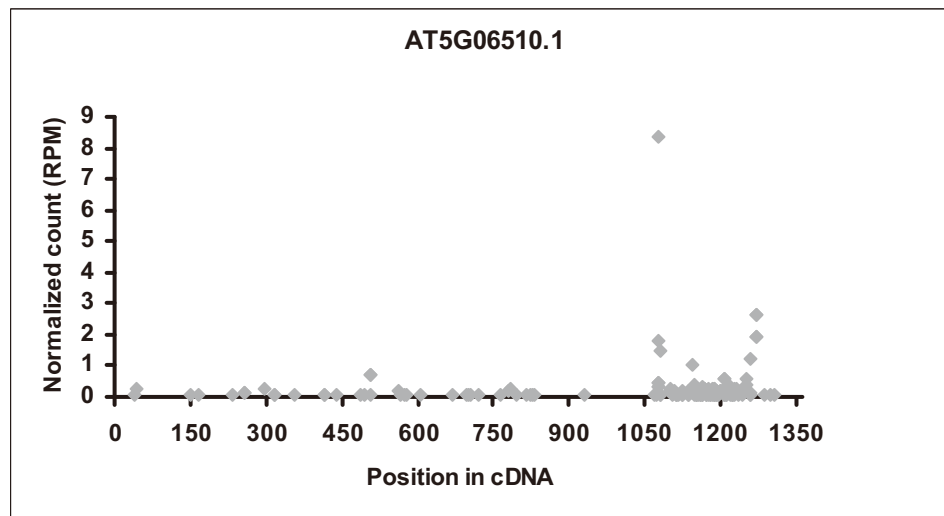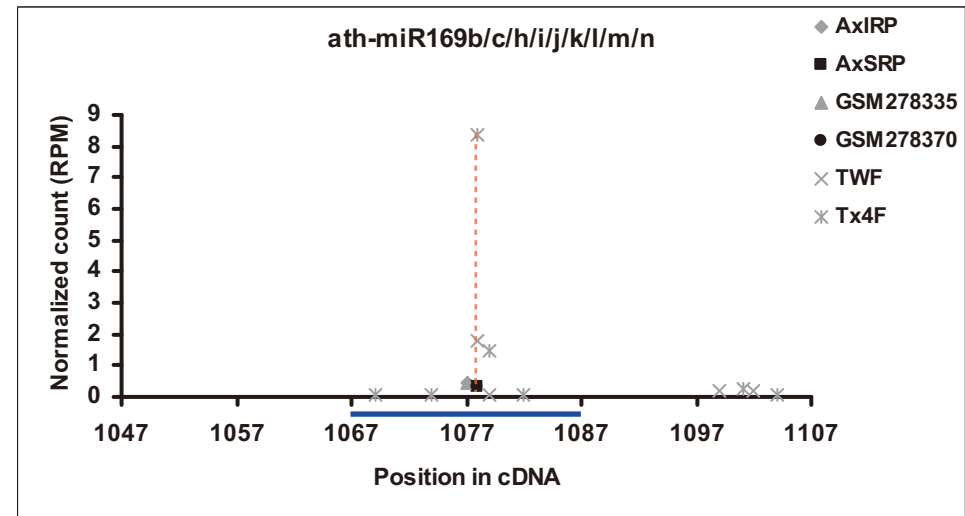

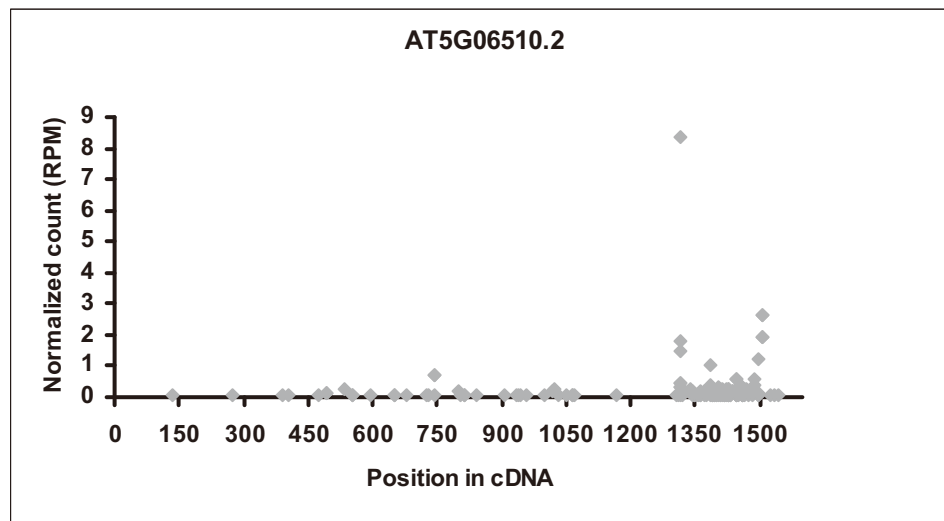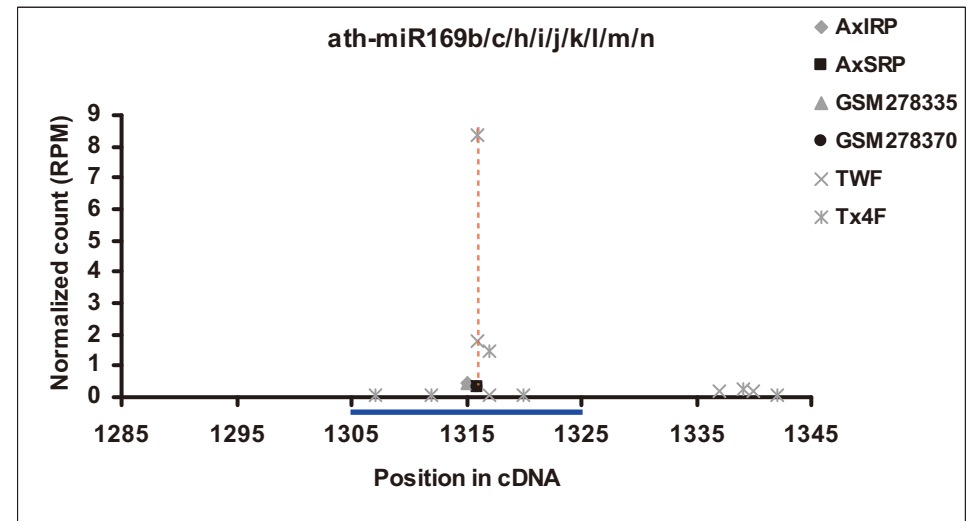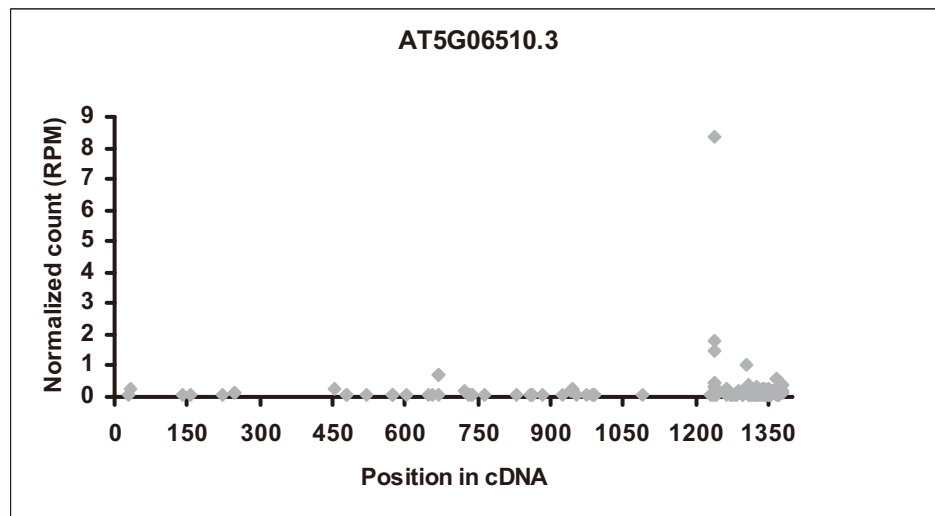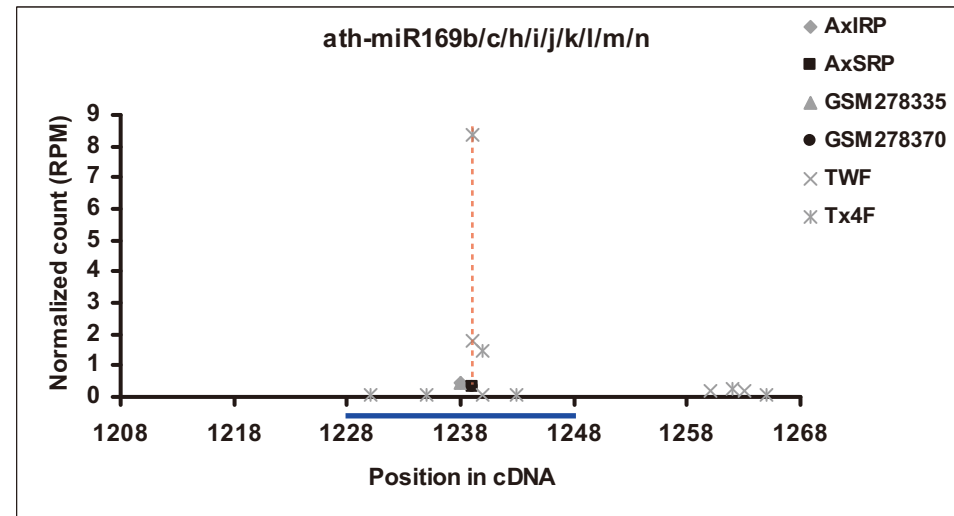

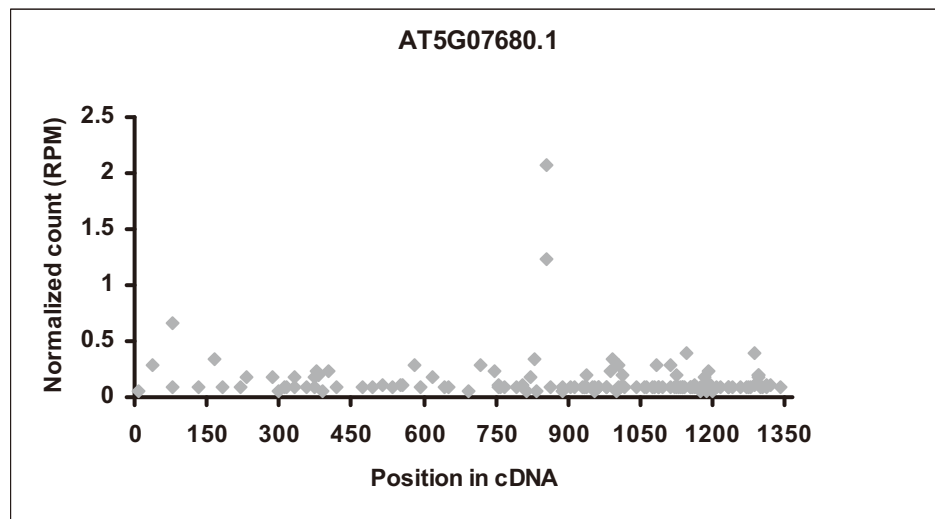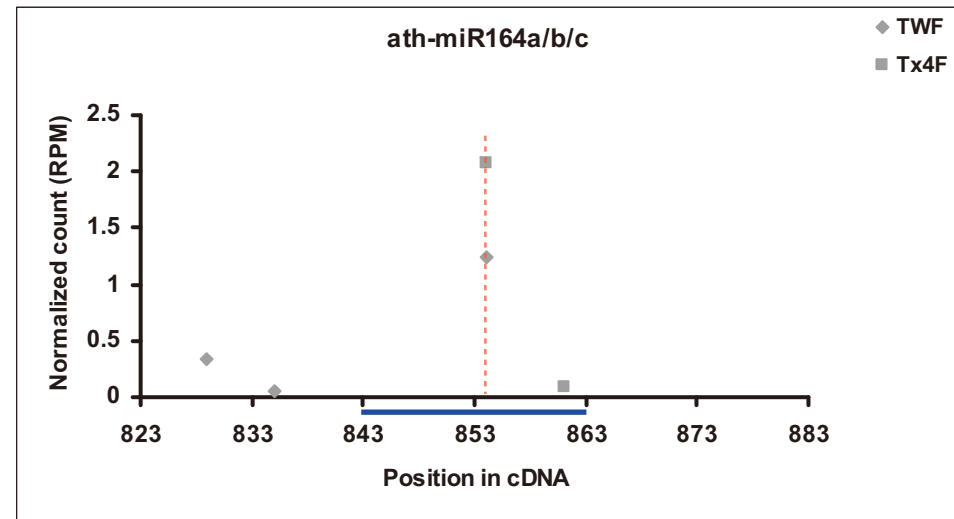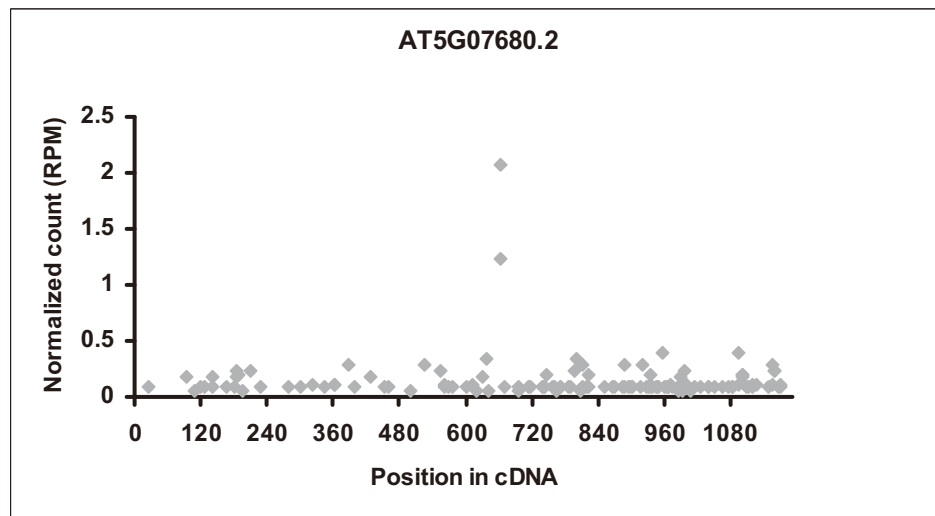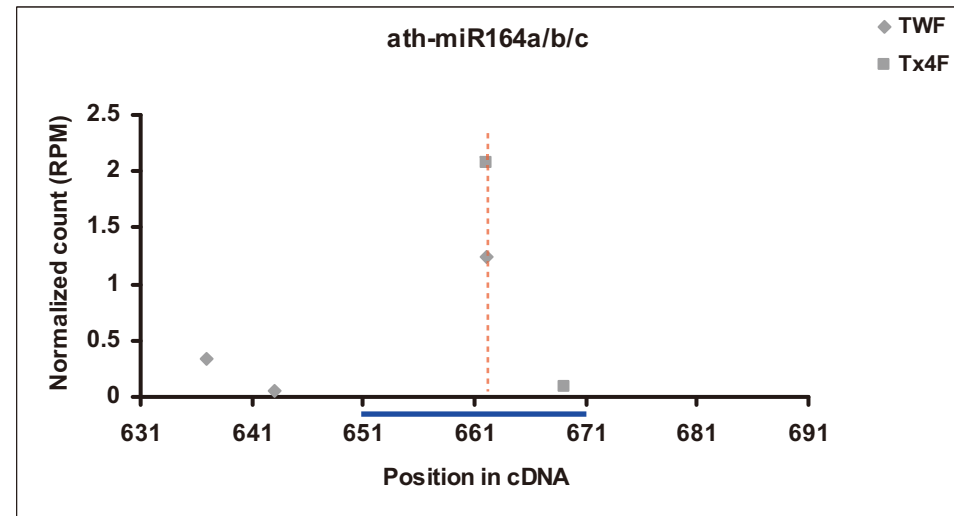

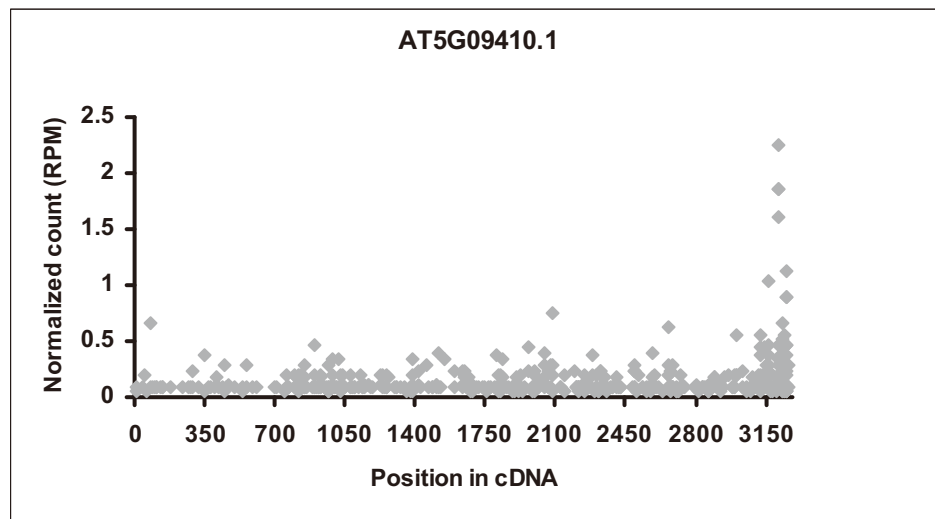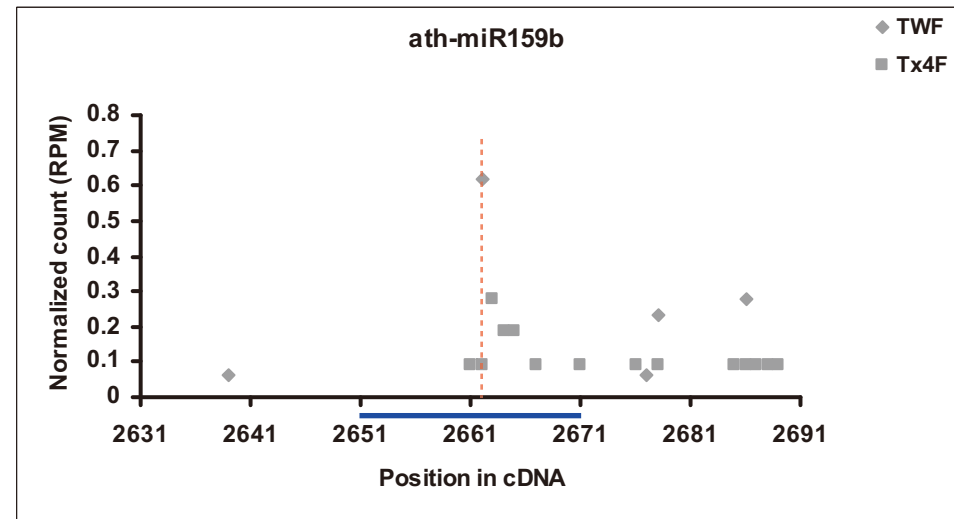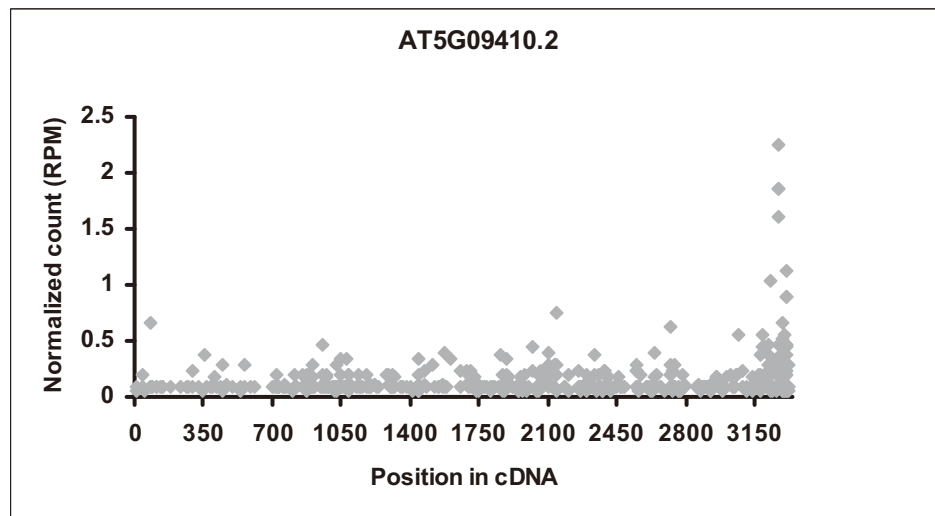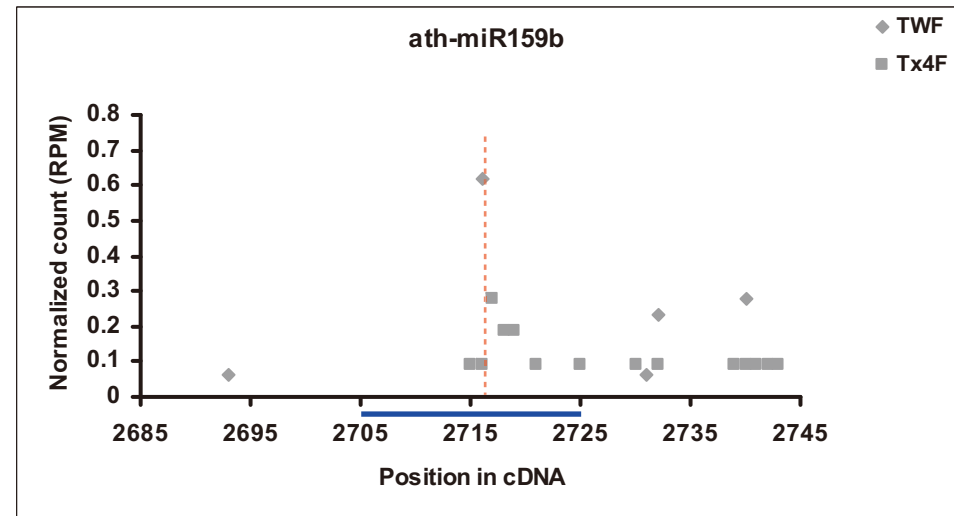

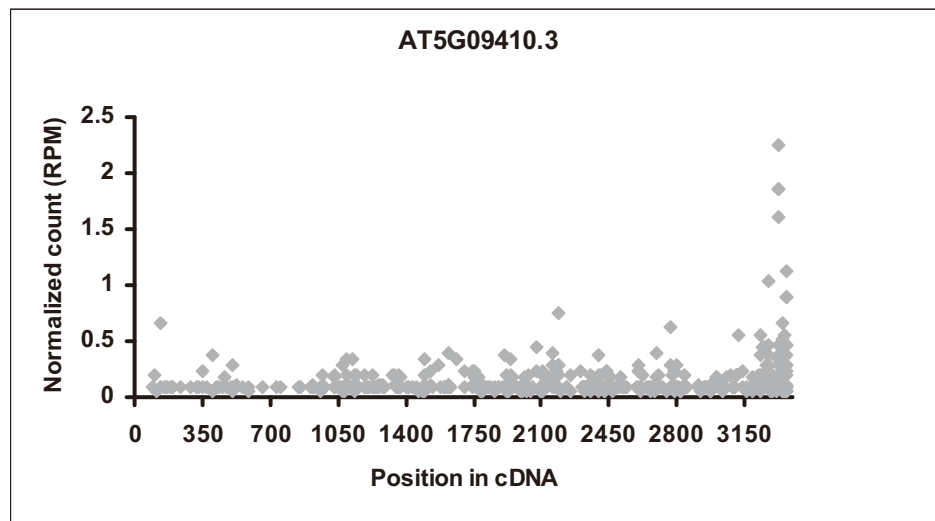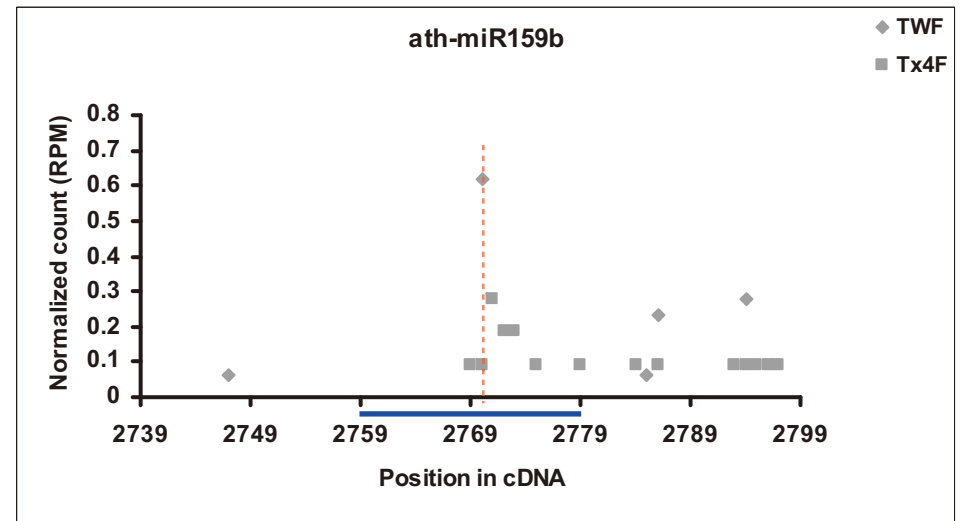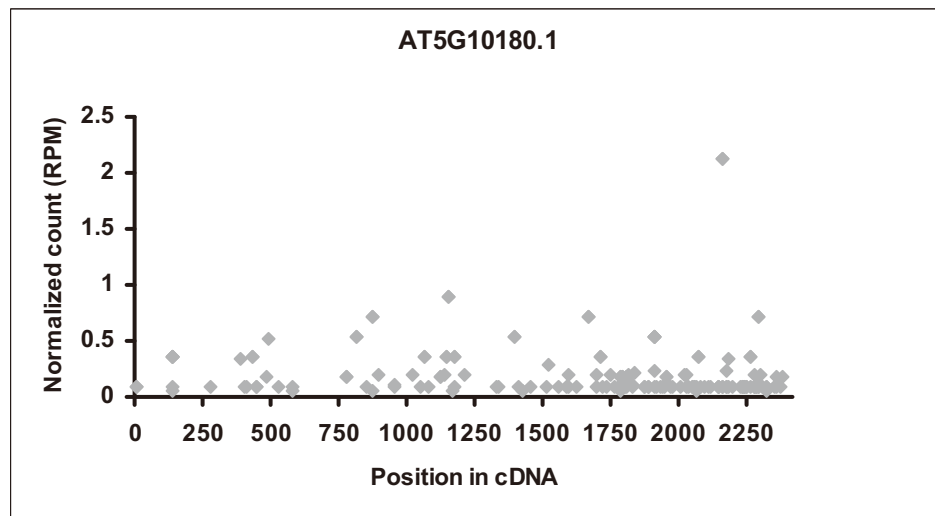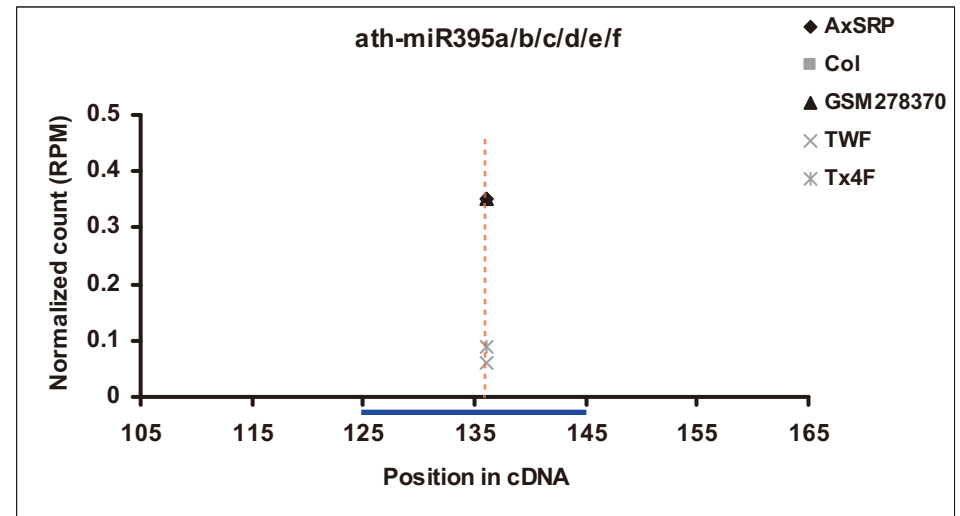

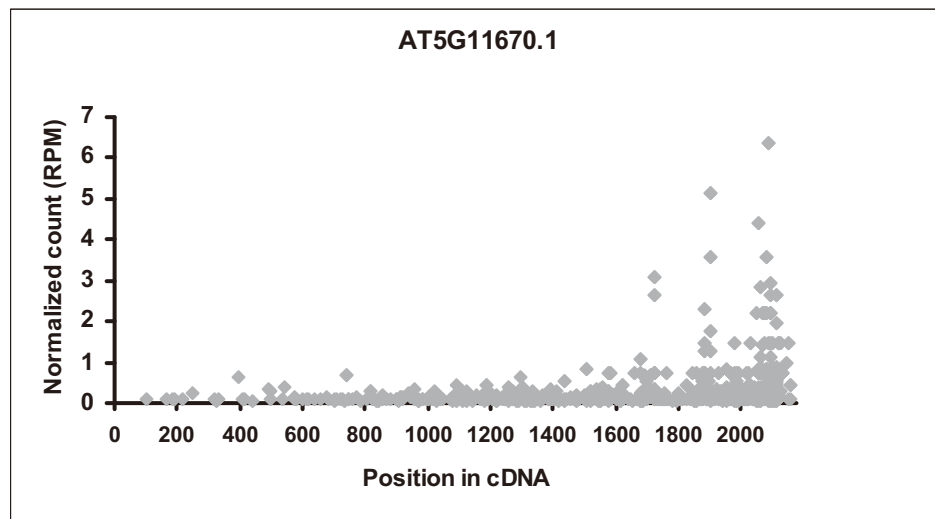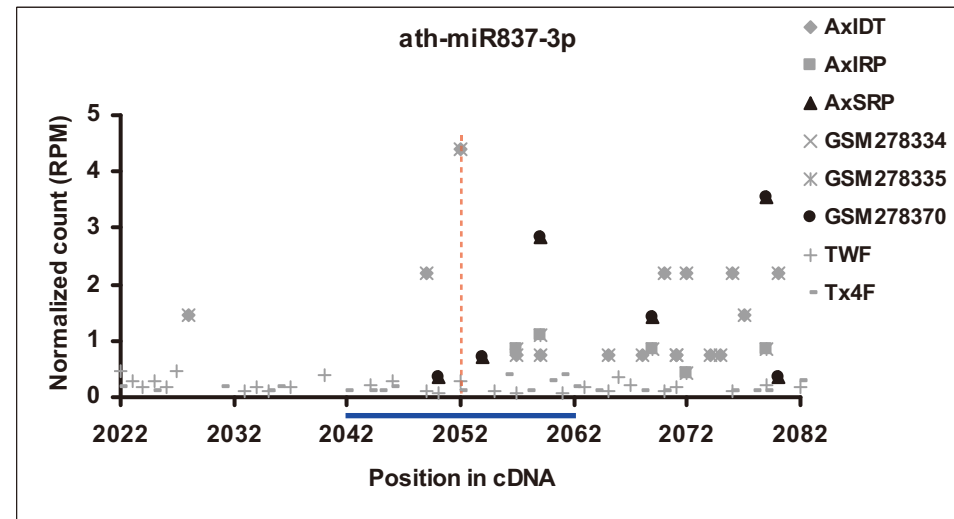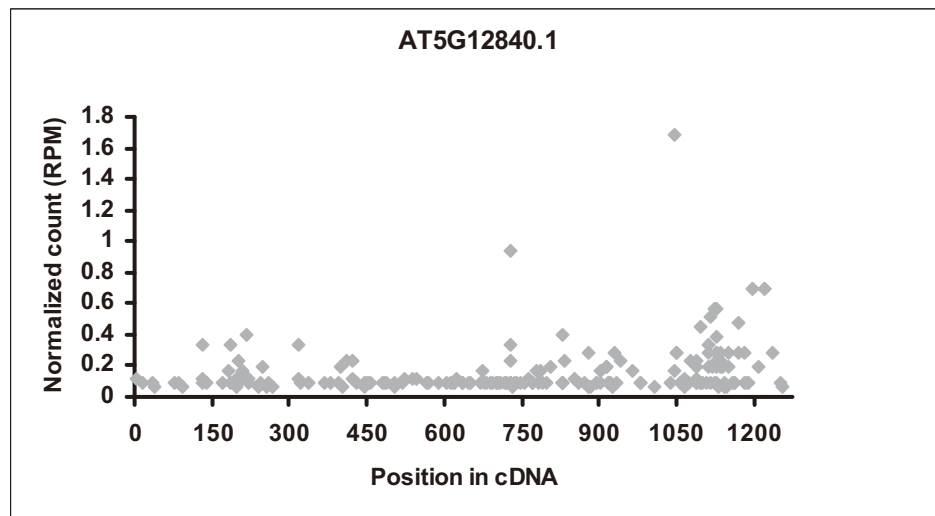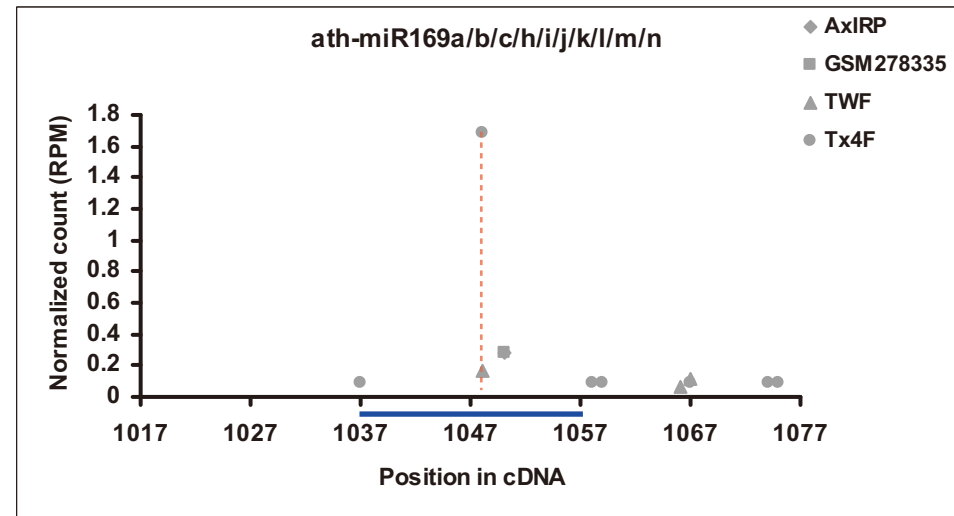

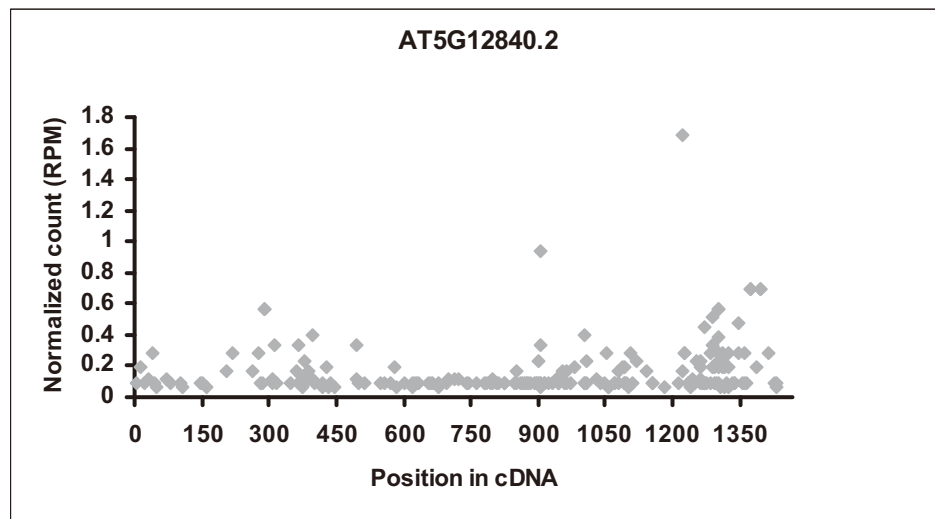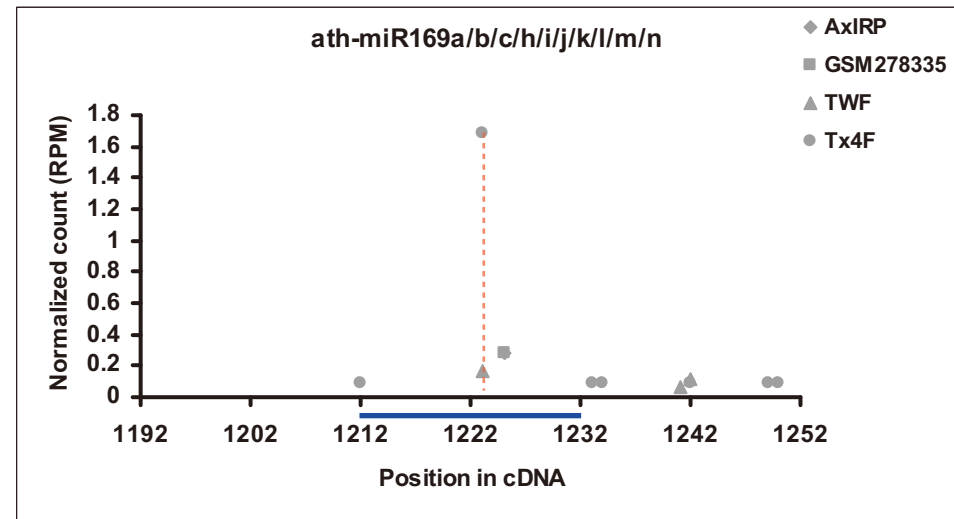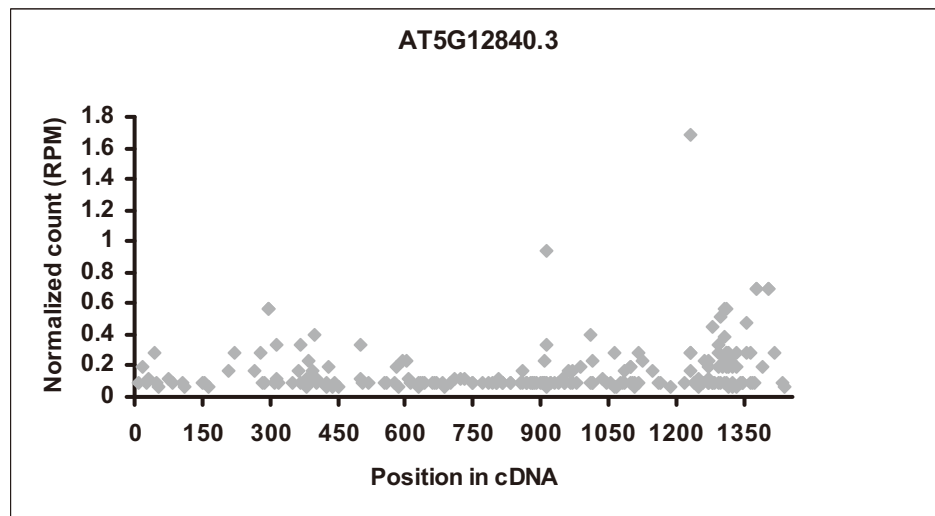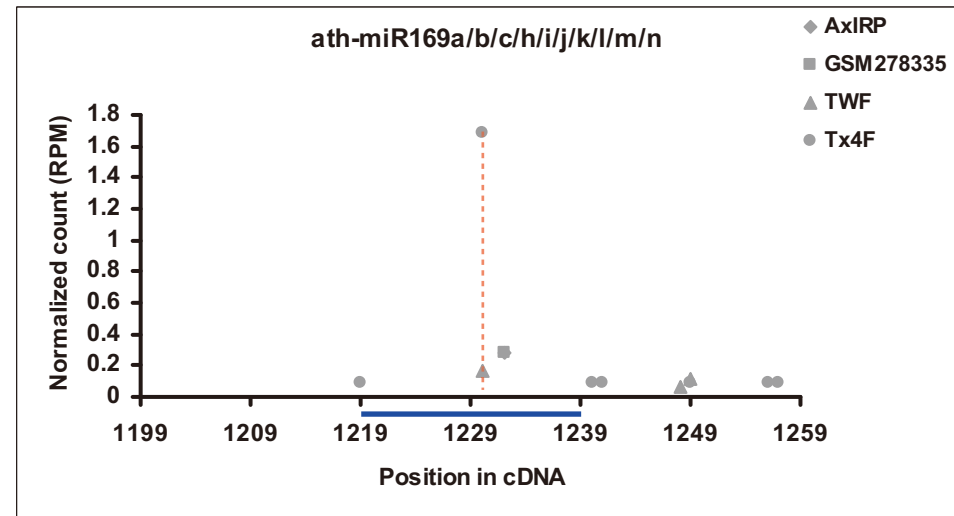

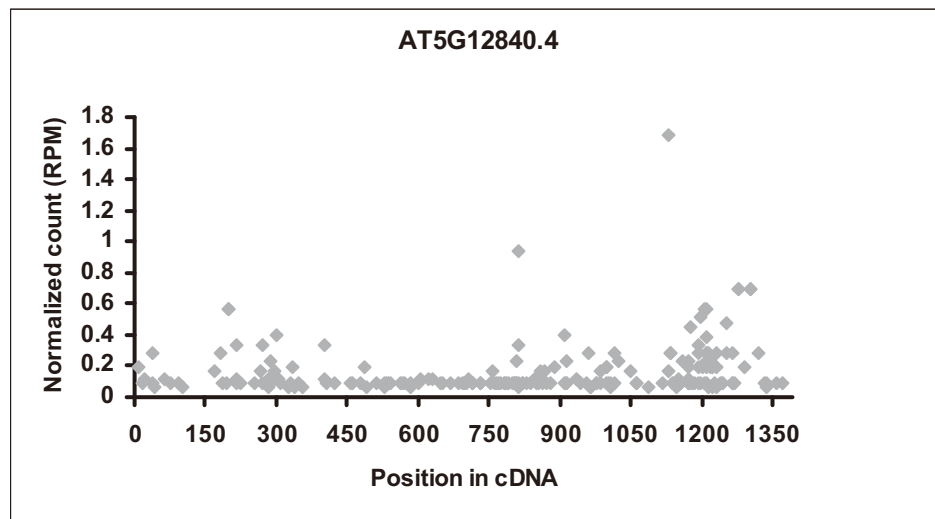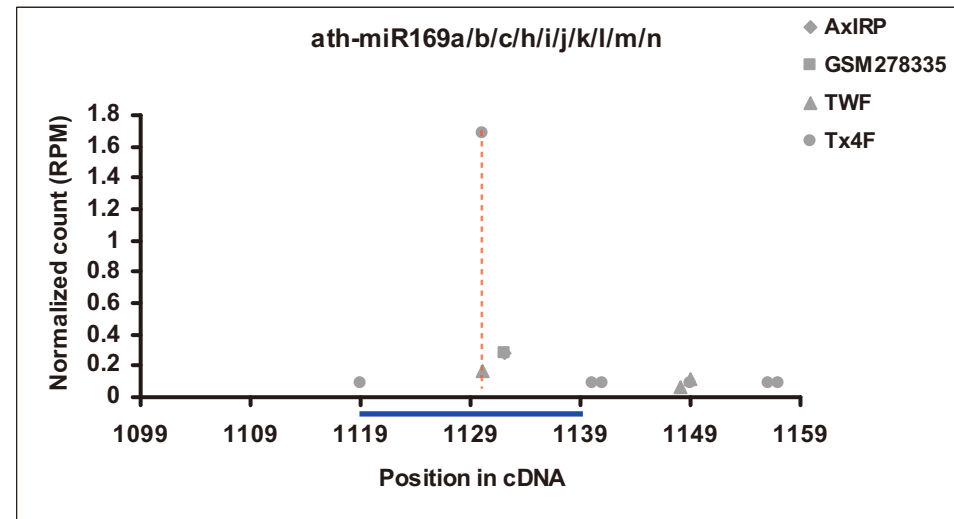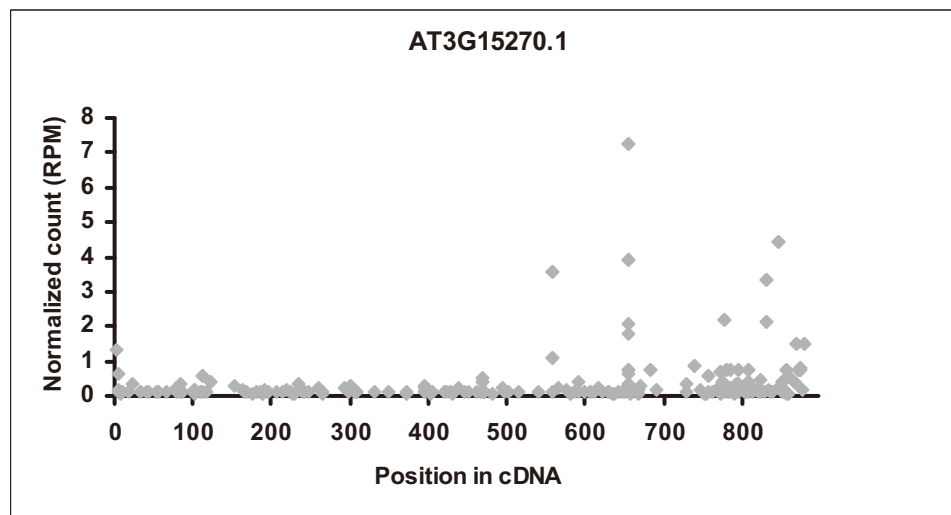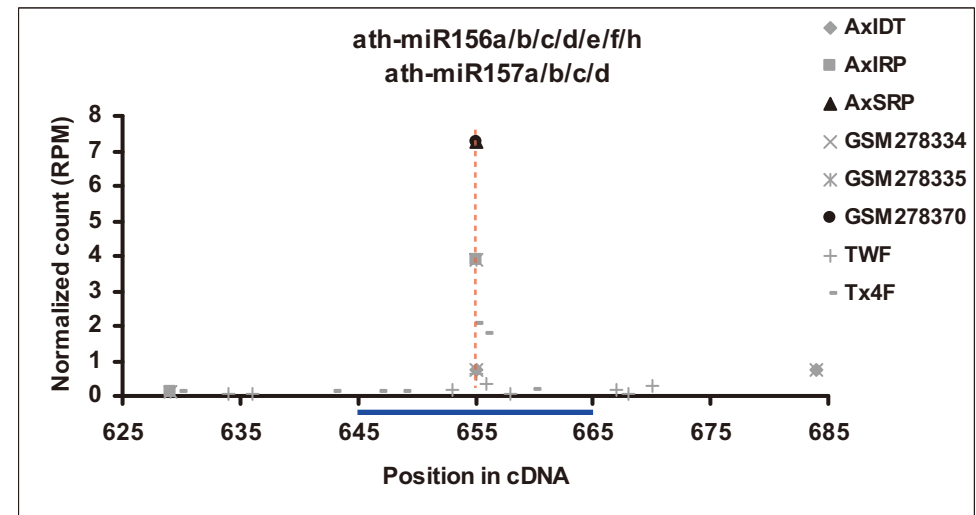

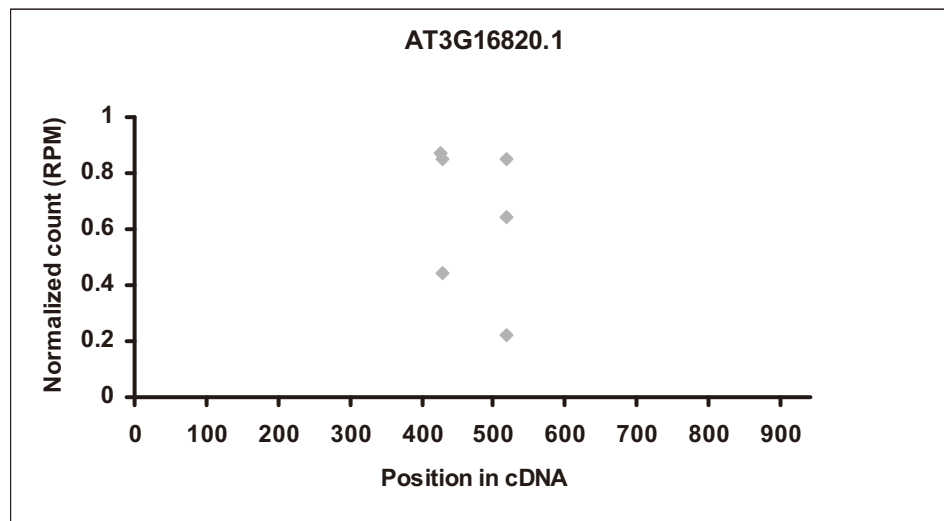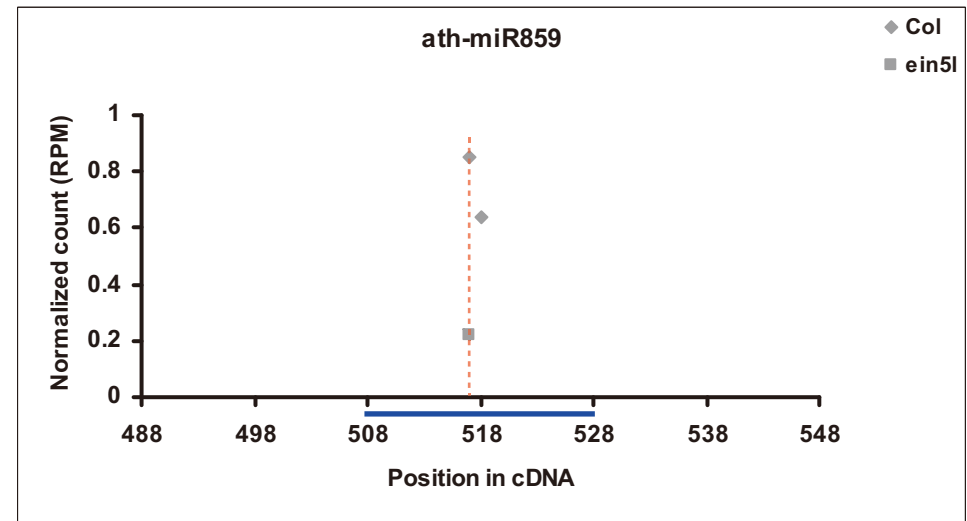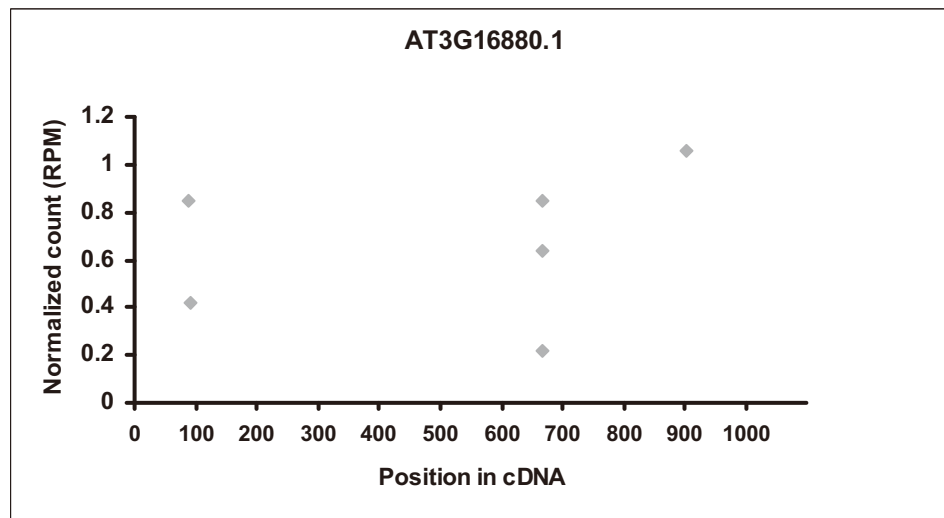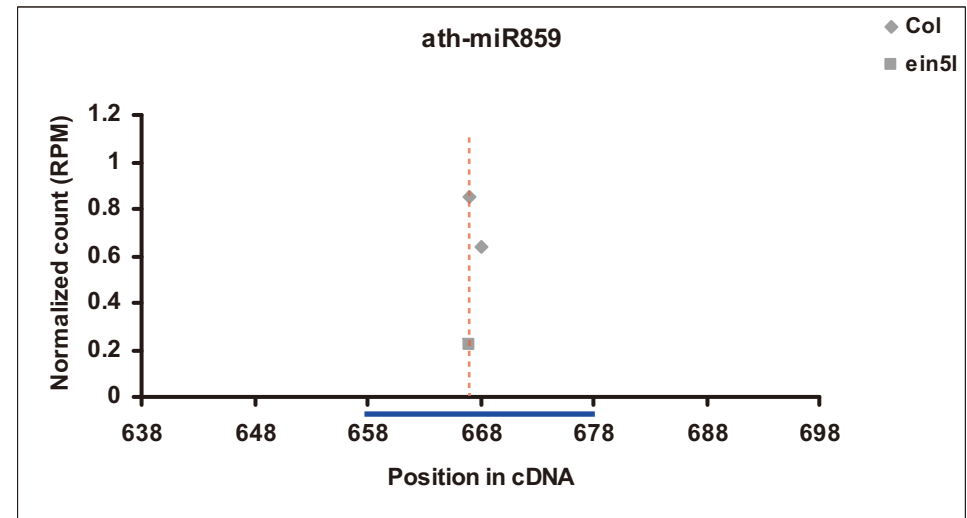

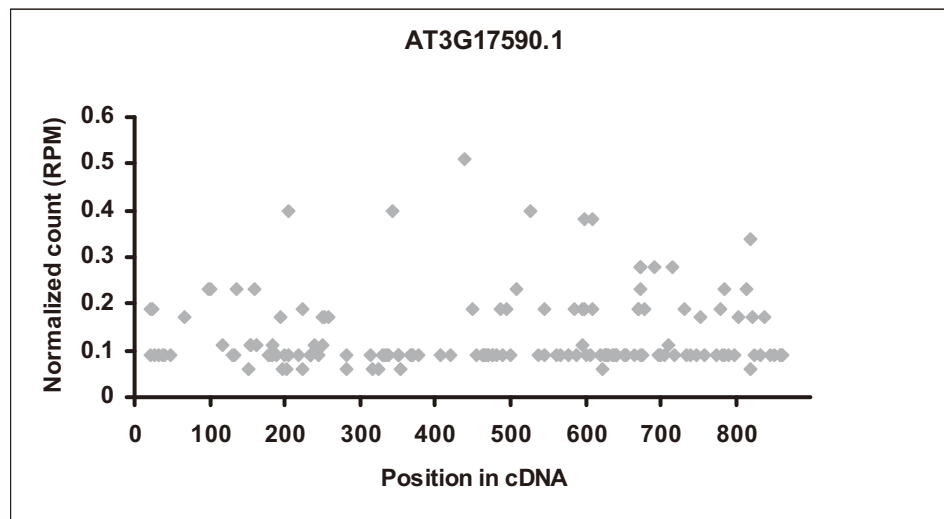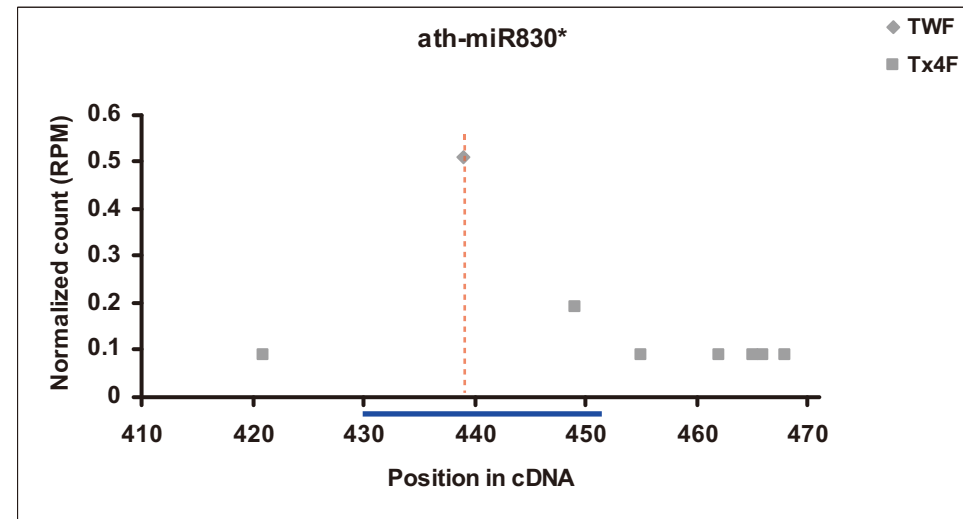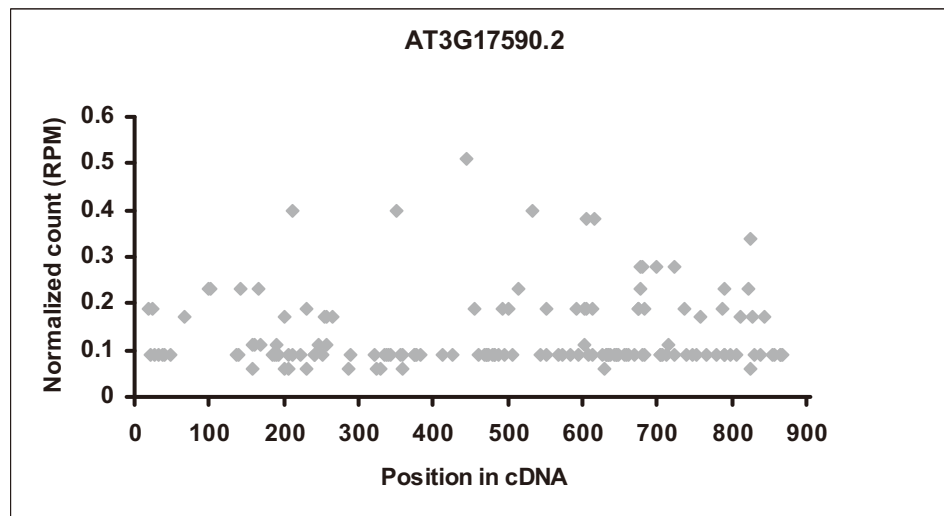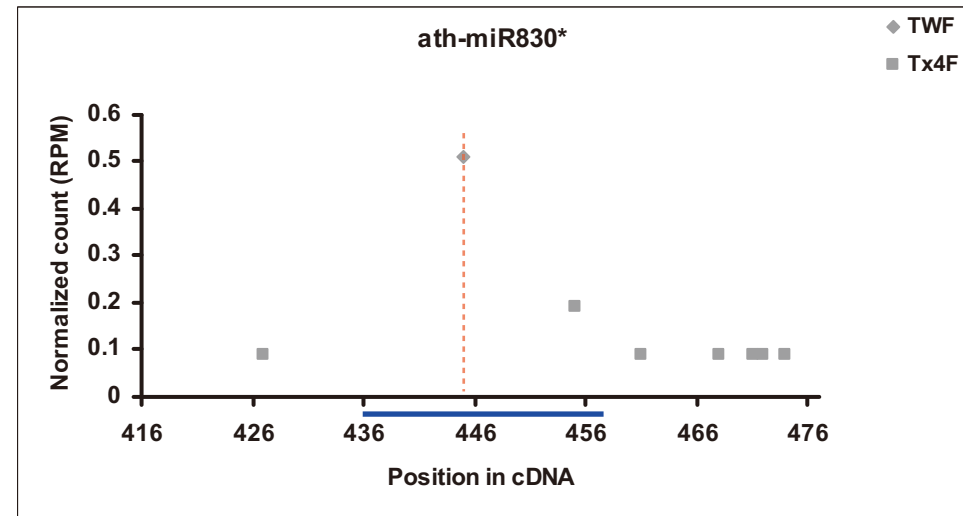

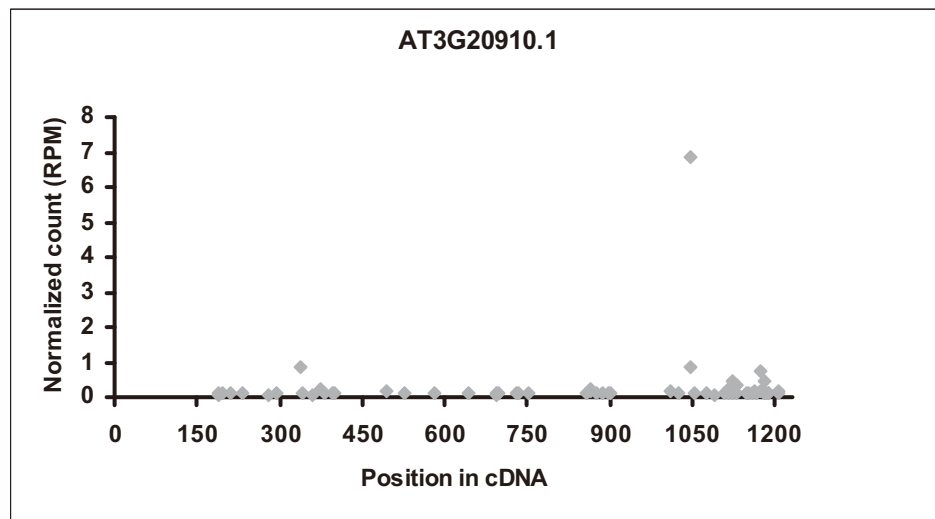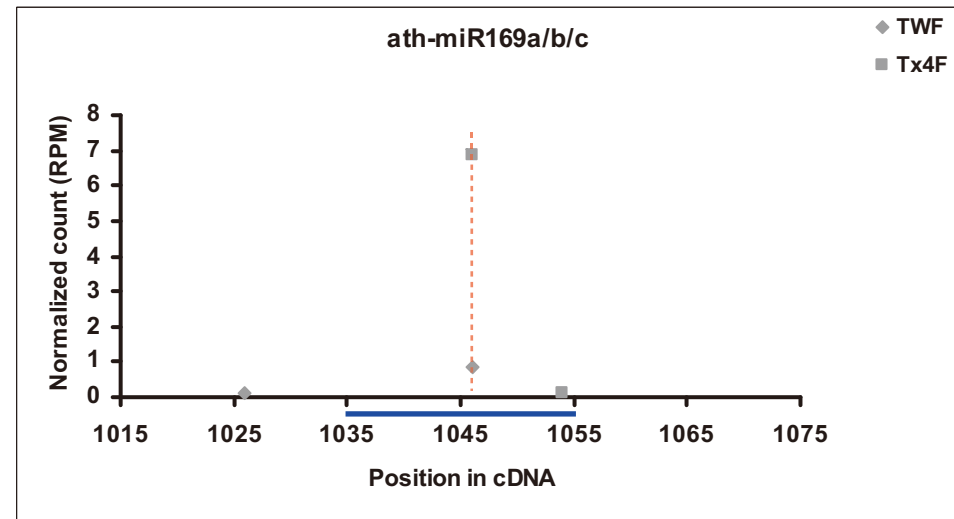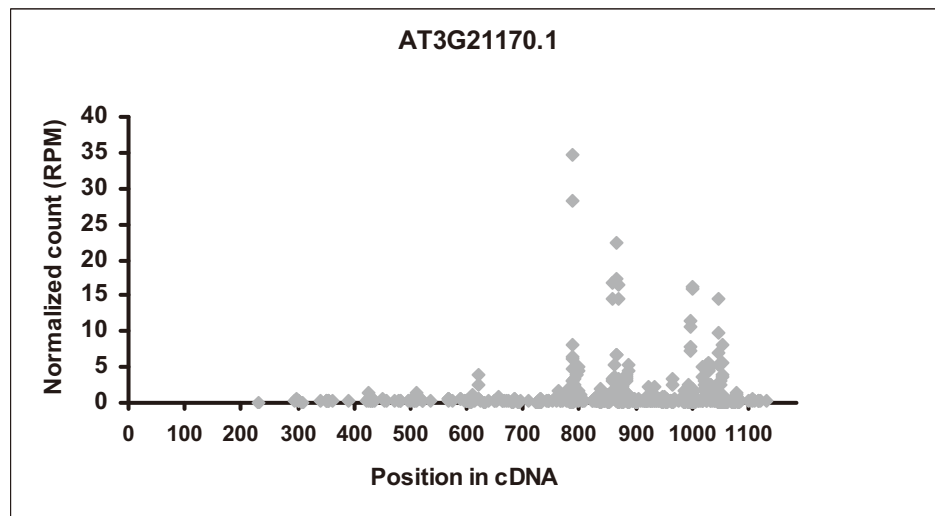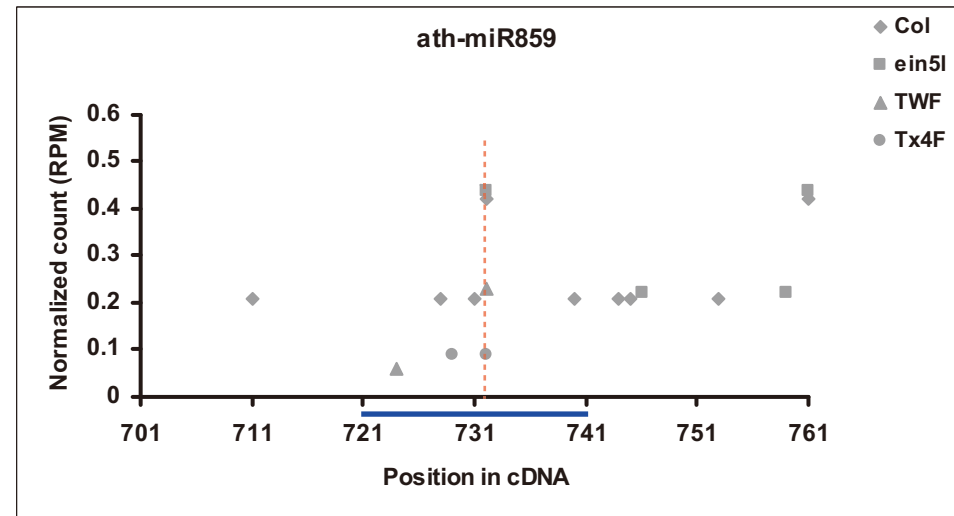

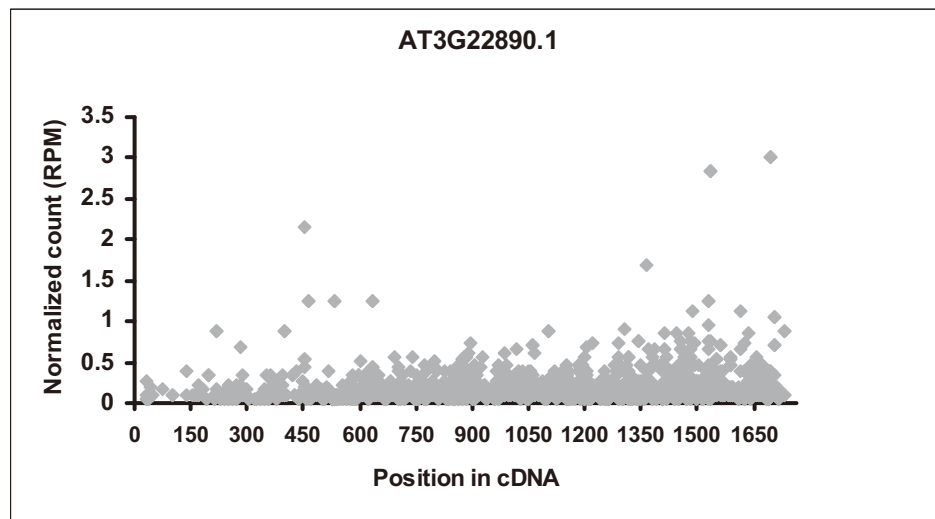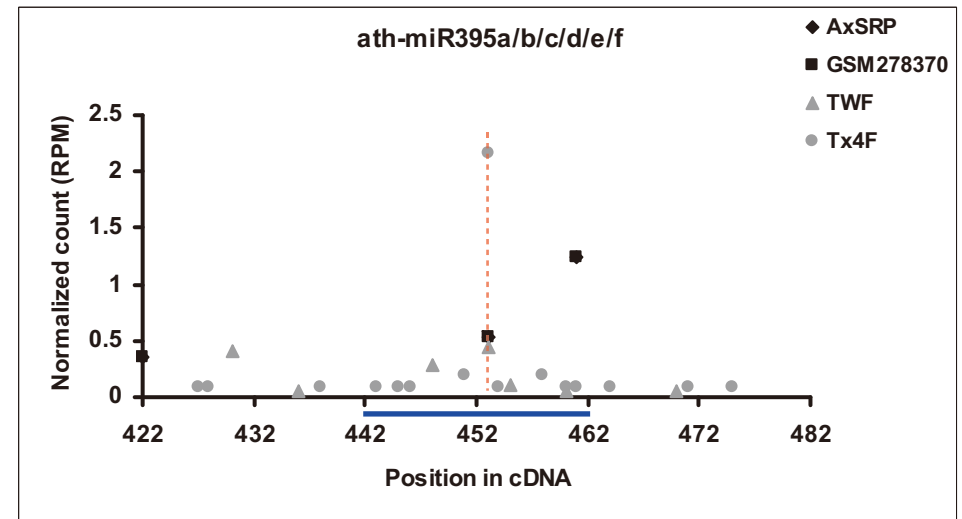

Supplement: Figure S6 — Degradome sequencing data-based identification of the targets regulated by the organ-specific microRNAs in Arabidopsis . For all the sub-figures, the left panels depict the degradome signals all along the target transcripts, and the right panels provide detailed views of the signals within the regions surrounding the target recognition sites (denoted by blue horizontal lines, and the red dotted lines indicate the most prominent cleavage sites). The transcript IDs are shown on the left panels, and the microRNAs listed on the right. For all the panels, the x axes measure the positions of the signals along the transcripts, and the y axes measure the degradome signal intensity (in RPM, reads per million). For all the right panels depicting the observed cleavage signals, the signals belonging to the libraries prepared from seedlings (AxSRP and GSM278370) were denoted by black symbols, and those belonging to the libraries prepared from inflorescences (AxIDT, AxIRP, Col, ein5l, GSM278333, GSM278334, GSM278335, TWF, and Tx4F) were denoted by gray symbols. (PDF) [file pone.0050870.s006.pdf]

(A)

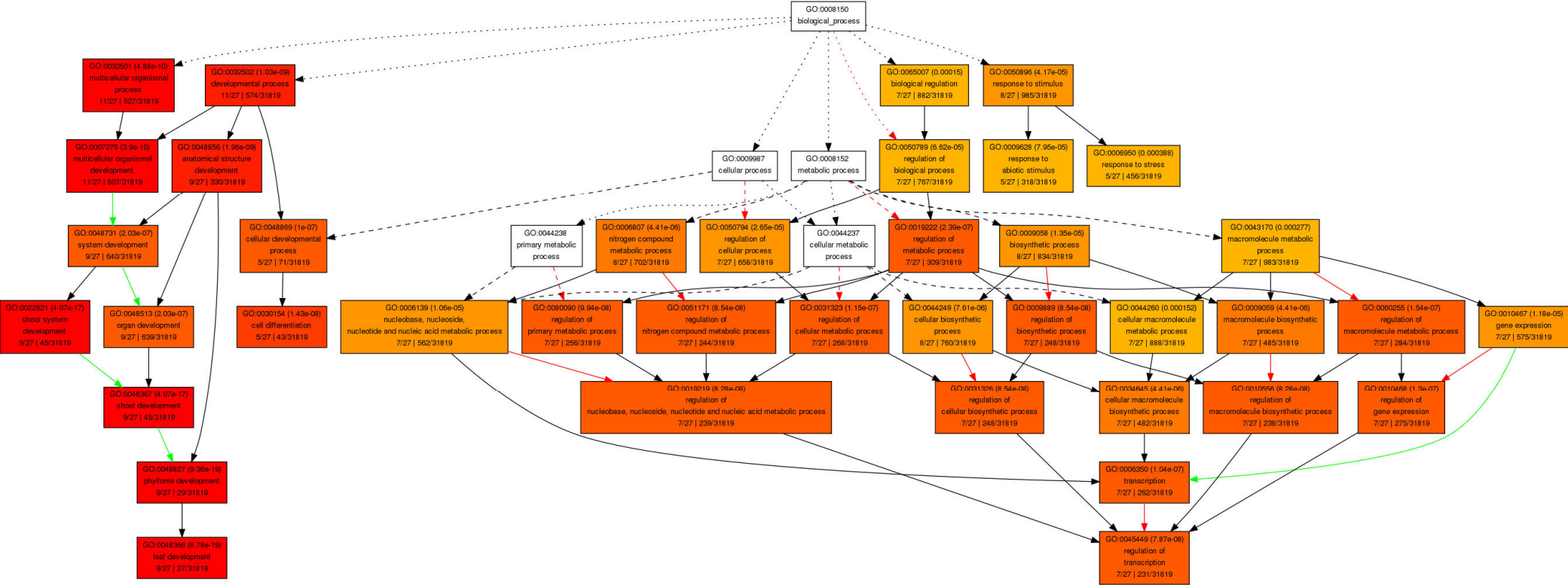

**(B)**

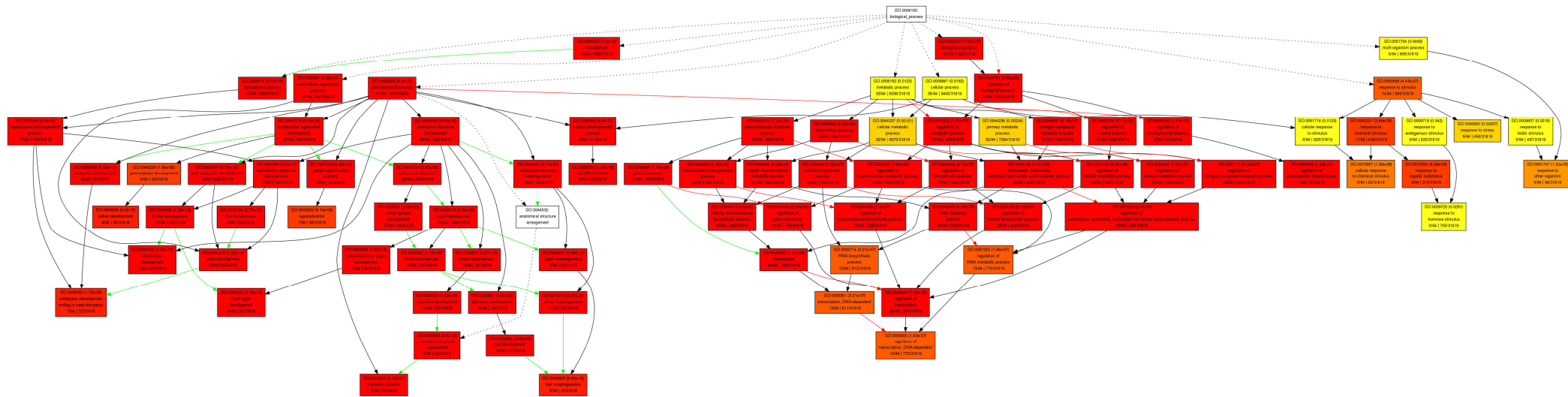

(C)

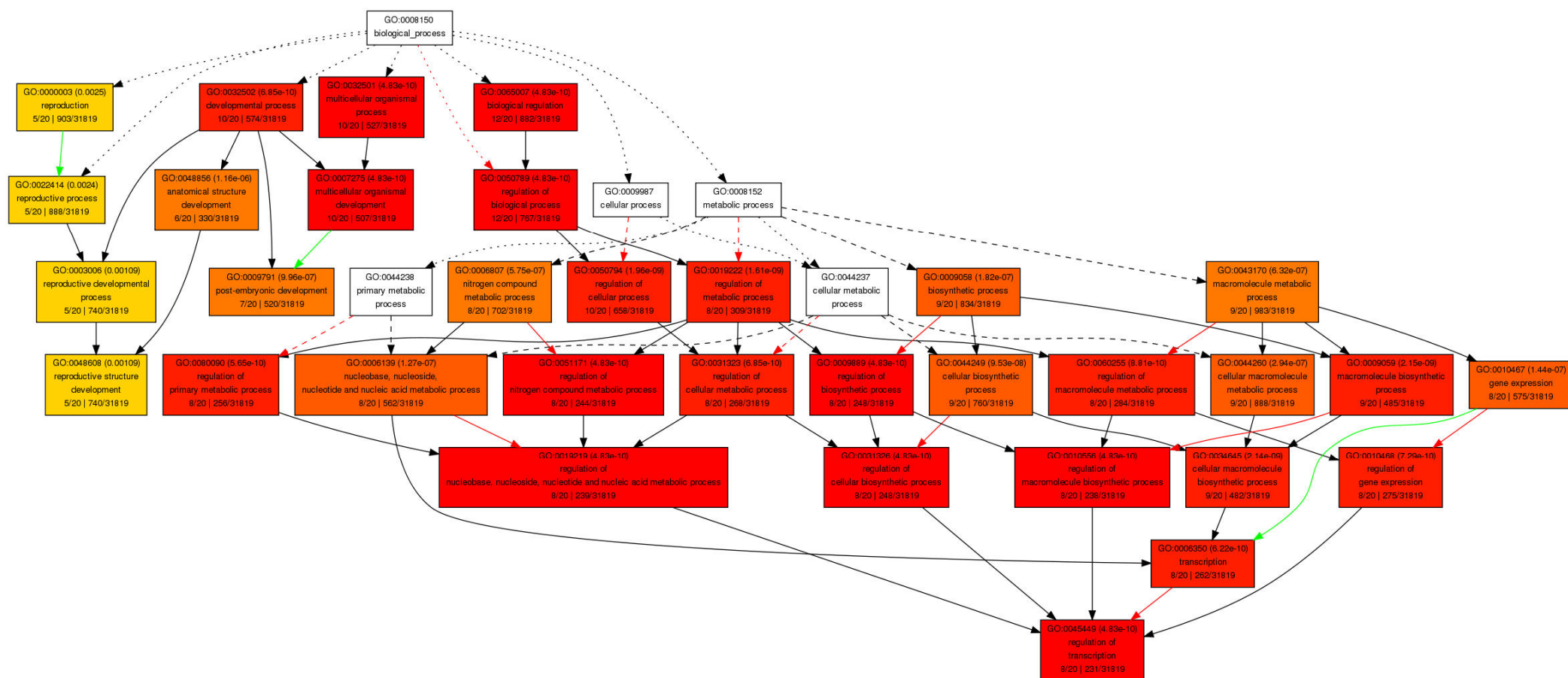

(D)

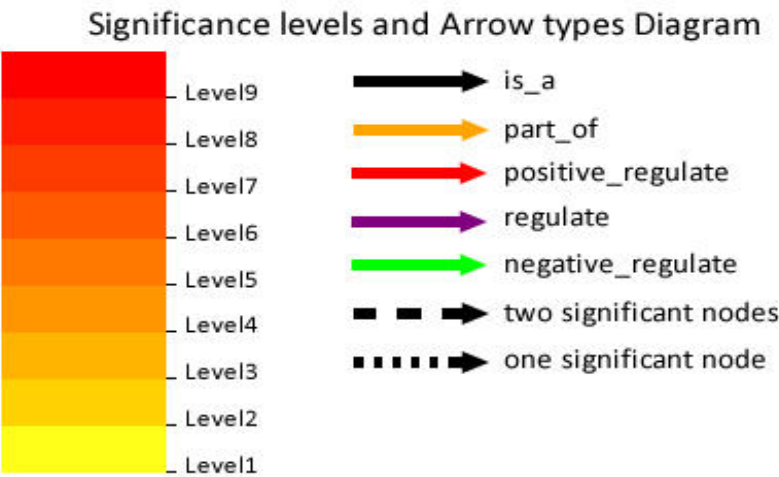

Supplement: Figure S7 — GO (Gene Ontology) term enrichment analysis of the validated targets of the organ-specific microRNAs in ARGONAUTE 1 (AGO1) of Arabidopsis . Based on the small RNA high-throughput sequencing data (GSM707682, GSM707683, GSM707684, and GSM707685), the targets of the organ-specific microRNAs only identified from the AGO1-related library group were included for this analysis. (A) Analysis of the targets of the leaf-specific microRNAs within the “Biological Process” category. (B) Analysis of the targets of the flower-specific microRNAs within the “Biological Process” category. (C) Analysis of the targets of the seedling-specific microRNAs within the “Biological Process” category. This analysis was performed by using agriGO [29], selecting the “Arabidopsis genome locus (TAIR)” as a control set. For (A) to (C), the figure keys are shown in (D). (PDF) [file pone.0050870.s007.pdf]
