# Supplementary material for: Expression-Based Functional Investigation of the Organ-Specific MicroRNAs in Arabidopsis
Source: PLoS One. 2012 Nov 30;7(11):e50870. doi: 10.1371/journal.pone.0050870 (PMC3511311; doi:10.1371/journal.pone.0050870)
Supplement: Table S1 — Expression of the 266 miRBase-registered microRNA(*)s in different organs in Arabidopsis . All the high-throughput sequencing data sets were retrieved from GEO (Gene Expression Omnibus; http://www.ncbi.nlm.nih.gov/geo/) [68]: WT_Flower (GSM707678), WT_Leaf (GSM707679), WT_Root (GSM707680), WT_Seedling (GSM707681), AGO1_Flower (GSM707682), AGO1_Leaf (GSM707683), AGO1_Root (GSM707684), AGO1_Seedling (GSM707685), AGO4_Flower (GSM707686), AGO4_Leaf (GSM707687), AGO4_Root (GSM707688), and AGO4_Seedling (GSM707689). The expression levels were shown by normalized read counts (in RPM; reads per million). (PDF) [file pone.0050870.s008.pdf]

		0	0<-<1	1<=-<10	10<=-<100	100<=-<1000	000<=-<1000	0000<=-10000	100000<=				
**miRNA**	**Sequence**	**WT_Flower**	**WT_Leaf**	**WT_Root**	**WT_Seedling**	**AGO1_Flower**	**AGO1_Leaf**	**AGO1_Root**	**AGO1_Seedling**	**AGO4_Flower**	**AGO4_Leaf**	**AGO4_Root**	**AGO4_Seedling**
ath-miR156a	UGACAGAAGAGAGUGAGCAC	108.36	238.06	51.23	1495.53	199.78	341.54	46.38	5994.85	8.75	1.03	0.8	39.17
ath-miR156b	UGACAGAAGAGAGUGAGCAC	108.36	238.06	51.23	1495.53	199.78	341.54	46.38	5994.85	8.75	1.03	0.8	39.17
ath-miR156c	UGACAGAAGAGAGUGAGCAC	108.36	238.06	51.23	1495.53	199.78	341.54	46.38	5994.85	8.75	1.03	0.8	39.17
ath-miR156d	UGACAGAAGAGAGUGAGCAC	108.36	238.06	51.23	1495.53	199.78	341.54	46.38	5994.85	8.75	1.03	0.8	39.17
ath-miR156e	UGACAGAAGAGAGUGAGCAC	108.36	238.06	51.23	1495.53	199.78	341.54	46.38	5994.85	8.75	1.03	0.8	39.17
ath-miR156f	UGACAGAAGAGAGUGAGCAC	108.36	238.06	51.23	1495.53	199.78	341.54	46.38	5994.85	8.75	1.03	0.8	39.17
ath-miR156g	CGACAGAAGAGAGUGAGCAC	0	0	0	1.29	0.25	0.36	0	7.23	0	0	0	0
ath-miR156h	UGACAGAAGAAAGAGAGCAC	13.29	0.2	0.29	0.18	67.59	0.72	0	0.83	2.67	0	0	0
ath-miR157a	UUGACAGAAGAUAGAGAGCAC	89.02	715.58	2.59	1656.07	167.23	1637.4	3.14	7452.19	4.38	3.34	0	29.26
ath-miR157b	UUGACAGAAGAUAGAGAGCAC	89.02	715.58	2.59	1656.07	167.23	1637.4	3.14	7452.19	4.38	3.34	0	29.26
ath-miR157c	UUGACAGAAGAUAGAGAGCAC	89.02	715.58	2.59	1656.07	167.23	1637.4	3.14	7452.19	4.38	3.34	0	29.26
ath-miR157d	UGACAGAAGAUAGAGAGCAC	5.64	22.41	0	46.98	9.26	41.62	0	211.59	0.73	0	0	0.24
ath-miR158a	UCCCAAAUGUAGACAAAGCA	13378.09	25016.54	11683.21	12735.1	41217.85	143709.88	63787.33	235267.58	1080.57	599.35	829.03	1219.12
ath-miR158b	CCCCAAAUGUAGACAAAGCA	16.31	11.4	25.04	21.27	130.68	303.87	305.77	1113.51	2.19	1.29	3.47	3.78
ath-miR159a	UUUGGAUUGAAGGGAGCUCUA	4532.41	4894.6	2550.03	2736.01	49830.64	74556.8	18349.26	25792.98	328.98	97.75	127.44	96.03
ath-miR159b	UUUGGAUUGAAGGGAGCUCUU	2653.42	5624.98	1256.02	3434.76	27199.01	110106.13	13623.99	24839.19	243.4	102.64	116.49	120.8
ath-miR159c	UUUGGAUUGAAGGGAGCUCCU	1.41	3.2	0.86	2.77	50.32	171.13	31.83	20.04	0.49	0.26	0.8	0.24
ath-miR160a	UGCCUGGCUCCCUGUAUGCCA	120.24	218.45	263.06	231.56	2413.62	2840.7	1765.83	4143.89	29.66	7.46	15.23	15.81
ath-miR160b	UGCCUGGCUCCCUGUAUGCCA	120.24	218.45	263.06	231.56	2413.62	2840.7	1765.83	4143.89	29.66	7.46	15.23	15.81
ath-miR160c	UGCCUGGCUCCCUGUAUGCCA	120.24	218.45	263.06	231.56	2413.62	2840.7	1765.83	4143.89	29.66	7.46	15.23	15.81
ath-miR161.1	UGAAAGUGACUACAUCGGGGU	694.68	1045.26	652.76	803.99	4336.56	8996.04	4568.45	6624.24	51.31	23.67	46.49	31.38
ath-miR161.2	UCAAUGCAUUGAAAGUGACUA	904.55	2140.73	1615.79	955.65	4549.61	2939	2979.47	12175.67	99.45	11.06	13.89	50.96
ath-miR162a	UCGAUAAACCUCUGCAUCCAG	373.62	389.9	219.89	136.5	1712.14	3161.44	996.7	1405.06	25.04	4.37	8.55	5.66
ath-miR162b	UCGAUAAACCUCUGCAUCCAG	373.62	389.9	219.89	136.5	1712.14	3161.44	996.7	1405.06	25.04	4.37	8.55	5.66
ath-miR163	UUGAAGAGGACUUGGAACUUCGAU	3690.3	8029.77	2072.83	2218.51	49547.99	50470.38	6225.81	35596.78	173.37	101.35	51.56	57.57
ath-miR164a	UGGAGAAGCAGGGCACGUGCA	151.06	107.83	85.19	196.6	984.13	798.61	400.49	968.05	14.83	1.03	6.14	3.78
ath-miR164b	UGGAGAAGCAGGGCACGUGCA	151.06	107.83	85.19	196.6	984.13	798.61	400.49	968.05	14.83	1.03	6.14	3.78
ath-miR164c	UGGAGAAGCAGGGCACGUGCG	2.82	4	1.15	5.36	8.01	20.45	4.72	27.07	0.24	0	0	0
ath-miR165a	UCGGACCAGGCUUCAUCCCCC	750.87	412.3	474.6	332.55	26140.03	17565.86	6791.36	19209.65	267.95	28.81	158.97	82.34
ath-miR165b	UCGGACCAGGCUUCAUCCCCC	750.87	412.3	474.6	332.55	26140.03	17565.86	6791.36	19209.65	267.95	28.81	158.97	82.34
ath-miR166a	UCGGACCAGGCUUCAUUCCCC	3261.89	2154.13	1228.68	1448.18	76756.02	56156.81	14661.96	42640.47	966.77	87.72	299.5	213.29
ath-miR166b	UCGGACCAGGCUUCAUUCCCC	3261.89	2154.13	1228.68	1448.18	76756.02	56156.81	14661.96	42640.47	966.77	87.72	299.5	213.29
ath-miR166c	UCGGACCAGGCUUCAUUCCCC	3261.89	2154.13	1228.68	1448.18	76756.02	56156.81	14661.96	42640.47	966.77	87.72	299.5	213.29
ath-miR166d	UCGGACCAGGCUUCAUUCCCC	3261.89	2154.13	1228.68	1448.18	76756.02	56156.81	14661.96	42640.47	966.77	87.72	299.5	213.29
ath-miR166e	UCGGACCAGGCUUCAUUCCCC	3261.89	2154.13	1228.68	1448.18	76756.02	56156.81	14661.96	42640.47	966.77	87.72	299.5	213.29
ath-miR166f	UCGGACCAGGCUUCAUUCCCC	3261.89	2154.13	1228.68	1448.18	76756.02	56156.81	14661.96	42640.47	966.77	87.72	299.5	213.29
ath-miR166g	UCGGACCAGGCUUCAUUCCCC	3261.89	2154.13	1228.68	1448.18	76756.02	56156.81	14661.96	42640.47	966.77	87.72	299.5	213.29
ath-miR167a	UGAAGCUGCCAGCAUGAUCUA	1991.78	3575.48	505.4	2368.5	6554.16	6224.22	921.24	13857.2	156.83	5.14	7.21	56.39
ath-miR167b	UGAAGCUGCCAGCAUGAUCUA	1991.78	3575.48	505.4	2368.5	6554.16	6224.22	921.24	13857.2	156.83	5.14	7.21	56.39
ath-miR167c	UAAGCUGCCAGCAUGAUCUUG	1.21	2.2	123.76	17.02	3.25	8.97	300.27	395.28	0.24	1.54	3.21	0.47
ath-miR167d	UGAAGCUGCCAGCAUGAUCUGG	44.31	290.67	3.17	223.98	137.44	1171.73	5.9	1137.27	1.95	1.03	0.53	3.54
ath-miR168a	UCGCUUGGUGCAGGUCGGGAA	266.47	435.31	174.99	166.64	1012.92	1608.34	349.79	1250.3	12.4	2.32	8.55	4.48
ath-miR168b	UCGCUUGGUGCAGGUCGGGAA	266.47	435.31	174.99	166.64	1012.92	1608.34	349.79	1250.3	12.4	2.32	8.55	4.48
ath-miR169a	CAGCCAAGGAUGACUUGCCGA	54.18	254.06	225.36	50.86	16.27	85.74	92.36	387.84	0.73	0	0	0.47
ath-miR169b	CAGCCAAGGAUGACUUGCCGG	4.23	3.8	90.37	22.01	13.02	11.48	108.47	200.63	0.24	0	0.53	0.24
ath-miR169c	CAGCCAAGGAUGACUUGCCGG	4.23	3.8	90.37	22.01	13.02	11.48	108.47	200.63	0.24	0	0.53	0.24
ath-miR169d	UGAGCCAAGGAUGACUUGCCG	13.49	9.2	10.94	3.7	31.79	31.21	29.48	28.31	0.24	0	0	0
ath-miR169e	UGAGCCAAGGAUGACUUGCCG	13.49	9.2	10.94	3.7	31.79	31.21	29.48	28.31	0.24	0	0	0
ath-miR169f	UGAGCCAAGGAUGACUUGCCG	13.49	9.2	10.94	3.7	31.79	31.21	29.48	28.31	0.24	0	0	0
ath-miR169g	UGAGCCAAGGAUGACUUGCCG	13.49	9.2	10.94	3.7	31.79	31.21	29.48	28.31	0.24	0	0	0
ath-miR169g*	UCCGGCAAGUUGACCUUGGCU	0	0	0	0	0	0	0	0.21	0	0	0	0
ath-miR169h	UAGCCAAGGAUGACUUGCCUG	7.65	38.41	39.43	10.73	4.51	113.37	88.82	77.48	0.49	0.26	0.27	0.47
ath-miR169i	UAGCCAAGGAUGACUUGCCUG	7.65	38.41	39.43	10.73	4.51	113.37	88.82	77.48	0.49	0.26	0.27	0.47
ath-miR169j	UAGCCAAGGAUGACUUGCCUG	7.65	38.41	39.43	10.73	4.51	113.37	88.82	77.48	0.49	0.26	0.27	0.47
ath-miR169k	UAGCCAAGGAUGACUUGCCUG	7.65	38.41	39.43	10.73	4.51	113.37	88.82	77.48	0.49	0.26	0.27	0.47
ath-miR169l	UAGCCAAGGAUGACUUGCCUG	7.65	38.41	39.43	10.73	4.51	113.37	88.82	77.48	0.49	0.26	0.27	0.47
ath-miR169m	UAGCCAAGGAUGACUUGCCUG	7.65	38.41	39.43	10.73	4.51	113.37	88.82	77.48	0.49	0.26	0.27	0.47
ath-miR169n	UAGCCAAGGAUGACUUGCCUG	7.65	38.41	39.43	10.73	4.51	113.37	88.82	77.48	0.49	0.26	0.27	0.47
ath-miR170	UGAUUGAGCCGUGUCAAUAUC	63.04	32.21	1.15	61.96	126.68	40.9	0.79	185.34	2.92	0	0	1.18
ath-miR171a	UGAUUGAGCCGCGCCAAUAUC	405.65	257.86	0.58	352.7	2174.29	370.25	1.18	1323.03	27.72	0.26	0	5.43
ath-miR171b	UUGAGCCGUGCCAAUAUCACG	66.47	40.81	37.13	47.35	736.28	318.94	148.17	466.77	9.24	0.26	1.07	3.3
ath-miR171c	UUGAGCCGUGCCAAUAUCACG	66.47	40.81	37.13	47.35	736.28	318.94	148.17	466.77	9.24	0.26	1.07	3.3
ath-miR172a	AGAAUCUUGAUGAUGCUGCAU	1154.91	2035.1	1466.7	929.76	5566.03	2707.96	540.01	1100.08	165.1	56.85	41.68	18.4
ath-miR172b	AGAAUCUUGAUGAUGCUGCAU	1154.91	2035.1	1466.7	929.76	5566.03	2707.96	540.01	1100.08	165.1	56.85	41.68	18.4
ath-miR172b*	GCAGCACCAUUAAGAUUCAC	22.56	76.82	5.18	22.01	0.5	0	1.57	0.83	0	0.26	0	0
ath-miR172c	AGAAUCUUGAUGAUGCUGCAG	617.94	6.8	562.68	16.28	2861.5	42.33	863.46	36.99	110.39	2.06	33.4	0.47
ath-miR172d	AGAAUCUUGAUGAUGCUGCAG	617.94	6.8	562.68	16.28	2861.5	42.33	863.46	36.99	110.39	2.06	33.4	0.47
ath-miR172e	GGAAUCUUGAUGAUGCUGCAU	33.43	5.4	260.47	26.63	158.47	19.01	117.12	36.99	2.43	0.77	7.75	1.18
ath-miR173	UUCGCUUGCAGAGAGAAAUCAC	859.63	1264.11	1120.46	1310.58	3485.12	4397.39	3875.95	9068.22	55.92	12.09	21.64	23.83
ath-miR1886.1	UGAGAGAAGUGAGAUGAAAUC	0.4	1.6	0.29	1.66	4.51	6.46	1.97	9.3	0.24	0	0	0
ath-miR1886.2	UGAGAUGAAAUCUUUGAUUGG	20.34	17.8	9.21	15.35	70.35	54.53	36.16	114.88	2.92	0	0.53	0
ath-miR1886.3	AAUUAAAGAUUUCAUCUUACU	3.22	4.8	1.44	4.99	0	0	0	1.03	0	0	0	0.24
ath-miR1887	UACUAAGUAGAGUCUAAGAGA	0.4	0	0	0	0.5	0	0	0	0	0	0	0
ath-miR1888	UAAGUUAAGAUUUGUGAAGAA	22.36	42.81	0.29	21.45	133.19	260.1	0.79	150.01	1.22	0.51	0	0.24
ath-miR2111a	UAAUCUGCAUCCUGAGGUUUA	1.01	0.6	2.01	9.62	4.01	0.72	0	3.31	0.24	0	0	0
ath-miR2111a*	GUCCUCGGGAUGCGGAUUACC	0	0	0.86	11.84	4.76	0	0	1.65	0	0	0	0.47
ath-miR2111b	UAAUCUGCAUCCUGAGGUUUA	1.01	0.6	2.01	9.62	4.01	0.72	0	3.31	0.24	0	0	0
ath-miR2111b*	AUCCUCGGGAUACAGUUUACC	3.02	1.8	3.74	18.87	2.25	1.08	0.39	3.72	0.49	0	0.27	0.47
ath-miR2112-3p	CUUUAUAUCCGCAUUUGCGCA	20.14	15.2	11.8	9.8	18.53	10.05	20.44	44.63	0.49	0	0	0.24
ath-miR2112-5p	CGCAAAUGCGGAUAUCAAUGU	4.83	1.6	1.44	1.11	0.25	0	0	0.41	0	0	0	0
ath-miR2933a	GAAAUCGGAGAGGAAAUUCGCC	0	0	0	0	0	0	0	0	0	0	0	0
ath-miR2933b	GAAAUCGGAGAGGAAAUUCGCC	0	0	0	0	0	0	0	0	0	0	0	0
ath-miR2934-3p	CAUCCAAGGUGUUUGUAGAAA	1.01	0	0	0	0	0.36	0	0.21	0	0	0	0
ath-miR2934-5p	UCUUUCUGCAAACGCCUUGGA	3.22	0	0	0.18	10.26	1.08	0	0	0.24	0	0	0
ath-miR2936	CUUGAGAGAGAGAACACAGACG	0	0	0	0	0	0	0	0	0	0	0	0
ath-miR2937	AUAAGAGCUGUUGAAGGAGUC	0	0	0	0	0	0	0	0	0	0	0	0
ath-miR2938	GAUCUUUUGAGAGGGUUCCAG	0	0	0	0	0	0	0	0	0	0	0	0
ath-miR2939	UAACGCACAACACUAAGCCAU	0	0	0	0	0	0	0	0	0.24	0	0	0
ath-miR319a	UUGGACUGAAGGGAGCUCCCU	4223.04	779.19	207.51	736.11	20449.58	5570.55	702.32	4553.01	211.54	12.09	6.68	20.76
ath-miR319b	UUGGACUGAAGGGAGCUCCCU	4223.04	779.19	207.51	736.11	20449.58	5570.55	702.32	4553.01	211.54	12.09	6.68	20.76
ath-miR319c	UUGGACUGAAGGGAGCUCCUU	93.86	24.81	15.83	43.65	790.35	528.46	204.37	307.25	4.38	0.77	1.34	1.18
ath-miR3434	ACUUGGCUGAUUCUAUUAUU	2.22	1.4	2.01	1.29	0	0	0	0	0	0	0	0
ath-miR3434*	UCAGAGUAUCAGCCAUGUGA	2.62	1	0	1.29	0	0	0	0	0	0	0	0
ath-miR3440b-3p	UGGAUUGGUCAAGGGAAGCGU	0.6	0.2	0.86	0.18	2	0	0.79	0.62	0	0	0	0
ath-miR3440b-5p	UUUUCUUGGCCCAUCCACUUC	0.6	0.6	0.29	1.29	2.25	1.79	1.57	4.96	0	0	0	0
ath-miR390a	AAGCUCAGGAGGGAUAGCGCC	459.42	375.29	98.14	331.25	181.75	87.9	121.05	408.29	466.61	21.09	45.69	94.38
ath-miR390b	AAGCUCAGGAGGGAUAGCGCC	459.42	375.29	98.14	331.25	181.75	87.9	121.05	408.29	466.61	21.09	45.69	94.38
ath-miR391	UUCGCAGGAGAGAUAGCGCCA	38.27	30.21	1.15	6.47	50.82	96.51	0	41.95	2.92	0.51	0	0
ath-miR3932a	AACUUUGUGAUGACAACGAAG	0.2	2	0	2.77	0	0	0	0	0	0	0	0
ath-miR3932b	AACUUUGUGAUGACAACGAAG	0.2	2	0	2.77	0	0	0	0	0	0	0	0
ath-miR3933	AGAAGCAAAAUGACGACUCGG	1.41	0	0	0	1.25	0	0	0	0	0	0	0
ath-miR393a	UCCAAAGGGAUCGCAUUGAUCC	226.79	343.28	94.12	449.07	1019.67	1657.5	246.82	1972.87	21.88	0.51	1.6	9.91
ath-miR393b	UCCAAAGGGAUCGCAUUGAUCC	226.79	343.28	94.12	449.07	1019.67	1657.5	246.82	1972.87	21.88	0.51	1.6	9.91
ath-miR394a	UUGGCAUUCUGUCCACCUCC	1383.51	462.91	158.59	222.87	5063.08	1253.53	321.1	1788.35	70.03	1.54	2.4	4.72
ath-miR394b	UUGGCAUUCUGUCCACCUCC	1383.51	462.91	158.59	222.87	5063.08	1253.53	321.1	1788.35	70.03	1.54	2.4	4.72
ath-miR395a	CUGAAGUGUUUGGGGGAACUC	9.06	19	33.39	19.6	126.93	509.81	324.63	99.18	0.49	0.26	2.4	1.65
ath-miR395b	CUGAAGUGUUUGGGGGGACUC	4.63	20	60.44	10.73	65.84	357.69	393.02	87.4	0.73	2.06	5.08	0
ath-miR395c	CUGAAGUGUUUGGGGGGACUC	4.63	20	60.44	10.73	65.84	357.69	393.02	87.4	0.73	2.06	5.08	0
ath-miR395d	CUGAAGUGUUUGGGGGAACUC	9.06	19	33.39	19.6	126.93	509.81	324.63	99.18	0.49	0.26	2.4	1.65
ath-miR395e	CUGAAGUGUUUGGGGGAACUC	9.06	19	33.39	19.6	126.93	509.81	324.63	99.18	0.49	0.26	2.4	1.65
ath-miR395f	CUGAAGUGUUUGGGGGGACUC	4.63	20	60.44	10.73	65.84	357.69	393.02	87.4	0.73	2.06	5.08	0
ath-miR396a	UUCCACAGCUUUCUUGAACUG	390.54	1100.87	773.07	692.09	1451.03	6282.7	2063.35	4774.31	16.05	12.6	16.56	21.71
ath-miR396b	UUCCACAGCUUUCUUGAACUU	2931.98	3823.74	1539.8	1911.49	7402.34	8813.79	2035.84	8517.76	112.58	14.15	35.8	39.17
ath-miR397a	UCAUUGAGUGCAGCGUUGAUG	0	0.2	0	6.29	30.79	0	0	17.56	0	0	0	0.47
ath-miR397b	UCAUUGAGUGCAUCGUUGAUG	1.21	0	0	39.39	140.45	3.23	0	269.85	0	0	0	0
ath-miR398a	UGUGUUCUCAGGUCACCCCUU	0.6	0.4	0.86	2.77	3.5	18.3	2.36	7.85	0	0	0	0.24
ath-miR398b	UGUGUUCUCAGGUCACCCCUG	0.81	0.2	0	40.69	120.92	84.31	1.97	206.21	1.22	0	0	3.78
ath-miR398c	UGUGUUCUCAGGUCACCCCUG	0.81	0.2	0	40.69	120.92	84.31	1.97	206.21	1.22	0	0	3.78
ath-miR399a	UGCCAAAGGAGAUUUGCCCUG	1.01	1.4	1.15	21.08	29.54	2.51	0.39	24.18	0	0.26	0	0.24
ath-miR399b	UGCCAAAGGAGAGUUGCCCUG	18.53	9.4	4.61	61.4	353.24	33.37	36.16	115.3	1.7	0	0.27	0.47
ath-miR399c	UGCCAAAGGAGAGUUGCCCUG	18.53	9.4	4.61	61.4	353.24	33.37	36.16	115.3	1.7	0	0.27	0.47
ath-miR399d	UGCCAAAGGAGAUUUGCCCCG	0.2	0	0	19.97	23.28	0.36	0	26.03	0	0	0	0.24
ath-miR399e	UGCCAAAGGAGAUUUGCCUCG	0	0	0	0	0.5	0	0	0	0	0	0	0
ath-miR399f	UGCCAAAGGAGAUUUGCCCGG	2.22	1.6	1.15	91.74	89.63	6.46	1.97	200.22	0	0	0	1.18
ath-miR400	UAUGAGAGUAUUAUAAGUCAC	94.46	939.83	303.07	698.2	341.73	4847.99	836.74	8399.99	7.29	4.12	10.69	38.93
ath-miR401	CGAAACUGGUGUCGACCGACA	0	0	0	0	0	0	0	0	0	0	0	0
ath-miR402	UUCGAGGCCUAUUAAACCUCUG	6.24	5.4	3.17	2.59	8.01	7.89	1.57	7.85	0	0	0	0
ath-miR403	UUAGAUUCACGCACAAACUCG	281.37	395.7	437.76	274.84	777.84	1828.63	892.15	1946.21	17.51	6.43	13.63	15.1
ath-miR404	AUUAACGCUGGCGGUUGCGGCAGC	0	0	0	0	0	0	0	0	0	0	0	0
ath-miR405a	AUGAGUUGGGUCUAACCCAUAACU	0.2	0.8	0.29	0	0	0	0	0	0.73	0	0	0.24
ath-miR405b	AUGAGUUGGGUCUAACCCAUAACU	0.2	0.8	0.29	0	0	0	0	0	0.73	0	0	0.24
ath-miR405d	AUGAGUUGGGUCUAACCCAUAACU	0.2	0.8	0.29	0	0	0	0	0	0.73	0	0	0.24
ath-miR406	UAGAAUGCUAUUGUAAUCCAG	0	0	0	0	0	0.36	0	0	0	0	0	0
ath-miR407	UUUAAAUCAUAUACUUUUGGU	0	0	0	0	0	0	0	0	0	0	0	0
ath-miR408	AUGCACUGCCUCUUCCCUGGC	624.99	8	6.62	545.98	11.27	11.48	0.39	9.92	25.29	4.37	2.67	627.14
ath-miR413	AUAGUUUCUCUUGUUCUGCAC	0	0	0	0	0	0	0	0	0	0	0	0
ath-miR414	UCAUCUUCAUCAUCAUCGUCA	0	0	0	0	0	0	0	0	0	0	0	0
ath-miR415	AACAGAGCAGAAACAGAACAU	0	0	0	0	0	0	0	0	0	0	0	0
ath-miR416	GGUUCGUACGUACACUGUUCA	0	0	0	0	0	0	0	0	0	0	0	0
ath-miR417	GAAGGUAGUGAAUUUGUUCGA	0	0	0	0	0	0	0	0	0	0	0	0
ath-miR418	UAAUGUGAUGAUGAACUGACC	0	0	0	0	0	0	0	0	0	0	0	0
ath-miR419	UUAUGAAUGCUGAGGAUGUUG	0	0	0	0	0	0	0	0	0	0	0	0
ath-miR420	UAAACUAAUCACGGAAAUGCA	0	0	0	0	0	0	0	0	0	0	0	0
ath-miR4221	UUUUCCUCUGUUGAAUUCUUGC	0	0	0	0.18	0.5	0	0.79	0.21	0	0	0	0
ath-miR4227	UCACUGGUACCAAUCAUUCCA	0	0	0	0	0	0	0	0	0	0	0	0
ath-miR4228	AUAGCCUUGAACGCCGUCGUU	0	0	0	0	0	0	0	0	0	0	0	0
ath-miR4239	UUUGUUAUUUUCGCAUGCUCC	0.4	0	0	0	0	0	0	0	0	0	0	0
ath-miR4240	UGACUAGACCCGUAACAUUAC	0.2	0	0	0	0	0	0	0	0	0	0	0
ath-miR4243	UUGAAAUUGUAGAUUUCGUAC	1.21	0	0	0	2.25	0	0	0	0	0	0	0
ath-miR426	UUUUGGAAAUUUGUCCUUACG	0	0	0	0	0	0	0	0	0	0	0	0
ath-miR447a	UUGGGGACGAGAUGUUUUGUUG	24.37	9.4	3.17	5.73	135.69	112.29	25.94	44.63	1.22	1.03	0.27	0.47
ath-miR447a.2	UAUGGAAGAAAUUGUAGUAUU	666.07	218.45	96.13	102.46	1983.77	1074.14	269.22	711	35.74	2.57	2.4	2.36
ath-miR447b	UUGGGGACGAGAUGUUUUGUUG	24.37	9.4	3.17	5.73	135.69	112.29	25.94	44.63	1.22	1.03	0.27	0.47
ath-miR447c-3p	UUGGGGACGACAUCUUUUGUUG	0	0	0	0	0	0	0	0	0	0	0	0
ath-miR447c-5p	CCCCUUACAAUGUCGAGUAAA	1.01	0.2	0	0	0.25	0	0	0	0	0	0	0
ath-miR472	UUUUUCCUACUCCGCCCAUACC	20.75	24.61	16.69	18.13	227.32	120.19	93.54	224.4	4.38	3.86	3.47	2.36
ath-miR5012	UUUUACUGCUACUUGUGUUCC	7.05	7	2.01	3.7	36.3	46.28	11.4	39.88	0.73	0	0	0.24
ath-miR5013	UUUGUGACAUCUAGGUGCUUU	0	0	0	0	1.75	0	0	0	0	0	0	0
ath-miR5014	UUGUACAAAUUUAAGUGUACG	2.01	4.4	1.73	0.92	8.26	17.22	1.57	14.26	0.24	0	0	0
ath-miR5015a	UUGGUGUUAUGUGUAGUCUUC	0	0	0	0	0.75	0	0	0	0	0	0	0
ath-miR5015b	UCUGUUGUUGUUGGUGUUAUG	0	0	0	0	0.75	0	0	0	0	0	0	0
ath-miR5016	UUCUUGUGGAUUCCUUGGAAA	0	0	0	0	0	0	0	0	0	0	0	0
ath-miR5017	UUAUACCAAAUUAAUAGCAAA	11.48	0	0	0	17.02	0.36	0	0	1.7	0	0	0
ath-miR5018	UUAAAGCUCCACCAUGAGUCCAAU	0	0	0	0	3.76	0	0	0	0	0	0	0
ath-miR5019	UGUUGGGAAAGAAAAACUCUU	1.61	0.2	1.15	0.37	9.51	0.36	4.32	2.48	0.24	0	0	0
ath-miR5020a	UGGAAGAAGGUGAGACUUGCA	1.01	0.2	0.29	0.37	6.51	0.72	0.39	1.86	0	0	0	0.24
ath-miR5020b	AUGGCAUGAAAGAAGGUGAGA	0	0.6	0	0	0	0	0	0	0	0	0	0
ath-miR5021	UGAGAAGAAGAAGAAGAAAA	0	0	0	0	0	0	0	0	0	0	0	0
ath-miR5022	GUCAUGGGGUAUGAUCGAAUG	0	0	0	0	0	0	0	0	0	0	0	0
ath-miR5023	AUUGGUAGUGGAUAAGGGGGC	0	0.2	0	0	0	0	0	0	0	0	0	0
ath-miR5024	AUGACAAGGCCAAGAUAUAACA	0	0.2	0	0.18	0	0	0	0	0	0	0	0
ath-miR5025	ACUGUAUAUAUGUAAGUGACA	0	0	0	0	0	0	0	0	0	0	0	0
ath-miR5026	ACUCAUAAGAUCGUGACACGU	20.14	317.88	5.47	629.02	11.77	102.61	2.36	284.32	2.19	5.92	0.53	8.02
ath-miR5027	ACCGGUUGGAACUUGCCUUAA	0.6	0.2	0.29	0.37	0.5	0.36	0	0.21	0	0	0	0
ath-miR5028	AAUUGGGUUUAUGCUAGAGUU	11.68	4.2	24.46	3.14	16.52	0.72	15.33	13.64	0.24	0	1.34	0
ath-miR5029	AAUGAGAGAGAACACUGCAAA	0	0	0	0	0	0	0	0	0	0	0	0
ath-miR771	UGAGCCUCUGUGGUAGCCCUCA	19.94	0.4	0	0	95.38	0.36	0.39	0.41	1.7	0	0	0
ath-miR773	UUUGCUUCCAGCUUUUGUCUC	6.24	11.4	30.51	1.85	42.06	30.85	117.12	51.86	0.97	0.77	0.53	0.24
ath-miR773b	UUUGAUUCCAGCUUUUGUCUC	0.2	0	0	0	0	0	0	0	0	0	0	0
ath-miR773b*	GGCAAUAACUUGAGCAAACA	0	0	0	0	0	0	0	0	0	0	0	0
ath-miR774	UUGGUUACCCAUAUGGCCAUC	1.61	0.2	2.01	0.37	8.76	0.72	1.97	1.03	0	0	0	0
ath-miR774b	UGAGAUGAAGAUAUGGGUGAU	0	0	0	0	0	0	0	0	0	0	0	0
ath-miR774b*	CAUCCAUAUUUUCAUCUCGAA	0	0	0	0	0	0	0	0	0	0	0	0
ath-miR775	UUCGAUGUCUAGCAGUGCCA	127.9	124.83	108.22	57.52	236.83	334.37	226.77	781.46	6.81	0.77	3.74	4.95
ath-miR776	UCUAAGUCUUCUAUUGAUGUU	5.04	0.2	0	0	5.51	0	0	0	0	0	0	0
ath-miR777	UACGCAUUGAGUUUCGUUGCUU	22.16	18.8	11.8	15.35	36.05	45.56	13.36	47.73	0.24	0	0	0
ath-miR778	UGGCUUGGUUUAUGUACACCG	1.01	0	0	0.18	4.01	0	0	0	0	0	0	0
ath-miR779.1	UUCUGCUAUGUUGCUGCUCAU	0.6	0	0.58	0.18	0.25	0	0.39	2.48	0	0	0	0
ath-miR779.2	UGAUUGGAAAUUUCGUUGACU	8.26	8.6	14.68	2.96	46.31	25.47	26.73	9.5	0.49	0.26	0.27	0.47
ath-miR780.1	UCUAGCAGCUGUUGAGCAGGU	103.33	0	0	0	198.28	0.36	1.18	0.21	7.05	0	0	0
ath-miR780.2	UUCUUCGUGAAUAUCUGGCAU	597.39	1	2.59	0.37	2565.84	6.82	4.32	2.69	39.15	0.26	0	0.24
ath-miR781	UUAGAGUUUUCUGGAUACUUA	22.76	17	12.09	14.43	46.57	63.5	29.48	102.28	0.73	0	0.27	0
ath-miR782	ACAAACACCUUGGAUGUUCUU	0.2	0.2	0	0	0.25	0.36	0	0.62	0	0	0	0
ath-miR783	AAGCUUUGCUCGUUCAUGUUC	0	0	0	0.18	0	0	0	0	0	0.26	0	0
ath-miR822	UGCGGGAAGCAUUUGCACAUG	19.34	111.63	537.92	158.69	85.37	252.21	916.13	575.87	1.22	7.97	12.02	1.18
ath-miR823	UGGGUGGUGAUCAUAUAAGAU	54.99	95.62	69.94	89.52	321.2	868.93	269.22	778.57	3.89	2.83	1.87	9.44
ath-miR824	UAGACCAUUUGUGAGAAGGGA	742.01	505.92	1807.47	308.13	524.48	659.77	746.34	1973.7	18.48	3.09	12.56	7.31
ath-miR825	UUCUCAAGAAGGUGCAUGAAC	17.12	79.42	0.29	46.24	54.58	237.86	1.97	310.15	0.49	0.77	0	1.89
ath-miR826	UAGUCCGGUUUUGGAUACGUG	4.83	0	0	1.48	8.51	0.36	0	0.41	0	0	0	0
ath-miR827	UUAGAUGACCAUCAACAAACU	16.11	26.01	5.47	45.87	233.83	123.42	7.86	301.88	3.65	0.26	0.27	4.72
ath-miR828	UCUUGCUUAAAUGAGUAUUCCA	2.22	41.61	0.86	2.4	6.26	97.23	0	10.33	0	0	0	0.24
ath-miR829.1	AGCUCUGAUACCAAAUGAUGGAAU	9.67	3.6	94.12	21.45	0.75	0.36	38.91	6.82	12.89	39.1	334.23	25.01
ath-miR829.2	CAAAUUAAAGCUUCAAGGUAG	4.23	1.2	58.14	6.29	6.01	1.79	54.24	82.03	0	0	2.14	0
ath-miR830	UAACUAUUUUGAGAAGAAGUG	6.65	6.8	5.47	3.14	22.78	11.84	7.47	20.25	0.49	0	0.27	0
ath-miR830*	UCUUCUCCAAAUAGUUUAGGUU	5.44	6.6	3.45	3.33	4.76	14.71	2.75	14.05	0.49	0	0	0
ath-miR831	UGAUCUCUUCGUACUCUUCUUG	0	0	0	0	0	0	0	0	0	0	0	0
ath-miR832-3p	UUGAUUCCCAAUCCAAGCAAG	1.61	0	0	0.18	13.52	0	0	0	0	0	0	0.24
ath-miR832-5p	UGCUGGGAUCGGGAAUCGAAA	0	0	0	0	4.51	0	0	0	0	0	0	0
ath-miR833-3p	UAGACCGAUGUCAACAAACAAG	8.46	4.2	1.44	3.7	41.81	15.43	2.36	69.01	0.24	0	0	0.24
ath-miR833-5p	UGUUUGUUGUACUCGGUCUAGU	1.61	1.2	0.86	1.11	9.26	3.23	0.39	0.83	0	0	0.27	0
ath-miR834	UGGUAGCAGUAGCGGUGGUAA	0	0	0	0	0	0	0	0	0	0	0	0
ath-miR835-3p	UGGAGAAGAUACGCAAGAAAG	0	0	0	0.18	0	0	0	0	0	0	0	0
ath-miR835-5p	UUCUUGCAUAUGUUCUUUAUC	1.41	1.6	0.29	0.18	3	4.31	1.57	4.96	0	0	0	0
ath-miR836	UCCUGUGUUUCCUUUGAUGCGUGG	0	0	0	0	0	0	0	0	0	0	0	0
ath-miR837-3p	AAACGAACAAAAAACUGAUGG	1.01	3	24.46	10.36	0.5	0.72	3.14	6.2	0	0	0.27	0
ath-miR837-5p	AUCAGUUUCUUGUUCGUUUCA	0.2	1	12.09	6.47	0	0.36	7.47	3.51	0	0	0.27	0
ath-miR838	UUUUCUUCUACUUCUUGCACA	3.83	12.6	4.03	7.58	4.76	15.79	10.22	32.65	0.24	0	0.27	0.24
ath-miR839	UACCAACCUUUCAUCGUUCCC	118.63	7.4	3.17	2.59	261.11	18.3	7.86	29.96	9.24	0	0.53	0.71

ath-miR840	ACACUGAAGGACCUAAACUAAC	5.64	20.81	4.32	5.55	3.5	2.51	2.75	3.31	1.95	2.57	3.74	1.89
ath-miR841	UACGAGCCACUUGAAACUGAA	0.81	12.2	0	34.03	2.25	84.67	0	134.31	0.24	0.26	0.27	0
ath-miR841b	UACGAGCCACUGGAAACUGAA	0.4	0.2	0.29	0.37	3	8.25	2.36	17.36	0	0	0	0
ath-miR841b*	CAAUUUCUAGUGGGUCGUAUU	0.2	1.2	0	1.66	0	1.44	0	2.89	0	0	0	0
ath-miR842	UCAUGGUCAGAUCCGUCAUCC	0.2	1.4	18.13	1.85	3.76	10.05	63.28	68.19	0	0	0.27	0.24
ath-miR843	UUUAGGUCGAGCUUCAUUGGA	0.81	89.02	0.29	3.88	5.76	42.69	0	52.69	0.49	0	0	4.95
ath-miR844	UGGUAAGAUUGCUUAUAAGCU	5.84	8	4.03	6.84	19.53	23.68	4.32	28.72	0.49	0	0	0.24
ath-miR844*	UUAUAAGCCAUCUUACUAGUU	13.09	17.6	8.35	11.84	14.02	22.6	2.75	17.77	1.22	0	0	0.24
ath-miR845a	CGGCUCUGAUACCAAUUGAUG	54.58	5.4	1.73	3.88	428.85	7.89	1.97	33.06	3.65	0.26	0	0
ath-miR845b	UCGCUCUGAUACCAAAUUGAUG	9.47	0	0	0	43.31	1.08	0	0	0	0	0	0
ath-miR846	UUGAAUUGAAGUGCUUGAAUU	9.87	51.01	772.49	132.06	60.08	396.44	1781.16	1040.57	0.49	3.34	17.9	1.18
ath-miR847	UCACUCCUCUUCUUCUUGAUG	5.24	19.2	0	5.92	12.77	63.5	0	60.95	0	0	0	0.71
ath-miR848	UGACAUGGGACUGCCUAAGCUA	1.01	1	0.58	1.85	7.01	3.59	3.54	13.84	0.24	0	0	0
ath-miR849	UAACUAAACAUUGGUGUAGUA	0.2	0.6	0	0.18	0	1.08	0	1.86	0	0	0	0
ath-miR850	UAAGAUCCGGACUACAACAAAG	0.4	3	0	1.48	1	9.33	0	14.26	0	0.77	0	0.24
ath-miR851-3p	UGGGUGGCAAACAAAGACGAC	3.02	0	0	0	2.25	0	0	0	0	0	0	0
ath-miR851-5p	UCUCGGUUCGCGAUCCACAAG	20.14	1	0	0.18	166.23	4.66	1.18	2.27	4.13	0	0	0
ath-miR852	AAGAUAAGCGCCUUAGUUCUG	1.21	5	1.15	4.25	0	1.08	0.79	3.1	0.24	0	0.53	0
ath-miR853	UCCCCUCUUUAGCUUGGAGAAG	5.64	7.2	4.89	2.77	14.27	22.6	10.22	29.34	0.73	0	0	0.24
ath-miR854a	GAUGAGGAUAGGGAGGAGGAG	0	0	0	0	0	0	0	0	0	0	0	0
ath-miR854b	GAUGAGGAUAGGGAGGAGGAG	0	0	0	0	0	0	0	0	0	0	0	0
ath-miR854c	GAUGAGGAUAGGGAGGAGGAG	0	0	0	0	0	0	0	0	0	0	0	0
ath-miR854d	GAUGAGGAUAGGGAGGAGGAG	0	0	0	0	0	0	0	0	0	0	0	0
ath-miR854e	GAUGAGGAUAGGGAGGAGGAG	0	0	0	0	0	0	0	0	0	0	0	0
ath-miR855	AGCAAAAGCUAAGGAAAAGGAA	0	0	0	0	0	0	0	0	0	0	0	0
ath-miR856	UAAUCCUACCAAUAACUUCAGC	28	0	0	0	16.02	0	0	0	4.38	0	0	0
ath-miR857	UUUUGUAUGUUGAAGGUGUAU	0	0	0	14.06	13.77	2.15	0	94.63	0	0	0	0.24
ath-miR858	UUUCGUUGUCUGUUCGACCUU	54.58	85.82	10.65	13.13	299.17	213.82	20.83	48.35	2.92	0.51	0	0.71
ath-miR859	UCUCUCUGUUGUGAAGUCAAA	15.31	1.4	4.32	0.92	10.01	1.44	9.83	3.51	0.24	0	0	0
ath-miR860	UCAAUAGAUUGGACUAUGUAU	12.49	8	16.69	39.76	0.25	0.36	19.65	42.77	2.67	0.26	0.27	2.36
ath-miR861-3p	GAUGGAUAUGUCUUCAAGGAC	4.83	4.2	0.29	5.73	0	0	0	0	0	0	0	0
ath-miR861-5p	CCUUGGAGAAAUAUGCGUCAA	0.4	0	0	0	0	0	0	0.21	0	0	0	0
ath-miR862-3p	AUAUGCUGGAUCUACUUGAAG	0	0	0	0	0	0	0	0	0	0	0	0
ath-miR862-5p	UCCAAUAGGUCGAGCAUGUGC	0.4	0.8	0.29	2.22	4.01	6.46	1.57	13.22	0	0	0	0
ath-miR863-3p	UUGAGAGCAACAAGACAUAAU	1.21	27.21	0.58	25.15	0.25	39.46	0	103.93	0	0	0	0
ath-miR863-5p	UUAUGUCUUGUUGAUCUCAAU	0	2.4	0	1.48	0	8.61	0.79	4.55	0	0	0	0
ath-miR864-3p	UAAAGUCAAUAAUACCUUGAAG	0.81	1.2	0.29	0.37	0.75	1.79	0	2.89	0	0.26	0	0
ath-miR864-5p	UCAGGUAUGAUUGACUUCAAA	12.08	16.8	6.04	11.47	40.56	47	7.07	81.82	0	0	0.27	0
ath-miR865-3p	UUUUUCCUCAAAUUUAUCCAA	0	0.4	0	0.37	0.25	0	0.39	0.41	0	0	0	0
ath-miR865-5p	AUGAAUUUGGAUCUAAUUGAG	1.21	1.6	1.44	0.18	0	0	0	0	0	0	0	0
ath-miR866-3p	ACAAAAUCCGUCUUUGAAGA	1.41	0.6	0.58	0.55	0	0.36	0	0.21	0	0	0	0
ath-miR866-5p	UCAAGGAACGGAUUUUGUUAA	0.81	0.6	0.58	0.55	2.75	0.72	0.39	1.86	0	0	0	0.24
ath-miR867	UUGAACAUGGUUUAUUAGGAA	82.58	0	0	0	286.4	0.36	0	0	4.38	0	0	0
ath-miR868-3p	CUUCUUAAGUGCUGAUAAUGC	1.61	0.2	0	0	7.01	0.36	0	0.41	0	0	0	0
ath-miR868-5p	UCAUGUCGUAAUAGUAGUCAC	5.24	0.4	0.29	0.18	16.27	1.44	1.18	0.41	0.24	0	0	0
ath-miR869.1	AUUGGUUCAAUUCUGGUGUUG	0	0	0.58	0	0	0	0	0	0	0	0	0
ath-miR869.2	UCUGGUGUUGAGAUAGUUGAC	6.24	0.6	64.18	13.13	30.54	19.73	159.57	146.5	0.73	0	1.87	1.65
ath-miR870	UAAUUUGGUGUUUCUUCGAUC	0.2	0.2	0	0.55	2	1.44	0.79	1.03	0	0	0	0
