# Supplementary material for: Expression-Based Functional Investigation of the Organ-Specific MicroRNAs in Arabidopsis
Source: PLoS One. 2012 Nov 30;7(11):e50870. doi: 10.1371/journal.pone.0050870 (PMC3511311; doi:10.1371/journal.pone.0050870)
Supplement: Table S2 — List of the organ-specific microRNAs identified from the WT (wild type)-related library group. For each organ-specific microRNA selected, the expression level in a specific organ (highlighted by different background colors) should be three times or more higher than the other two or three organs. The high-throughput sequencing data sets were retrieved from GEO (Gene Expression Omnibus; http://www.ncbi.nlm.nih.gov/geo/) [68]: WT_Flower (GSM707678), WT_Leaf (GSM707679), WT_Root (GSM707680), WT_Seedling (GSM707681). The expression levels were shown by normalized read counts (in RPM; reads per million). (PDF) [file pone.0050870.s009.pdf]

| miRNA | Sequence | WT_Flower | WT_Leaf | WT_Root | WT_Seedling |
|---|---|---|---|---|---|
| ath-miR156a | UGACAGAAGAGAGUGAGCAC | 108.36 | 238.06 | 51.23 | 1495.53 |
| ath-miR156b | UGACAGAAGAGAGUGAGCAC | 108.36 | 238.06 | 51.23 | 1495.53 |
| ath-miR156c | UGACAGAAGAGAGUGAGCAC | 108.36 | 238.06 | 51.23 | 1495.53 |
| ath-miR156d | UGACAGAAGAGAGUGAGCAC | 108.36 | 238.06 | 51.23 | 1495.53 |
| ath-miR156e | UGACAGAAGAGAGUGAGCAC | 108.36 | 238.06 | 51.23 | 1495.53 |
| ath-miR156f | UGACAGAAGAGAGUGAGCAC | 108.36 | 238.06 | 51.23 | 1495.53 |
| ath-miR156h | UGACAGAAGAAAGAGAGCAC | 13.29 | 0.2 | 0.29 | 0.18 |
| ath-miR157a | UUGACAGAAGAUAGAGAGCAC | 89.02 | 715.58 | 2.59 | 1656.07 |
| ath-miR157b | UUGACAGAAGAUAGAGAGCAC | 89.02 | 715.58 | 2.59 | 1656.07 |
| ath-miR157c | UUGACAGAAGAUAGAGAGCAC | 89.02 | 715.58 | 2.59 | 1656.07 |
| ath-miR157d | UGACAGAAGAUAGAGAGCAC | 5.64 | 22.41 | 0 | 46.98 |
| ath-miR163 | UUGAAGAGGACUUGGAACUUCGAU | 3690.3 | 8029.77 | 2072.83 | 2218.51 |
| ath-miR167c | UAAGCUGCCAGCAUGAUCUUG | 1.21 | 2.2 | 123.76 | 17.02 |
| ath-miR167d | UGAAGCUGCCAGCAUGAUCUGG | 44.31 | 290.67 | 3.17 | 223.98 |
| ath-miR169a | CAGCCAAGGAUGACUUGCCGA | 54.18 | 254.06 | 225.36 | 50.86 |
| ath-miR169b | CAGCCAAGGAUGACUUGCCGG | 4.23 | 3.8 | 90.37 | 22.01 |
| ath-miR169c | CAGCCAAGGAUGACUUGCCGG | 4.23 | 3.8 | 90.37 | 22.01 |
| ath-miR169h | UAGCCAAGGAUGACUUGCCUG | 7.65 | 38.41 | 39.43 | 10.73 |
| ath-miR169i | UAGCCAAGGAUGACUUGCCUG | 7.65 | 38.41 | 39.43 | 10.73 |
| ath-miR169j | UAGCCAAGGAUGACUUGCCUG | 7.65 | 38.41 | 39.43 | 10.73 |
| ath-miR169k | UAGCCAAGGAUGACUUGCCUG | 7.65 | 38.41 | 39.43 | 10.73 |
| ath-miR169l | UAGCCAAGGAUGACUUGCCUG | 7.65 | 38.41 | 39.43 | 10.73 |
| ath-miR169m | UAGCCAAGGAUGACUUGCCUG | 7.65 | 38.41 | 39.43 | 10.73 |
| ath-miR169n | UAGCCAAGGAUGACUUGCCUG | 7.65 | 38.41 | 39.43 | 10.73 |
| ath-miR172b* | GCAGCACCAUUAAGAUUCAC | 22.56 | 76.82 | 5.18 | 22.01 |
| ath-miR172c | AGAAUCUUGAUGAUGCUGCAG | 617.94 | 6.8 | 562.68 | 16.28 |
| ath-miR172d | AGAAUCUUGAUGAUGCUGCAG | 617.94 | 6.8 | 562.68 | 16.28 |
| ath-miR172e | GGAAUCUUGAUGAUGCUGCAU | 33.43 | 5.4 | 260.47 | 26.63 |
| ath-miR2111a* | GUCCUCGGGAUGCGGAUUACC | 0 | 0 | 0.86 | 11.84 |
| ath-miR2111b* | AUCCUCGGGAUACAGUUUACC | 3.02 | 1.8 | 3.74 | 18.87 |
| ath-miR319a | UUGGACUGAAGGGAGCUCCCU | 4223.04 | 779.19 | 207.51 | 736.11 |
| ath-miR319b | UUGGACUGAAGGGAGCUCCCU | 4223.04 | 779.19 | 207.51 | 736.11 |
| ath-miR319c | UUGGACUGAAGGGAGCUCCUU | 93.86 | 24.81 | 15.83 | 43.65 |
| ath-miR391 | UUCGCAGGAGAGAUAGCGCCA | 38.27 | 30.21 | 1.15 | 6.47 |
| ath-miR394a | UUGGCAUUCUGUCCACCUCC | 1383.51 | 462.91 | 158.59 | 222.87 |
| ath-miR394b | UUGGCAUUCUGUCCACCUCC | 1383.51 | 462.91 | 158.59 | 222.87 |
| ath-miR395b | CUGAAGUGUUUGGGGGGACUC | 4.63 | 20 | 60.44 | 10.73 |
| ath-miR395c | CUGAAGUGUUUGGGGGGACUC | 4.63 | 20 | 60.44 | 10.73 |
| ath-miR395f | CUGAAGUGUUUGGGGGGACUC | 4.63 | 20 | 60.44 | 10.73 |
| ath-miR397b | UCAUUGAGUGCAUCGUUGAUG | 1.21 | 0 | 0 | 39.39 |
| ath-miR398b | UGUGUUCUCAGGUCACCCCUG | 0.81 | 0.2 | 0 | 40.69 |
| ath-miR398c | UGUGUUCUCAGGUCACCCCUG | 0.81 | 0.2 | 0 | 40.69 |
| ath-miR399a | UGCCAAAGGAGAUUUGCCCUG | 1.01 | 1.4 | 1.15 | 21.08 |
| ath-miR399b | UGCCAAAGGAGAGUUGCCCUG | 18.53 | 9.4 | 4.61 | 61.4 |
| ath-miR399c | UGCCAAAGGAGAGUUGCCCUG | 18.53 | 9.4 | 4.61 | 61.4 |
| ath-miR399d | UGCCAAAGGAGAUUUGCCCCG | 0.2 | 0 | 0 | 19.97 |
| ath-miR399f | UGCCAAAGGAGAUUUGCCCGG | 2.22 | 1.6 | 1.15 | 91.74 |
| ath-miR400 | UAUGAGAGUAUUAUAAGUCAC | 94.46 | 939.83 | 303.07 | 698.2 |
| ath-miR408 | AUGCACUGCCUCUUCCCUGGC | 624.99 | 8 | 6.62 | 545.98 |
| ath-miR447a | UUGGGGACGAGAUGUUUUGUUG | 24.37 | 9.4 | 3.17 | 5.73 |
| ath-miR447a.2 | UAUGGAAGAAAUUGUAGUAUU | 666.07 | 218.45 | 96.13 | 102.46 |
| ath-miR447b | UUGGGGACGAGAUGUUUUGUUG | 24.37 | 9.4 | 3.17 | 5.73 |
| ath-miR5017 | UUAUACCAAAUUAAUAGCAAA | 11.48 | 0 | 0 | 0 |
| ath-miR5026 | ACUCAUAAGAUCGUGACACGU | 20.14 | 317.88 | 5.47 | 629.02 |
| ath-miR5028 | AAUUGGGUUUAUGCUAGAGUU | 11.68 | 4.2 | 24.46 | 3.14 |
| ath-miR771 | UGAGCCUCUGUGGUAGCCCUCA | 19.94 | 0.4 | 0 | 0 |
| ath-miR773 | UUUGCUUCCAGCUUUUGUCUC | 6.24 | 11.4 | 30.51 | 1.85 |
| ath-miR780.1 | UCUAGCAGCUGUUGAGCAGGU | 103.33 | 0 | 0 | 0 |
| ath-miR780.2 | UUCUUCGUGAAUAUCUGGCAU | 597.39 | 1 | 2.59 | 0.37 |
| ath-miR822 | UGCGGGAAGCAUUUGCACAUG | 19.34 | 111.63 | 537.92 | 158.69 |
| ath-miR824 | UAGACCAUUUGUGAGAAGGGA | 742.01 | 505.92 | 1807.47 | 308.13 |
| ath-miR825 | UUCUCAAGAAGGUGCAUGAAC | 17.12 | 79.42 | 0.29 | 46.24 |
| ath-miR828 | UCUUGCUUAAAUGAGUAUUCCA | 2.22 | 41.61 | 0.86 | 2.4 |
| ath-miR829.1 | AGCUCUGAUACCAAAUGAUGGAAU | 9.67 | 3.6 | 94.12 | 21.45 |
| ath-miR829.2 | CAAAUUAAAGCUUCAAGGUAG | 4.23 | 1.2 | 58.14 | 6.29 |
| ath-miR837-3p | AAACGAACAAAAAACUGAUGG | 1.01 | 3 | 24.46 | 10.36 |
| ath-miR837-5p | AUCAGUUUCUUGUUCGUUUCA | 0.2 | 1 | 12.09 | 6.47 |
| ath-miR838 | UUUUCUUCUACUUCUUGCACA | 3.83 | 12.6 | 4.03 | 7.58 |
| ath-miR839 | UACCAACCUUUCAUCGUUCCC | 118.63 | 7.4 | 3.17 | 2.59 |
| ath-miR840 | ACACUGAAGGACCUAAACUAAC | 5.64 | 20.81 | 4.32 | 5.55 |
| ath-miR841 | UACGAGCCACUUGAAACUGAA | 0.81 | 12.2 | 0 | 34.03 |
| ath-miR842 | UCAUGGUCAGAUCCGUCAUCC | 0.2 | 1.4 | 18.13 | 1.85 |
| ath-miR843 | UUUAGGUCGAGCUUCAUUGGA | 0.81 | 89.02 | 0.29 | 3.88 |
| ath-miR845a | CGGCUCUGAUACCAAUUGAUG | 54.58 | 5.4 | 1.73 | 3.88 |
| ath-miR846 | UUGAAUUGAAGUGCUUGAAUU | 9.87 | 51.01 | 772.49 | 132.06 |
| ath-miR847 | UCACUCCUCUUCUUCUUGAUG | 5.24 | 19.2 | 0 | 5.92 |
| ath-miR851-5p | UCUCGGUUCGCGAUCCACAAG | 20.14 | 1 | 0 | 0.18 |
| ath-miR856 | UAAUCCUACCAAUAACUUCAGC | 28 | 0 | 0 | 0 |
| ath-miR857 | UUUUGUAUGUUGAAGGUGUAU | 0 | 0 | 0 | 14.06 |
| ath-miR858 | UUUCGUUGUCUGUUCGACCUU | 54.58 | 85.82 | 10.65 | 13.13 |
| ath-miR859 | UCUCUCUGUUGUGAAGUCAAA | 15.31 | 1.4 | 4.32 | 0.92 |
| ath-miR860 | UCAAUAGAUUGGACUAUGUAU | 12.49 | 8 | 16.69 | 39.76 |
| ath-miR863-3p | UUGAGAGCAACAAGACAUAAU | 1.21 | 27.21 | 0.58 | 25.15 |
| ath-miR867 | UUGAACAUGGUUUAUUAGGAA | 82.58 | 0 | 0 | 0 |
| ath-miR869.2 | UCUGGUGUUGAGAUAGUUGAC | 6.24 | 0.6 | 64.18 | 13.13 |
