# Supplementary material for: Expression-Based Functional Investigation of the Organ-Specific MicroRNAs in Arabidopsis
Source: PLoS One. 2012 Nov 30;7(11):e50870. doi: 10.1371/journal.pone.0050870 (PMC3511311; doi:10.1371/journal.pone.0050870)
Supplement: Table S3 — List of the organ-specific microRNAs identified from the AGO1 (ARGONAUTE 1)-related library group. For each organ-specific microRNA selected, the expression level in a specific organ (highlighted by different background colors) should be three times or more higher than the other two or three organs. All the high-throughput sequencing data sets were retrieved from GEO (Gene Expression Omnibus; http://www.ncbi.nlm.nih.gov/geo/) [68]: AGO1_Flower (GSM707682), AGO1_Leaf (GSM707683), AGO1_Root (GSM707684), AGO1_Seedling (GSM707685). The expression levels were shown by normalized read counts (in RPM; reads per million). (PDF) [file pone.0050870.s010.pdf]

miRNA	Sequence	AGO1_Flower	AGO1_Leaf	AGO1_Root	AGO1_Seedling
ath-miR156a	UGACAGAAGAGAGUGAGCAC	199.78	341.54	46.38	5994.85
ath-miR156b	UGACAGAAGAGAGUGAGCAC	199.78	341.54	46.38	5994.85
ath-miR156c	UGACAGAAGAGAGUGAGCAC	199.78	341.54	46.38	5994.85
ath-miR156d	UGACAGAAGAGAGUGAGCAC	199.78	341.54	46.38	5994.85
ath-miR156e	UGACAGAAGAGAGUGAGCAC	199.78	341.54	46.38	5994.85
ath-miR156f	UGACAGAAGAGAGUGAGCAC	199.78	341.54	46.38	5994.85
ath-miR156h	UGACAGAAGAAAGAGAGCAC	67.59	0.72	0	0.83
ath-miR157a	UUGACAGAAGAUAGAGAGCAC	167.23	1637.4	3.14	7452.19
ath-miR157b	UUGACAGAAGAUAGAGAGCAC	167.23	1637.4	3.14	7452.19
ath-miR157c	UUGACAGAAGAUAGAGAGCAC	167.23	1637.4	3.14	7452.19
ath-miR157d	UGACAGAAGAUAGAGAGCAC	9.26	41.62	0	211.59
ath-miR158a	UCCCAAAUGUAGACAAAGCA	41217.85	143709.88	63787.33	235267.58
ath-miR158b	CCCCAAAUGUAGACAAAGCA	130.68	303.87	305.77	1113.51
ath-miR159b	UUUGGAUUGAAGGGAGCUCUU	27199.01	110106.13	13623.99	24839.19
ath-miR159c	UUUGGAUUGAAGGGAGCUCCU	50.32	171.13	31.83	20.04
ath-miR161.2	UCAAUGCAUUGAAAGUGACUA	4549.61	2939	2979.47	12175.67
ath-miR164c	UGGAGAAGCAGGGCACGUGCG	8.01	20.45	4.72	27.07
ath-miR167c	UAAGCUGCCAGCAUGAUCUUG	3.25	8.97	300.27	395.28
ath-miR167d	UGAAGCUGCCAGCAUGAUCUGG	137.44	1171.73	5.9	1137.27
ath-miR169a	CAGCCAAGGAUGACUUGCCGA	16.27	85.74	92.36	387.84
ath-miR169b	CAGCCAAGGAUGACUUGCCGG	13.02	11.48	108.47	200.63
ath-miR169c	CAGCCAAGGAUGACUUGCCGG	13.02	11.48	108.47	200.63
ath-miR170	UGAUUGAGCCGUGUCAAUAUC	126.68	40.9	0.79	185.34
ath-miR171a	UGAUUGAGCCGCGCCAAUAUC	2174.29	370.25	1.18	1323.03
ath-miR172a	AGAAUCUUGAUGAUGCUGCAU	5566.03	2707.96	540.01	1100.08
ath-miR172b	AGAAUCUUGAUGAUGCUGCAU	5566.03	2707.96	540.01	1100.08
ath-miR172c	AGAAUCUUGAUGAUGCUGCAG	2861.5	42.33	863.46	36.99
ath-miR172d	AGAAUCUUGAUGAUGCUGCAG	2861.5	42.33	863.46	36.99
ath-miR172e	GGAAUCUUGAUGAUGCUGCAU	158.47	19.01	117.12	36.99
ath-miR2934-5p	UCUUUCUGCAAACGCCUUGGA	10.26	1.08	0	0
ath-miR319a	UUGGACUGAAGGGAGCUCCCU	20449.58	5570.55	702.32	4553.01
ath-miR319b	UUGGACUGAAGGGAGCUCCCU	20449.58	5570.55	702.32	4553.01
ath-miR390a	AAGCUCAGGAGGGAUAGCGCC	181.75	87.9	121.05	408.29
ath-miR390b	AAGCUCAGGAGGGAUAGCGCC	181.75	87.9	121.05	408.29
ath-miR394a	UUGGCAUUCUGUCCACCUCC	5063.08	1253.53	321.1	1788.35
ath-miR394b	UUGGCAUUCUGUCCACCUCC	5063.08	1253.53	321.1	1788.35
ath-miR395a	CUGAAGUGUUUGGGGGAACUC	126.93	509.81	324.63	99.18
ath-miR395b	CUGAAGUGUUUGGGGGGACUC	65.84	357.69	393.02	87.4
ath-miR395c	CUGAAGUGUUUGGGGGGACUC	65.84	357.69	393.02	87.4
ath-miR395d	CUGAAGUGUUUGGGGGAACUC	126.93	509.81	324.63	99.18
ath-miR395e	CUGAAGUGUUUGGGGGAACUC	126.93	509.81	324.63	99.18
ath-miR395f	CUGAAGUGUUUGGGGGGACUC	65.84	357.69	393.02	87.4
ath-miR396a	UUCCACAGCUUUCUUGAACUG	1451.03	6282.7	2063.35	4774.31
ath-miR397a	UCAUUGAGUGCAGCGUUGAUG	30.79	0	0	17.56
ath-miR397b	UCAUUGAGUGCAUCGUUGAUG	140.45	3.23	0	269.85
ath-miR398a	UGUGUUCUCAGGUCACCCCUU	3.5	18.3	2.36	7.85
ath-miR399a	UGCCAAAGGAGAUUUGCCCUG	29.54	2.51	0.39	24.18
ath-miR399b	UGCCAAAGGAGAGUUGCCCUG	353.24	33.37	36.16	115.3
ath-miR399c	UGCCAAAGGAGAGUUGCCCUG	353.24	33.37	36.16	115.3
ath-miR399d	UGCCAAAGGAGAUUUGCCCCG	23.28	0.36	0	26.03
ath-miR399f	UGCCAAAGGAGAUUUGCCCGG	89.63	6.46	1.97	200.22
ath-miR400	UAUGAGAGUAUUAUAAGUCAC	341.73	4847.99	836.74	8399.99
ath-miR447a	UUGGGGACGAGAUGUUUUGUUG	135.69	112.29	25.94	44.63
ath-miR447b	UUGGGGACGAGAUGUUUUGUUG	135.69	112.29	25.94	44.63
ath-miR5017	UUAUACCAAAUUAAUAGCAAA	17.02	0.36	0	0
ath-miR5026	ACUCAUAAGAUCGUGACACGU	11.77	102.61	2.36	284.32
ath-miR771	UGAGCCUCUGUGGUAGCCCUCA	95.38	0.36	0.39	0.41
ath-miR775	UUCGAUGUCUAGCAGUGCCA	236.83	334.37	226.77	781.46
ath-miR780.1	UCUAGCAGCUGUUGAGCAGGU	198.28	0.36	1.18	0.21
ath-miR780.2	UUCUUCGUGAAUAUCUGGCAU	2565.84	6.82	4.32	2.69
ath-miR822	UGCGGGAAGCAUUUGCACAUG	85.37	252.21	916.13	575.87
ath-miR825	UUCUCAAGAAGGUGCAUGAAC	54.58	237.86	1.97	310.15
ath-miR828	UCUUGCUUAAAUGAGUAUUCCA	6.26	97.23	0	10.33
ath-miR829.1	AGCUCUGAUACCAAAUGAUGGAA	0.75	0.36	38.91	6.82
ath-miR829.2	CAAAUUAAAGCUUCAAGGUAG	6.01	1.79	54.24	82.03
ath-miR830*	UCUUCUCCAAAUAGUUUAGGUU	4.76	14.71	2.75	14.05
ath-miR832-3p	UUGAUUCCCAAUCCAAGCAAG	13.52	0	0	0
ath-miR833-3p	UAGACCGAUGUCAACAAACAAG	41.81	15.43	2.36	69.01
ath-miR838	UUUUCUUCUACUUCUUGCACA	4.76	15.79	10.22	32.65
ath-miR839	UACCAACCUUUCAUCGUUCCC	261.11	18.3	7.86	29.96
ath-miR841	UACGAGCCACUUGAAACUGAA	2.25	84.67	0	134.31
ath-miR841b	UACGAGCCACUGGAAACUGAA	3	8.25	2.36	17.36
ath-miR842	UCAUGGUCAGAUCCGUCAUCC	3.76	10.05	63.28	68.19
ath-miR843	UUUAGGUCGAGCUUCAUUGGA	5.76	42.69	0	52.69
ath-miR845a	CGGCUCUGAUACCAAUUGAUG	428.85	7.89	1.97	33.06
ath-miR845b	UCGCUCUGAUACCAAAUUGAUG	43.31	1.08	0	0
ath-miR846	UUGAAUUGAAGUGCUUGAAUU	60.08	396.44	1781.16	1040.57
ath-miR847	UCACUCCUCUUCUUCUUGAUG	12.77	63.5	0	60.95
ath-miR848	UGACAUGGGACUGCCUAAGCUA	7.01	3.59	3.54	13.84
ath-miR850	UAAGAUCCGGACUACAACAAAG	1	9.33	0	14.26
ath-miR851-5p	UCUCGGUUCGCGAUCCACAAG	166.23	4.66	1.18	2.27
ath-miR856	UAAUCCUACCAAUAACUUCAGC	16.02	0	0	0
ath-miR857	UUUUGUAUGUUGAAGGUGUAU	13.77	2.15	0	94.63
ath-miR858	UUUCGUUGUCUGUUCGACCUU	299.17	213.82	20.83	48.35
ath-miR860	UCAAUAGAUUGGACUAUGUAU	0.25	0.36	19.65	42.77
ath-miR862-5p	UCCAAUAGGUCGAGCAUGUGC	4.01	6.46	1.57	13.22
ath-miR863-3p	UUGAGAGCAACAAGACAUAAU	0.25	39.46	0	103.93
ath-miR867	UUGAACAUGGUUUAUUAGGAA	286.4	0.36	0	0
ath-miR868-5p	UCAUGUCGUAAUAGUAGUCAC	16.27	1.44	1.18	0.41
ath-miR869.2	UCUGGUGUUGAGAUAGUUGAC	30.54	19.73	159.57	146.5
