# Supplementary material for: Expression-Based Functional Investigation of the Organ-Specific MicroRNAs in Arabidopsis
Source: PLoS One. 2012 Nov 30;7(11):e50870. doi: 10.1371/journal.pone.0050870 (PMC3511311; doi:10.1371/journal.pone.0050870)
Supplement: Table S4 — List of the organ-specific microRNAs from the AGO4 (ARGONAUTE 4)-related library group. For each organ-specific microRNA selected, the expression level in a specific organ (highlighted by different background colors) should be three times or much higher than the other two or three organs. All the high-throughput sequencing data sets were retrieved from GEO (Gene Expression Omnibus; http://www.ncbi.nlm.nih.gov/geo/) [68]: AGO4_Flower (GSM707686), AGO4_Leaf (GSM707687), AGO4_Root (GSM707688), and AGO4_Seedling (GSM707689). The expression levels were shown by normalized read counts (in RPM; reads per million). (PDF) [file pone.0050870.s011.pdf]

miRNA	Sequence	AGO4_Flower	AGO4_Leaf	AGO4_Root	AGO4_Seedling
ath-miR156a	UGACAGAAGAGAGUGAGCAC	8.75	1.03	0.8	39.17
ath-miR156b	UGACAGAAGAGAGUGAGCAC	8.75	1.03	0.8	39.17
ath-miR156c	UGACAGAAGAGAGUGAGCAC	8.75	1.03	0.8	39.17
ath-miR156d	UGACAGAAGAGAGUGAGCAC	8.75	1.03	0.8	39.17
ath-miR156e	UGACAGAAGAGAGUGAGCAC	8.75	1.03	0.8	39.17
ath-miR156f	UGACAGAAGAGAGUGAGCAC	8.75	1.03	0.8	39.17
ath-miR157a	UUGACAGAAGAUAGAGAGCAC	4.38	3.34	0	29.26
ath-miR157b	UUGACAGAAGAUAGAGAGCAC	4.38	3.34	0	29.26
ath-miR157c	UUGACAGAAGAUAGAGAGCAC	4.38	3.34	0	29.26
ath-miR159a	UUUGGAUUGAAGGGAGCUCUA	328.98	97.75	127.44	96.03
ath-miR161.2	UCAAUGCAUUGAAAGUGACUA	99.45	11.06	13.89	50.96
ath-miR162a	UCGAUAAACCUCUGCAUCCAG	25.04	4.37	8.55	5.66
ath-miR162b	UCGAUAAACCUCUGCAUCCAG	25.04	4.37	8.55	5.66
ath-miR163	UUGAAGAGGACUUGGAACUUCGAU	173.37	101.35	51.56	57.57
ath-miR164a	UGGAGAAGCAGGGCACGUGCA	14.83	1.03	6.14	3.78
ath-miR164b	UGGAGAAGCAGGGCACGUGCA	14.83	1.03	6.14	3.78
ath-miR165a	UCGGACCAGGCUUCAUCCCCC	267.95	28.81	158.97	82.34
ath-miR165b	UCGGACCAGGCUUCAUCCCCC	267.95	28.81	158.97	82.34
ath-miR166a	UCGGACCAGGCUUCAUUCCCC	966.77	87.72	299.5	213.29
ath-miR166b	UCGGACCAGGCUUCAUUCCCC	966.77	87.72	299.5	213.29
ath-miR166c	UCGGACCAGGCUUCAUUCCCC	966.77	87.72	299.5	213.29
ath-miR166d	UCGGACCAGGCUUCAUUCCCC	966.77	87.72	299.5	213.29
ath-miR166e	UCGGACCAGGCUUCAUUCCCC	966.77	87.72	299.5	213.29
ath-miR166f	UCGGACCAGGCUUCAUUCCCC	966.77	87.72	299.5	213.29
ath-miR166g	UCGGACCAGGCUUCAUUCCCC	966.77	87.72	299.5	213.29
ath-miR167a	UGAAGCUGCCAGCAUGAUCUA	156.83	5.14	7.21	56.39
ath-miR167b	UGAAGCUGCCAGCAUGAUCUA	156.83	5.14	7.21	56.39
ath-miR171a	UGAUUGAGCCGCGCCAAUAUC	27.72	0.26	0	5.43
ath-miR172a	AGAAUCUUGAUGAUGCUGCAU	165.1	56.85	41.68	18.4
ath-miR172b	AGAAUCUUGAUGAUGCUGCAU	165.1	56.85	41.68	18.4
ath-miR172c	AGAAUCUUGAUGAUGCUGCAG	110.39	2.06	33.4	0.47
ath-miR172d	AGAAUCUUGAUGAUGCUGCAG	110.39	2.06	33.4	0.47
ath-miR319a	UUGGACUGAAGGGAGCUCCCU	211.54	12.09	6.68	20.76
ath-miR319b	UUGGACUGAAGGGAGCUCCCU	211.54	12.09	6.68	20.76
ath-miR390a	AAGCUCAGGAGGGAUAGCGCC	466.61	21.09	45.69	94.38
ath-miR390b	AAGCUCAGGAGGGAUAGCGCC	466.61	21.09	45.69	94.38
ath-miR393a	UCCAAAGGGAUCGCAUUGAUCC	21.88	0.51	1.6	9.91
ath-miR393b	UCCAAAGGGAUCGCAUUGAUCC	21.88	0.51	1.6	9.91
ath-miR394a	UUGGCAUUCUGUCCACCUCC	70.03	1.54	2.4	4.72
ath-miR394b	UUGGCAUUCUGUCCACCUCC	70.03	1.54	2.4	4.72
ath-miR396b	UUCCACAGCUUUCUUGAACUU	112.58	14.15	35.8	39.17
ath-miR400	UAUGAGAGUAUUAUAAGUCAC	7.29	4.12	10.69	38.93
ath-miR408	AUGCACUGCCUCUUCCCUGGC	25.29	4.37	2.67	627.14
ath-miR447a.2	UAUGGAAGAAAUUGUAGUAUU	35.74	2.57	2.4	2.36
ath-miR780.2	UUCUUCGUGAAUAUCUGGCAU	39.15	0.26	0	0.24
ath-miR822	UGCGGGAAGCAUUUGCACAUG	1.22	7.97	12.02	1.18
ath-miR829.1	AGCUCUGAUACCAAAUGAUGGAAU	12.89	39.1	334.23	25.01
ath-miR846	UUGAAUUGAAGUGCUUGAAUU	0.49	3.34	17.9	1.18
